# EGFR and MMP-9 are associated with neointimal hyperplasia in systemic-to-pulmonary shunts in children with complex cyanotic heart disease

**DOI:** 10.1007/s00335-023-09982-3

**Published:** 2023-03-03

**Authors:** Philip Kottmann, Katja Eildermann, Sarala Raj Murthi, Julie Cleuziou, Julia Lemmer, Keti Vitanova, Maria von Stumm, Luisa Lehmann, Jürgen Hörer, Peter Ewert, Matthias Sigler, Rüdiger Lange, Harald Lahm, Martina Dreßen, Peter Lichtner, Cordula M. Wolf

**Affiliations:** 1 Department of Congenital Heart Defects and Pediatric Cardiology, German Heart Center Munich, Technical University of Munich, School of Medicine & Health, Lazarettstrasse 36, 80636 Munich, Germany; 2 Department of Congenital and Pediatric Heart Surgery, German Heart Center Munich, Technical University of Munich, School of Medicine & Health, Munich, Germany; 3 Division of Congenital and Pediatric Heart Surgery, University Hospital of Munich, Ludwig-Maximilian University Munich, Munich, Germany; 4 Institute for Translational Cardiac Surgery (INSURE), German Heart Center Munich, Technical University of Munich, School of Medicine & Health, Munich, Germany; 5 Department of Pediatrics and Adolescent Medicine–Paediatric Cardiology, Intensive Care Medicine and Pneumology, University Medical Center, Goettingen, Germany; 6 Department of Cardiovascular Surgery, German Heart Center Munich, Technical University of Munich, School of Medicine & Health, Munich, Germany; 7 DZHK (German Centre for Cardiovascular Research), Partner Site Munich Heart Alliance, Munich, Germany; 8 Institute of Human Genetics, Helmholtz Centrum Munich, German Research Center for Environmental Health (GmbH), Neuherberg, Germany

## Abstract

Systemic-to-pulmonary shunt malfunction contributes to morbidity in children with complex congenital heart disease after palliative procedure. Neointimal hyperplasia might play a role in the pathogenesis increasing risk for shunt obstruction. The aim was to evaluate the role of epidermal growth factor receptor (EGFR) and matrix-metalloproteinase 9 (MMP-9) in the formation of neointimal within shunts. Immunohistochemistry was performed with anti-EGFR and anti-MMP-9 on shunts removed at follow-up palliative or corrective procedure. Whole-genome single-nucleotide polymorphisms genotyping was performed on DNA extracted from patients´ blood samples and allele frequencies were compared between the group of patients with shunts displaying severe stenosis (≥ 40% of lumen) and the remaining group. Immunohistochemistry detected EGFR and MMP-9 in 24 of 31 shunts, located mainly in the luminal area. Cross-sectional area of EGFR and MMP-9 measured in median 0.19 mm^2^ (IQR 0.1–0.3 mm^2^) and 0.04 mm^2^ (IQR 0.03–0.09 mm^2^), respectively, and correlated positively with the area of neointimal measured on histology (*r* = 0.729, *p* < 0.001 and *r* = 0.0479, *p* = 0.018, respectively). There was a trend of inverse correlation between the dose of acetylsalicylic acid and the degree of EGFR, but not MMP-9, expression within neointima. Certain alleles in epidermal growth factor (EGF) and tissue inhibitor of metalloproteinases 1 (TIMP-1) were associated with increased stenosis and neointimal hyperplasia within shunts. EGFR and MMP-9 contribute to neointimal proliferation in SP shunts of children with complex cyanotic heart disease. SP shunts from patients carrying certain risk alleles in the genes encoding for EGF and TIMP-1 displayed increased neointima.

**Supplementary Information:**

The online version contains supplementary material available at 10.1007/s00335-023-09982-3.

## Introduction

Systemic-to-pulmonary (SP) shunts are artificial polytetrafluoroethylene (PTFE) grafts implanted in children with complex congenital cyanotic heart defects in order to secure the pulmonary perfusion between Norwood stage I and II palliation, corrective surgery or another follow-up procedure. Interstage mortality of these infants remains high and is directly influenced by the patency of these grafts, which are at risk of obstruction due to neointimal formation or thrombosis (Agarwal et al. 2017; Fenton et al. 2003; Monagle 2005; Vitanova et al. 2019). Neointimal hyperplasia is associated with interstage morbidity in children with complex and congenital heart disease (Kottmann et al. 2022) and is caused by the foreign body response, which involves the infiltration of immune cells, the formation of granulation tissue, and the generation of a fibrous capsule around foreign material. The resulting pathological vascular remodeling and tissue deposition within the shunt lead to gradual shunt dysfunction (Lee and Ul Haq 2015).

Epidermal growth factor receptor (EGFR) and matrix-metalloproteinase 9 (MMP-9) are proteins linked to hyperproliferative diseases such as bronchial carcinoma (Gong et al. 2016; Pao et al. 2005) and are associated with the formation of neointimal hyperplasia in numerous studies (Newby 2005; Sanchez-Guerrero et al. 2013). In separate rat and porcine study models, targeted suppression of EGFR (Chan et al. 2003; Trieu et al. 2000) and MMP-9 (Song et al. 2020) has been demonstrated to significantly reduce the formation of neointimal hyperplasia after vascular injury.

Since the pathophysiology of neointimal hyperplasia in SP shunts of infants with complex congenital heart disease remains largely unknown, we aimed to identify the role of EGFR and MMP-9 in the formation of neointima in SP shunts. Previous research has suggested that acetylsalicylic acid (ASS) might play a role in neointimal growth (Kottmann et al. 2022). Therefore, we also examined the possible association between ASA and expression of the two proteins.

EGFR and MMP-9 were quantified by immunohistochemistry (IHC) in SP shunts explanted during follow-up surgery and the positively stained area was correlated with the area of neointimal hyperplasia. In addition, we aimed to discover alleles of single-nucleotide polymorphisms (SNPs) in related genes that may predispose to the formation of neointimal hyperplasia.

Currently, there is no drug therapy addressing neointimal hyperplasia in SP shunts of children with cyanotic heart disease. The findings of this study shall contribute to understand the parts of the complex pathogenesis of shunt malfunction and shall provide a basis to identify potential therapeutics to prevent the formation of neointimal hyperplasia in SP shunts.

## Methods

### Patients and patient material

SP shunts were fixed and stored in formalin immediately after explantation and until further processing. Peripheral blood was collected from respective patients and stored in the cardiovascular biobank at the German Heart Center Munich (KaBi-DHM).

Demographic and clinical data were collected from medical charts at the timepoints date of birth, shunt implantation, and shunt takedown.

### Histological processing of the shunt material

Explanted, formalin-fixed shunts were cut into two pieces. One piece was embedded in synthetic resin (methyl methacrylate, Technovit 9100, Kulze Wehrheim, Germany) as described (Quentin et al. 2009); the other in paraffin after dehydration. Standard protocols were applied for the preparation and histochemical stainings Richardson, Hematoxylin/Eosin, and Elastica van Gieson (Mulisch 2010; Quentin et al. 2009). IHC stainings were performed as previously described (Quentin et al. 2009) and optimized for each antibody (Online supplementary, Table 1).

Dotslide system (Olympus) was used to digitize all stained sections and the Olyvia software (Olympus Center Valley, PA, USA) for visualization.

### Evaluation of the tissue

ImageJ (ImageJ, US National Institutes of Health, Bethesda, MD, USA) was used for histopathological quantification. For the determination of neointimal hyperplasia, the tissue protruding into the lumen of the shunt and the area of thrombi was measured manually and the greatest value was used for analysis. The ratio of the cross-sectional area of neointimal hyperplasia divided by the cross-sectional area of the potential shunt lumen amounts to the relative shunt stenosis.

$${\text{Shunt}}\,{\text{stenosis}}\left[ \% \right] = {{{\text{Neo}}\,{\text{intima}}\left[ {{\text{mm}}^{{2}} } \right]} \mathord{\left/ {\vphantom {{{\text{Neo}}\,{\text{intima}}\left[ {{\text{mm}}^{{2}} } \right]} {{\text{Potential}}\,{\text{shunt}}\,{\text{lumen}}\,\left[ {{\text{mm}}^{{2}} } \right]}}} \right. \kern-0pt} {{\text{Potential}}\,{\text{shunt}}\,{\text{lumen}}\,\left[ {{\text{mm}}^{{2}} } \right]}}$$

EGFR and MMP-9 were analyzed by measuring the stained area using the color threshold function of ImageJ. Stained area was then related to the area of neointimal tissue.

### Identification of single-nucleotide polymorphisms coding for neointimal hyperplasia

Blood samples of the affected patients were obtained after written consent by the parents. High-quality DNA was purified using the DNeasy Blood and Tissue Kit (Qiagen; Hilden, Germany) according to the manufacturer’s recommendation. Concentration was determined by NanoDrop™ spectrophotometer and the integrity of genomic DNA was confirmed by gel electrophoresis. DNA was analyzed for SNPs using the Infinium Omniexpress Kit (Illumina, Inc., San Diego, CA, USA). The Infinium high-density DNA analysis solution combines the Infinium assay with BeadChip microarrays to perform a large genome-wide query of genetic DNA variations. Standard genome-wide SNP genotyping was performed.

Bioinformatic analysis of the Infinium BeadChip data was carried out using PLINK (PUTTY Link). Two categories of clusters were formed based on the shunt stenosis caused by neointimal hyperplasia (Group 1 = 0–39.9% lumen stenosis (*n* = 26); Group 2 =  ≥ 40% lumen stenosis (*n* = 5)). A cut-off of 40% was chosen based on a preliminary analysis suggesting clinical relevance in that a shunt stenosis of greater than 40% was associated with an increased risk for cardiac interventions, such as balloon dilatation or shunt stenting (Kottmann et al. 2022). PLINK calculated the SNPs that significantly differed in the two groups (*p*-value < 0.01, Chi’s Square).

Significant SNPs were selected semi-quantitatively based on the following criteria: statistical significance in PLINK cluster analysis, gene associations with EGFR and MMP-9 pathways, and previous presence of the individual SNPs in scientific publications.

SNP data were analyzed with the web-based SNPnexus tool (www.snp-nexus.org), which provides bioinformatic data from different genome databases and assigns them to the entered SNP query, enabling to allocate overlaps with structural DNA elements to predict functional gene protein consequences and to retrieve links with previous genetic disease studies (Chelala et al. 2008; Dayem Ullah et al. 2012, 2013; Dayem Ullah et al. 2018). GRCh/hg19 was used for the analysis of the human genome. Allele frequencies of the study population were stratified and genotypes linked with neointimal formation and shunt stenosis were determined as alleles in association with neointimal hyperplasia and relative shunt stenosis.

### Statistics

SPSS Version 27 was used for all statistical analysis. Data are provided as median and interquartile range (IQR). Mann–Whitney *U* tests were applied for nominal variables with two categories for non-parametric analysis. Kruskall–Wallis test was used for more than two categories. To determine the strength of the association between two variables, Spearman's Rho non-parametric test was performed. For the comparison of nominal variables, Chi’s Square and Fisher’s exact tests were used, respectively. A multivariate linear regression was conducted to examine the association between EGFR [mm^2^], MMP-9 [mm^2^], and ASA dosage per bodyweight [mg/kg/BW] and their impact on neointimal hyperplasia. *p*-values are stated raw unless a number below *p* = 0.001, in which case they are corrected to *p* < 0.001.

**Fig. 1:**
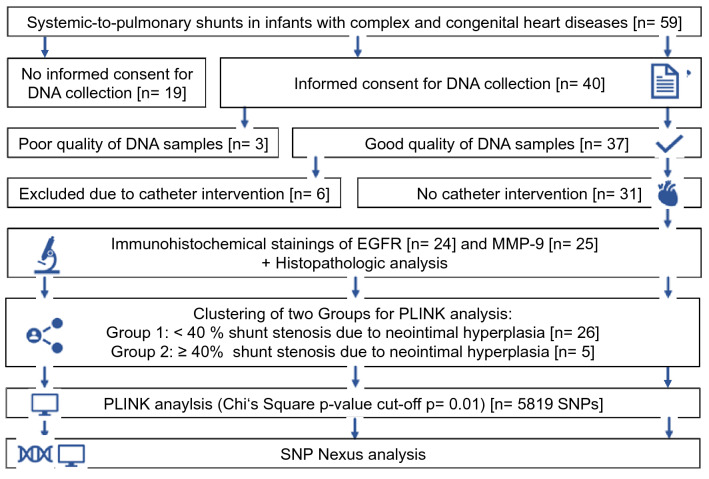
Visual abstract. Flow chart depicting the study design; *EGFR* epidermal growth factor receptor; *MMP-9* matrix-metalloproteinase-9; *SNP* single-nucleotide polymorphism; *PLINK* PUTTY Link

An overview of the study design can be found in Fig. 1.

## Results

### Patient characteristics

SP shunts and blood for DNA extraction were collected between February 2011 and August 2016. 40 patients with Caucasian background that underwent palliative correction using a PTFE shunt gave written consent to the study and were initially included into the study. Three samples had to be excluded due to poor quality of extracted the DNA. Another six patients were excluded because of previous stent placement or balloon dilatation prior to shunt explantation, which could have influenced neointimal proliferation. SP shunt neointimal hyperplasia, SNP analysis from patient blood DNA, and clinical data interpretation were analyzed from 31 patients (18 (58%) male), as outlined in Fig. 1. The most common underlying structural heart disease included hypoplastic left heart syndrome in 15 patients (48%) and pulmonary atresia with and without ventricular septal defect in 7 patients (23%) (Table 1). At the time of implantation, children were in median 9 (IQR 7–14) days old. SP shunt was explanted after a median of 117 (IQR 85–198) days due to a Glenn procedure, corrective surgery, or shunt revision. The diameter of the implanted shunts was 3 mm in 3 (9.7%), 3.5 mm in 17 (54.8%), 4 mm in 2 (6.6%), and 5 mm in 9 (29%) patients (Table 1).

**Table 1:** Demographic parameters

Clinical parameters	Characters	No. (%)/median [range]
Gender	Male	18 (58.1%)
	Female	13 (41.9%)
Diagnosis	HLHS	15 (48.4%)
	PA + VSD	6 (19.4%)
	PA	1 (3.2%)
	TOF	3 (9.7%)
	DORV	1 (3.2%)
	TA	2 (6.5%)
	TGA	1 (3.2%)
	Other	2 (6.5%)
Shunt type	mBTTS	21 (67.7%)
	RVPA	7 (22.6%)
	CS	3 (9.7%)
Shunt diameter [mm]	3	3 (9.7%)
	3.5	17 (54.8%)
	4	2 (6.5%)
	5	9 (29%)
Birth weight [grams]		3060 [2650–3340]
Age at implantation [days]		9 [7–14]
Days of implantation [days]		117 [85–198]
ASA dosage [mg/kg/BW]		2.50 [2.09–2.98]

No. (%), number of patients (percentage)

*HLHS* hypoplastic left heart syndrome; *TOF* tetralogy of Fallot; *PA + VSD* pulmonal atresia with ventricular septal defect; *PA* pulmonal atresia; *TGA* transposition of great arteries; *DORV* double outlet right ventricle; *TA* tricuspidal atresia; *mBTTS* modified Blalock-Taussig-Thomas Shunt; *RVPA* right-ventricle-to-pulmonary-artery shunt; *CS* central shunt; *ASA dosage [mg/kg/BW]* ASA dosage per kilogram bodyweight at the time of shunt removal

Platelet inhibition was performed in the form of ASA dosed to efficacy based on thrombocyte-functioning test prior to stage I palliation and measured in median 2.5 mg /kg/BW [2–3 mg/kg/BW] at the time of shunt removal.

### Morphometric measurements of shunt lumen and neointimal hyperplasia

**Fig. 2:**
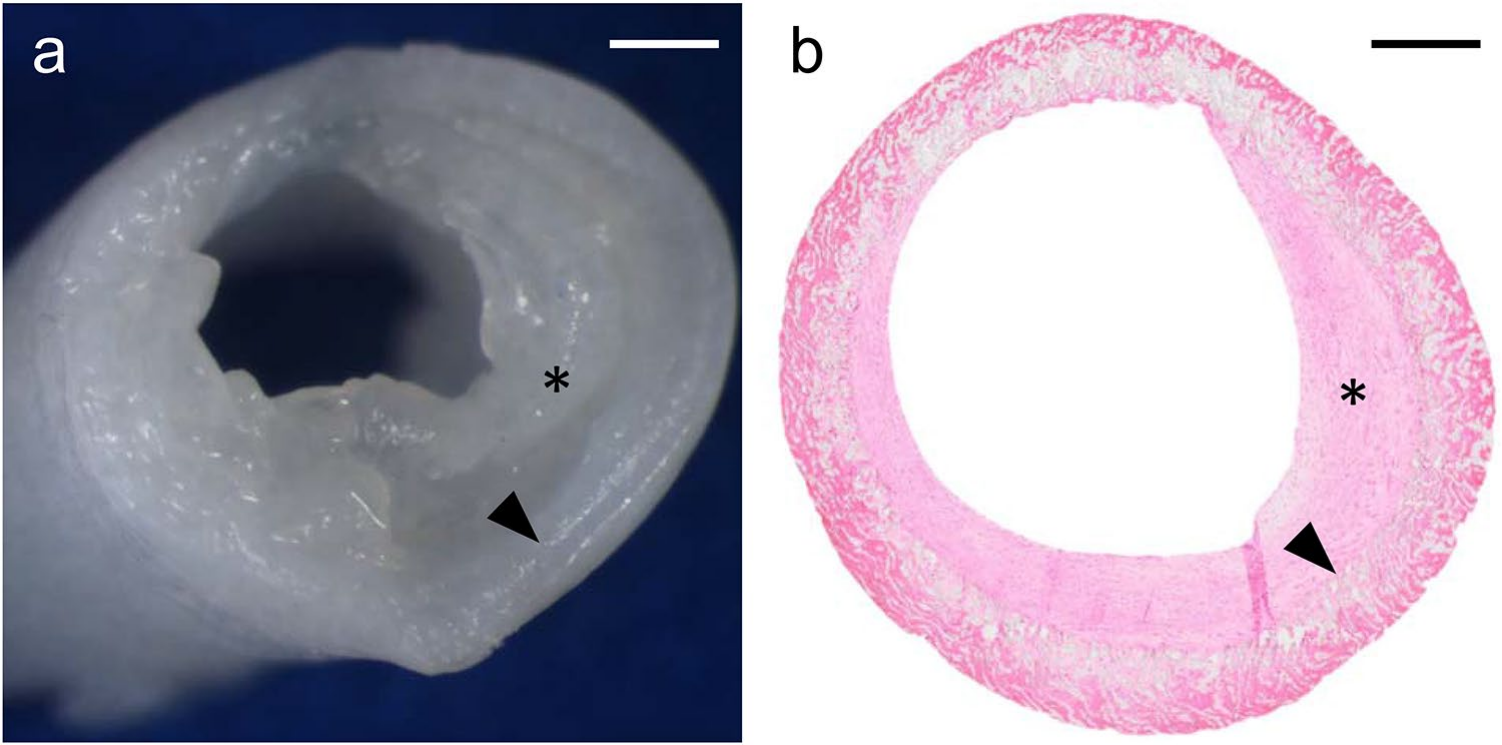
Macroscopical and microscopical shunt image. **a** macroscopical image of a systemic-to-pulmonary PTFE shunt; scale bar 500 µm. **b** Hematoxylin/Eosin (HE) image showing the cross-section of the identical shunt; scale bar 500 µm. In both panels, the star [*] depicts the neointimal formation and the [triangle] shows the border between PTFE material and neointimal hyperplasia

Neointimal hyperplasia occurred in 20 out of 31 shunts (65%). The median of shunt stenosis was 17% (IQR 2–34%) and greater than 40% in five patients. The area of neointima was in median 0.84 mm^2^ (IQR 0.14–2.54 mm^2^) (Table 2, Asterix in Fig. 2). A comparison of macroscopy and microscopy of neointimal hyperplasia is shown in Fig. 2.

**Table 2:** Histopathological and immunohistochemical parameters

Histopathological parameters	Categories	No. (%)/median [range]
Neointimal hyperplasia	Yes	20 (64.5%)
	No	11 (35.5%)
Area of neointimal hyperplasia [mm^2^]		0.84 [0.14–2.58]
Shunt stenosis due to neointimal hyperplasia [%]		16.93 [2.16–34]
Thrombi	Yes	12 (61.3%)
	No	19 (38.7%)
Area of thrombi [mm^2^]		0.13 [0.08–0.48]
Shunt stenosis due to thrombi [%]		2.07 [0.41–6.46]
EGFR [mm^2^]		0.19 [0.1–0.3]
MMP-9 [mm^2^]		0.04 [0.03–0.09]

No. (%), number of patients (percentage)

*EGFR* epidermal growth factor receptor; *MMP-9* matrix-metalloproteinase-9; *mm* millimeter; *mm*^2^ square millimeter

### Immunohistochemical detection and measurement of EGFR and MMP-9

Due to insufficient quality, seven samples of the IHC for EGFR and six samples for MMP-9 were not included in the analysis.

**Fig. 3:**
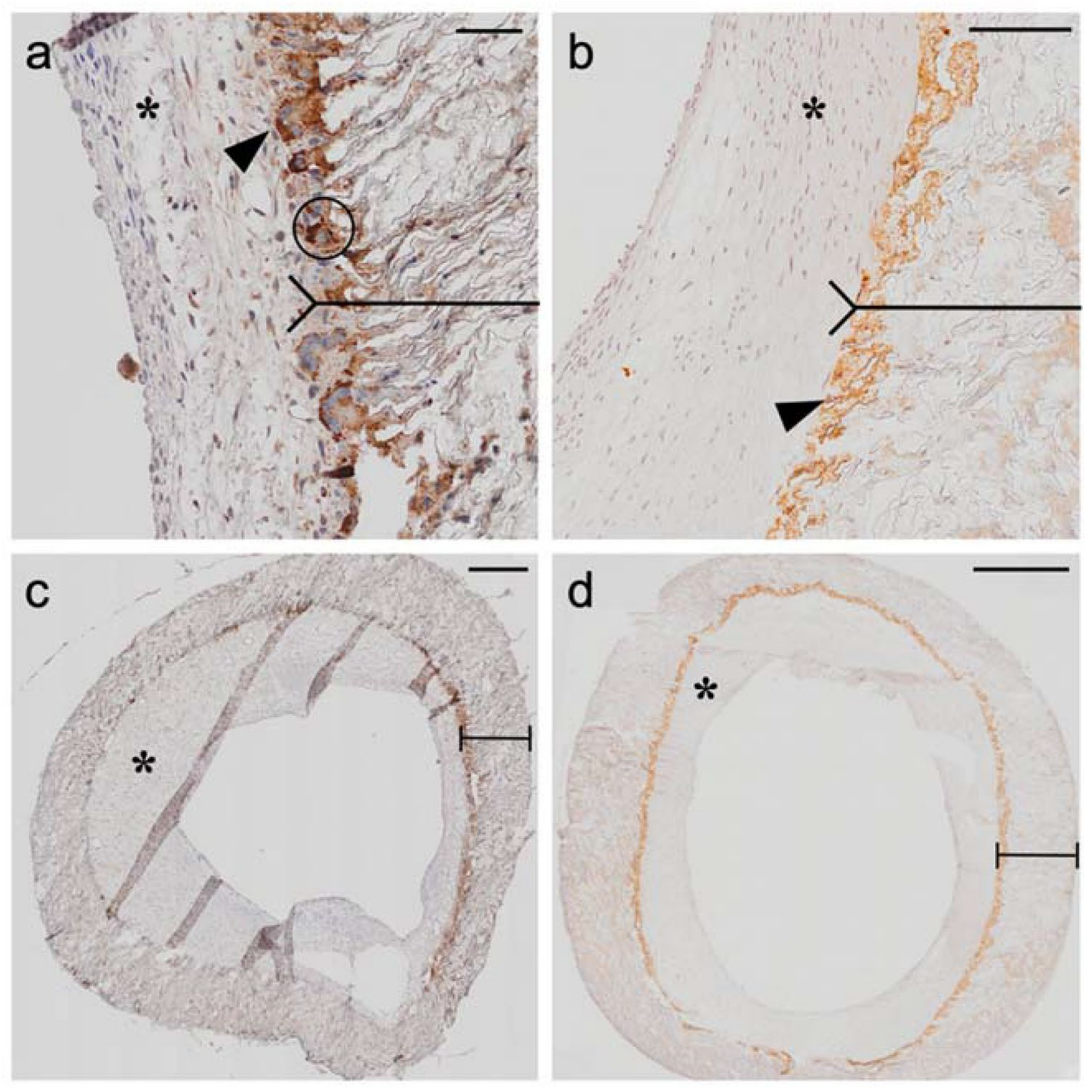
Immunohistochemical staining of EGFR and MMP-9. Cross-sectional images of systemic-to-pulmonary shunts implanted in children with complex and congenital heart disease. In section **a** and **b**, the reverse arrow points to the border between PTFE material and neointimal formation, whereas the star [*] displays neointimal hyperplasia. Scale bar **a** = 20 µm, **b** = 50 µm. In section **c** and **d**, the double-ending line represents the PTFE material. The triangles point toward the targeted proteins of the immunohistochemical staining which resemble MMP-9 in panel **a** and **c** and EGFR in panel **b** and **d**. Scale bars in **c**–**d** = 500 µm. Both stainings demonstrate that the proteins are located mainly in the luminal area of the PTFE material, which is illustrated by the line in panel **a** and **b**. Morphology identifies stained cells as macrophages (Circle, panel **a**). *EGFR* epidermal growth factor receptor; *MMP-9* matrix-metalloproteinase-9

**Fig. 4:**
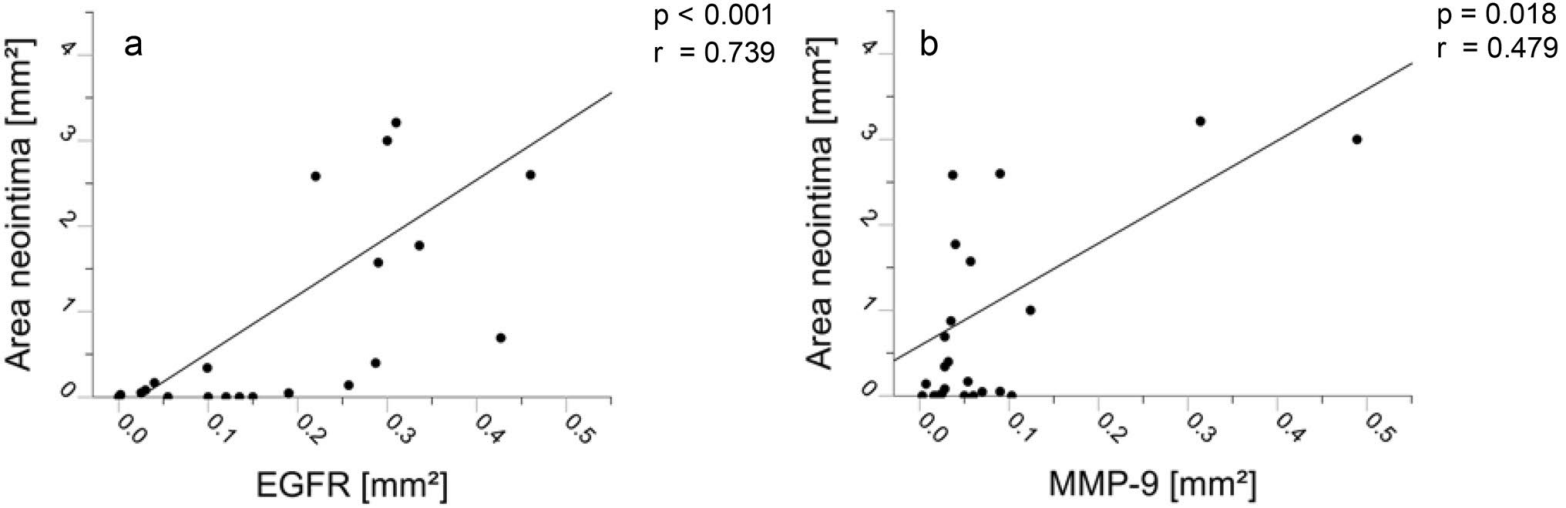
EGFR [mm^2^] and MMP-9 [mm^2^] are associated with neointima hyperplasia [mm^2^]. Dot-Plots. Spearman correlations showing the correlations between the variables MMP-9 [mm^2^] and EGFR [mm^**2**^] and neointimal hyperplasia [mm^**2**^], [mm^**2**^] = square millimeters; *r* correlation-coefficient; *EGFR* epidermal growth factor receptor; *MMP-9* matrix-metalloproteinase-9

**Fig. 5:**
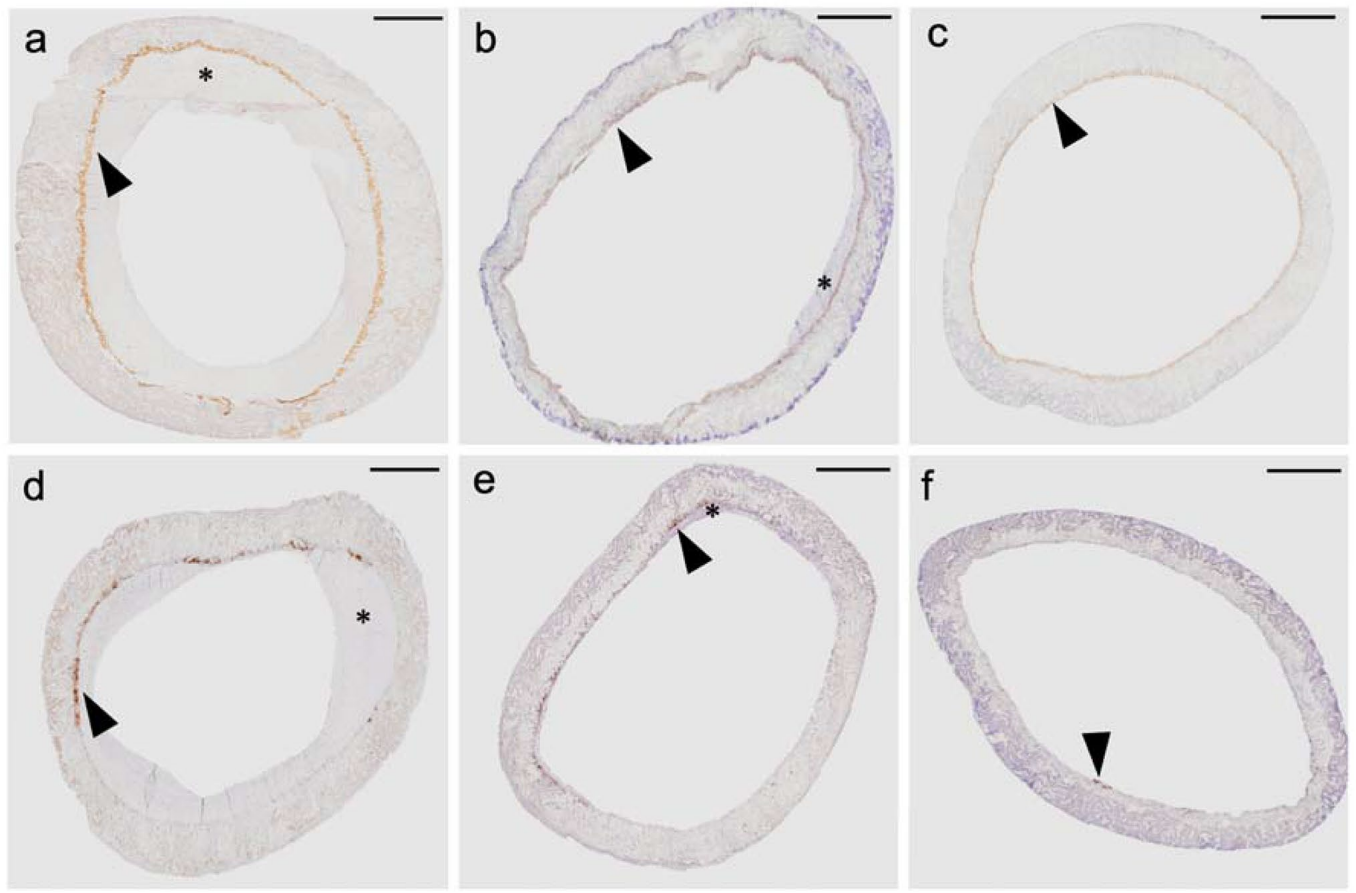
EGFR and MMP-9. Comparison of systemic-to-pulmonary shunts with respect to neointimal proliferation and immunohistochemical (IHC) stainings of EGFR (panel **a**, **b**, **c**) and MMP-9 (**d**, **e**, **f**). Panelsl (**a**, **d**) show severe neointimal proliferation and greater distribution of the IHC staining EGFR and MMP-9 compared to panels (**b**, **e**) showing mild and panel (**c**, **f**) presenting zero neointimal proliferation and fewer IHC stainings. The star [*] displays neointimal hyperplasia and the triangles point toward EGFR or MMP-9-positive areas. Scale bar in all panels = 500 µm. *EGFR* epidermal growth factor receptor; *MMP-9* matrix-metalloproteinase-9

The stained area after EGFR detection was in median 0.19 mm^2^ (IQR 0.1–0.3 mm^2^, *n* = 24), per cross-section, that of MMP-9 in median 0.04 mm^2^ (IQR 0.03–0.09 mm^2^, *n* = 23) (Table 2). The area of EGFR and MMP-9 significantly correlated with the area of neointimal hyperplasia (Fig. 3). EGFR and MMP-9 were mainly detected in the luminal region of the PTFE material and showed a ring-like structure (see Fig. 4). Cell morphology was that of macrophages and foreign body giant cells, which partly migrated into the shunt material and assembled between neointima and PTFE biomaterial (Fig. 4). Figure 5 visualizes the correlation from Fig. 3 by showing shunts with severe neointimal proliferation and a greater amount of EGFR and MMP-9 next to those with mild expression and no tissue formation.

There was a trend of negative correlation between the ASA dosage per kilogram of body weight and the cross-sectional area of EGFR (*p* = 0.073, *r* = − 0.381, Spearman-Rho). There was no correlation between ASS dosage and MMP-9 expression (*p* = 0.719, *r* = − 0.077, Spearman-Rho).

### Multivariate regression

In a multivariate regression, we examined the influence of the variables EGFR [mm^2^], MMP-9 [mm^2^], and acetylsalicylic acid (ASA) dosage [mg/kg/BW] on the variable neointimal hyperplasia [mm^2^]. The variables MMP-9 [mm^2^] and EGFR [mm^2^] remained as significant predictors for greater neointimal formation on regression analysis (p = 0.001; adjusted R^2^ = 0.690, online supplementary tables 2–6).

### Single-nucleotide polymorphisms

PLINK analysis from patient DNA resulted in 5819 SNPs that differed significantly between shunts with severe stenosis (> 40%) and shunts with mild to no stenosis (Chi’s Square, *p*-value cut-off = 0.01). Out of these, 2270 SNPs located in protein coding genes and of these, 86 were nonsynonymous, meaning that they alter the amino acid sequence of the encoded protein**.** Remaining SNPs were either located in noncoding intron variants or in the following transcript structures coding variants: 5′-UTR, 5′ upstream, 3′-UTR, 3′ upstream, and 3′ downstream transcript variant isoforms.

Evaluation of all SNPs for their relationship to genes and pathways around EGFR returned the SNPs, rs2237051, rs2298989, and rs2298999 which were all located within the EGF gene that on chromosome 4 between the bases 109970517 and 109980042. Related to MMP-9 was the SNP rs6609533 within the TIMP-1 gene on the X-chromosome at base position 47585887 (Table 4).

**Fig. 6:**
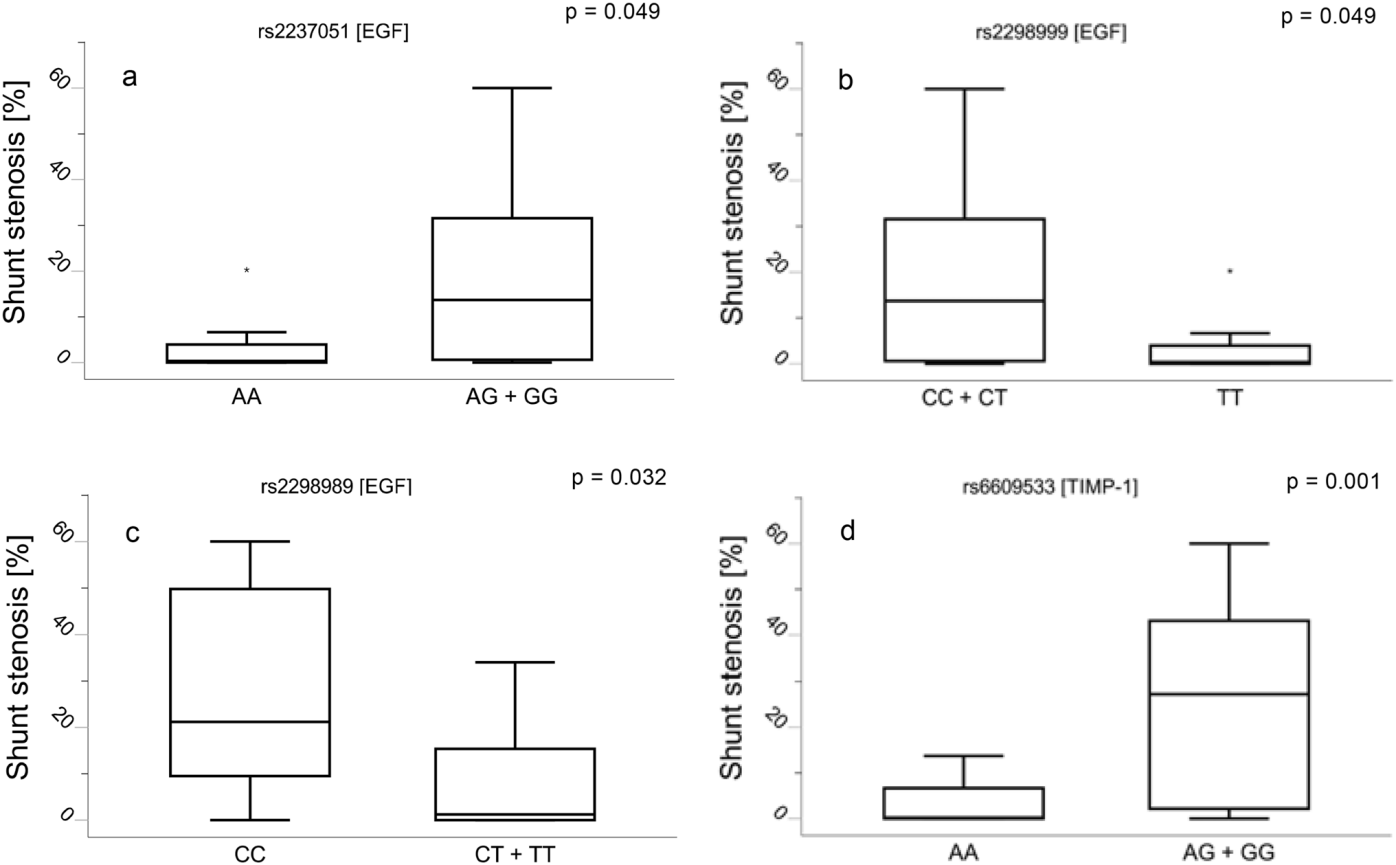
Alleles associated with relative shunt stenosis due to neointimal hyperplasia. Distribution of shunt stenosis and neointimal hyperplasia (*y*-axis [%]) is shown in patients grouped for distinct genotypes. *EGF* epidermal growth factor; *TIMP-1* tissue inhibitor of metalloproteinase-1; *A* adenine; *G* guanine; *C* cytosine; *T* thymine; * extruders

Allele stratification revealed allele “C” in rs2298989 [EGF] and rs2298999 [EGF] to be associated with higher expression in neointimal hyperplasia and shunt stenosis. For the polymorphisms rs2237051 [EGF] and 6609533 [TIMP-1], allele “G” was associated with greater neointima and relative shunt stenosis (Table 3 and Fig. 6).

**Table 3:** Single-nucleotide polymorphisms and allele-specific distribution of neointimal hyperplasia and shunt stenosis in systemic-to-pulmonary PTFE shunts

Gene	ID	REF	ALT	Minor	MAF	Allele	Frequency	Stenosis [%]	p_1_	Neointima [mm^2^]	p_2_
							No. (%)	Median [IQR]		Median [IQR]	
EGF	rs2237051	A	A	G	0.382188	AA	8 (25.8%)	0.34 [0–3.95]	0.049	0.02 [0–0.19]	0.028
						AG	14 (45.2%)	4.73 [0–29.13]	0.936	0.28 [0–1.57]	0.872
						GG	9 (29%)	17.04 [2.16–48.67]	0.071	1 [0.06–3]	0.053
EGF	rs2298999	T	C	T	0.332268	CC	11 (35.5%)	17.04 [0.17–48.67]	0.087	1 [0.03–3]	0.066
						CT	12 (38.7%)	4.73 [0.51–23.09]	0.935	0.28 [0.04–1.26]	0.870
						TT	8 (25.8%)	0.34 [0–3.95]	0.49	0.02 [0–0.19]	0.028
EGF	rs2298989	T	C	T	0.357628	CC	8 (25.8%)	21.18 [9.49–49.82]	0.032	1.98 [0.38–3.11]	0.022
						CT	14 (45.2%)	2.1 [0–17.06]	0.601	0.15 [0–0.95]	0.658
						TT	9 (29%)	0.69 [0–6.66]	0.293	0.05 [0–0.34]	0.201
TIMP-1	rs6609533	A	G	G	0.473377	AA	17 (54.8%)	0.17 [0–6.66]	0.001	0.03 [0–0.34]	0.002
						AG	7 (22.6%)	34 [6.63–48.67]	0.01	1.57 [0.4–3.21]	0.019
						GG	7 (22.6%)	25.32 [1.25–42.28]	0.197	1.77 [0.05–2.96]	0.197

Each single-nucleotide polymorphism (SNP) and the corresponding overlapping gene with genomic coordinates. Distribution of neointimal hyperplasia [mm^2^] measured in shunts from patients with the respective genotype (e.g., AA; AG; GG) is shown. Additionally, the table shows the reference (REF)-, altered (ALT)- , and minority alleles (MA) with the belonging minor allele frequencies (MAF) of each polymorphism. ID, rs number; REF, reference allele; ALT, observed allele; Minor, minor allele; MAF, minor allele frequency (1000 Genomes); No. (%) patients in percent; p_1_, *p*-value of Mann–Whitney U tests comparing the different genotypes against distribution of shunt stenosis; p_2_, *p*-value of Mann–Whitney *U* tests comparing the different genotypes against the area distribution of neointimal hyperplasia; EGF, epidermal growth factor; TIMP-1, tissue inhibitor of metalloproteinases 1; A, Adenine; G, Guanine; C, Cytosine; T, Thymine; p-value Mann–Whitney-U test comparing one allele frequency with two other frequencies (e.g., AA vs. AG + GG, etc.)

### Functional annotations of selected SNPs by SNPnexus

Due to alternative splicing and thus various transcript isoforms, each SNP can take different forms of transcript modifications. All functional annotations of transcripts are summarized in Table 4. Transcript isoforms of rs2237051 [EGF] and rs6609533 [TIMP-1] are classified as nonsynonymous, leading to a missense variation, which in the first case manifests as an amino acid exchange from methionine to isoleucine and in the second case from threonine to alanine or from threonine to serine. In different transcript variants, rs2237051 [EGF] and rs6609533 [TIMP-1] are considered to be in the 5′ upstream 3′ upstream region, respectively. rs2298999 [EGF] and rs2298989 are located on intronic and non-coding-intronic DNA elements (see online supplementary tables 7–9 for detailed information about each transcript isoform and whole PLINK analysis). SNPnexus analysis can be reproduced by entering the PLINK analysis (online supplementary table 9) in the query function of the web-based tool www.snp-nexus.org.

**Table 4:** Detailed information about respective single-nucleotide polymorphisms (SNPs)

Gene	rs number	Chr: base position	Annotations of transcript isoforms	AA change
*EGF*	rs2237051	4:109980042	Coding nonsyn, 5upstream	M < I
*EGF*	rs2298999	4:109990751	Non coding intronic, intronic	
*EGF*	rs2298989	4:109970517	Non coding intronic, intronic	
*TIMP-1*	rs6609533	X:47585887	Coding nonsyn, 3upstream, intronic, 3utr	T > A|T > S

Transcript modifications resulting in various predicted protein consequences are shown in the “Annotation” column [ENSEMBL]. Due to alternative splicing and thus various transcript isoforms, each SNP can take different forms of transcript modifications. Chr, chromosome number; 3utr, 3′ untranslated region of transcript; 3upstream, within 2 kb upstream of the 3′ end of a transcript; 5upstream, within 2 kb upstream of the 5′ end of a transcript AA change [ENSEMBL], reference amino acid(s)," > ", observed amino acid(s); M, methionine; I, isoleucine; T, threonine; S, serine; EGF, epidermal growth factor; TIMP-1, tissue inhibitor of metalloproteinases 1

## Discussion

Neointimal hyperplasia in systemic-to-pulmonary (SP) shunts has recently been associated with interstage morbidity in children with complex and congenital heart defects (Kottmann et al. 2022). Understanding the complex pathophysiology of neointimal formation could identify potential drug targets to reduce cardiovascular interventions in SP shunts in this critical ill population. To our knowledge, this is the first study to examine the expression of EGFR and MMP-9 and to assess the presence of risk alleles for neointimal formation in SP shunts from children with complex cyanotic congenital heart disease. Neointimal hyperplasia is subject to a multifactorial genesis: initial endothelial dysfunction and activation of the connected vessel are accompanied by platelet activation, aggregation, and thrombus formation (Angelini et al. 1990; Bonatti et al. 2004; Zain et al. 2020). This is followed by leukocyte and macrophage recruitment (Angelini et al. 1990) causing thrombus degradation, which in turn results in fibrosis. Metalloproteinase-induced extracellular matrix degradation leads to facilitated migration and proliferation of SMCs and monocytes, causing enrichment of these cells in the shunt lumen (Rotmans et al. 2004). Under certain conditions, these migrated SMCs undergo a phenotype switch from the contractile-silent into the secretory-proliferative phenotype aggravating the hyperproliferation inside the shunt lumen (Campbell and Campbell 1990; Thyberg et al. 1995). The final consequence is an accumulation of SMCs and myofibroblasts in the shunt lumen depositing extracellular matrix and collagen, which leads to gradual progression of shunt stenosis to a total shunt occlusion (Casscells 1992; Clowes and Reidy 1991).

**Fig. 7:**
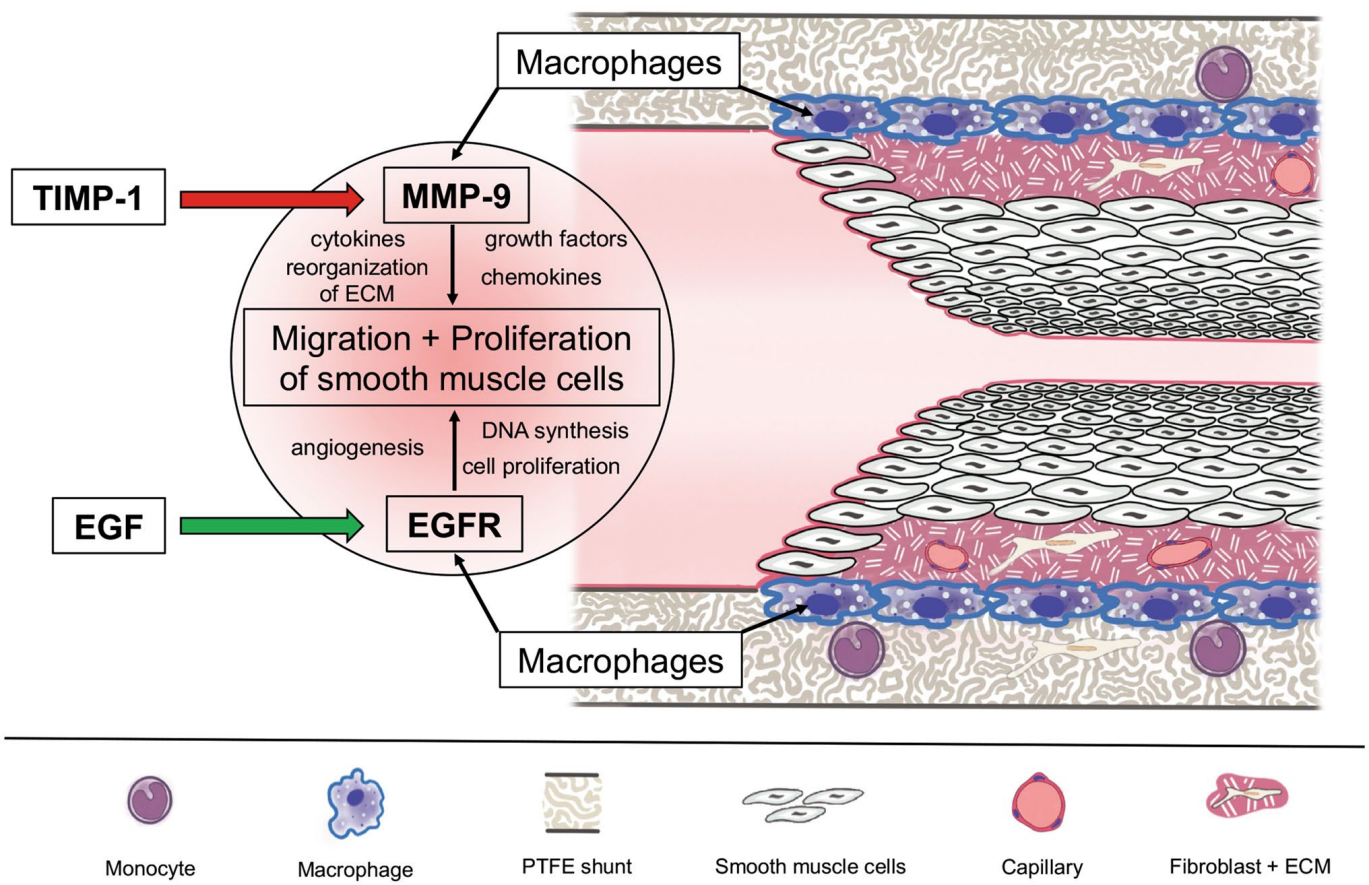
Possible pathways influencing neointimal hyperplasia in systemic-to-pulmonary shunts EGFR and its ligand EGF play a crucial role in the regulation of cell proliferation and differentiation. Upregulation of the receptor was associated with the growth of neointimal hyperplasia. By binding to EGFR on the cell surface of, e.g., macrophages, tyrosine kinase cascade activity stimulates the expression of genes involved in DNA synthesis, cell proliferation, and angiogenesis. Therefore, upregulation of this pathway by, e.g., single-nucleotide polymorphisms could promote the enhanced formation of neointimal hyperplasia. MMP-9 secreted from macrophages promotes the migration and proliferation of smooth muscle cells and monocytes by the degradation and reorganization of extracellular matrix and the regulation of cytokines, chemokines, and growth factors. TIMP-1 serves as a strong inhibitor of the metalloproteinases, so that a loss of function of TIMP-1, as indicated in our SNP analysis, could lead to a predominance of MMP-9 and the formation of neointimal hyperplasia. *EGF* epidermal growth factor; *TIMP-1* tissue inhibitor of metalloproteinase-1; *EGFR* epidermal growth factor receptor; *MMP-9* matrix-metalloproteinase-9; *ECM* extra cellular matrix

The aim of the study was to quantify EGFR and MMP-9 in explanted SP shunts of children with complex cyanotic heart disease and to identify the risk alleles in related genes which might impact signaling pathways promoting the formation of neointimal hyperplasia. A schematic representation of the possible pathophysiology and pathways of the formation of neointimal hyperplasia in SP shunts are shown in Fig. 7.

### EGF/EGFR

EGF plays a major role as growth factor in the regulation of cell proliferation and differentiation (Sanchez-Guerrero et al. 2013). Its receptor EGFR is expressed by numerous cells such as vascular smooth muscle cells, macrophages, and endothelial cells (Bagheri-Yarmand et al. 2000; Lamb et al. 2004; Tamura et al. 2001). EGF binds with high affinity to its membrane-bound receptor that is located on the cell surface and triggers intrinsic tyrosine kinase activity (Johnson et al. 2004). The tyrosine kinase initiates a signaling cascade influencing cell metabolism and thus stimulates the expression of genes enhancing DNA synthesis, cell proliferation, and angiogenesis (Bagheri-Yarmand et al. 2000; Johnson et al. 2004; Okada et al. 1997).

Dysregulation of this tightly balanced system by overexpression, amplification, or mutation has been associated with hyperproliferative diseases such as cancer and the presence of neointimal hyperplasia (Chan et al. 2003; Huang and Harari 1999; Olayioye et al. 2000; Trieu et al. 2000).

Trieu et al. and Chan et al. demonstrated in separate rodent models significantly less restenosis from neointimal hyperplasia by inhibition of EGFR with an EGFR inhibitor (EGFR genistein) and by blocking the receptor with a monoclonal IgG antibody after carotid artery injury (Chan et al. 2003; Trieu et al. 2000).

Nicholl et al. showed that blocking EGFR in rat aortic SMCs suppresses not only cell proliferation but also migration of vascular smooth muscle cells in vitro (Nicholl et al. 2005).

In a recent study, Foth et al. found an upregulation of EGFR by 2.8-fold in the thickened walls of bioprosthetic valved conduits and demonstrated that the interaction of macrophages and EGFR in particular might play a role in conduit stenosis (Foth et al. 2021). In line with other studies, they report that EGFR-positive macrophages possibly reflect an activated state responsible for inflammatory processes and thus the formation of neotissue (Hardbower et al. 2017; Hoyer et al. 2019). A blockade of EGFR reduces the chronic inflammatory process, potentially resulting in decreased stenosis in conduits (Foth et al. 2021; Tang et al. 2019).

In our study, the cross-sectional amount of EGFR significantly correlated with the area of neointimal hyperplasia and was an independent predictor for its formation in multivariate analysis. Additionally, we identified SNPs with risk genotypes of *EGF* in patients with severe shunts stenosis. rs2237051 leads to a missense variant and amino acid change whereas rs22989999 and rs229898989 lead to intronic alterations, which could have consequences on protein function or expression. Our data provide evidence for the integrity of the EGFR signaling pathway in the pathophysiology of neointimal formation in SP shunts from children with complex congenital heart disease. Suppression of neointimal hyperplasia by targeted blockade of the EGFR signaling pathway as shown in preclinical models with distinct pathophysiologies (Chan et al. 2003; Nicholl et al. 2005; Trieu et al. 2000) might serve as a possible therapeutic option for children with cyanotic heart disease requiring placement of SP shunts, specifically in the smallest size shunts given their high risk for obstruction (Wells et al. 2005).

### MMP-9/TIMP-1

MMP-9 degrades proteins of the extracellular matrix directly and stimulates cytokines and chemokines to regulate tissue remodeling (Yabluchanskiy et al. 2013). Invasion of monocytes, neovascularization, and neointimal growth all require the protease activity of MMP-9, identifying this enzyme as a possible therapeutic target to suppress intimal proliferation (Watanabe et al. 2018). In a porcine model, the use of stents eluting the MMP-9 inhibitor “GM6001” showed potent inhibition of intimal hyperplasia, an increase in luminal area, and no obvious thrombosis in explanted arteries (Song et al. 2020). On the basis of our immunohistochemical staining, we hypothesize that MMP-9 is also involved in the pathophysiology of neointimal proliferation in SP shunts.

TIMP-1 is a strong inhibitor of the matrix-metalloproteinases. In the form of a cytokine, TIMP-1 has been linked to numerous effects such as cell growth, cell differentiation, apoptosis, and angiogenesis. TIMP-1 was also found to be involved in the pathways “Interleukin Signaling” and “Signaling by IL-10,” which both play a crucial role in the recruitment of leukocytes and macrophages (Ouyang et al. 2003).

Three separate studies demonstrated a significant reduction of neointimal hyperplasia in a rat model of injured arteries after adeno-associated gene transfer of AAV-TIMP-1 compared with untreated arteries in rats without gene transduction (Dollery et al. 1999; Furman et al. 2002; Ramirez Correa et al. 2004).

Similar to the pathophysiology of neointimal hyperplasia, COPD emphysema is a result of thickened bronchiolar wall due to airway remodeling throughout peribronchiolar fibrosis and an increase of airway smooth muscle mass (Siafakas et al. 2007). Kumar et al. identified the SNP rs6609533 of the *TIMP-1* gene as a risk variant for the development of COPD. In this study, COPD patients carrying the SNP rs6609533 showed significantly lower concentrations of TIMP-1 compared to controls (Kumar et al. 2011). In our study, the SNP rs6609533 with allele G was found to be associated with a greater extent of hyperplasia and shunt stenosis. In different transcript isoforms, this polymorphism was annotated to result in changes at the mRNA level in the exon region, as well as in the 3′ UTR and intronic region which may have regulatory impact on gene expression. In our study, immunohistochemistry showed that MMP-9 correlated significantly with the area of neointimal hyperplasia and was an independent predictor for its formation in multivariate analysis. Like EGFR, MMP-9 formed a ring-like structure in the luminal area of the PTFE layer of the shunt. In line with the results of our study, Kumar et al. demonstrated the downregulatory function of rs6609533 in *TIMP-1* in shunt tissue, resulting in enhanced expression of MMPs and thereby facilitating the migration of SMCs and the formation of neointimal hyperplasia.

Children with complex cyanotic heart disease are often treated with platelet inhibition like ASA dosed to efficacy based on thrombocyte-functioning test prior to stage I palliation. In a previous study, we identified clinical factors associated with increased neointimal formation and found a significant inverse correlation between ASA dosage and neointimal hyperplasia, meaning that a lower per kilogram body weight ASA dosage was related to greater neointimal formation (Kottmann et al. 2022). In this study, we see a trend of greater expression of EGFR in shunts removed from patients receiving lower per weight ASS dosage, with no correlation observed on MMP-9 expression. Despite the small sample size limiting statistical significances, this finding might suggest that ASS exhibits its protective effect via reduced EGFR and not MMP-9 expression.

In our study, the immunohistochemical staining for EGFR and MMP-9 showed a significant association to neointimal hyperplasia in multivariate and correlation analysis. SNP evaluation on DNA extracted from peripheral blood of affected patients identified four risk alleles on the genes *EGF* and *TIMP-1* associated with greater neointimal hyperplasia and shunt stenosis. Alterations in the amino acid sequence of the proteins may have a functional impact on protein function and / or protein expression.

In summary, our findings indicate a potential role of EGFR and MMP-9 possibly regulated by EGF and TIMP-1 in the formation of neointimal hyperplasia of SP shunts from children with cyanotic heart disease. Evidence from preclinical studies that targeted these genes to suppress their pathways indicated a reduction of hyperplasia in distinct shunt models. Novel approaches, such as coating PTFE shunts with agents specifically suppressing pathways around EGF/EGFR and TIMP-1/MMP-9, might significantly reduce neointimal hyperplasia in SP shunts and therefore improve the outcome of children with complex and congenital heart defects.

## Limitations

The main limitation of the present study is the restricted sample size given the rarity of the disease studied. Also, the association between protein expression and amount of neointimal proliferation does not conclude causality. Furthermore, the exact influence of the polymorphisms on protein function was not assessed within in the scope of this study.

Methodology of this analysis in a retrospective setting did not allow to specifically examine the effect of hypoxemia on immunohistopathological findings. As reduced oxygen saturation is a strong stimulus for vasculature (Forsythe et al. 1996)), it would have been interesting to investigate the correlation of transcutaneous oxygen saturation and the degree of stenosis, EGFR, and MMP-9 expression. However, documentation of oxygen saturation in medical charts was biased by the fact that many patients received supplemental oxygen and for the majority of cases, it was not possible to evaluate the native oxygen saturation, in the absence of oxygen supplementation. Due to limited shunt material available, only selected stainings could be performed. Other possible signaling pathways were therefore not explored within the scope of this analysis.

## Supplementary Information

Below is the link to the electronic supplementary material.

Supplementary file1 (PDF 2992 KB)

## Data Availability

All data generated or analyzed during this study are included in this published article (and its supplementary information files).

## Declarations

### Accession IDs

Homo sapiens; 9606.

### Accession ID of the core genes studied

EGFR; EGF; MMP-9; TIMP-1.
