# Supplementary material for: EGFR and MMP-9 are associated with neointimal hyperplasia in systemic-to-pulmonary shunts in children with complex cyanotic heart disease
Source: Mamm Genome. 2023 Mar 3;34(2):285–97. doi: 10.1007/s00335-023-09982-3 (PMC10290590; doi:10.1007/s00335-023-09982-3)
Supplement: Supplementary file 1 — Supplementary file1 (PDF 2992 KB) [file 335_2023_9982_MOESM1_ESM.pdf]

Online supplements

Table 1

Antigen	Antibody	Company	Pre-treatment	Seconday Antibody
EGFR	Monoclonal Mouse Anti Human EGFR	DAKO M3563	Proteinase K, DAKO S3004, 0,05 M TRIS/HCl pH 7,6	Zytochem plus HRP/Polymer System (Mouse/Rabbit), POLHRP-100
MMP-9	Monoclonal Mouse Anti-MMP9 (5G3)	abcam ab119906	Target Retrieval Puffer high pH DAKO S3307	Zytochem plus HRP/Polymer System (Mouse/Rabbit), POLHRP-100

***Tbl. 1** Antibodies used in this study, including exact antibody information, companiess, pre-treatments and secondary antibodies*

Multivariate regression

Table 2

Variables Entered/Removed[a]

Model	Variables Entered	Variables Removed	Method
1	MMP-9 [mm^2], ASA dosage [mg/kg/BW], EGFR [mm^2][b]	.	Enter

a. Dependent Variable: Area neointima [mm^2]

b. All requested variables entered.

Table 3

Model Summary

Model	R	R Square	Adjusted R Square	Std. Error of the Estimate
1	0.870[a]	0.756	0.690	0.87411

a. Predictors: (Constant). MMP-9 [mm^2]. ASA dosage [mg/kg/BW]. EGFR [mm^2]

Table 4

ANOVA[a]

Model		Sum of Squares	df	Mean Square	F	Sig.
1	Regression	26.100	3	8.700	11.386	0.001[b]
	Residual	8.405	11	0.764		
	Total	34.505	14			

a. Dependent Variable: Area neointima [mm^2]

b. Predictors: (Constant). MMP-9 [mm^2], ASA dosage [mg/kg/BW], EGFR [mm^2]

Table 5

Coefficients[a]

Model		Unstandardized Coefficients		Standardized Coefficients	t
		B	Std. Error	Beta	
1	(Constant)	-2.265	1.326		-1.709
	ASA dosage [mg/kg/BW]	0.594	0.447	0.220	1.328
	EGFR [mm^2]	7.026	1.868	0.641	3.762
	MMP-9 [mm^2]	7.498	2.261	0.516	3.317

Table 6

Coefficients[a]

Model		Sig.
1	(Constant)	0.116
	ASA dosage [mg/kg/BW]	0.211
	EGFR [mm^2]	0.003
	MMP-9 [mm^2]	0.007

a. Dependent Variable: Area neointima [mm^2]

Table 7

Variation ID	Chr.	Position	Variant	Strand	Symbol	Transcript	Predicted Function	CDNA Position	CDS Position	AA Position	AA Change	Detail	Splice Distance
rs2298989	chr4	110891673	T|C	1	EGF	ENST00000265171	intronic	None	None	None	None	None	1398
rs2298989	chr4	110891673	T|C	1	EGF	ENST00000502579	non-coding intronic	None	None	None	None	None	1398
rs2298989	chr4	110891673	T|C	1	EGF	ENST00000503392	intronic	None	None	None	None	None	1398
rs2298989	chr4	110891673	T|C	1	EGF	ENST00000509793	intronic	None	None	None	None	None	1398
rs2237051	chr4	110901198	G|A	1	EGF	ENST00000511228	5upstream	None	None	None	None	None	None
rs2237051	chr4	110901198	G|A	1	EGF	ENST00000509996	5upstream	None	None	None	None	None	None
rs2237051	chr4	110901198	G|A	1	EGF	ENST00000265171	coding	2569	2124	708	M>I	nonsyn	None
rs2237051	chr4	110901198	G|A	1	EGF	ENST00000503392	coding	2219	2124	708	M>I	nonsyn	None
rs2237051	chr4	110901198	G|A	1	EGF	ENST00000509793	coding	2450	1998	666	M>I	nonsyn	None
rs2298999	chr4	110911907	T|C	1	EGF	ENST00000509996	non-coding intronic	None	None	None	None	None	2496
rs2298999	chr4	110911907	T|C	1	EGF	ENST00000265171	intronic	None	None	None	None	None	2042
rs2298999	chr4	110911907	T|C	1	EGF	ENST00000509793	intronic	None	None	None	None	None	2042
rs2298999	chr4	110911907	T|C	1	EGF	ENST00000503392	intronic	None	None	None	None	None	2042

Tbl. 7 EGF - Predicted annotation of different transcript isoforms [ENSEMBL] *ID = Identification; Chr: = Chromosome; Position = Exact base position on DNA strand; EGF = Epidermal growth factor; CDNA = Complementary deoxyribonucleic acid; CDS = Coding sequence; AA = Amino acid*

Table 8

Variation ID	Chr	Position	Variant	Strand	Symbol	Transcript	Predicted Function	CDNA Position	CDS Position	AA Position	AA Change	Detail	Splice Distance
rs6609533	chrX	47445286	A|G|T	1	TIMP1	ENST00000377018	coding	1219	655	219	T>A|T>S	nonsyn|nonsyn	None
rs6609533	chrX	47445286	A|K	1	TIMP1	ENST00000441738	3downstream	None	None	None	None	None	None
rs6609533	chrX	47445286	A|K	1	TIMP1	ENST00000456754	3utr	959	None	None	None	None	None
rs6609533	chrX	47445286	A|K	1	TIMP1	ENST00000218388	intronic	None	None	None	None	None	220
rs6609533	chrX	47445286	A|K	1	TIMP1	ENST00000377017	intronic	None	None	None	None	None	220
rs6609533	chrX	47445286	A|K	1	TIMP1	ENST00000445623	intronic	None	None	None	None	None	220

Tbl. 8 ***TIMP-1 - Predicted annotation of different transcript isoforms [ENSEMBL]*** *ID = Identification; Chr: = Chromosome; Position = Exact base position on DNA strand; TIMP-1 = Tissue inhibitor of metalloproteinases; CDNA = Complementary deoxyribonucleic acid; CDS = Coding sequence; AA = Amino acid*

Table 9 – PLINK analysis

CHR		SNP	A1	A2	TEST	AFF	UNAFF	CHISQ	DF	p-value
18	dbsnp	rs8094616	G	A	ALLELIC	17/5	14/36	15.13	1	0.0001004
23	dbsnp	rs5966792	A	G	ALLELIC	17/5	14/36	15.13	1	0.0001004
7	dbsnp	rs933423	A	G	ALLELIC	8/12	2/48	15.12	1	0.0001009
17	dbsnp	rs8075084	A	G	ALLELIC	8/12	2/48	15.12	1	0.0001009
3	dbsnp	rs10935496	A	G	ALLELIC	6/16	0/50	14.88	1	0.0001148
8	dbsnp	rs264813	C	A	ALLELIC	6/16	0/50	14.88	1	0.0001148
10	dbsnp	rs266092	A	T	ALLELIC	6/16	0/50	14.88	1	0.0001148
16	dbsnp	rs7187569	A	G	ALLELIC	6/16	0/50	14.88	1	0.0001148
17	dbsnp	rs9900865	A	G	ALLELIC	6/16	0/50	14.88	1	0.0001148
23	dbsnp	rs180488	G	A	ALLELIC	6/16	0/50	14.88	1	0.0001148
23	dbsnp	rs180490	A	G	ALLELIC	6/16	0/50	14.88	1	0.0001148
1	dbsnp	rs11120922	A	G	ALLELIC	12/10	6/44	14.75	1	0.0001228
2	dbsnp	rs7602912	A	G	ALLELIC	12/10	6/44	14.75	1	0.0001228
2	dbsnp	rs10933143	A	G	ALLELIC	12/10	6/44	14.75	1	0.0001228
5	dbsnp	rs10059632	G	A	ALLELIC	12/10	6/44	14.75	1	0.0001228
22	dbsnp	rs5770650	A	C	ALLELIC	12/10	6/44	14.75	1	0.0001228
20	dbsnp	rs11906748	A	G	ALLELIC	1/21	26/24	14.68	1	0.0001274
2	dbsnp	rs6747078	G	A	ALLELIC	13/7	9/41	14.64	1	0.0001299
4	dbsnp	rs4861106	A	G	ALLELIC	13/7	9/41	14.64	1	0.0001299
5	dbsnp	rs6858929	A	G	ALLELIC	14/8	9/41	14.64	1	0.0001304
10	dbsnp	rs2458698	G	A	ALLELIC	14/8	9/41	14.64	1	0.0001304
15	dbsnp	rs11072463	C	A	ALLELIC	14/8	9/41	14.64	1	0.0001304
11	dbsnp	rs17127950	G	A	ALLELIC	6/10	0/34	14.49	1	0.000141
20	dbsnp	rs6065411	C	A	ALLELIC	11/7	6/38	14.47	1	0.0001426
6	dbsnp	rs6911180	A	G	ALLELIC	2/18	30/20	14.39	1	0.0001485
6	dbsnp	rs3757030	G	A	ALLELIC	2/18	30/20	14.39	1	0.0001485
14	dbsnp	rs17758181	G	A	ALLELIC	11/9	6/44	14.37	1	0.0001506
22	dbsnp	rs134099	G	A	ALLELIC	11/9	6/44	14.37	1	0.0001506
9	dbsnp	rs2779562	A	G	ALLELIC	3/19	31/19	14.34	1	0.0001527
9	dbsnp	rs2779572	A	C	ALLELIC	3/19	31/19	14.34	1	0.0001527
4	dbsnp	rs11731909	C	A	ALLELIC	6/16	0/48	14.32	1	0.0001544
2	dbsnp	rs6435692	G	A	ALLELIC	1/19	27/23	14.29	1	0.0001566
10	dbsnp	rs10903784	A	C	ALLELIC	15/5	13/37	14.29	1	0.0001566
15	dbsnp	rs7168561	G	A	ALLELIC	15/5	13/37	14.29	1	0.0001566
20	dbsnp	rs6025157	A	G	ALLELIC	17/5	14/34	14.15	1	0.0001689
1	dbsnp	rs860107	G	A	ALLELIC	11/11	5/45	14.14	1	0.0001694
9	dbsnp	rs2472459	A	G	ALLELIC	11/11	5/45	14.14	1	0.0001694
11	dbsnp	rs2631511	A	G	ALLELIC	11/11	5/45	14.14	1	0.0001694
12	dbsnp	rs7968680	G	A	ALLELIC	11/11	5/45	14.14	1	0.0001694
12	dbsnp	rs1470321	A	G	ALLELIC	11/11	5/45	14.14	1	0.0001694
14	dbsnp	rs7153751	G	A	ALLELIC	11/11	5/45	14.14	1	0.0001694
23	dbsnp	rs6609542	G	A	ALLELIC	15/7	11/39	14.12	1	0.0001712
23	dbsnp	rs4824629	G	A	ALLELIC	15/7	11/39	14.12	1	0.0001712
23	dbsnp	rs5926852	G	A	ALLELIC	4/18	33/17	13.98	1	0.0001843
8	dbsnp	rs6998952	A	G	ALLELIC	16/6	13/37	13.87	1	0.0001962
8	dbsnp	rs13272740	A	G	ALLELIC	16/6	13/37	13.87	1	0.0001962

2	dbsnp	rs2888051	A	G	ALLELIC	2/20	28/22	13.83	1	0.0002
15	dbsnp	rs12911648	A	C	ALLELIC	2/20	28/22	13.83	1	0.0002
23	dbsnp	rs6654815	C	A	ALLELIC	2/20	28/22	13.83	1	0.0002
1	dbsnp	rs833976	A	C	ALLELIC	17/5	15/35	13.83	1	0.0002004
23	dbsnp	rs2283735	G	A	ALLELIC	15/7	10/36	13.81	1	0.0002025
1	dbsnp	rs6677745	A	C	ALLELIC	7/15	1/49	13.75	1	0.0002084
2	dbsnp	rs2129495	A	C	ALLELIC	7/15	1/49	13.75	1	0.0002084
2	dbsnp	rs7591531	A	G	ALLELIC	7/15	1/49	13.75	1	0.0002084
7	dbsnp	rs3108406	A	G	ALLELIC	7/15	1/49	13.75	1	0.0002084
7	dbsnp	rs7778019	A	G	ALLELIC	7/15	1/49	13.75	1	0.0002084
8	dbsnp	rs264809	C	A	ALLELIC	7/15	1/49	13.75	1	0.0002084
9	dbsnp	rs4237192	G	A	ALLELIC	7/15	1/49	13.75	1	0.0002084
10	dbsnp	rs3006509	G	A	ALLELIC	7/15	1/49	13.75	1	0.0002084
15	dbsnp	rs2220806	C	A	ALLELIC	7/15	1/49	13.75	1	0.0002084
18	dbsnp	rs7350995	G	A	ALLELIC	7/15	1/49	13.75	1	0.0002084
23	dbsnp	rs1619562	C	A	ALLELIC	7/15	1/49	13.75	1	0.0002084
23	dbsnp	rs722220	A	G	ALLELIC	7/15	1/49	13.75	1	0.0002084
23	dbsnp	rs5906161	A	G	ALLELIC	7/15	1/49	13.75	1	0.0002084
23	dbsnp	rs5951352	C	A	ALLELIC	7/15	1/49	13.75	1	0.0002084
1	dbsnp	rs12026592	G	A	ALLELIC	13/9	8/42	13.73	1	0.0002109
1	dbsnp	rs12041138	A	G	ALLELIC	13/9	8/42	13.73	1	0.0002109
1	dbsnp	rs1592251	A	C	ALLELIC	13/9	8/42	13.73	1	0.0002109
5	dbsnp	rs4272164	C	A	ALLELIC	13/9	8/42	13.73	1	0.0002109
14	dbsnp	rs7155151	C	A	ALLELIC	13/9	8/42	13.73	1	0.0002109
16	dbsnp	rs6500514	G	A	ALLELIC	13/9	8/42	13.73	1	0.0002109
3	dbsnp	rs1353322	A	G	ALLELIC	17/3	18/32	13.72	1	0.0002122
3	dbsnp	rs778521	A	G	ALLELIC	17/3	18/32	13.72	1	0.0002122
1	dbsnp	rs4911985	G	A	ALLELIC	10/12	4/46	13.68	1	0.0002164
1	dbsnp	rs12741053	G	A	ALLELIC	10/12	4/46	13.68	1	0.0002164
1	dbsnp	rs12742348	A	G	ALLELIC	10/12	4/46	13.68	1	0.0002164
1	dbsnp	rs12030570	A	G	ALLELIC	10/12	4/46	13.68	1	0.0002164
12	dbsnp	rs17813210	C	A	ALLELIC	10/12	4/46	13.68	1	0.0002164
12	dbsnp	rs2403151	G	A	ALLELIC	10/12	4/46	13.68	1	0.0002164
13	dbsnp	rs11618329	G	A	ALLELIC	10/12	4/46	13.68	1	0.0002164
15	dbsnp	rs7174702	G	A	ALLELIC	10/12	4/46	13.68	1	0.0002164
23	dbsnp	rs16987956	A	G	ALLELIC	10/12	4/46	13.68	1	0.0002164
23	dbsnp	rs17002540	A	G	ALLELIC	10/12	4/46	13.68	1	0.0002164
18	dbsnp	rs2957139	G	A	ALLELIC	10/10	5/45	13.58	1	0.0002291
18	dbsnp	rs2957152	G	A	ALLELIC	10/10	5/45	13.58	1	0.0002291
2	dbsnp	rs7589542	A	G	ALLELIC	12/8	8/42	13.55	1	0.000232
8	dbsnp	rs6997004	G	A	ALLELIC	12/8	8/42	13.55	1	0.000232
9	dbsnp	rs7044062	C	A	ALLELIC	12/8	8/42	13.55	1	0.000232
1	dbsnp	rs4658204	A	G	ALLELIC	5/15	0/50	13.46	1	0.0002435
6	dbsnp	rs1567266	A	G	ALLELIC	5/15	0/50	13.46	1	0.0002435
18	dbsnp	rs3169983	G	A	ALLELIC	5/15	0/50	13.46	1	0.0002435
1	dbsnp	rs763173	A	C	ALLELIC	9/13	3/47	13.41	1	0.0002509
1	dbsnp	rs4908625	A	G	ALLELIC	9/13	3/47	13.41	1	0.0002509
2	dbsnp	rs7575939	A	G	ALLELIC	9/13	3/47	13.41	1	0.0002509

5	dbsnp	rs12659043	A	G	ALLELIC	9/13	3/47	13.41	1	0.0002509
5	dbsnp	rs17156302	A	G	ALLELIC	9/13	3/47	13.41	1	0.0002509
5	dbsnp	rs2913810	G	A	ALLELIC	9/13	3/47	13.41	1	0.0002509
6	dbsnp	rs12191018	A	C	ALLELIC	9/13	3/47	13.41	1	0.0002509
7	dbsnp	rs10228406	A	G	ALLELIC	9/13	3/47	13.41	1	0.0002509
7	dbsnp	rs7801041	G	A	ALLELIC	9/13	3/47	13.41	1	0.0002509
13	dbsnp	rs9568485	A	C	ALLELIC	9/13	3/47	13.41	1	0.0002509
14	dbsnp	rs8012926	C	A	ALLELIC	9/13	3/47	13.41	1	0.0002509
23	dbsnp	rs12847086	G	A	ALLELIC	9/13	3/47	13.41	1	0.0002509
23	dbsnp	rs5909981	G	A	ALLELIC	9/13	3/47	13.41	1	0.0002509
23	dbsnp	rs16997304	G	A	ALLELIC	9/13	3/47	13.41	1	0.0002509
23	dbsnp	rs5975040	G	A	ALLELIC	9/13	3/47	13.41	1	0.0002509
23	dbsnp	rs17313456	A	G	ALLELIC	9/13	3/47	13.41	1	0.0002509
1	dbsnp	rs4908626	A	C	ALLELIC	8/14	2/48	13.38	1	0.0002544
1	dbsnp	rs11579620	A	G	ALLELIC	8/14	2/48	13.38	1	0.0002544
1	dbsnp	rs12405458	G	A	ALLELIC	8/14	2/48	13.38	1	0.0002544
4	dbsnp	rs6856663	A	G	ALLELIC	8/14	2/48	13.38	1	0.0002544
5	dbsnp	rs4865540	A	C	ALLELIC	8/14	2/48	13.38	1	0.0002544
8	dbsnp	rs1552286	G	A	ALLELIC	8/14	2/48	13.38	1	0.0002544
9	dbsnp	rs10975121	G	A	ALLELIC	8/14	2/48	13.38	1	0.0002544
9	dbsnp	rs10968572	A	G	ALLELIC	8/14	2/48	13.38	1	0.0002544
9	dbsnp	rs10491888	G	A	ALLELIC	8/14	2/48	13.38	1	0.0002544
11	dbsnp	rs12797204	A	G	ALLELIC	8/14	2/48	13.38	1	0.0002544
16	dbsnp	rs8054996	A	G	ALLELIC	8/14	2/48	13.38	1	0.0002544
19	dbsnp	rs10408156	G	A	ALLELIC	8/14	2/48	13.38	1	0.0002544
23	dbsnp	rs5979643	A	G	ALLELIC	8/14	2/48	13.38	1	0.0002544
23	dbsnp	rs1921696	G	A	ALLELIC	8/14	2/48	13.38	1	0.0002544
23	dbsnp	rs17331524	A	G	ALLELIC	8/14	2/48	13.38	1	0.0002544
4	dbsnp	rs933830	C	A	ALLELIC	11/7	8/42	13.38	1	0.0002547
12	dbsnp	rs11057065	G	A	ALLELIC	16/4	16/34	13.26	1	0.0002707
12	dbsnp	rs2072449	A	G	ALLELIC	16/4	16/34	13.26	1	0.0002707
6	dbsnp	rs6926382	A	C	ALLELIC	3/19	30/20	13.23	1	0.0002758
9	dbsnp	rs7023422	G	A	ALLELIC	13/7	10/40	13.11	1	0.0002933
11	dbsnp	rs925151	A	G	ALLELIC	13/7	10/40	13.11	1	0.0002933
20	dbsnp	rs571847	A	G	ALLELIC	13/7	10/40	13.11	1	0.0002933
23	dbsnp	rs4898	G	A	ALLELIC	13/7	10/40	13.11	1	0.0002933
23	dbsnp	rs6609533	G	A	ALLELIC	13/7	10/40	13.11	1	0.0002933
23	dbsnp	rs2070584	C	A	ALLELIC	13/7	10/40	13.11	1	0.0002933
1	dbsnp	rs11120900	G	A	ALLELIC	14/8	10/40	13.09	1	0.0002967
2	dbsnp	rs2111485	A	G	ALLELIC	14/8	10/40	13.09	1	0.0002967
5	dbsnp	rs11167499	G	A	ALLELIC	14/8	10/40	13.09	1	0.0002967
10	dbsnp	rs1010719	A	G	ALLELIC	14/8	10/40	13.09	1	0.0002967
12	dbsnp	rs11045018	A	G	ALLELIC	14/8	10/40	13.09	1	0.0002967
23	dbsnp	rs5905615	G	A	ALLELIC	14/8	10/40	13.09	1	0.0002967
23	dbsnp	rs9320072	A	G	ALLELIC	14/8	10/40	13.09	1	0.0002967
23	dbsnp	rs12389634	C	A	ALLELIC	14/8	10/40	13.09	1	0.0002967
23	dbsnp	rs1209098	A	G	ALLELIC	14/8	10/40	13.09	1	0.0002967
14	dbsnp	rs1200412	C	A	ALLELIC	0/22	21/29	13.04	1	0.0003041

23	dbsnp	rs17338737	A	G	ALLELIC	0/22	21/29	13.04	1	0.0003041
2	dbsnp	rs1515114	A	G	ALLELIC	15/5	14/36	13.01	1	0.0003107
9	dbsnp	rs4372077	C	A	ALLELIC	10/12	4/44	12.99	1	0.0003128
12	dbsnp	rs10506807	G	A	ALLELIC	9/11	4/46	12.93	1	0.0003229
12	dbsnp	rs12302671	A	G	ALLELIC	9/11	4/46	12.93	1	0.0003229
12	dbsnp	rs7135927	G	A	ALLELIC	9/11	4/46	12.93	1	0.0003229
14	dbsnp	rs178497	A	C	ALLELIC	9/11	4/46	12.93	1	0.0003229
18	dbsnp	rs12959488	A	G	ALLELIC	9/11	4/46	12.93	1	0.0003229
2	dbsnp	rs280199	A	G	ALLELIC	12/10	7/43	12.93	1	0.0003235
2	dbsnp	rs7588213	C	A	ALLELIC	12/10	7/43	12.93	1	0.0003235
2	dbsnp	rs280194	G	A	ALLELIC	12/10	7/43	12.93	1	0.0003235
2	dbsnp	rs2249979	A	G	ALLELIC	12/10	7/43	12.93	1	0.0003235
2	dbsnp	rs984971	G	A	ALLELIC	12/10	7/43	12.93	1	0.0003235
10	dbsnp	rs2351347	G	A	ALLELIC	12/10	7/43	12.93	1	0.0003235
15	dbsnp	rs4779779	A	C	ALLELIC	12/10	7/43	12.93	1	0.0003235
18	dbsnp	rs12968085	A	G	ALLELIC	12/10	7/43	12.93	1	0.0003235
23	dbsnp	rs5943636	A	G	ALLELIC	12/10	7/43	12.93	1	0.0003235
7	dbsnp	rs702479	G	A	ALLELIC	11/7	5/31	12.83	1	0.0003404
13	dbsnp	rs7324573	A	G	ALLELIC	0/20	22/28	12.83	1	0.0003405
5	dbsnp	rs2251105	A	G	ALLELIC	4/18	32/18	12.83	1	0.0003413
6	dbsnp	rs2211074	A	G	ALLELIC	4/18	32/18	12.83	1	0.0003413
9	dbsnp	rs1182654	A	G	ALLELIC	18/4	18/32	12.83	1	0.0003413
2	dbsnp	rs7562693	G	A	ALLELIC	2/20	27/23	12.81	1	0.0003449
9	dbsnp	rs2065378	C	A	ALLELIC	2/20	27/23	12.81	1	0.0003449
23	dbsnp	rs5931086	A	G	ALLELIC	2/20	27/23	12.81	1	0.0003449
2	dbsnp	rs7426206	G	A	ALLELIC	1/21	24/26	12.73	1	0.0003602
11	dbsnp	rs7947488	A	G	ALLELIC	1/21	24/26	12.73	1	0.0003602
1	dbsnp	rs2841977	G	A	ALLELIC	15/7	12/38	12.72	1	0.0003609
3	dbsnp	rs6550734	C	A	ALLELIC	15/7	12/38	12.72	1	0.0003609
4	dbsnp	rs6840470	G	A	ALLELIC	15/7	12/38	12.72	1	0.0003609
10	dbsnp	rs2388097	G	A	ALLELIC	15/7	12/38	12.72	1	0.0003609
23	dbsnp	rs4829067	G	A	ALLELIC	15/7	12/38	12.72	1	0.0003609
23	dbsnp	rs2765815	A	G	ALLELIC	15/7	12/38	12.72	1	0.0003609
23	dbsnp	rs1998837	G	A	ALLELIC	15/7	12/38	12.72	1	0.0003609
23	dbsnp	rs2742917	G	A	ALLELIC	15/7	12/38	12.72	1	0.0003609
8	dbsnp	rs7003874	A	G	ALLELIC	17/5	16/34	12.61	1	0.0003831
13	dbsnp	rs1322960	G	A	ALLELIC	17/5	16/34	12.61	1	0.0003831
13	dbsnp	rs2806947	G	A	ALLELIC	17/5	16/34	12.61	1	0.0003831
20	dbsnp	rs6091845	A	G	ALLELIC	17/5	16/34	12.61	1	0.0003831
23	dbsnp	rs5909374	A	G	ALLELIC	17/5	16/34	12.61	1	0.0003831
23	dbsnp	rs707287	G	A	ALLELIC	17/5	16/34	12.61	1	0.0003831
23	dbsnp	rs5966620	A	G	ALLELIC	17/5	16/34	12.61	1	0.0003831
12	dbsnp	rs10850830	A	G	ALLELIC	16/6	14/36	12.57	1	0.000391
12	dbsnp	rs10850834	G	A	ALLELIC	16/6	14/36	12.57	1	0.000391
1	dbsnp	rs2246315	A	G	ALLELIC	11/9	7/43	12.57	1	0.0003917
8	dbsnp	rs2244715	A	G	ALLELIC	11/9	7/43	12.57	1	0.0003917
10	dbsnp	rs3006399	A	C	ALLELIC	11/9	7/43	12.57	1	0.0003917
20	dbsnp	rs471085	C	A	ALLELIC	8/12	3/47	12.47	1	0.0004139

7	dbsnp	rs17735470	C	A	ALLELIC	6/14	1/49	12.44	1	0.0004192
10	dbsnp	rs644556	G	A	ALLELIC	6/14	1/49	12.44	1	0.0004192
22	dbsnp	rs5767152	A	G	ALLELIC	6/14	1/49	12.44	1	0.0004192
2	dbsnp	rs1357142	A	G	ALLELIC	1/19	25/25	12.39	1	0.0004315
18	dbsnp	rs8097554	G	A	ALLELIC	1/19	25/25	12.39	1	0.0004315
2	dbsnp	rs6715314	G	A	ALLELIC	2/18	28/22	12.34	1	0.0004426
6	dbsnp	rs7745188	C	A	ALLELIC	13/7	10/38	12.3	1	0.0004521
23	dbsnp	rs12852016	G	A	ALLELIC	14/8	10/38	12.27	1	0.000461
4	dbsnp	rs1525760	G	A	ALLELIC	7/13	2/48	12.25	1	0.0004645
4	dbsnp	rs1588041	A	G	ALLELIC	7/13	2/48	12.25	1	0.0004645
12	dbsnp	rs17022417	G	A	ALLELIC	7/13	2/48	12.25	1	0.0004645
12	dbsnp	rs7132880	G	A	ALLELIC	7/13	2/48	12.25	1	0.0004645
16	dbsnp	rs9302767	C	A	ALLELIC	7/13	2/48	12.25	1	0.0004645
19	dbsnp	rs9917029	G	A	ALLELIC	7/13	2/48	12.25	1	0.0004645
23	dbsnp	rs5953452	G	A	ALLELIC	7/13	2/48	12.25	1	0.0004645
2	dbsnp	rs10933140	G	A	ALLELIC	11/11	6/44	12.23	1	0.0004699
5	dbsnp	rs2973820	A	G	ALLELIC	11/11	6/44	12.23	1	0.0004699
5	dbsnp	rs7715312	A	G	ALLELIC	11/11	6/44	12.23	1	0.0004699
8	dbsnp	rs1456306	A	G	ALLELIC	11/11	6/44	12.23	1	0.0004699
11	dbsnp	rs10741878	G	A	ALLELIC	11/11	6/44	12.23	1	0.0004699
14	dbsnp	rs12717214	A	G	ALLELIC	11/11	6/44	12.23	1	0.0004699
14	dbsnp	rs1884810	G	A	ALLELIC	11/11	6/44	12.23	1	0.0004699
15	dbsnp	rs12903635	A	G	ALLELIC	11/11	6/44	12.23	1	0.0004699
18	dbsnp	rs12456915	G	A	ALLELIC	11/11	6/44	12.23	1	0.0004699
20	dbsnp	rs6077792	A	C	ALLELIC	11/11	6/44	12.23	1	0.0004699
20	dbsnp	rs1591168	A	G	ALLELIC	11/11	6/44	12.23	1	0.0004699
23	dbsnp	rs6610563	A	G	ALLELIC	11/11	6/44	12.23	1	0.0004699
23	dbsnp	rs6520278	A	G	ALLELIC	11/11	6/44	12.23	1	0.0004699
23	dbsnp	rs6418213	A	C	ALLELIC	11/11	6/44	12.23	1	0.0004699
23	dbsnp	rs6616285	A	C	ALLELIC	11/11	6/44	12.23	1	0.0004699
23	dbsnp	rs5945042	A	G	ALLELIC	11/11	6/44	12.23	1	0.0004699
23	dbsnp	rs5931774	G	A	ALLELIC	11/11	6/44	12.23	1	0.0004699
1	dbsnp	rs17131417	A	G	ALLELIC	5/17	0/50	12.21	1	0.0004749
2	dbsnp	rs4671716	G	A	ALLELIC	5/17	0/50	12.21	1	0.0004749
2	dbsnp	rs11687583	A	G	ALLELIC	5/17	0/50	12.21	1	0.0004749
2	dbsnp	rs4414697	A	G	ALLELIC	5/17	0/50	12.21	1	0.0004749
5	dbsnp	rs13163497	A	G	ALLELIC	5/17	0/50	12.21	1	0.0004749
6	dbsnp	rs770939	A	G	ALLELIC	5/17	0/50	12.21	1	0.0004749
6	dbsnp	rs16877559	A	G	ALLELIC	5/17	0/50	12.21	1	0.0004749
6	dbsnp	rs1935606	C	A	ALLELIC	5/17	0/50	12.21	1	0.0004749
6	dbsnp	rs1342059	A	G	ALLELIC	5/17	0/50	12.21	1	0.0004749
7	dbsnp	rs1464807	A	C	ALLELIC	5/17	0/50	12.21	1	0.0004749
7	dbsnp	rs7801900	A	G	ALLELIC	5/17	0/50	12.21	1	0.0004749
7	dbsnp	rs1593306	C	A	ALLELIC	5/17	0/50	12.21	1	0.0004749
8	dbsnp	rs3096605	A	G	ALLELIC	5/17	0/50	12.21	1	0.0004749
9	dbsnp	rs10120911	A	G	ALLELIC	5/17	0/50	12.21	1	0.0004749
10	dbsnp	rs1041158	A	G	ALLELIC	5/17	0/50	12.21	1	0.0004749
10	dbsnp	rs7904589	A	G	ALLELIC	5/17	0/50	12.21	1	0.0004749

12	dbsnp	rs1903934	G	A	ALLELIC	5/17	0/50	12.21	1	0.0004749
12	dbsnp	rs1025090	C	A	ALLELIC	5/17	0/50	12.21	1	0.0004749
12	dbsnp	rs10880758	G	A	ALLELIC	5/17	0/50	12.21	1	0.0004749
12	dbsnp	rs17302436	G	A	ALLELIC	5/17	0/50	12.21	1	0.0004749
12	dbsnp	rs17022408	G	A	ALLELIC	5/17	0/50	12.21	1	0.0004749
12	dbsnp	rs1607807	C	A	ALLELIC	5/17	0/50	12.21	1	0.0004749
12	dbsnp	rs12427269	A	G	ALLELIC	5/17	0/50	12.21	1	0.0004749
12	dbsnp	rs12317950	G	A	ALLELIC	5/17	0/50	12.21	1	0.0004749
16	dbsnp	rs12446573	G	A	ALLELIC	5/17	0/50	12.21	1	0.0004749
20	dbsnp	rs6084145	G	A	ALLELIC	5/17	0/50	12.21	1	0.0004749
20	dbsnp	rs292117	C	A	ALLELIC	5/17	0/50	12.21	1	0.0004749
20	dbsnp	rs10485791	G	A	ALLELIC	5/17	0/50	12.21	1	0.0004749
23	dbsnp	rs7892146	A	G	ALLELIC	5/17	0/50	12.21	1	0.0004749
23	dbsnp	rs7887668	C	A	ALLELIC	5/17	0/50	12.21	1	0.0004749
23	dbsnp	rs5990844	G	A	ALLELIC	5/17	0/50	12.21	1	0.0004749
23	dbsnp	rs12009317	A	G	ALLELIC	5/17	0/50	12.21	1	0.0004749
23	dbsnp	rs2071583	C	A	ALLELIC	5/17	0/50	12.21	1	0.0004749
23	dbsnp	rs5951729	C	A	ALLELIC	5/17	0/50	12.21	1	0.0004749
23	dbsnp	rs10284225	G	A	ALLELIC	5/17	0/50	12.21	1	0.0004749
23	dbsnp	rs5974525	A	G	ALLELIC	5/17	0/50	12.21	1	0.0004749
23	dbsnp	rs2719849	A	G	ALLELIC	5/17	0/50	12.21	1	0.0004749
23	dbsnp	rs7052815	G	A	ALLELIC	5/17	0/50	12.21	1	0.0004749
23	dbsnp	rs5980360	G	A	ALLELIC	5/17	0/50	12.21	1	0.0004749
1	dbsnp	rs2290501	C	A	ALLELIC	0/22	20/30	12.18	1	0.0004819
1	dbsnp	rs10802944	G	A	ALLELIC	0/22	20/30	12.18	1	0.0004819
2	dbsnp	rs10084179	A	G	ALLELIC	0/22	20/30	12.18	1	0.0004819
14	dbsnp	rs12892702	A	C	ALLELIC	12/10	7/41	12.18	1	0.0004824
8	dbsnp	rs1879571	A	G	ALLELIC	3/19	29/21	12.18	1	0.0004836
8	dbsnp	rs2272761	G	A	ALLELIC	3/19	29/21	12.18	1	0.0004836
17	dbsnp	rs8072684	C	A	ALLELIC	3/19	29/21	12.18	1	0.0004836
23	dbsnp	rs5924847	C	A	ALLELIC	3/19	29/21	12.18	1	0.0004836
23	dbsnp	rs2734239	A	G	ALLELIC	3/19	29/21	12.18	1	0.0004836
1	dbsnp	rs12082618	G	A	ALLELIC	13/9	9/41	12.16	1	0.0004891
1	dbsnp	rs12744184	G	A	ALLELIC	13/9	9/41	12.16	1	0.0004891
1	dbsnp	rs718609	A	G	ALLELIC	13/9	9/41	12.16	1	0.0004891
4	dbsnp	rs2522482	A	G	ALLELIC	13/9	9/41	12.16	1	0.0004891
5	dbsnp	rs2914928	G	A	ALLELIC	13/9	9/41	12.16	1	0.0004891
5	dbsnp	rs3776081	G	A	ALLELIC	13/9	9/41	12.16	1	0.0004891
7	dbsnp	rs6952398	G	A	ALLELIC	13/9	9/41	12.16	1	0.0004891
9	dbsnp	rs2515618	A	G	ALLELIC	13/9	9/41	12.16	1	0.0004891
10	dbsnp	rs12412805	A	G	ALLELIC	13/9	9/41	12.16	1	0.0004891
10	dbsnp	rs10787373	A	G	ALLELIC	13/9	9/41	12.16	1	0.0004891
15	dbsnp	rs11637620	A	G	ALLELIC	13/9	9/41	12.16	1	0.0004891
15	dbsnp	rs4347602	A	C	ALLELIC	13/9	9/41	12.16	1	0.0004891
21	dbsnp	rs2837341	A	G	ALLELIC	13/9	9/41	12.16	1	0.0004891
22	dbsnp	rs1421312	G	A	ALLELIC	13/9	9/41	12.16	1	0.0004891
23	dbsnp	rs170243	A	G	ALLELIC	13/9	9/41	12.16	1	0.0004891
23	dbsnp	rs12559028	A	C	ALLELIC	13/9	9/41	12.16	1	0.0004891

6	dbsnp	rs3901172	C	A	ALLELIC	7/11	2/42	12.14	1	0.000493
4	dbsnp	rs11132270	C	A	ALLELIC	16/4	17/33	12.13	1	0.0004959
11	dbsnp	rs4340049	G	A	ALLELIC	4/16	33/17	12.13	1	0.0004959
23	dbsnp	rs4827950	A	G	ALLELIC	15/5	14/34	12.12	1	0.0004975
1	dbsnp	rs10917052	A	G	ALLELIC	0/20	21/29	12	1	0.000532
8	dbsnp	rs7825742	G	A	ALLELIC	12/8	9/41	12	1	0.000532
10	dbsnp	rs6583692	C	A	ALLELIC	12/8	9/41	12	1	0.000532
11	dbsnp	rs10742464	A	G	ALLELIC	12/8	9/41	12	1	0.000532
12	dbsnp	rs3782683	C	A	ALLELIC	12/8	9/41	12	1	0.000532
12	dbsnp	rs2277414	A	C	ALLELIC	12/8	9/41	12	1	0.000532
18	dbsnp	rs11081202	C	A	ALLELIC	12/8	9/41	12	1	0.000532
18	dbsnp	rs4548980	G	A	ALLELIC	12/8	9/41	12	1	0.000532
1	dbsnp	rs12743229	G	A	ALLELIC	6/14	1/47	11.91	1	0.000557
2	dbsnp	rs1972947	G	A	ALLELIC	15/7	12/36	11.87	1	0.0005698
16	dbsnp	rs7198064	G	A	ALLELIC	2/20	26/24	11.84	1	0.0005809
19	dbsnp	rs4807101	A	G	ALLELIC	2/20	26/24	11.84	1	0.0005809
20	dbsnp	rs6040446	G	A	ALLELIC	2/20	26/24	11.84	1	0.0005809
20	dbsnp	rs714691	G	A	ALLELIC	2/20	26/24	11.84	1	0.0005809
17	dbsnp	rs4968932	A	G	ALLELIC	1/21	23/27	11.81	1	0.0005877
20	dbsnp	rs6028900	A	C	ALLELIC	1/21	23/27	11.81	1	0.0005877
12	dbsnp	rs11171747	C	A	ALLELIC	15/5	15/35	11.81	1	0.0005883
12	dbsnp	rs7960225	A	G	ALLELIC	15/5	15/35	11.81	1	0.0005883
12	dbsnp	rs1545686	A	G	ALLELIC	15/5	15/35	11.81	1	0.0005883
12	dbsnp	rs7967594	A	C	ALLELIC	15/5	15/35	11.81	1	0.0005883
5	dbsnp	rs173969	A	G	ALLELIC	2/20	25/23	11.77	1	0.0006025
2	dbsnp	rs6757981	A	G	ALLELIC	5/17	0/48	11.75	1	0.000609
12	dbsnp	rs10880755	A	C	ALLELIC	5/17	0/48	11.75	1	0.000609
1	dbsnp	rs2419370	A	G	ALLELIC	18/4	19/31	11.74	1	0.0006109
2	dbsnp	rs721087	A	G	ALLELIC	18/4	19/31	11.74	1	0.0006109
10	dbsnp	rs7070915	A	G	ALLELIC	4/18	31/19	11.74	1	0.0006109
14	dbsnp	rs10148947	G	A	ALLELIC	4/18	31/19	11.74	1	0.0006109
16	dbsnp	rs2352933	C	A	ALLELIC	4/18	31/19	11.74	1	0.0006109
19	dbsnp	rs2278498	A	G	ALLELIC	18/4	19/31	11.74	1	0.0006109
21	dbsnp	rs2822919	A	C	ALLELIC	4/18	31/19	11.74	1	0.0006109
23	dbsnp	rs5926878	C	A	ALLELIC	4/18	31/19	11.74	1	0.0006109
23	dbsnp	rs1551201	G	A	ALLELIC	4/18	31/19	11.74	1	0.0006109
23	dbsnp	rs5926891	A	G	ALLELIC	4/18	31/19	11.74	1	0.0006109
1	dbsnp	rs10912773	A	G	ALLELIC	13/7	11/39	11.72	1	0.0006171
10	dbsnp	rs12413892	G	A	ALLELIC	13/7	11/39	11.72	1	0.0006171
8	dbsnp	rs387706	A	C	ALLELIC	10/10	6/44	11.7	1	0.0006253
8	dbsnp	rs3779951	A	G	ALLELIC	10/10	6/44	11.7	1	0.0006253
10	dbsnp	rs12775504	C	A	ALLELIC	10/10	6/44	11.7	1	0.0006253
10	dbsnp	rs10786770	G	A	ALLELIC	10/10	6/44	11.7	1	0.0006253
12	dbsnp	rs2728553	A	C	ALLELIC	10/10	6/44	11.7	1	0.0006253
14	dbsnp	rs178488	G	A	ALLELIC	10/10	6/44	11.7	1	0.0006253
18	dbsnp	rs3017366	G	A	ALLELIC	10/10	6/44	11.7	1	0.0006253
18	dbsnp	rs3017368	G	A	ALLELIC	10/10	6/44	11.7	1	0.0006253
23	dbsnp	rs844971	A	G	ALLELIC	10/10	6/44	11.7	1	0.0006253

1	dbsnp	rs6424228	A	C	ALLELIC	14/8	11/39	11.69	1	0.00063
2	dbsnp	rs7587978	A	G	ALLELIC	14/8	11/39	11.69	1	0.00063
2	dbsnp	rs9288518	G	A	ALLELIC	14/8	11/39	11.69	1	0.00063
5	dbsnp	rs6890771	A	G	ALLELIC	14/8	11/39	11.69	1	0.00063
9	dbsnp	rs7035592	G	A	ALLELIC	14/8	11/39	11.69	1	0.00063
9	dbsnp	rs2472509	C	A	ALLELIC	14/8	11/39	11.69	1	0.00063
10	dbsnp	rs871988	C	A	ALLELIC	14/8	11/39	11.69	1	0.00063
12	dbsnp	rs10881068	G	A	ALLELIC	14/8	11/39	11.69	1	0.00063
13	dbsnp	rs11843134	A	C	ALLELIC	14/8	11/39	11.69	1	0.00063
18	dbsnp	rs1557399	A	G	ALLELIC	14/8	11/39	11.69	1	0.00063
23	dbsnp	rs5963712	A	C	ALLELIC	14/8	11/39	11.69	1	0.00063
23	dbsnp	rs2294219	G	A	ALLELIC	14/8	11/39	11.69	1	0.00063
1	dbsnp	rs6670617	A	C	ALLELIC	14/6	13/37	11.67	1	0.0006342
1	dbsnp	rs4650989	A	G	ALLELIC	14/6	13/37	11.67	1	0.0006342
1	dbsnp	rs7555067	A	G	ALLELIC	14/6	13/37	11.67	1	0.0006342
1	dbsnp	rs17301013	A	G	ALLELIC	14/6	13/37	11.67	1	0.0006342
1	dbsnp	rs6664864	G	A	ALLELIC	14/6	13/37	11.67	1	0.0006342
1	dbsnp	rs3018410	A	G	ALLELIC	14/6	13/37	11.67	1	0.0006342
3	dbsnp	rs17273488	A	G	ALLELIC	14/6	13/37	11.67	1	0.0006342
6	dbsnp	rs1023276	A	G	ALLELIC	14/6	13/37	11.67	1	0.0006342
8	dbsnp	rs12680016	G	A	ALLELIC	14/6	13/37	11.67	1	0.0006342
23	dbsnp	rs5907636	G	A	ALLELIC	14/6	13/37	11.67	1	0.0006342
23	dbsnp	rs2719873	A	G	ALLELIC	14/6	13/37	11.67	1	0.0006342
1	dbsnp	rs11579521	A	G	ALLELIC	10/12	5/45	11.64	1	0.0006441
1	dbsnp	rs12404736	A	G	ALLELIC	10/12	5/45	11.64	1	0.0006441
1	dbsnp	rs1619058	C	A	ALLELIC	10/12	5/45	11.64	1	0.0006441
1	dbsnp	rs11208349	A	G	ALLELIC	10/12	5/45	11.64	1	0.0006441
1	dbsnp	rs211762	A	G	ALLELIC	10/12	5/45	11.64	1	0.0006441
1	dbsnp	rs7534846	G	A	ALLELIC	10/12	5/45	11.64	1	0.0006441
2	dbsnp	rs16858333	G	A	ALLELIC	10/12	5/45	11.64	1	0.0006441
2	dbsnp	rs972485	A	G	ALLELIC	10/12	5/45	11.64	1	0.0006441
2	dbsnp	rs6758770	G	A	ALLELIC	10/12	5/45	11.64	1	0.0006441
8	dbsnp	rs10503873	G	A	ALLELIC	10/12	5/45	11.64	1	0.0006441
9	dbsnp	rs517928	A	G	ALLELIC	10/12	5/45	11.64	1	0.0006441
15	dbsnp	rs692768	G	A	ALLELIC	10/12	5/45	11.64	1	0.0006441
15	dbsnp	rs11633454	A	G	ALLELIC	10/12	5/45	11.64	1	0.0006441
16	dbsnp	rs205162	A	G	ALLELIC	10/12	5/45	11.64	1	0.0006441
18	dbsnp	rs2460072	A	G	ALLELIC	10/12	5/45	11.64	1	0.0006441
18	dbsnp	rs11659363	C	A	ALLELIC	10/12	5/45	11.64	1	0.0006441
18	dbsnp	rs7229063	A	G	ALLELIC	10/12	5/45	11.64	1	0.0006441
22	dbsnp	rs17749540	A	G	ALLELIC	10/12	5/45	11.64	1	0.0006441
23	dbsnp	rs12859265	G	A	ALLELIC	10/12	5/45	11.64	1	0.0006441
23	dbsnp	rs6621220	G	A	ALLELIC	10/12	5/45	11.64	1	0.0006441
23	dbsnp	rs11796161	A	C	ALLELIC	10/12	5/45	11.64	1	0.0006441
23	dbsnp	rs11796157	G	A	ALLELIC	10/12	5/45	11.64	1	0.0006441
23	dbsnp	rs1005214	G	A	ALLELIC	10/12	5/45	11.64	1	0.0006441
23	dbsnp	rs1016738	A	G	ALLELIC	10/12	5/45	11.64	1	0.0006441
23	dbsnp	rs5945032	A	G	ALLELIC	10/12	5/45	11.64	1	0.0006441

23	dbsnp	rs11795948	G	A	ALLELIC	10/12	5/45	11.64	1	0.0006441
23	dbsnp	rs5977112	G	A	ALLELIC	10/12	5/45	11.64	1	0.0006441
8	dbsnp	rs7013846	C	A	ALLELIC	11/11	6/42	11.54	1	0.0006822
10	dbsnp	rs2541229	A	G	ALLELIC	8/8	2/28	11.52	1	0.0006896
11	dbsnp	rs6589988	G	A	ALLELIC	1/19	24/26	11.5	1	0.0006941
2	dbsnp	rs4313962	A	G	ALLELIC	5/17	33/17	11.48	1	0.000704
4	dbsnp	rs13435197	A	G	ALLELIC	17/5	17/33	11.48	1	0.000704
8	dbsnp	rs2005213	G	A	ALLELIC	17/5	17/33	11.48	1	0.000704
10	dbsnp	rs9423641	A	C	ALLELIC	17/5	17/33	11.48	1	0.000704
13	dbsnp	rs9554051	G	A	ALLELIC	17/5	17/33	11.48	1	0.000704
13	dbsnp	rs1218885	A	G	ALLELIC	5/17	33/17	11.48	1	0.000704
14	dbsnp	rs1110867	G	A	ALLELIC	17/5	17/33	11.48	1	0.000704
22	dbsnp	rs5756471	A	G	ALLELIC	17/5	17/33	11.48	1	0.000704
23	dbsnp	rs7892218	A	G	ALLELIC	17/5	17/33	11.48	1	0.000704
23	dbsnp	rs5909459	A	G	ALLELIC	17/5	17/33	11.48	1	0.000704
1	dbsnp	rs2785776	C	A	ALLELIC	15/7	13/37	11.44	1	0.0007194
1	dbsnp	rs1509703	A	G	ALLELIC	15/7	13/37	11.44	1	0.0007194
1	dbsnp	rs2000054	G	A	ALLELIC	15/7	13/37	11.44	1	0.0007194
1	dbsnp	rs7528316	A	G	ALLELIC	15/7	13/37	11.44	1	0.0007194
1	dbsnp	rs2369435	A	G	ALLELIC	15/7	13/37	11.44	1	0.0007194
2	dbsnp	rs6547465	G	A	ALLELIC	15/7	13/37	11.44	1	0.0007194
2	dbsnp	rs12470218	G	A	ALLELIC	15/7	13/37	11.44	1	0.0007194
2	dbsnp	rs7422703	A	G	ALLELIC	15/7	13/37	11.44	1	0.0007194
2	dbsnp	rs6713510	A	G	ALLELIC	15/7	13/37	11.44	1	0.0007194
8	dbsnp	rs12549294	A	G	ALLELIC	15/7	13/37	11.44	1	0.0007194
9	dbsnp	rs2209440	C	A	ALLELIC	15/7	13/37	11.44	1	0.0007194
1	dbsnp	rs4072431	A	G	ALLELIC	2/18	27/23	11.4	1	0.0007353
1	dbsnp	rs750439	G	A	ALLELIC	2/18	27/23	11.4	1	0.0007353
1	dbsnp	rs1080266	G	A	ALLELIC	16/6	15/35	11.38	1	0.000744
4	dbsnp	rs13110584	G	A	ALLELIC	16/6	15/35	11.38	1	0.000744
5	dbsnp	rs6887571	G	A	ALLELIC	16/6	15/35	11.38	1	0.000744
23	dbsnp	rs7884878	G	A	ALLELIC	16/6	15/35	11.38	1	0.000744
7	dbsnp	rs10238563	A	G	ALLELIC	0/22	19/31	11.36	1	0.0007516
2	dbsnp	rs3820720	G	A	ALLELIC	12/10	8/42	11.31	1	0.000769
2	dbsnp	rs2943641	A	G	ALLELIC	12/10	8/42	11.31	1	0.000769
2	dbsnp	rs2894597	G	A	ALLELIC	12/10	8/42	11.31	1	0.000769
5	dbsnp	rs766558	A	G	ALLELIC	12/10	8/42	11.31	1	0.000769
5	dbsnp	rs12654207	G	A	ALLELIC	12/10	8/42	11.31	1	0.000769
6	dbsnp	rs901418	A	G	ALLELIC	12/10	8/42	11.31	1	0.000769
8	dbsnp	rs2235116	G	A	ALLELIC	12/10	8/42	11.31	1	0.000769
9	dbsnp	rs17617904	A	G	ALLELIC	12/10	8/42	11.31	1	0.000769
14	dbsnp	rs11159148	A	G	ALLELIC	12/10	8/42	11.31	1	0.000769
14	dbsnp	rs11623090	G	A	ALLELIC	12/10	8/42	11.31	1	0.000769
15	dbsnp	rs4244896	A	G	ALLELIC	12/10	8/42	11.31	1	0.000769
23	dbsnp	rs7060369	A	G	ALLELIC	12/10	8/42	11.31	1	0.000769
23	dbsnp	rs5921097	G	A	ALLELIC	12/10	8/42	11.31	1	0.000769
23	dbsnp	rs7884358	G	A	ALLELIC	12/10	8/42	11.31	1	0.000769
9	dbsnp	rs210081	A	G	ALLELIC	16/4	17/31	11.23	1	0.000803

1	dbsnp	rs11261071	A	G	ALLELIC	0/20	20/30	11.2	1	0.000818
20	dbsnp	rs7272911	A	G	ALLELIC	0/20	20/30	11.2	1	0.000818
20	dbsnp	rs6108871	G	A	ALLELIC	3/19	27/21	11.19	1	0.0008241
8	dbsnp	rs732563	G	A	ALLELIC	3/19	28/22	11.18	1	0.0008254
15	dbsnp	rs12904870	A	G	ALLELIC	3/19	28/22	11.18	1	0.0008254
20	dbsnp	rs6040442	G	A	ALLELIC	3/19	28/22	11.18	1	0.0008254
23	dbsnp	rs17282598	A	G	ALLELIC	3/19	28/22	11.18	1	0.0008254
1	dbsnp	rs954472	A	G	ALLELIC	9/13	4/46	11.18	1	0.0008255
1	dbsnp	rs885141	A	G	ALLELIC	9/13	4/46	11.18	1	0.0008255
1	dbsnp	rs1336874	G	A	ALLELIC	9/13	4/46	11.18	1	0.0008255
1	dbsnp	rs6692148	G	A	ALLELIC	9/13	4/46	11.18	1	0.0008255
1	dbsnp	rs7530493	A	C	ALLELIC	9/13	4/46	11.18	1	0.0008255
5	dbsnp	rs2624417	A	C	ALLELIC	9/13	4/46	11.18	1	0.0008255
5	dbsnp	rs172438	A	G	ALLELIC	9/13	4/46	11.18	1	0.0008255
5	dbsnp	rs4597996	A	G	ALLELIC	9/13	4/46	11.18	1	0.0008255
5	dbsnp	rs4401597	A	G	ALLELIC	9/13	4/46	11.18	1	0.0008255
7	dbsnp	rs13228677	A	G	ALLELIC	9/13	4/46	11.18	1	0.0008255
9	dbsnp	rs10758063	A	G	ALLELIC	9/13	4/46	11.18	1	0.0008255
9	dbsnp	rs10465076	G	A	ALLELIC	9/13	4/46	11.18	1	0.0008255
9	dbsnp	rs13283404	A	G	ALLELIC	9/13	4/46	11.18	1	0.0008255
11	dbsnp	rs11026055	A	G	ALLELIC	9/13	4/46	11.18	1	0.0008255
11	dbsnp	rs11026086	A	G	ALLELIC	9/13	4/46	11.18	1	0.0008255
12	dbsnp	rs10861439	G	A	ALLELIC	9/13	4/46	11.18	1	0.0008255
13	dbsnp	rs9549448	G	A	ALLELIC	9/13	4/46	11.18	1	0.0008255
15	dbsnp	rs11632161	A	G	ALLELIC	9/13	4/46	11.18	1	0.0008255
15	dbsnp	rs2615194	G	A	ALLELIC	9/13	4/46	11.18	1	0.0008255
15	dbsnp	rs12901555	A	G	ALLELIC	9/13	4/46	11.18	1	0.0008255
23	dbsnp	rs1617688	G	A	ALLELIC	9/13	4/46	11.18	1	0.0008255
23	dbsnp	rs6640047	G	A	ALLELIC	9/13	4/46	11.18	1	0.0008255
23	dbsnp	rs5927915	C	A	ALLELIC	9/13	4/46	11.18	1	0.0008255
23	dbsnp	rs5924360	C	A	ALLELIC	9/13	4/46	11.18	1	0.0008255
23	dbsnp	rs4825476	G	A	ALLELIC	9/13	4/46	11.18	1	0.0008255
23	dbsnp	rs5975024	G	A	ALLELIC	9/13	4/46	11.18	1	0.0008255
23	dbsnp	rs9781523	A	G	ALLELIC	9/13	4/46	11.18	1	0.0008255
1	dbsnp	rs10864302	A	G	ALLELIC	6/16	1/49	11.12	1	0.000855
1	dbsnp	rs205485	A	C	ALLELIC	6/16	1/49	11.12	1	0.000855
1	dbsnp	rs616546	G	A	ALLELIC	6/16	1/49	11.12	1	0.000855
1	dbsnp	rs522821	A	G	ALLELIC	6/16	1/49	11.12	1	0.000855
1	dbsnp	rs544621	A	C	ALLELIC	6/16	1/49	11.12	1	0.000855
1	dbsnp	rs3814302	G	A	ALLELIC	6/16	1/49	11.12	1	0.000855
1	dbsnp	rs2275247	G	A	ALLELIC	6/16	1/49	11.12	1	0.000855
1	dbsnp	rs6696836	G	A	ALLELIC	6/16	1/49	11.12	1	0.000855
1	dbsnp	rs10908401	C	A	ALLELIC	6/16	1/49	11.12	1	0.000855
1	dbsnp	rs677661	G	A	ALLELIC	6/16	1/49	11.12	1	0.000855
1	dbsnp	rs1889759	G	A	ALLELIC	6/16	1/49	11.12	1	0.000855
2	dbsnp	rs17672973	A	G	ALLELIC	6/16	1/49	11.12	1	0.000855
2	dbsnp	rs13019896	A	G	ALLELIC	6/16	1/49	11.12	1	0.000855
2	dbsnp	rs12185625	G	A	ALLELIC	6/16	1/49	11.12	1	0.000855

3	dbsnp	rs3774490	A	G	ALLELIC	6/16	1/49	11.12	1	0.000855
4	dbsnp	rs6815347	A	C	ALLELIC	6/16	1/49	11.12	1	0.000855
4	dbsnp	rs11724057	A	G	ALLELIC	6/16	1/49	11.12	1	0.000855
4	dbsnp	rs12513116	A	C	ALLELIC	6/16	1/49	11.12	1	0.000855
7	dbsnp	rs12704841	A	G	ALLELIC	6/16	1/49	11.12	1	0.000855
7	dbsnp	rs3108433	A	G	ALLELIC	6/16	1/49	11.12	1	0.000855
7	dbsnp	rs3108440	A	G	ALLELIC	6/16	1/49	11.12	1	0.000855
7	dbsnp	rs1263550	G	A	ALLELIC	6/16	1/49	11.12	1	0.000855
7	dbsnp	rs1242786	A	G	ALLELIC	6/16	1/49	11.12	1	0.000855
8	dbsnp	rs2898250	A	G	ALLELIC	6/16	1/49	11.12	1	0.000855
8	dbsnp	rs6984094	G	A	ALLELIC	6/16	1/49	11.12	1	0.000855
8	dbsnp	rs10112573	G	A	ALLELIC	6/16	1/49	11.12	1	0.000855
8	dbsnp	rs264824	A	G	ALLELIC	6/16	1/49	11.12	1	0.000855
10	dbsnp	rs619319	G	A	ALLELIC	6/16	1/49	11.12	1	0.000855
10	dbsnp	rs3781141	A	G	ALLELIC	6/16	1/49	11.12	1	0.000855
11	dbsnp	rs925005	A	G	ALLELIC	6/16	1/49	11.12	1	0.000855
11	dbsnp	rs16919815	A	G	ALLELIC	6/16	1/49	11.12	1	0.000855
12	dbsnp	rs719840	C	A	ALLELIC	6/16	1/49	11.12	1	0.000855
12	dbsnp	rs10772590	A	G	ALLELIC	6/16	1/49	11.12	1	0.000855
12	dbsnp	rs10785556	A	G	ALLELIC	6/16	1/49	11.12	1	0.000855
12	dbsnp	rs11182916	A	G	ALLELIC	6/16	1/49	11.12	1	0.000855
12	dbsnp	rs11182918	G	A	ALLELIC	6/16	1/49	11.12	1	0.000855
13	dbsnp	rs17196064	A	G	ALLELIC	6/16	1/49	11.12	1	0.000855
14	dbsnp	rs1253704	A	G	ALLELIC	6/16	1/49	11.12	1	0.000855
15	dbsnp	rs17782975	G	A	ALLELIC	6/16	1/49	11.12	1	0.000855
16	dbsnp	rs12445976	G	A	ALLELIC	6/16	1/49	11.12	1	0.000855
16	dbsnp	rs1500356	G	A	ALLELIC	6/16	1/49	11.12	1	0.000855
17	dbsnp	rs17803227	A	G	ALLELIC	6/16	1/49	11.12	1	0.000855
19	dbsnp	rs11667173	G	A	ALLELIC	6/16	1/49	11.12	1	0.000855
22	dbsnp	rs5997027	A	G	ALLELIC	6/16	1/49	11.12	1	0.000855
22	dbsnp	rs6004904	A	G	ALLELIC	6/16	1/49	11.12	1	0.000855
22	dbsnp	rs5754819	G	A	ALLELIC	6/16	1/49	11.12	1	0.000855
23	dbsnp	rs12391871	A	G	ALLELIC	6/16	1/49	11.12	1	0.000855
23	dbsnp	rs4827120	G	A	ALLELIC	6/16	1/49	11.12	1	0.000855
23	dbsnp	rs5912276	G	A	ALLELIC	6/16	1/49	11.12	1	0.000855
23	dbsnp	rs2220117	G	A	ALLELIC	6/16	1/49	11.12	1	0.000855
4	dbsnp	rs4437231	C	A	ALLELIC	16/4	18/32	11.07	1	0.0008765
11	dbsnp	rs4910031	A	G	ALLELIC	4/16	32/18	11.07	1	0.0008765
1	dbsnp	rs2699385	G	A	ALLELIC	10/12	5/43	11	1	0.0009112
1	dbsnp	rs2275447	G	A	ALLELIC	11/9	8/42	10.99	1	0.0009172
3	dbsnp	rs7622900	G	A	ALLELIC	11/9	8/42	10.99	1	0.0009172
14	dbsnp	rs10135750	G	A	ALLELIC	11/9	8/42	10.99	1	0.0009172
22	dbsnp	rs9616810	A	G	ALLELIC	11/9	8/42	10.99	1	0.0009172
1	dbsnp	rs9435854	G	A	ALLELIC	1/21	22/28	10.94	1	0.0009414
1	dbsnp	rs9435856	A	C	ALLELIC	1/21	22/28	10.94	1	0.0009414
1	dbsnp	rs9435858	G	A	ALLELIC	1/21	22/28	10.94	1	0.0009414
2	dbsnp	rs6435689	A	G	ALLELIC	1/21	22/28	10.94	1	0.0009414
3	dbsnp	rs711578	G	A	ALLELIC	1/21	22/28	10.94	1	0.0009414

4	dbsnp	rs4862523	G	A	ALLELIC	1/21	22/28	10.94	1	0.0009414
5	dbsnp	rs6601018	A	G	ALLELIC	1/21	22/28	10.94	1	0.0009414
5	dbsnp	rs2411906	G	A	ALLELIC	1/21	22/28	10.94	1	0.0009414
7	dbsnp	rs1729831	G	A	ALLELIC	1/21	22/28	10.94	1	0.0009414
8	dbsnp	rs7815138	G	A	ALLELIC	1/21	22/28	10.94	1	0.0009414
10	dbsnp	rs7905986	A	C	ALLELIC	9/11	5/45	10.94	1	0.0009424
11	dbsnp	rs5030779	A	G	ALLELIC	9/11	5/45	10.94	1	0.0009424
13	dbsnp	rs17078885	G	A	ALLELIC	9/11	5/45	10.94	1	0.0009424
13	dbsnp	rs7317974	A	G	ALLELIC	9/11	5/45	10.94	1	0.0009424
16	dbsnp	rs11074714	G	A	ALLELIC	9/11	5/45	10.94	1	0.0009424
18	dbsnp	rs1010800	G	A	ALLELIC	9/11	5/45	10.94	1	0.0009424
21	dbsnp	rs2838870	A	G	ALLELIC	9/11	5/45	10.94	1	0.0009424
0	dbsnp	rs913356	C	A	ALLELIC	2/20	25/25	10.91	1	0.0009569
2	dbsnp	rs6435690	G	A	ALLELIC	2/20	25/25	10.91	1	0.0009569
5	dbsnp	rs12516622	C	A	ALLELIC	2/20	25/25	10.91	1	0.0009569
7	dbsnp	rs2727744	G	A	ALLELIC	2/20	25/25	10.91	1	0.0009569
8	dbsnp	rs2090789	A	G	ALLELIC	2/20	25/25	10.91	1	0.0009569
20	dbsnp	rs6040436	A	G	ALLELIC	2/20	25/25	10.91	1	0.0009569
21	dbsnp	rs2091886	G	A	ALLELIC	14/8	11/37	10.89	1	0.0009644
1	dbsnp	rs3890825	G	A	ALLELIC	8/14	3/47	10.88	1	0.000971
1	dbsnp	rs10783011	A	G	ALLELIC	8/14	3/47	10.88	1	0.000971
1	dbsnp	rs16827043	G	A	ALLELIC	8/14	3/47	10.88	1	0.000971
3	dbsnp	rs2054873	G	A	ALLELIC	8/14	3/47	10.88	1	0.000971
4	dbsnp	rs2126854	A	G	ALLELIC	8/14	3/47	10.88	1	0.000971
5	dbsnp	rs6884205	A	C	ALLELIC	8/14	3/47	10.88	1	0.000971
6	dbsnp	rs4609015	G	A	ALLELIC	8/14	3/47	10.88	1	0.000971
6	dbsnp	rs7770214	A	G	ALLELIC	8/14	3/47	10.88	1	0.000971
6	dbsnp	rs6900725	G	A	ALLELIC	8/14	3/47	10.88	1	0.000971
6	dbsnp	rs4712993	A	G	ALLELIC	8/14	3/47	10.88	1	0.000971
6	dbsnp	rs7744254	G	A	ALLELIC	8/14	3/47	10.88	1	0.000971
6	dbsnp	rs6456721	G	A	ALLELIC	8/14	3/47	10.88	1	0.000971
6	dbsnp	rs12191917	A	G	ALLELIC	8/14	3/47	10.88	1	0.000971
6	dbsnp	rs2393652	G	A	ALLELIC	8/14	3/47	10.88	1	0.000971
6	dbsnp	rs2893845	A	G	ALLELIC	8/14	3/47	10.88	1	0.000971
6	dbsnp	rs7751280	A	G	ALLELIC	8/14	3/47	10.88	1	0.000971
6	dbsnp	rs10946829	A	G	ALLELIC	8/14	3/47	10.88	1	0.000971
6	dbsnp	rs3846843	A	G	ALLELIC	8/14	3/47	10.88	1	0.000971
6	dbsnp	rs2893847	A	C	ALLELIC	8/14	3/47	10.88	1	0.000971
6	dbsnp	rs2893848	G	A	ALLELIC	8/14	3/47	10.88	1	0.000971
6	dbsnp	rs17539219	C	A	ALLELIC	8/14	3/47	10.88	1	0.000971
6	dbsnp	rs4712996	G	A	ALLELIC	8/14	3/47	10.88	1	0.000971
6	dbsnp	rs17611438	A	C	ALLELIC	8/14	3/47	10.88	1	0.000971
6	dbsnp	rs3846848	A	G	ALLELIC	8/14	3/47	10.88	1	0.000971
6	dbsnp	rs12212943	A	G	ALLELIC	8/14	3/47	10.88	1	0.000971
6	dbsnp	rs543363	A	G	ALLELIC	8/14	3/47	10.88	1	0.000971
7	dbsnp	rs10952955	A	C	ALLELIC	8/14	3/47	10.88	1	0.000971
8	dbsnp	rs9657422	G	A	ALLELIC	8/14	3/47	10.88	1	0.000971
9	dbsnp	rs874183	C	A	ALLELIC	8/14	3/47	10.88	1	0.000971

10	dbsnp	rs1168581	A	G	ALLELIC	8/14	3/47	10.88	1	0.000971
11	dbsnp	rs7115946	G	A	ALLELIC	8/14	3/47	10.88	1	0.000971
12	dbsnp	rs870779	A	C	ALLELIC	8/14	3/47	10.88	1	0.000971
14	dbsnp	rs1242094	C	A	ALLELIC	8/14	3/47	10.88	1	0.000971
15	dbsnp	rs266305	G	A	ALLELIC	8/14	3/47	10.88	1	0.000971
15	dbsnp	rs12907153	A	G	ALLELIC	8/14	3/47	10.88	1	0.000971
16	dbsnp	rs8064029	A	G	ALLELIC	8/14	3/47	10.88	1	0.000971
16	dbsnp	rs1045643	A	G	ALLELIC	8/14	3/47	10.88	1	0.000971
17	dbsnp	rs277061	C	A	ALLELIC	8/14	3/47	10.88	1	0.000971
17	dbsnp	rs277067	A	G	ALLELIC	8/14	3/47	10.88	1	0.000971
23	dbsnp	rs964481	G	A	ALLELIC	8/14	3/47	10.88	1	0.000971
23	dbsnp	rs5971423	G	A	ALLELIC	8/14	3/47	10.88	1	0.000971
23	dbsnp	rs7052771	A	G	ALLELIC	8/14	3/47	10.88	1	0.000971
1	dbsnp	rs2040433	G	A	ALLELIC	7/15	2/48	10.81	1	0.00101
1	dbsnp	rs2746347	A	G	ALLELIC	7/15	2/48	10.81	1	0.00101
2	dbsnp	rs2255293	A	G	ALLELIC	7/15	2/48	10.81	1	0.00101
2	dbsnp	rs7605442	A	G	ALLELIC	7/15	2/48	10.81	1	0.00101
4	dbsnp	rs12503110	A	G	ALLELIC	7/15	2/48	10.81	1	0.00101
5	dbsnp	rs13180609	A	G	ALLELIC	7/15	2/48	10.81	1	0.00101
6	dbsnp	rs11967105	G	A	ALLELIC	7/15	2/48	10.81	1	0.00101
7	dbsnp	rs4732432	G	A	ALLELIC	7/15	2/48	10.81	1	0.00101
7	dbsnp	rs12666394	A	C	ALLELIC	7/15	2/48	10.81	1	0.00101
7	dbsnp	rs2216982	A	G	ALLELIC	7/15	2/48	10.81	1	0.00101
8	dbsnp	rs12679786	G	A	ALLELIC	7/15	2/48	10.81	1	0.00101
8	dbsnp	rs6473383	A	G	ALLELIC	7/15	2/48	10.81	1	0.00101
9	dbsnp	rs7039708	A	G	ALLELIC	7/15	2/48	10.81	1	0.00101
10	dbsnp	rs2068043	A	G	ALLELIC	7/15	2/48	10.81	1	0.00101
10	dbsnp	rs1442550	A	C	ALLELIC	7/15	2/48	10.81	1	0.00101
11	dbsnp	rs12418506	A	G	ALLELIC	7/15	2/48	10.81	1	0.00101
11	dbsnp	rs6649	A	G	ALLELIC	7/15	2/48	10.81	1	0.00101
12	dbsnp	rs6539475	C	A	ALLELIC	7/15	2/48	10.81	1	0.00101
12	dbsnp	rs7972050	A	G	ALLELIC	7/15	2/48	10.81	1	0.00101
12	dbsnp	rs11066865	A	G	ALLELIC	7/15	2/48	10.81	1	0.00101
13	dbsnp	rs4147057	A	C	ALLELIC	7/15	2/48	10.81	1	0.00101
13	dbsnp	rs2151380	A	G	ALLELIC	7/15	2/48	10.81	1	0.00101
14	dbsnp	rs17104366	A	G	ALLELIC	7/15	2/48	10.81	1	0.00101
15	dbsnp	rs12438794	A	G	ALLELIC	7/15	2/48	10.81	1	0.00101
15	dbsnp	rs8041665	A	G	ALLELIC	7/15	2/48	10.81	1	0.00101
15	dbsnp	rs8037309	A	G	ALLELIC	7/15	2/48	10.81	1	0.00101
15	dbsnp	rs1565563	A	G	ALLELIC	7/15	2/48	10.81	1	0.00101
18	dbsnp	rs2941840	A	G	ALLELIC	7/15	2/48	10.81	1	0.00101
20	dbsnp	rs6090818	C	A	ALLELIC	7/15	2/48	10.81	1	0.00101
20	dbsnp	rs4810051	A	G	ALLELIC	7/15	2/48	10.81	1	0.00101
20	dbsnp	rs404727	A	G	ALLELIC	7/15	2/48	10.81	1	0.00101
23	dbsnp	rs12842919	C	A	ALLELIC	7/15	2/48	10.81	1	0.00101
23	dbsnp	rs12834033	G	A	ALLELIC	7/15	2/48	10.81	1	0.00101
23	dbsnp	rs7058600	G	A	ALLELIC	7/15	2/48	10.81	1	0.00101
1	dbsnp	rs10800431	A	G	ALLELIC	13/9	10/40	10.74	1	0.001049

1	dbsnp	rs7538259	A	G	ALLELIC	13/9	10/40	10.74	1	0.001049
2	dbsnp	rs2020037	C	A	ALLELIC	13/9	10/40	10.74	1	0.001049
2	dbsnp	rs2943657	G	A	ALLELIC	13/9	10/40	10.74	1	0.001049
2	dbsnp	rs2943658	A	G	ALLELIC	13/9	10/40	10.74	1	0.001049
2	dbsnp	rs2943659	G	A	ALLELIC	13/9	10/40	10.74	1	0.001049
2	dbsnp	rs13402622	G	A	ALLELIC	13/9	10/40	10.74	1	0.001049
5	dbsnp	rs11951225	G	A	ALLELIC	13/9	10/40	10.74	1	0.001049
7	dbsnp	rs505245	G	A	ALLELIC	13/9	10/40	10.74	1	0.001049
8	dbsnp	rs3015729	G	A	ALLELIC	13/9	10/40	10.74	1	0.001049
9	dbsnp	rs2383044	A	G	ALLELIC	13/9	10/40	10.74	1	0.001049
12	dbsnp	rs2579257	A	G	ALLELIC	13/9	10/40	10.74	1	0.001049
12	dbsnp	rs2579260	G	A	ALLELIC	13/9	10/40	10.74	1	0.001049
12	dbsnp	rs1298698	A	G	ALLELIC	13/9	10/40	10.74	1	0.001049
13	dbsnp	rs431103	G	A	ALLELIC	13/9	10/40	10.74	1	0.001049
15	dbsnp	rs4932557	A	C	ALLELIC	13/9	10/40	10.74	1	0.001049
22	dbsnp	rs2235324	G	A	ALLELIC	13/9	10/40	10.74	1	0.001049
22	dbsnp	rs2413451	G	A	ALLELIC	13/9	10/40	10.74	1	0.001049
22	dbsnp	rs2743825	C	A	ALLELIC	13/9	10/40	10.74	1	0.001049
23	dbsnp	rs1014178	A	G	ALLELIC	13/9	10/40	10.74	1	0.001049
1	dbsnp	rs2996295	A	G	ALLELIC	4/18	30/20	10.72	1	0.00106
2	dbsnp	rs1834216	G	A	ALLELIC	4/18	30/20	10.72	1	0.00106
6	dbsnp	rs9450485	A	G	ALLELIC	4/18	30/20	10.72	1	0.00106
8	dbsnp	rs2947255	G	A	ALLELIC	18/4	20/30	10.72	1	0.00106
12	dbsnp	rs1469664	A	C	ALLELIC	4/18	30/20	10.72	1	0.00106
18	dbsnp	rs8093150	C	A	ALLELIC	4/18	30/20	10.72	1	0.00106
21	dbsnp	rs2824224	A	G	ALLELIC	4/18	30/20	10.72	1	0.00106
1	dbsnp	rs7541947	A	G	ALLELIC	15/5	16/34	10.71	1	0.001068
3	dbsnp	rs7374735	A	G	ALLELIC	15/5	16/34	10.71	1	0.001068
10	dbsnp	rs34123069	A	C	ALLELIC	15/5	16/34	10.71	1	0.001068
12	dbsnp	rs4491324	A	G	ALLELIC	15/5	16/34	10.71	1	0.001068
15	dbsnp	rs11072447	G	A	ALLELIC	15/5	16/34	10.71	1	0.001068
15	dbsnp	rs11639300	A	G	ALLELIC	15/5	16/34	10.71	1	0.001068
23	dbsnp	rs6620925	A	G	ALLELIC	15/5	16/34	10.71	1	0.001068
2	dbsnp	rs12622595	G	A	ALLELIC	1/19	23/27	10.66	1	0.001096
3	dbsnp	rs13080353	A	G	ALLELIC	1/19	23/27	10.66	1	0.001096
3	dbsnp	rs12494491	A	C	ALLELIC	1/19	23/27	10.66	1	0.001096
1	dbsnp	rs1050316	C	A	ALLELIC	3/17	29/21	10.64	1	0.001104
13	dbsnp	rs11147624	G	A	ALLELIC	3/17	29/21	10.64	1	0.001104
13	dbsnp	rs11147625	G	A	ALLELIC	3/17	29/21	10.64	1	0.001104
16	dbsnp	rs6501143	A	C	ALLELIC	3/17	29/21	10.64	1	0.001104
16	dbsnp	rs7201055	A	G	ALLELIC	3/17	29/21	10.64	1	0.001104
16	dbsnp	rs7200772	A	G	ALLELIC	3/17	29/21	10.64	1	0.001104
1	dbsnp	rs953035	A	G	ALLELIC	6/16	1/47	10.64	1	0.001109
18	dbsnp	rs213070	G	A	ALLELIC	6/16	1/47	10.64	1	0.001109
1	dbsnp	rs3789689	C	A	ALLELIC	4/16	0/50	10.61	1	0.001127
1	dbsnp	rs1778193	A	G	ALLELIC	4/16	0/50	10.61	1	0.001127
2	dbsnp	rs10496080	C	A	ALLELIC	4/16	0/50	10.61	1	0.001127
4	dbsnp	rs6531972	C	A	ALLELIC	12/8	10/40	10.61	1	0.001127

5	dbsnp	rs2937761	G	A	ALLELIC	4/16	0/50	10.61	1	0.001127
7	dbsnp	rs11767642	A	C	ALLELIC	12/8	10/40	10.61	1	0.001127
7	dbsnp	rs13223714	G	A	ALLELIC	12/8	10/40	10.61	1	0.001127
8	dbsnp	rs1482207	G	A	ALLELIC	4/16	0/50	10.61	1	0.001127
8	dbsnp	rs11775579	G	A	ALLELIC	12/8	10/40	10.61	1	0.001127
9	dbsnp	rs2472519	G	A	ALLELIC	12/10	8/40	10.61	1	0.001127
11	dbsnp	rs4636701	A	G	ALLELIC	4/16	0/50	10.61	1	0.001127
11	dbsnp	rs878611	A	G	ALLELIC	4/16	0/50	10.61	1	0.001127
12	dbsnp	rs17302889	G	A	ALLELIC	4/16	0/50	10.61	1	0.001127
12	dbsnp	rs12298894	C	A	ALLELIC	4/16	0/50	10.61	1	0.001127
14	dbsnp	rs1289253	A	G	ALLELIC	12/8	10/40	10.61	1	0.001127
16	dbsnp	rs3891920	G	A	ALLELIC	4/16	0/50	10.61	1	0.001127
16	dbsnp	rs4888200	G	A	ALLELIC	4/16	0/50	10.61	1	0.001127
19	dbsnp	rs1531517	A	G	ALLELIC	4/16	0/50	10.61	1	0.001127
20	dbsnp	rs6030128	A	G	ALLELIC	4/16	0/50	10.61	1	0.001127
23	dbsnp	rs3810682	C	G	ALLELIC	12/8	10/40	10.61	1	0.001127
1	dbsnp	rs10800328	G	A	ALLELIC	9/13	4/44	10.59	1	0.001139
23	dbsnp	rs6629492	A	G	ALLELIC	1/21	20/26	10.57	1	0.00115
1	dbsnp	rs6673711	G	A	ALLELIC	11/11	7/43	10.56	1	0.001156
1	dbsnp	rs7515752	G	A	ALLELIC	11/11	7/43	10.56	1	0.001156
1	dbsnp	rs834343	C	A	ALLELIC	11/11	7/43	10.56	1	0.001156
1	dbsnp	rs6426525	A	G	ALLELIC	11/11	7/43	10.56	1	0.001156
1	dbsnp	rs11804896	G	A	ALLELIC	11/11	7/43	10.56	1	0.001156
2	dbsnp	rs736687	G	A	ALLELIC	0/22	18/32	10.56	1	0.001156
2	dbsnp	rs12613541	G	A	ALLELIC	11/11	7/43	10.56	1	0.001156
2	dbsnp	rs4848142	A	G	ALLELIC	11/11	7/43	10.56	1	0.001156
2	dbsnp	rs11897843	G	A	ALLELIC	11/11	7/43	10.56	1	0.001156
2	dbsnp	rs900439	C	A	ALLELIC	11/11	7/43	10.56	1	0.001156
3	dbsnp	rs355057	A	C	ALLELIC	11/11	7/43	10.56	1	0.001156
3	dbsnp	rs357139	A	G	ALLELIC	11/11	7/43	10.56	1	0.001156
3	dbsnp	rs12638211	G	A	ALLELIC	11/11	7/43	10.56	1	0.001156
6	dbsnp	rs571299	A	G	ALLELIC	11/11	7/43	10.56	1	0.001156
6	dbsnp	rs7756226	A	G	ALLELIC	11/11	7/43	10.56	1	0.001156
7	dbsnp	rs4484577	A	C	ALLELIC	11/11	7/43	10.56	1	0.001156
7	dbsnp	rs896170	G	A	ALLELIC	11/11	7/43	10.56	1	0.001156
7	dbsnp	rs1002292	C	A	ALLELIC	11/11	7/43	10.56	1	0.001156
8	dbsnp	rs17698981	A	C	ALLELIC	11/11	7/43	10.56	1	0.001156
8	dbsnp	rs10504943	A	G	ALLELIC	11/11	7/43	10.56	1	0.001156
8	dbsnp	rs4339605	G	A	ALLELIC	11/11	7/43	10.56	1	0.001156
9	dbsnp	rs6479020	C	A	ALLELIC	11/11	7/43	10.56	1	0.001156
10	dbsnp	rs4748453	A	G	ALLELIC	11/11	7/43	10.56	1	0.001156
12	dbsnp	rs7972545	A	G	ALLELIC	11/11	7/43	10.56	1	0.001156
12	dbsnp	rs7309603	A	G	ALLELIC	11/11	7/43	10.56	1	0.001156
12	dbsnp	rs11105483	A	G	ALLELIC	11/11	7/43	10.56	1	0.001156
13	dbsnp	rs10976	A	G	ALLELIC	11/11	7/43	10.56	1	0.001156
13	dbsnp	rs17054541	A	G	ALLELIC	11/11	7/43	10.56	1	0.001156
13	dbsnp	rs516144	C	A	ALLELIC	11/11	7/43	10.56	1	0.001156
13	dbsnp	rs9518476	A	G	ALLELIC	11/11	7/43	10.56	1	0.001156

16	dbsnp	rs2646130	G	A	ALLELIC	11/11	7/43	10.56	1	0.001156
17	dbsnp	rs320637	G	A	ALLELIC	11/11	7/43	10.56	1	0.001156
23	dbsnp	rs1048118	A	G	ALLELIC	11/11	7/43	10.56	1	0.001156
23	dbsnp	rs2235182	G	A	ALLELIC	11/11	7/43	10.56	1	0.001156
23	dbsnp	rs8177079	G	A	ALLELIC	11/11	7/43	10.56	1	0.001156
23	dbsnp	rs2362162	A	G	ALLELIC	0/22	18/32	10.56	1	0.001156
10	dbsnp	rs4934956	A	G	ALLELIC	16/6	15/33	10.52	1	0.001182
23	dbsnp	rs4893257	G	A	ALLELIC	16/6	15/33	10.52	1	0.001182
1	dbsnp	rs12088073	C	A	ALLELIC	14/6	14/36	10.5	1	0.001194
1	dbsnp	rs6425284	A	G	ALLELIC	14/6	14/36	10.5	1	0.001194
1	dbsnp	rs10157433	G	A	ALLELIC	2/18	26/24	10.5	1	0.001194
2	dbsnp	rs6738304	A	G	ALLELIC	2/18	26/24	10.5	1	0.001194
3	dbsnp	rs317572	G	A	ALLELIC	2/18	26/24	10.5	1	0.001194
3	dbsnp	rs7630344	A	G	ALLELIC	14/6	14/36	10.5	1	0.001194
4	dbsnp	rs4355345	A	G	ALLELIC	2/18	26/24	10.5	1	0.001194
5	dbsnp	rs88183	A	G	ALLELIC	2/18	26/24	10.5	1	0.001194
5	dbsnp	rs155796	A	G	ALLELIC	2/18	26/24	10.5	1	0.001194
16	dbsnp	rs6500165	A	G	ALLELIC	14/6	14/36	10.5	1	0.001194
18	dbsnp	rs12969516	G	A	ALLELIC	14/6	14/36	10.5	1	0.001194
23	dbsnp	rs5934845	A	C	ALLELIC	2/18	26/24	10.5	1	0.001194
1	dbsnp	rs2038025	A	G	ALLELIC	13/7	12/38	10.46	1	0.00122
1	dbsnp	rs1793319	A	G	ALLELIC	13/7	12/38	10.46	1	0.00122
1	dbsnp	rs6688577	A	G	ALLELIC	13/7	12/38	10.46	1	0.00122
2	dbsnp	rs10932837	G	A	ALLELIC	13/7	12/38	10.46	1	0.00122
6	dbsnp	rs1485042	C	A	ALLELIC	13/7	12/38	10.46	1	0.00122
11	dbsnp	rs11035061	G	A	ALLELIC	13/7	12/38	10.46	1	0.00122
12	dbsnp	rs7296925	A	G	ALLELIC	13/7	12/38	10.46	1	0.00122
12	dbsnp	rs2578467	A	G	ALLELIC	13/7	12/38	10.46	1	0.00122
23	dbsnp	rs1921396	A	C	ALLELIC	13/7	12/38	10.46	1	0.00122
11	dbsnp	rs11033544	A	G	ALLELIC	6/12	2/46	10.45	1	0.001223
5	dbsnp	rs2548627	A	G	ALLELIC	6/8	2/34	10.44	1	0.001236
3	dbsnp	rs10510380	G	A	ALLELIC	0/20	19/31	10.43	1	0.001239
4	dbsnp	rs10520563	G	A	ALLELIC	0/20	19/31	10.43	1	0.001239
23	dbsnp	rs12015040	G	A	ALLELIC	0/20	19/31	10.43	1	0.001239
23	dbsnp	rs731326	G	A	ALLELIC	0/20	19/31	10.43	1	0.001239
1	dbsnp	rs815730	A	C	ALLELIC	17/5	18/32	10.42	1	0.001248
1	dbsnp	rs845629	G	A	ALLELIC	17/5	18/32	10.42	1	0.001248
2	dbsnp	rs984578	A	G	ALLELIC	5/17	32/18	10.42	1	0.001248
2	dbsnp	rs10169352	G	A	ALLELIC	17/5	18/32	10.42	1	0.001248
2	dbsnp	rs9309383	A	G	ALLELIC	17/5	18/32	10.42	1	0.001248
5	dbsnp	rs25994	G	A	ALLELIC	17/5	18/32	10.42	1	0.001248
6	dbsnp	rs1325183	G	A	ALLELIC	5/17	32/18	10.42	1	0.001248
6	dbsnp	rs1325182	A	C	ALLELIC	17/5	18/32	10.42	1	0.001248
6	dbsnp	rs1325181	C	A	ALLELIC	5/17	32/18	10.42	1	0.001248
6	dbsnp	rs13202251	A	G	ALLELIC	5/17	32/18	10.42	1	0.001248
8	dbsnp	rs2898280	G	A	ALLELIC	5/17	32/18	10.42	1	0.001248
9	dbsnp	rs1182653	A	C	ALLELIC	17/5	18/32	10.42	1	0.001248
18	dbsnp	rs2156650	A	G	ALLELIC	17/5	18/32	10.42	1	0.001248

21	dbsnp	rs2822537	A	G	ALLELIC	17/5	18/32	10.42	1	0.001248
21	dbsnp	rs2822538	G	A	ALLELIC	17/5	18/32	10.42	1	0.001248
23	dbsnp	rs5909377	A	G	ALLELIC	17/5	18/32	10.42	1	0.001248
23	dbsnp	rs12013261	G	A	ALLELIC	17/5	18/32	10.42	1	0.001248
1	dbsnp	rs11261070	C	A	ALLELIC	14/8	12/38	10.4	1	0.001258
2	dbsnp	rs332925	G	A	ALLELIC	14/8	12/38	10.4	1	0.001258
2	dbsnp	rs10192798	A	C	ALLELIC	14/8	12/38	10.4	1	0.001258
2	dbsnp	rs207916	G	A	ALLELIC	14/8	12/38	10.4	1	0.001258
2	dbsnp	rs958960	C	A	ALLELIC	14/8	12/38	10.4	1	0.001258
4	dbsnp	rs11730030	A	G	ALLELIC	14/8	12/38	10.4	1	0.001258
6	dbsnp	rs793834	A	G	ALLELIC	14/8	12/38	10.4	1	0.001258
6	dbsnp	rs1200425	A	G	ALLELIC	14/8	12/38	10.4	1	0.001258
6	dbsnp	rs1144168	A	G	ALLELIC	14/8	12/38	10.4	1	0.001258
8	dbsnp	rs7002465	A	G	ALLELIC	14/8	12/38	10.4	1	0.001258
9	dbsnp	rs7029249	A	G	ALLELIC	14/8	12/38	10.4	1	0.001258
10	dbsnp	rs10823229	G	A	ALLELIC	14/8	12/38	10.4	1	0.001258
10	dbsnp	rs10823231	A	G	ALLELIC	14/8	12/38	10.4	1	0.001258
10	dbsnp	rs7894063	G	A	ALLELIC	14/8	12/38	10.4	1	0.001258
13	dbsnp	rs7985661	A	G	ALLELIC	14/8	12/38	10.4	1	0.001258
13	dbsnp	rs12860317	A	G	ALLELIC	14/8	12/38	10.4	1	0.001258
14	dbsnp	rs1984139	A	C	ALLELIC	14/8	12/38	10.4	1	0.001258
20	dbsnp	rs6087445	A	G	ALLELIC	14/8	12/38	10.4	1	0.001258
20	dbsnp	rs6092361	A	G	ALLELIC	14/8	12/38	10.4	1	0.001258
23	dbsnp	rs12387077	G	A	ALLELIC	14/8	12/38	10.4	1	0.001258
23	dbsnp	rs2266879	G	A	ALLELIC	14/8	12/38	10.4	1	0.001258
7	dbsnp	rs12702842	G	A	ALLELIC	11/9	7/37	10.39	1	0.001264
6	dbsnp	rs13211518	A	G	ALLELIC	8/14	3/45	10.33	1	0.00131
9	dbsnp	rs17774840	G	A	ALLELIC	8/14	3/45	10.33	1	0.00131
12	dbsnp	rs7970004	A	G	ALLELIC	8/14	3/45	10.33	1	0.00131
17	dbsnp	rs3111847	G	A	ALLELIC	8/14	3/45	10.33	1	0.00131
1	dbsnp	rs12065389	G	A	ALLELIC	8/12	4/46	10.3	1	0.001331
1	dbsnp	rs12021710	A	G	ALLELIC	8/12	4/46	10.3	1	0.001331
7	dbsnp	rs273129	C	A	ALLELIC	8/12	4/46	10.3	1	0.001331
8	dbsnp	rs7812637	G	A	ALLELIC	8/12	4/46	10.3	1	0.001331
8	dbsnp	rs6991030	C	A	ALLELIC	8/12	4/46	10.3	1	0.001331
8	dbsnp	rs760297	G	A	ALLELIC	8/12	4/46	10.3	1	0.001331
9	dbsnp	rs12348317	A	G	ALLELIC	8/12	4/46	10.3	1	0.001331
9	dbsnp	rs744121	A	G	ALLELIC	8/12	4/46	10.3	1	0.001331
9	dbsnp	rs4877368	A	G	ALLELIC	8/12	4/46	10.3	1	0.001331
11	dbsnp	rs1489196	A	G	ALLELIC	8/12	4/46	10.3	1	0.001331
11	dbsnp	rs12577738	A	G	ALLELIC	8/12	4/46	10.3	1	0.001331
13	dbsnp	rs9550354	G	A	ALLELIC	8/12	4/46	10.3	1	0.001331
15	dbsnp	rs11635037	A	C	ALLELIC	8/12	4/46	10.3	1	0.001331
15	dbsnp	rs7163377	G	A	ALLELIC	8/12	4/46	10.3	1	0.001331
17	dbsnp	rs2079719	A	G	ALLELIC	8/12	4/46	10.3	1	0.001331
20	dbsnp	rs6076864	G	A	ALLELIC	8/12	4/46	10.3	1	0.001331
23	dbsnp	rs5921443	A	C	ALLELIC	7/15	2/46	10.3	1	0.001334
2	dbsnp	rs2075302	A	G	ALLELIC	16/6	16/34	10.26	1	0.001357

2	dbsnp	rs11679244	C	A	ALLELIC	16/6	16/34	10.26	1	0.001357
2	dbsnp	rs1035629	G	A	ALLELIC	16/6	16/34	10.26	1	0.001357
8	dbsnp	rs3824319	G	A	ALLELIC	16/6	16/34	10.26	1	0.001357
12	dbsnp	rs10846411	A	G	ALLELIC	16/6	16/34	10.26	1	0.001357
15	dbsnp	rs7179928	G	A	ALLELIC	16/6	16/34	10.26	1	0.001357
15	dbsnp	rs4238358	A	G	ALLELIC	16/6	16/34	10.26	1	0.001357
23	dbsnp	rs9724106	A	G	ALLELIC	16/6	16/34	10.26	1	0.001357
2	dbsnp	rs10183136	G	A	ALLELIC	15/7	14/36	10.25	1	0.001363
2	dbsnp	rs13401339	A	G	ALLELIC	15/7	14/36	10.25	1	0.001363
4	dbsnp	rs876365	G	A	ALLELIC	15/7	14/36	10.25	1	0.001363
6	dbsnp	rs742556	G	A	ALLELIC	15/7	14/36	10.25	1	0.001363
7	dbsnp	rs10261505	C	A	ALLELIC	15/7	14/36	10.25	1	0.001363
8	dbsnp	rs2970636	A	G	ALLELIC	15/7	14/36	10.25	1	0.001363
8	dbsnp	rs2970627	A	G	ALLELIC	15/7	14/36	10.25	1	0.001363
8	dbsnp	rs3015775	C	A	ALLELIC	15/7	14/36	10.25	1	0.001363
8	dbsnp	rs2040115	G	A	ALLELIC	15/7	14/36	10.25	1	0.001363
8	dbsnp	rs12549902	G	A	ALLELIC	15/7	14/36	10.25	1	0.001363
11	dbsnp	rs7929943	A	C	ALLELIC	15/7	14/36	10.25	1	0.001363
12	dbsnp	rs10746022	A	G	ALLELIC	15/7	14/36	10.25	1	0.001363
12	dbsnp	rs1469662	A	G	ALLELIC	15/7	14/36	10.25	1	0.001363
14	dbsnp	rs12890243	G	A	ALLELIC	15/7	14/36	10.25	1	0.001363
15	dbsnp	rs12901001	A	G	ALLELIC	15/7	14/36	10.25	1	0.001363
17	dbsnp	rs11658574	A	G	ALLELIC	15/7	14/36	10.25	1	0.001363
20	dbsnp	rs2143199	G	A	ALLELIC	15/7	14/36	10.25	1	0.001363
22	dbsnp	rs5752867	A	G	ALLELIC	15/7	14/36	10.25	1	0.001363
3	dbsnp	rs10865884	C	A	ALLELIC	3/19	27/23	10.24	1	0.001374
3	dbsnp	rs17749633	A	G	ALLELIC	3/19	27/23	10.24	1	0.001374
8	dbsnp	rs2272762	A	G	ALLELIC	3/19	27/23	10.24	1	0.001374
8	dbsnp	rs7834641	A	G	ALLELIC	3/19	27/23	10.24	1	0.001374
8	dbsnp	rs2440681	A	G	ALLELIC	3/19	27/23	10.24	1	0.001374
11	dbsnp	rs3108814	A	C	ALLELIC	3/19	27/23	10.24	1	0.001374
16	dbsnp	rs6540277	C	A	ALLELIC	3/19	27/23	10.24	1	0.001374
20	dbsnp	rs6078105	A	G	ALLELIC	3/19	27/23	10.24	1	0.001374
23	dbsnp	rs4828954	G	A	ALLELIC	3/19	27/23	10.24	1	0.001374
23	dbsnp	rs12856122	A	C	ALLELIC	3/19	27/23	10.24	1	0.001374
23	dbsnp	rs1324805	A	G	ALLELIC	3/19	27/23	10.24	1	0.001374
23	dbsnp	rs4893538	C	A	ALLELIC	3/19	27/23	10.24	1	0.001374
23	dbsnp	rs6655225	G	A	ALLELIC	3/19	27/23	10.24	1	0.001374
4	dbsnp	rs6845326	A	G	ALLELIC	1/21	21/29	10.1	1	0.001482
6	dbsnp	rs519167	A	G	ALLELIC	1/21	21/29	10.1	1	0.001482
6	dbsnp	rs3757031	A	G	ALLELIC	1/21	21/29	10.1	1	0.001482
7	dbsnp	rs9690428	G	A	ALLELIC	1/21	21/29	10.1	1	0.001482
19	dbsnp	rs6510125	A	G	ALLELIC	1/21	21/29	10.1	1	0.001482
20	dbsnp	rs6099266	A	G	ALLELIC	1/21	21/29	10.1	1	0.001482
21	dbsnp	rs2837958	A	G	ALLELIC	1/21	21/29	10.1	1	0.001482
23	dbsnp	rs10521931	G	A	ALLELIC	1/21	21/29	10.1	1	0.001482
23	dbsnp	rs4543701	G	A	ALLELIC	1/21	21/29	10.1	1	0.001482
3	dbsnp	rs2062587	G	A	ALLELIC	4/16	31/19	10.08	1	0.001499

3	dbsnp	rs7651477	G	A	ALLELIC	4/16	31/19	10.08	1	0.001499
6	dbsnp	rs1007664	A	G	ALLELIC	4/16	31/19	10.08	1	0.001499
22	dbsnp	rs5756477	G	A	ALLELIC	16/4	19/31	10.08	1	0.001499
1	dbsnp	rs4656671	A	G	ALLELIC	10/10	7/43	10.07	1	0.001508
1	dbsnp	rs12128350	C	A	ALLELIC	10/10	7/43	10.07	1	0.001508
1	dbsnp	rs2801193	A	G	ALLELIC	10/10	7/43	10.07	1	0.001508
2	dbsnp	rs2943634	A	C	ALLELIC	10/10	7/43	10.07	1	0.001508
3	dbsnp	rs9871575	G	A	ALLELIC	10/10	7/43	10.07	1	0.001508
4	dbsnp	rs6821896	A	G	ALLELIC	10/10	7/43	10.07	1	0.001508
5	dbsnp	rs3805651	G	A	ALLELIC	10/10	7/43	10.07	1	0.001508
6	dbsnp	rs1200426	A	C	ALLELIC	10/10	7/43	10.07	1	0.001508
6	dbsnp	rs6938035	A	G	ALLELIC	10/10	7/43	10.07	1	0.001508
7	dbsnp	rs2177558	A	G	ALLELIC	10/10	7/43	10.07	1	0.001508
9	dbsnp	rs10283740	G	A	ALLELIC	10/10	7/43	10.07	1	0.001508
12	dbsnp	rs2520500	G	A	ALLELIC	10/10	7/43	10.07	1	0.001508
12	dbsnp	rs1489900	G	A	ALLELIC	10/10	7/43	10.07	1	0.001508
12	dbsnp	rs7971807	G	A	ALLELIC	10/10	7/43	10.07	1	0.001508
13	dbsnp	rs1928117	G	A	ALLELIC	10/10	7/43	10.07	1	0.001508
18	dbsnp	rs11659315	A	G	ALLELIC	10/10	7/43	10.07	1	0.001508
1	dbsnp	rs11161636	A	G	ALLELIC	2/20	24/26	10.03	1	0.001544
2	dbsnp	rs13016249	G	A	ALLELIC	2/20	24/26	10.03	1	0.001544
2	dbsnp	rs12617678	A	G	ALLELIC	2/20	24/26	10.03	1	0.001544
2	dbsnp	rs13418455	A	G	ALLELIC	2/20	24/26	10.03	1	0.001544
3	dbsnp	rs13075986	C	A	ALLELIC	2/20	24/26	10.03	1	0.001544
6	dbsnp	rs9473663	C	A	ALLELIC	2/20	24/26	10.03	1	0.001544
6	dbsnp	rs505347	A	G	ALLELIC	2/20	24/26	10.03	1	0.001544
6	dbsnp	rs10946177	A	G	ALLELIC	2/20	24/26	10.03	1	0.001544
6	dbsnp	rs6907103	G	A	ALLELIC	2/20	24/26	10.03	1	0.001544
11	dbsnp	rs17661249	G	A	ALLELIC	2/20	24/26	10.03	1	0.001544
11	dbsnp	rs10894728	A	G	ALLELIC	2/20	24/26	10.03	1	0.001544
14	dbsnp	rs6574903	A	G	ALLELIC	2/20	24/26	10.03	1	0.001544
20	dbsnp	rs6040466	A	G	ALLELIC	2/20	24/26	10.03	1	0.001544
20	dbsnp	rs6040469	A	G	ALLELIC	2/20	24/26	10.03	1	0.001544
20	dbsnp	rs6040471	A	G	ALLELIC	2/20	24/26	10.03	1	0.001544
13	dbsnp	rs3764134	C	A	ALLELIC	13/9	10/38	10.01	1	0.001558
21	dbsnp	rs564352	G	A	ALLELIC	13/9	10/38	10.01	1	0.001558
16	dbsnp	rs2745145	A	G	ALLELIC	2/20	23/25	9.905	1	0.001648
1	dbsnp	rs3103780	G	A	ALLELIC	10/12	6/44	9.893	1	0.001659
1	dbsnp	rs396106	G	A	ALLELIC	10/12	6/44	9.893	1	0.001659
1	dbsnp	rs1943448	G	A	ALLELIC	10/12	6/44	9.893	1	0.001659
1	dbsnp	rs6679215	C	A	ALLELIC	10/12	6/44	9.893	1	0.001659
1	dbsnp	rs12733626	A	C	ALLELIC	10/12	6/44	9.893	1	0.001659
1	dbsnp	rs2797239	A	G	ALLELIC	10/12	6/44	9.893	1	0.001659
1	dbsnp	rs2797246	A	G	ALLELIC	10/12	6/44	9.893	1	0.001659
2	dbsnp	rs4577244	A	G	ALLELIC	10/12	6/44	9.893	1	0.001659
2	dbsnp	rs11688605	G	A	ALLELIC	10/12	6/44	9.893	1	0.001659
2	dbsnp	rs11680458	A	C	ALLELIC	10/12	6/44	9.893	1	0.001659
2	dbsnp	rs2677510	A	G	ALLELIC	10/12	6/44	9.893	1	0.001659

2	dbsnp	rs2894604	A	G	ALLELIC	10/12	6/44	9.893	1	0.001659
4	dbsnp	rs1982349	G	A	ALLELIC	10/12	6/44	9.893	1	0.001659
4	dbsnp	rs3822262	C	A	ALLELIC	10/12	6/44	9.893	1	0.001659
4	dbsnp	rs295238	G	A	ALLELIC	10/12	6/44	9.893	1	0.001659
6	dbsnp	rs7767977	G	A	ALLELIC	10/12	6/44	9.893	1	0.001659
6	dbsnp	rs17693403	A	G	ALLELIC	10/12	6/44	9.893	1	0.001659
7	dbsnp	rs17207352	A	G	ALLELIC	10/12	6/44	9.893	1	0.001659
7	dbsnp	rs3778850	A	G	ALLELIC	10/12	6/44	9.893	1	0.001659
9	dbsnp	rs2761134	A	G	ALLELIC	10/12	6/44	9.893	1	0.001659
9	dbsnp	rs10735599	G	A	ALLELIC	10/12	6/44	9.893	1	0.001659
9	dbsnp	rs11142498	A	G	ALLELIC	10/12	6/44	9.893	1	0.001659
9	dbsnp	rs4744607	G	A	ALLELIC	10/12	6/44	9.893	1	0.001659
11	dbsnp	rs2307073	G	A	ALLELIC	10/12	6/44	9.893	1	0.001659
11	dbsnp	rs7121190	G	A	ALLELIC	10/12	6/44	9.893	1	0.001659
12	dbsnp	rs4529948	A	C	ALLELIC	10/12	6/44	9.893	1	0.001659
12	dbsnp	rs11171821	A	G	ALLELIC	10/12	6/44	9.893	1	0.001659
13	dbsnp	rs2162250	G	A	ALLELIC	10/12	6/44	9.893	1	0.001659
13	dbsnp	rs2243409	A	G	ALLELIC	10/12	6/44	9.893	1	0.001659
13	dbsnp	rs7321084	A	G	ALLELIC	10/12	6/44	9.893	1	0.001659
13	dbsnp	rs2391655	C	A	ALLELIC	10/12	6/44	9.893	1	0.001659
14	dbsnp	rs1950214	A	C	ALLELIC	10/12	6/44	9.893	1	0.001659
16	dbsnp	rs1874008	A	G	ALLELIC	10/12	6/44	9.893	1	0.001659
18	dbsnp	rs12456875	A	G	ALLELIC	10/12	6/44	9.893	1	0.001659
18	dbsnp	rs4517886	A	C	ALLELIC	10/12	6/44	9.893	1	0.001659
18	dbsnp	rs654466	G	A	ALLELIC	10/12	6/44	9.893	1	0.001659
18	dbsnp	rs2957134	C	A	ALLELIC	10/12	6/44	9.893	1	0.001659
20	dbsnp	rs4507151	A	C	ALLELIC	10/12	6/44	9.893	1	0.001659
23	dbsnp	rs658454	G	A	ALLELIC	10/12	6/44	9.893	1	0.001659
23	dbsnp	rs12392496	C	A	ALLELIC	10/12	6/44	9.893	1	0.001659
1	dbsnp	rs4648873	G	A	ALLELIC	12/10	9/41	9.876	1	0.001674
1	dbsnp	rs1320967	G	A	ALLELIC	12/10	9/41	9.876	1	0.001674
1	dbsnp	rs4656656	A	C	ALLELIC	12/10	9/41	9.876	1	0.001674
1	dbsnp	rs10912463	A	G	ALLELIC	12/10	9/41	9.876	1	0.001674
2	dbsnp	rs280210	A	G	ALLELIC	12/10	9/41	9.876	1	0.001674
2	dbsnp	rs775806	A	G	ALLELIC	12/10	9/41	9.876	1	0.001674
2	dbsnp	rs280213	A	C	ALLELIC	12/10	9/41	9.876	1	0.001674
2	dbsnp	rs2972143	A	G	ALLELIC	12/10	9/41	9.876	1	0.001674
4	dbsnp	rs12510706	A	G	ALLELIC	12/10	9/41	9.876	1	0.001674
4	dbsnp	rs2389089	A	G	ALLELIC	12/10	9/41	9.876	1	0.001674
4	dbsnp	rs10516621	G	A	ALLELIC	12/10	9/41	9.876	1	0.001674
4	dbsnp	rs13106872	G	A	ALLELIC	12/10	9/41	9.876	1	0.001674
4	dbsnp	rs1549805	C	A	ALLELIC	12/10	9/41	9.876	1	0.001674
4	dbsnp	rs13151706	G	A	ALLELIC	12/10	9/41	9.876	1	0.001674
4	dbsnp	rs1821011	G	A	ALLELIC	12/10	9/41	9.876	1	0.001674
5	dbsnp	rs3094352	A	G	ALLELIC	12/10	9/41	9.876	1	0.001674
5	dbsnp	rs2936940	A	G	ALLELIC	12/10	9/41	9.876	1	0.001674
6	dbsnp	rs12211463	C	A	ALLELIC	12/10	9/41	9.876	1	0.001674
7	dbsnp	rs4729736	G	A	ALLELIC	12/10	9/41	9.876	1	0.001674

8	dbsnp	rs13250356	A	C	ALLELIC	12/10	9/41	9.876	1	0.001674
8	dbsnp	rs11135740	A	G	ALLELIC	12/10	9/41	9.876	1	0.001674
8	dbsnp	rs7826337	A	G	ALLELIC	12/10	9/41	9.876	1	0.001674
8	dbsnp	rs13280665	G	A	ALLELIC	12/10	9/41	9.876	1	0.001674
9	dbsnp	rs611698	G	A	ALLELIC	12/10	9/41	9.876	1	0.001674
9	dbsnp	rs10978080	A	G	ALLELIC	12/10	9/41	9.876	1	0.001674
9	dbsnp	rs11790833	A	G	ALLELIC	12/10	9/41	9.876	1	0.001674
10	dbsnp	rs2458705	A	G	ALLELIC	12/10	9/41	9.876	1	0.001674
10	dbsnp	rs11511761	A	G	ALLELIC	12/10	9/41	9.876	1	0.001674
12	dbsnp	rs2125716	A	G	ALLELIC	12/10	9/41	9.876	1	0.001674
13	dbsnp	rs2408213	G	A	ALLELIC	12/10	9/41	9.876	1	0.001674
13	dbsnp	rs9536383	A	G	ALLELIC	12/10	9/41	9.876	1	0.001674
14	dbsnp	rs8005039	G	A	ALLELIC	12/10	9/41	9.876	1	0.001674
14	dbsnp	rs1242095	C	A	ALLELIC	12/10	9/41	9.876	1	0.001674
15	dbsnp	rs6496200	A	G	ALLELIC	12/10	9/41	9.876	1	0.001674
20	dbsnp	rs11086562	G	A	ALLELIC	12/10	9/41	9.876	1	0.001674
23	dbsnp	rs5921281	G	A	ALLELIC	12/10	9/41	9.876	1	0.001674
1	dbsnp	rs2798111	G	A	ALLELIC	1/19	22/28	9.849	1	0.001699
1	dbsnp	rs6658433	A	G	ALLELIC	1/19	22/28	9.849	1	0.001699
22	dbsnp	rs713938	G	A	ALLELIC	1/19	22/28	9.849	1	0.001699
23	dbsnp	rs6639663	A	G	ALLELIC	18/4	19/27	9.848	1	0.0017
4	dbsnp	rs11721925	G	A	ALLELIC	7/13	3/47	9.812	1	0.001734
4	dbsnp	rs17022918	G	A	ALLELIC	7/13	3/47	9.812	1	0.001734
4	dbsnp	rs6827776	A	G	ALLELIC	7/13	3/47	9.812	1	0.001734
6	dbsnp	rs12193553	A	C	ALLELIC	7/13	3/47	9.812	1	0.001734
6	dbsnp	rs6928430	A	G	ALLELIC	7/13	3/47	9.812	1	0.001734
7	dbsnp	rs1523641	G	A	ALLELIC	7/13	3/47	9.812	1	0.001734
9	dbsnp	rs11999442	A	G	ALLELIC	7/13	3/47	9.812	1	0.001734
11	dbsnp	rs11037217	A	G	ALLELIC	7/13	3/47	9.812	1	0.001734
11	dbsnp	rs371614	A	G	ALLELIC	7/13	3/47	9.812	1	0.001734
12	dbsnp	rs12371967	G	A	ALLELIC	7/13	3/47	9.812	1	0.001734
13	dbsnp	rs17078882	A	G	ALLELIC	7/13	3/47	9.812	1	0.001734
13	dbsnp	rs4772455	G	A	ALLELIC	7/13	3/47	9.812	1	0.001734
23	dbsnp	rs5979984	G	A	ALLELIC	7/13	3/47	9.812	1	0.001734
23	dbsnp	rs5990853	A	G	ALLELIC	7/13	3/47	9.812	1	0.001734
21	dbsnp	rs4816274	A	C	ALLELIC	4/18	28/20	9.8	1	0.001745
23	dbsnp	rs17282591	A	G	ALLELIC	4/18	28/20	9.8	1	0.001745
1	dbsnp	rs16837408	A	G	ALLELIC	0/22	17/33	9.792	1	0.001753
2	dbsnp	rs2631976	A	G	ALLELIC	0/22	17/33	9.792	1	0.001753
2	dbsnp	rs2541169	G	A	ALLELIC	0/22	17/33	9.792	1	0.001753
4	dbsnp	rs10857310	G	A	ALLELIC	0/22	17/33	9.792	1	0.001753
15	dbsnp	rs1470678	G	A	ALLELIC	0/22	17/33	9.792	1	0.001753
23	dbsnp	rs5950318	A	G	ALLELIC	0/22	17/33	9.792	1	0.001753
23	dbsnp	rs2035111	G	A	ALLELIC	0/22	17/33	9.792	1	0.001753
8	dbsnp	rs12678092	A	G	ALLELIC	7/15	2/44	9.78	1	0.001765
1	dbsnp	rs2274316	C	A	ALLELIC	4/18	29/21	9.757	1	0.001787
1	dbsnp	rs1925950	G	A	ALLELIC	4/18	29/21	9.757	1	0.001787
3	dbsnp	rs2581623	G	A	ALLELIC	4/18	29/21	9.757	1	0.001787

8	dbsnp	rs6991079	A	C	ALLELIC	4/18	29/21	9.757	1	0.001787
23	dbsnp	rs1493058	G	A	ALLELIC	4/18	29/21	9.757	1	0.001787
23	dbsnp	rs6634674	G	A	ALLELIC	4/18	29/21	9.757	1	0.001787
13	dbsnp	rs9598786	A	G	ALLELIC	3/17	27/21	9.744	1	0.001799
3	dbsnp	rs2162683	A	G	ALLELIC	3/17	28/22	9.733	1	0.00181
4	dbsnp	rs1328925	G	A	ALLELIC	3/17	28/22	9.733	1	0.00181
14	dbsnp	rs4981135	G	A	ALLELIC	3/17	28/22	9.733	1	0.00181
12	dbsnp	rs4759303	G	A	ALLELIC	0/20	18/32	9.692	1	0.00185
23	dbsnp	rs2362161	A	G	ALLELIC	0/20	18/32	9.692	1	0.00185
14	dbsnp	rs10138062	A	G	ALLELIC	15/5	17/33	9.677	1	0.001866
15	dbsnp	rs4779843	C	A	ALLELIC	15/5	17/33	9.677	1	0.001866
15	dbsnp	rs4886467	A	C	ALLELIC	15/5	17/33	9.677	1	0.001866
17	dbsnp	rs271650	A	G	ALLELIC	15/5	17/33	9.677	1	0.001866
23	dbsnp	rs6628760	A	G	ALLELIC	15/5	17/33	9.677	1	0.001866
2	dbsnp	rs10169170	G	A	ALLELIC	2/18	25/25	9.647	1	0.001897
3	dbsnp	rs4685723	A	G	ALLELIC	2/18	25/25	9.647	1	0.001897
1	dbsnp	rs7540400	A	G	ALLELIC	5/15	1/49	9.643	1	0.001901
4	dbsnp	rs4452425	C	A	ALLELIC	5/15	1/49	9.643	1	0.001901
4	dbsnp	rs10003327	C	A	ALLELIC	5/15	1/49	9.643	1	0.001901
7	dbsnp	rs6959619	G	A	ALLELIC	5/15	1/49	9.643	1	0.001901
8	dbsnp	rs2338958	A	G	ALLELIC	5/15	1/49	9.643	1	0.001901
12	dbsnp	rs11104814	A	G	ALLELIC	5/15	1/49	9.643	1	0.001901
14	dbsnp	rs10149959	G	A	ALLELIC	5/15	1/49	9.643	1	0.001901
23	dbsnp	rs12011358	G	A	ALLELIC	5/15	1/49	9.643	1	0.001901
23	dbsnp	rs5990897	G	A	ALLELIC	5/15	1/49	9.643	1	0.001901
23	dbsnp	rs6653777	G	A	ALLELIC	5/15	1/49	9.643	1	0.001901
1	dbsnp	rs6692242	A	G	ALLELIC	4/18	0/50	9.626	1	0.001919
1	dbsnp	rs4660838	G	A	ALLELIC	4/18	0/50	9.626	1	0.001919
1	dbsnp	rs17192059	G	A	ALLELIC	4/18	0/50	9.626	1	0.001919
1	dbsnp	rs12094184	A	C	ALLELIC	4/18	0/50	9.626	1	0.001919
1	dbsnp	rs1332791	A	G	ALLELIC	4/18	0/50	9.626	1	0.001919
1	dbsnp	rs12077203	A	G	ALLELIC	4/18	0/50	9.626	1	0.001919
1	dbsnp	rs2802984	A	G	ALLELIC	4/18	0/50	9.626	1	0.001919
1	dbsnp	rs7542764	A	G	ALLELIC	4/18	0/50	9.626	1	0.001919
1	dbsnp	rs12097629	A	G	ALLELIC	4/18	0/50	9.626	1	0.001919
2	dbsnp	rs2373530	A	G	ALLELIC	4/18	0/50	9.626	1	0.001919
2	dbsnp	rs11674390	G	A	ALLELIC	4/18	0/50	9.626	1	0.001919
2	dbsnp	rs10204882	G	A	ALLELIC	4/18	0/50	9.626	1	0.001919
2	dbsnp	rs12991146	A	C	ALLELIC	4/18	0/50	9.626	1	0.001919
2	dbsnp	rs6728812	A	G	ALLELIC	4/18	0/50	9.626	1	0.001919
2	dbsnp	rs17556229	A	G	ALLELIC	4/18	0/50	9.626	1	0.001919
2	dbsnp	rs13426918	G	A	ALLELIC	4/18	0/50	9.626	1	0.001919
2	dbsnp	rs10206655	A	C	ALLELIC	4/18	0/50	9.626	1	0.001919
2	dbsnp	rs1837146	A	G	ALLELIC	4/18	0/50	9.626	1	0.001919
2	dbsnp	rs2368388	A	G	ALLELIC	4/18	0/50	9.626	1	0.001919
2	dbsnp	rs17830265	A	G	ALLELIC	4/18	0/50	9.626	1	0.001919
2	dbsnp	rs16850199	G	A	ALLELIC	4/18	0/50	9.626	1	0.001919
2	dbsnp	rs11902313	C	A	ALLELIC	4/18	0/50	9.626	1	0.001919

2	dbsnp	rs10180563	A	C	ALLELIC	4/18	0/50	9.626	1	0.001919
2	dbsnp	rs10169266	G	A	ALLELIC	4/18	0/50	9.626	1	0.001919
2	dbsnp	rs7593557	A	G	ALLELIC	4/18	0/50	9.626	1	0.001919
3	dbsnp	rs7645841	A	C	ALLELIC	4/18	0/50	9.626	1	0.001919
3	dbsnp	rs4257505	A	G	ALLELIC	4/18	0/50	9.626	1	0.001919
4	dbsnp	rs886366	G	A	ALLELIC	4/18	0/50	9.626	1	0.001919
4	dbsnp	rs11721958	G	A	ALLELIC	4/18	0/50	9.626	1	0.001919
4	dbsnp	rs6838195	A	G	ALLELIC	4/18	0/50	9.626	1	0.001919
4	dbsnp	rs1370300	A	G	ALLELIC	4/18	0/50	9.626	1	0.001919
4	dbsnp	rs186080	G	A	ALLELIC	4/18	0/50	9.626	1	0.001919
4	dbsnp	rs6553315	A	G	ALLELIC	4/18	0/50	9.626	1	0.001919
5	dbsnp	rs6860611	C	A	ALLELIC	4/18	0/50	9.626	1	0.001919
5	dbsnp	rs1036599	A	C	ALLELIC	4/18	0/50	9.626	1	0.001919
5	dbsnp	rs17104268	C	A	ALLELIC	4/18	0/50	9.626	1	0.001919
5	dbsnp	rs17104331	G	A	ALLELIC	4/18	0/50	9.626	1	0.001919
6	dbsnp	rs794785	A	G	ALLELIC	4/18	0/50	9.626	1	0.001919
6	dbsnp	rs707831	A	G	ALLELIC	4/18	0/50	9.626	1	0.001919
6	dbsnp	rs707836	G	A	ALLELIC	4/18	0/50	9.626	1	0.001919
6	dbsnp	rs9462540	A	G	ALLELIC	4/18	0/50	9.626	1	0.001919
6	dbsnp	rs9394616	A	G	ALLELIC	4/18	0/50	9.626	1	0.001919
6	dbsnp	rs12175102	G	A	ALLELIC	4/18	0/50	9.626	1	0.001919
6	dbsnp	rs9394619	G	A	ALLELIC	4/18	0/50	9.626	1	0.001919
6	dbsnp	rs2917887	A	C	ALLELIC	4/18	0/50	9.626	1	0.001919
6	dbsnp	rs3012519	A	G	ALLELIC	4/18	0/50	9.626	1	0.001919
6	dbsnp	rs1437579	G	A	ALLELIC	4/18	0/50	9.626	1	0.001919
6	dbsnp	rs7758873	A	C	ALLELIC	4/18	0/50	9.626	1	0.001919
6	dbsnp	rs16877616	G	A	ALLELIC	4/18	0/50	9.626	1	0.001919
6	dbsnp	rs592805	G	A	ALLELIC	4/18	0/50	9.626	1	0.001919
6	dbsnp	rs1066335	G	A	ALLELIC	4/18	0/50	9.626	1	0.001919
6	dbsnp	rs494991	A	G	ALLELIC	4/18	0/50	9.626	1	0.001919
6	dbsnp	rs9918469	A	G	ALLELIC	4/18	0/50	9.626	1	0.001919
6	dbsnp	rs9401149	A	C	ALLELIC	4/18	0/50	9.626	1	0.001919
6	dbsnp	rs259390	C	A	ALLELIC	4/18	0/50	9.626	1	0.001919
6	dbsnp	rs6915111	A	G	ALLELIC	4/18	0/50	9.626	1	0.001919
6	dbsnp	rs7766568	A	G	ALLELIC	4/18	0/50	9.626	1	0.001919
7	dbsnp	rs17169573	A	C	ALLELIC	4/18	0/50	9.626	1	0.001919
7	dbsnp	rs12699826	G	A	ALLELIC	4/18	0/50	9.626	1	0.001919
7	dbsnp	rs16479	G	A	ALLELIC	4/18	0/50	9.626	1	0.001919
7	dbsnp	rs16139	G	A	ALLELIC	4/18	0/50	9.626	1	0.001919
7	dbsnp	rs10230036	G	A	ALLELIC	4/18	0/50	9.626	1	0.001919
7	dbsnp	rs17149199	G	A	ALLELIC	4/18	0/50	9.626	1	0.001919
7	dbsnp	rs12540545	G	A	ALLELIC	4/18	0/50	9.626	1	0.001919
7	dbsnp	rs17150293	A	G	ALLELIC	4/18	0/50	9.626	1	0.001919
7	dbsnp	rs16871759	A	G	ALLELIC	4/18	0/50	9.626	1	0.001919
8	dbsnp	rs7841466	A	G	ALLELIC	4/18	0/50	9.626	1	0.001919
8	dbsnp	rs11166852	A	G	ALLELIC	4/18	0/50	9.626	1	0.001919
9	dbsnp	rs12554573	G	A	ALLELIC	4/18	0/50	9.626	1	0.001919
9	dbsnp	rs16920640	A	G	ALLELIC	4/18	0/50	9.626	1	0.001919

9	dbsnp	rs7469637	A	C	ALLELIC	4/18	0/50	9.626	1	0.001919
10	dbsnp	rs870376	G	A	ALLELIC	4/18	0/50	9.626	1	0.001919
10	dbsnp	rs16927796	A	G	ALLELIC	4/18	0/50	9.626	1	0.001919
10	dbsnp	rs17153622	A	G	ALLELIC	4/18	0/50	9.626	1	0.001919
10	dbsnp	rs3750776	G	A	ALLELIC	4/18	0/50	9.626	1	0.001919
10	dbsnp	rs11190421	A	G	ALLELIC	4/18	0/50	9.626	1	0.001919
10	dbsnp	rs12247992	A	G	ALLELIC	4/18	0/50	9.626	1	0.001919
10	dbsnp	rs2270962	A	G	ALLELIC	4/18	0/50	9.626	1	0.001919
10	dbsnp	rs7924284	A	G	ALLELIC	4/18	0/50	9.626	1	0.001919
10	dbsnp	rs12241379	A	G	ALLELIC	4/18	0/50	9.626	1	0.001919
10	dbsnp	rs12218543	A	G	ALLELIC	4/18	0/50	9.626	1	0.001919
10	dbsnp	rs10885917	G	A	ALLELIC	4/18	0/50	9.626	1	0.001919
10	dbsnp	rs11817421	A	C	ALLELIC	4/18	0/50	9.626	1	0.001919
10	dbsnp	rs11197640	G	A	ALLELIC	4/18	0/50	9.626	1	0.001919
11	dbsnp	rs3812774	A	G	ALLELIC	4/18	0/50	9.626	1	0.001919
11	dbsnp	rs232253	G	A	ALLELIC	4/18	0/50	9.626	1	0.001919
11	dbsnp	rs11600671	A	G	ALLELIC	4/18	0/50	9.626	1	0.001919
11	dbsnp	rs12418439	A	G	ALLELIC	4/18	0/50	9.626	1	0.001919
11	dbsnp	rs11222684	A	G	ALLELIC	4/18	0/50	9.626	1	0.001919
11	dbsnp	rs1543121	A	C	ALLELIC	4/18	0/50	9.626	1	0.001919
11	dbsnp	rs12803146	A	G	ALLELIC	4/18	0/50	9.626	1	0.001919
12	dbsnp	rs2071064	A	G	ALLELIC	4/18	0/50	9.626	1	0.001919
12	dbsnp	rs17022336	G	A	ALLELIC	4/18	0/50	9.626	1	0.001919
12	dbsnp	rs17302157	A	C	ALLELIC	4/18	0/50	9.626	1	0.001919
12	dbsnp	rs9919733	G	A	ALLELIC	4/18	0/50	9.626	1	0.001919
12	dbsnp	rs7965399	G	A	ALLELIC	4/18	0/50	9.626	1	0.001919
12	dbsnp	rs17035610	A	G	ALLELIC	4/18	0/50	9.626	1	0.001919
12	dbsnp	rs12296651	G	A	ALLELIC	4/18	0/50	9.626	1	0.001919
12	dbsnp	rs7305410	A	G	ALLELIC	4/18	0/50	9.626	1	0.001919
12	dbsnp	rs2001789	A	G	ALLELIC	4/18	0/50	9.626	1	0.001919
13	dbsnp	rs9582652	G	A	ALLELIC	4/18	0/50	9.626	1	0.001919
13	dbsnp	rs9582653	A	C	ALLELIC	4/18	0/50	9.626	1	0.001919
14	dbsnp	rs1997903	A	G	ALLELIC	4/18	0/50	9.626	1	0.001919
14	dbsnp	rs11627861	A	G	ALLELIC	4/18	0/50	9.626	1	0.001919
14	dbsnp	rs17108244	G	A	ALLELIC	4/18	0/50	9.626	1	0.001919
15	dbsnp	rs11732	G	A	ALLELIC	4/18	0/50	9.626	1	0.001919
15	dbsnp	rs8027135	G	A	ALLELIC	4/18	0/50	9.626	1	0.001919
15	dbsnp	rs10518810	A	G	ALLELIC	4/18	0/50	9.626	1	0.001919
15	dbsnp	rs16953240	A	C	ALLELIC	4/18	0/50	9.626	1	0.001919
15	dbsnp	rs7183937	A	G	ALLELIC	4/18	0/50	9.626	1	0.001919
15	dbsnp	rs7181999	G	A	ALLELIC	4/18	0/50	9.626	1	0.001919
15	dbsnp	rs2414418	G	A	ALLELIC	4/18	0/50	9.626	1	0.001919
15	dbsnp	rs7168305	A	G	ALLELIC	4/18	0/50	9.626	1	0.001919
16	dbsnp	rs35262813	A	G	ALLELIC	4/18	0/50	9.626	1	0.001919
16	dbsnp	rs7188098	A	G	ALLELIC	4/18	0/50	9.626	1	0.001919
17	dbsnp	rs1017346	G	A	ALLELIC	4/18	0/50	9.626	1	0.001919
17	dbsnp	rs205023	G	A	ALLELIC	4/18	0/50	9.626	1	0.001919
17	dbsnp	rs9911317	A	G	ALLELIC	4/18	0/50	9.626	1	0.001919

17	dbsnp	rs16966910	G	A	ALLELIC	4/18	0/50	9.626	1	0.001919
17	dbsnp	rs1553469	A	C	ALLELIC	4/18	0/50	9.626	1	0.001919
17	dbsnp	rs9910698	A	G	ALLELIC	4/18	0/50	9.626	1	0.001919
17	dbsnp	rs9892195	C	A	ALLELIC	4/18	0/50	9.626	1	0.001919
18	dbsnp	rs8087427	A	G	ALLELIC	4/18	0/50	9.626	1	0.001919
18	dbsnp	rs1261117	G	A	ALLELIC	4/18	0/50	9.626	1	0.001919
18	dbsnp	rs1484736	A	G	ALLELIC	4/18	0/50	9.626	1	0.001919
18	dbsnp	rs17088367	A	G	ALLELIC	4/18	0/50	9.626	1	0.001919
19	dbsnp	rs11879985	A	G	ALLELIC	4/18	0/50	9.626	1	0.001919
20	dbsnp	rs6047045	A	C	ALLELIC	4/18	0/50	9.626	1	0.001919
20	dbsnp	rs6047076	A	C	ALLELIC	4/18	0/50	9.626	1	0.001919
20	dbsnp	rs5743509	A	G	ALLELIC	4/18	0/50	9.626	1	0.001919
20	dbsnp	rs6122279	A	G	ALLELIC	4/18	0/50	9.626	1	0.001919
21	dbsnp	rs2823739	G	A	ALLELIC	4/18	0/50	9.626	1	0.001919
21	dbsnp	rs13050646	A	C	ALLELIC	4/18	0/50	9.626	1	0.001919
22	dbsnp	rs132550	A	G	ALLELIC	4/18	0/50	9.626	1	0.001919
22	dbsnp	rs5762916	A	G	ALLELIC	4/18	0/50	9.626	1	0.001919
22	dbsnp	rs11089668	G	A	ALLELIC	4/18	0/50	9.626	1	0.001919
22	dbsnp	rs4393836	A	G	ALLELIC	4/18	0/50	9.626	1	0.001919
22	dbsnp	rs5758976	G	A	ALLELIC	4/18	0/50	9.626	1	0.001919
22	dbsnp	rs753086	A	G	ALLELIC	4/18	0/50	9.626	1	0.001919
22	dbsnp	rs2267465	A	G	ALLELIC	4/18	0/50	9.626	1	0.001919
22	dbsnp	rs11090909	A	C	ALLELIC	4/18	0/50	9.626	1	0.001919
22	dbsnp	rs11090911	A	G	ALLELIC	4/18	0/50	9.626	1	0.001919
23	dbsnp	rs1989813	A	G	ALLELIC	4/18	0/50	9.626	1	0.001919
23	dbsnp	rs7062942	G	A	ALLELIC	4/18	0/50	9.626	1	0.001919
23	dbsnp	rs5990961	A	G	ALLELIC	4/18	0/50	9.626	1	0.001919
23	dbsnp	rs5990842	A	C	ALLELIC	4/18	0/50	9.626	1	0.001919
23	dbsnp	rs2057781	A	G	ALLELIC	4/18	0/50	9.626	1	0.001919
23	dbsnp	rs1882402	G	A	ALLELIC	4/18	0/50	9.626	1	0.001919
23	dbsnp	rs7065988	C	A	ALLELIC	4/18	0/50	9.626	1	0.001919
23	dbsnp	rs5971431	G	A	ALLELIC	4/18	0/50	9.626	1	0.001919
23	dbsnp	rs6628307	A	G	ALLELIC	4/18	0/50	9.626	1	0.001919
23	dbsnp	rs1736868	G	A	ALLELIC	4/18	0/50	9.626	1	0.001919
23	dbsnp	rs2157722	A	G	ALLELIC	4/18	0/50	9.626	1	0.001919
23	dbsnp	rs17266807	A	G	ALLELIC	4/18	0/50	9.626	1	0.001919
23	dbsnp	rs7066136	G	A	ALLELIC	4/18	0/50	9.626	1	0.001919
23	dbsnp	rs2286479	A	G	ALLELIC	4/18	0/50	9.626	1	0.001919
23	dbsnp	rs6418831	A	G	ALLELIC	4/18	0/50	9.626	1	0.001919
23	dbsnp	rs10521851	G	A	ALLELIC	4/18	0/50	9.626	1	0.001919
23	dbsnp	rs2224145	A	G	ALLELIC	4/18	0/50	9.626	1	0.001919
23	dbsnp	rs36029735	A	C	ALLELIC	4/18	0/50	9.626	1	0.001919
23	dbsnp	rs17253613	A	G	ALLELIC	4/18	0/50	9.626	1	0.001919
23	dbsnp	rs12010819	A	G	ALLELIC	4/18	0/50	9.626	1	0.001919
23	dbsnp	rs12012815	A	G	ALLELIC	4/18	0/50	9.626	1	0.001919
23	dbsnp	rs17253640	G	A	ALLELIC	4/18	0/50	9.626	1	0.001919
1	dbsnp	rs4659120	A	G	ALLELIC	11/9	9/41	9.583	1	0.001964
1	dbsnp	rs12744341	C	A	ALLELIC	11/9	9/41	9.583	1	0.001964

2	dbsnp	rs2713539	A	G	ALLELIC	11/9	9/41	9.583	1	0.001964
3	dbsnp	rs4270456	G	A	ALLELIC	11/9	9/41	9.583	1	0.001964
5	dbsnp	rs4701486	C	A	ALLELIC	11/9	9/41	9.583	1	0.001964
5	dbsnp	rs7709145	G	A	ALLELIC	11/9	9/41	9.583	1	0.001964
9	dbsnp	rs4574938	A	G	ALLELIC	11/9	9/41	9.583	1	0.001964
12	dbsnp	rs2048499	A	G	ALLELIC	11/9	9/41	9.583	1	0.001964
12	dbsnp	rs11059422	A	G	ALLELIC	11/9	9/41	9.583	1	0.001964
14	dbsnp	rs10150720	A	G	ALLELIC	11/9	9/41	9.583	1	0.001964
14	dbsnp	rs1953236	A	G	ALLELIC	11/9	9/41	9.583	1	0.001964
18	dbsnp	rs2322095	C	A	ALLELIC	11/9	9/41	9.583	1	0.001964
23	dbsnp	rs13328614	C	A	ALLELIC	11/9	9/41	9.583	1	0.001964
11	dbsnp	rs4329682	A	G	ALLELIC	5/17	30/18	9.545	1	0.002004
23	dbsnp	rs524376	A	G	ALLELIC	5/17	30/18	9.545	1	0.002004
1	dbsnp	rs11165867	A	G	ALLELIC	6/14	2/48	9.54	1	0.00201
2	dbsnp	rs17021075	C	A	ALLELIC	6/14	2/48	9.54	1	0.00201
2	dbsnp	rs7607734	G	A	ALLELIC	6/14	2/48	9.54	1	0.00201
2	dbsnp	rs2304700	A	C	ALLELIC	6/14	2/48	9.54	1	0.00201
2	dbsnp	rs2568675	G	A	ALLELIC	6/14	2/48	9.54	1	0.00201
4	dbsnp	rs7683391	G	A	ALLELIC	6/14	2/48	9.54	1	0.00201
4	dbsnp	rs7677050	A	G	ALLELIC	6/14	2/48	9.54	1	0.00201
5	dbsnp	rs40627	G	A	ALLELIC	6/14	2/48	9.54	1	0.00201
6	dbsnp	rs9482329	A	G	ALLELIC	6/14	2/48	9.54	1	0.00201
6	dbsnp	rs4618543	G	A	ALLELIC	6/14	2/48	9.54	1	0.00201
7	dbsnp	rs2299395	G	A	ALLELIC	6/14	2/48	9.54	1	0.00201
8	dbsnp	rs12676497	A	C	ALLELIC	6/14	2/48	9.54	1	0.00201
8	dbsnp	rs7838257	A	G	ALLELIC	6/14	2/48	9.54	1	0.00201
10	dbsnp	rs17701618	G	A	ALLELIC	6/14	2/48	9.54	1	0.00201
11	dbsnp	rs4391777	A	C	ALLELIC	6/14	2/48	9.54	1	0.00201
12	dbsnp	rs11831905	A	G	ALLELIC	6/14	2/48	9.54	1	0.00201
14	dbsnp	rs2293729	G	A	ALLELIC	6/14	2/48	9.54	1	0.00201
15	dbsnp	rs16976343	G	A	ALLELIC	6/14	2/48	9.54	1	0.00201
16	dbsnp	rs7193224	A	G	ALLELIC	6/14	2/48	9.54	1	0.00201
23	dbsnp	rs4827171	C	A	ALLELIC	6/14	2/48	9.54	1	0.00201
23	dbsnp	rs5908557	A	C	ALLELIC	6/14	2/48	9.54	1	0.00201
20	dbsnp	rs11086906	G	A	ALLELIC	5/11	2/48	9.493	1	0.002062
4	dbsnp	rs7678151	A	G	ALLELIC	15/7	14/34	9.463	1	0.002096
1	dbsnp	rs2815274	G	A	ALLELIC	13/9	11/39	9.458	1	0.002102
1	dbsnp	rs815342	G	A	ALLELIC	13/9	11/39	9.458	1	0.002102
1	dbsnp	rs2990998	A	G	ALLELIC	13/9	11/39	9.458	1	0.002102
2	dbsnp	rs10929383	A	G	ALLELIC	13/9	11/39	9.458	1	0.002102
2	dbsnp	rs4952738	A	G	ALLELIC	13/9	11/39	9.458	1	0.002102
2	dbsnp	rs13007371	A	G	ALLELIC	13/9	11/39	9.458	1	0.002102
2	dbsnp	rs4662269	C	A	ALLELIC	13/9	11/39	9.458	1	0.002102
4	dbsnp	rs278927	G	A	ALLELIC	13/9	11/39	9.458	1	0.002102
4	dbsnp	rs17442189	G	A	ALLELIC	13/9	11/39	9.458	1	0.002102
4	dbsnp	rs4699293	A	G	ALLELIC	13/9	11/39	9.458	1	0.002102
5	dbsnp	rs727432	A	C	ALLELIC	13/9	11/39	9.458	1	0.002102
5	dbsnp	rs10054679	A	C	ALLELIC	13/9	11/39	9.458	1	0.002102

6	dbsnp	rs9449760	A	G	ALLELIC	13/9	11/39	9.458	1	0.002102
6	dbsnp	rs2480191	G	A	ALLELIC	13/9	11/39	9.458	1	0.002102
6	dbsnp	rs9480206	A	C	ALLELIC	13/9	11/39	9.458	1	0.002102
7	dbsnp	rs6965444	A	G	ALLELIC	13/9	11/39	9.458	1	0.002102
10	dbsnp	rs11251294	A	G	ALLELIC	13/9	11/39	9.458	1	0.002102
10	dbsnp	rs1227783	A	G	ALLELIC	13/9	11/39	9.458	1	0.002102
10	dbsnp	rs11195620	G	A	ALLELIC	13/9	11/39	9.458	1	0.002102
11	dbsnp	rs11031509	A	G	ALLELIC	13/9	11/39	9.458	1	0.002102
12	dbsnp	rs7972834	C	A	ALLELIC	13/9	11/39	9.458	1	0.002102
12	dbsnp	rs7139244	G	A	ALLELIC	13/9	11/39	9.458	1	0.002102
12	dbsnp	rs2417821	G	A	ALLELIC	13/9	11/39	9.458	1	0.002102
13	dbsnp	rs663497	A	G	ALLELIC	13/9	11/39	9.458	1	0.002102
13	dbsnp	rs7334529	G	A	ALLELIC	13/9	11/39	9.458	1	0.002102
13	dbsnp	rs9596775	A	G	ALLELIC	13/9	11/39	9.458	1	0.002102
13	dbsnp	rs9536381	A	G	ALLELIC	13/9	11/39	9.458	1	0.002102
13	dbsnp	rs2567778	A	C	ALLELIC	13/9	11/39	9.458	1	0.002102
14	dbsnp	rs12590112	G	A	ALLELIC	13/9	11/39	9.458	1	0.002102
14	dbsnp	rs4905298	G	A	ALLELIC	13/9	11/39	9.458	1	0.002102
14	dbsnp	rs4905963	A	G	ALLELIC	13/9	11/39	9.458	1	0.002102
14	dbsnp	rs8005782	A	G	ALLELIC	13/9	11/39	9.458	1	0.002102
14	dbsnp	rs7494065	G	A	ALLELIC	13/9	11/39	9.458	1	0.002102
15	dbsnp	rs4430697	G	A	ALLELIC	13/9	11/39	9.458	1	0.002102
16	dbsnp	rs3764286	G	A	ALLELIC	13/9	11/39	9.458	1	0.002102
16	dbsnp	rs8057850	G	A	ALLELIC	13/9	11/39	9.458	1	0.002102
16	dbsnp	rs9928856	A	G	ALLELIC	13/9	11/39	9.458	1	0.002102
16	dbsnp	rs8044338	A	G	ALLELIC	13/9	11/39	9.458	1	0.002102
16	dbsnp	rs4843542	A	G	ALLELIC	13/9	11/39	9.458	1	0.002102
18	dbsnp	rs2115980	C	A	ALLELIC	13/9	11/39	9.458	1	0.002102
21	dbsnp	rs7275695	A	G	ALLELIC	13/9	11/39	9.458	1	0.002102
21	dbsnp	rs7276622	A	G	ALLELIC	13/9	11/39	9.458	1	0.002102
23	dbsnp	rs980489	A	G	ALLELIC	13/9	11/39	9.458	1	0.002102
23	dbsnp	rs2213955	C	A	ALLELIC	13/9	11/39	9.458	1	0.002102
23	dbsnp	rs5929477	A	G	ALLELIC	13/9	11/39	9.458	1	0.002102
23	dbsnp	rs2097402	A	G	ALLELIC	13/9	11/39	9.458	1	0.002102
16	dbsnp	rs447205	A	G	ALLELIC	2/14	21/15	9.433	1	0.002131
1	dbsnp	rs867810	G	A	ALLELIC	17/5	19/31	9.425	1	0.00214
1	dbsnp	rs1004766	G	A	ALLELIC	17/5	19/31	9.425	1	0.00214
1	dbsnp	rs1337444	A	G	ALLELIC	5/17	31/19	9.425	1	0.00214
1	dbsnp	rs10753936	A	G	ALLELIC	17/5	19/31	9.425	1	0.00214
1	dbsnp	rs6693576	A	C	ALLELIC	17/5	19/31	9.425	1	0.00214
3	dbsnp	rs184653	C	A	ALLELIC	17/5	19/31	9.425	1	0.00214
4	dbsnp	rs12650562	A	G	ALLELIC	5/17	31/19	9.425	1	0.00214
4	dbsnp	rs7668399	A	G	ALLELIC	5/17	31/19	9.425	1	0.00214
4	dbsnp	rs893970	G	A	ALLELIC	5/17	31/19	9.425	1	0.00214
5	dbsnp	rs11948619	A	G	ALLELIC	5/17	31/19	9.425	1	0.00214
6	dbsnp	rs9369399	C	A	ALLELIC	5/17	31/19	9.425	1	0.00214
6	dbsnp	rs6936906	G	A	ALLELIC	5/17	31/19	9.425	1	0.00214
6	dbsnp	rs7759596	G	A	ALLELIC	5/17	31/19	9.425	1	0.00214

7	dbsnp	rs1862083	G	A	ALLELIC	5/17	31/19	9.425	1	0.00214
8	dbsnp	rs2280803	A	G	ALLELIC	5/17	31/19	9.425	1	0.00214
8	dbsnp	rs2466213	A	C	ALLELIC	5/17	31/19	9.425	1	0.00214
10	dbsnp	rs1329178	G	A	ALLELIC	17/5	19/31	9.425	1	0.00214
11	dbsnp	rs7104808	G	A	ALLELIC	5/17	31/19	9.425	1	0.00214
12	dbsnp	rs7952758	G	A	ALLELIC	5/17	31/19	9.425	1	0.00214
12	dbsnp	rs10850883	G	A	ALLELIC	5/17	31/19	9.425	1	0.00214
18	dbsnp	rs1560430	A	C	ALLELIC	17/5	19/31	9.425	1	0.00214
18	dbsnp	rs1430551	G	A	ALLELIC	17/5	19/31	9.425	1	0.00214
20	dbsnp	rs6031839	G	A	ALLELIC	17/5	19/31	9.425	1	0.00214
20	dbsnp	rs6020972	G	A	ALLELIC	5/17	31/19	9.425	1	0.00214
23	dbsnp	rs7057951	A	C	ALLELIC	17/5	19/31	9.425	1	0.00214
1	dbsnp	rs263533	A	G	ALLELIC	14/6	15/35	9.42	1	0.002147
3	dbsnp	rs4465907	A	G	ALLELIC	14/6	15/35	9.42	1	0.002147
10	dbsnp	rs2799003	C	A	ALLELIC	14/6	15/35	9.42	1	0.002147
13	dbsnp	rs1343610	A	G	ALLELIC	14/6	15/35	9.42	1	0.002147
14	dbsnp	rs1953273	A	G	ALLELIC	14/6	15/35	9.42	1	0.002147
16	dbsnp	rs7185776	A	C	ALLELIC	14/6	15/35	9.42	1	0.002147
18	dbsnp	rs4890569	A	G	ALLELIC	14/6	15/35	9.42	1	0.002147
18	dbsnp	rs1395849	A	G	ALLELIC	14/6	15/35	9.42	1	0.002147
1	dbsnp	rs1288520	A	G	ALLELIC	12/8	11/39	9.351	1	0.002229
1	dbsnp	rs386978	A	G	ALLELIC	12/8	11/39	9.351	1	0.002229
2	dbsnp	rs12989336	G	A	ALLELIC	12/8	11/39	9.351	1	0.002229
2	dbsnp	rs2300484	A	G	ALLELIC	12/8	11/39	9.351	1	0.002229
2	dbsnp	rs6547460	A	G	ALLELIC	12/8	11/39	9.351	1	0.002229
2	dbsnp	rs1518434	A	G	ALLELIC	12/8	11/39	9.351	1	0.002229
3	dbsnp	rs3846051	G	A	ALLELIC	12/8	11/39	9.351	1	0.002229
5	dbsnp	rs3822432	G	A	ALLELIC	12/8	11/39	9.351	1	0.002229
8	dbsnp	rs11136139	C	A	ALLELIC	12/8	11/39	9.351	1	0.002229
8	dbsnp	rs12543341	G	A	ALLELIC	12/8	11/39	9.351	1	0.002229
8	dbsnp	rs2226405	C	A	ALLELIC	12/8	11/39	9.351	1	0.002229
12	dbsnp	rs10877894	A	G	ALLELIC	12/8	11/39	9.351	1	0.002229
13	dbsnp	rs7334690	A	G	ALLELIC	12/8	11/39	9.351	1	0.002229
13	dbsnp	rs9536375	A	G	ALLELIC	12/8	11/39	9.351	1	0.002229
15	dbsnp	rs11630458	A	G	ALLELIC	12/8	11/39	9.351	1	0.002229
1	dbsnp	rs2778003	A	G	ALLELIC	3/19	26/24	9.348	1	0.002233
1	dbsnp	rs2758644	A	G	ALLELIC	3/19	26/24	9.348	1	0.002233
2	dbsnp	rs12712085	A	G	ALLELIC	3/19	26/24	9.348	1	0.002233
3	dbsnp	rs9310707	A	G	ALLELIC	3/19	26/24	9.348	1	0.002233
3	dbsnp	rs9869237	G	A	ALLELIC	3/19	26/24	9.348	1	0.002233
4	dbsnp	rs4555581	A	G	ALLELIC	3/19	26/24	9.348	1	0.002233
7	dbsnp	rs10081184	G	A	ALLELIC	3/19	26/24	9.348	1	0.002233
7	dbsnp	rs12538868	A	G	ALLELIC	3/19	26/24	9.348	1	0.002233
9	dbsnp	rs720268	A	G	ALLELIC	3/19	26/24	9.348	1	0.002233
13	dbsnp	rs7999638	C	A	ALLELIC	3/19	26/24	9.348	1	0.002233
14	dbsnp	rs1953430	G	A	ALLELIC	3/19	26/24	9.348	1	0.002233
14	dbsnp	rs12587814	G	A	ALLELIC	3/19	26/24	9.348	1	0.002233
14	dbsnp	rs929328	A	G	ALLELIC	3/19	26/24	9.348	1	0.002233

14	dbsnp	rs741099	G	A	ALLELIC	3/19	26/24	9.348	1	0.002233
18	dbsnp	rs599680	A	G	ALLELIC	3/19	26/24	9.348	1	0.002233
18	dbsnp	rs8092112	A	C	ALLELIC	3/19	26/24	9.348	1	0.002233
20	dbsnp	rs6040463	G	A	ALLELIC	3/19	26/24	9.348	1	0.002233
23	dbsnp	rs5915313	G	A	ALLELIC	3/19	26/24	9.348	1	0.002233
23	dbsnp	rs7886473	A	G	ALLELIC	3/19	26/24	9.348	1	0.002233
23	dbsnp	rs5985760	A	C	ALLELIC	3/19	26/24	9.348	1	0.002233
1	dbsnp	rs6669501	A	G	ALLELIC	9/13	5/45	9.318	1	0.002269
1	dbsnp	rs635154	A	G	ALLELIC	9/13	5/45	9.318	1	0.002269
1	dbsnp	rs4658714	G	A	ALLELIC	9/13	5/45	9.318	1	0.002269
2	dbsnp	rs16856070	A	G	ALLELIC	9/13	5/45	9.318	1	0.002269
2	dbsnp	rs7587413	C	A	ALLELIC	9/13	5/45	9.318	1	0.002269
2	dbsnp	rs10195015	A	G	ALLELIC	9/13	5/45	9.318	1	0.002269
3	dbsnp	rs11130935	G	A	ALLELIC	9/13	5/45	9.318	1	0.002269
3	dbsnp	rs6780927	A	G	ALLELIC	9/13	5/45	9.318	1	0.002269
4	dbsnp	rs2687427	G	A	ALLELIC	9/13	5/45	9.318	1	0.002269
4	dbsnp	rs4693532	A	G	ALLELIC	9/13	5/45	9.318	1	0.002269
4	dbsnp	rs7656460	G	A	ALLELIC	9/13	5/45	9.318	1	0.002269
4	dbsnp	rs1392751	A	G	ALLELIC	9/13	5/45	9.318	1	0.002269
5	dbsnp	rs167214	G	A	ALLELIC	9/13	5/45	9.318	1	0.002269
5	dbsnp	rs1374002	A	G	ALLELIC	9/13	5/45	9.318	1	0.002269
5	dbsnp	rs1995274	A	G	ALLELIC	9/13	5/45	9.318	1	0.002269
5	dbsnp	rs10075885	C	A	ALLELIC	9/13	5/45	9.318	1	0.002269
6	dbsnp	rs4257844	A	G	ALLELIC	9/13	5/45	9.318	1	0.002269
6	dbsnp	rs9487847	A	G	ALLELIC	9/13	5/45	9.318	1	0.002269
6	dbsnp	rs2237246	A	G	ALLELIC	9/13	5/45	9.318	1	0.002269
6	dbsnp	rs9375224	G	A	ALLELIC	9/13	5/45	9.318	1	0.002269
6	dbsnp	rs1746477	A	G	ALLELIC	9/13	5/45	9.318	1	0.002269
7	dbsnp	rs2526625	A	C	ALLELIC	9/13	5/45	9.318	1	0.002269
8	dbsnp	rs4298474	A	G	ALLELIC	9/13	5/45	9.318	1	0.002269
9	dbsnp	rs7048007	G	A	ALLELIC	9/13	5/45	9.318	1	0.002269
9	dbsnp	rs10970374	A	G	ALLELIC	9/13	5/45	9.318	1	0.002269
9	dbsnp	rs10813601	A	G	ALLELIC	9/13	5/45	9.318	1	0.002269
9	dbsnp	rs2447721	A	G	ALLELIC	9/13	5/45	9.318	1	0.002269
9	dbsnp	rs10970378	A	G	ALLELIC	9/13	5/45	9.318	1	0.002269
9	dbsnp	rs10511880	A	G	ALLELIC	9/13	5/45	9.318	1	0.002269
9	dbsnp	rs7022623	A	G	ALLELIC	9/13	5/45	9.318	1	0.002269
10	dbsnp	rs7905625	A	G	ALLELIC	9/13	5/45	9.318	1	0.002269
10	dbsnp	rs266088	A	G	ALLELIC	9/13	5/45	9.318	1	0.002269
10	dbsnp	rs807051	A	G	ALLELIC	9/13	5/45	9.318	1	0.002269
11	dbsnp	rs11022066	G	A	ALLELIC	9/13	5/45	9.318	1	0.002269
11	dbsnp	rs11037491	A	C	ALLELIC	9/13	5/45	9.318	1	0.002269
11	dbsnp	rs2902373	A	G	ALLELIC	9/13	5/45	9.318	1	0.002269
11	dbsnp	rs17574672	A	G	ALLELIC	9/13	5/45	9.318	1	0.002269
11	dbsnp	rs2884183	A	G	ALLELIC	9/13	5/45	9.318	1	0.002269
12	dbsnp	rs17776335	A	G	ALLELIC	9/13	5/45	9.318	1	0.002269
13	dbsnp	rs2049892	G	A	ALLELIC	9/13	5/45	9.318	1	0.002269
13	dbsnp	rs10508103	A	G	ALLELIC	9/13	5/45	9.318	1	0.002269

13	dbsnp	rs17546403	G	A	ALLELIC	9/13	5/45	9.318	1	0.002269
13	dbsnp	rs9525307	A	C	ALLELIC	9/13	5/45	9.318	1	0.002269
15	dbsnp	rs514438	G	A	ALLELIC	9/13	5/45	9.318	1	0.002269
15	dbsnp	rs12912744	A	G	ALLELIC	9/13	5/45	9.318	1	0.002269
15	dbsnp	rs16957680	C	A	ALLELIC	9/13	5/45	9.318	1	0.002269
15	dbsnp	rs17727853	C	A	ALLELIC	9/13	5/45	9.318	1	0.002269
15	dbsnp	rs16977964	A	G	ALLELIC	9/13	5/45	9.318	1	0.002269
15	dbsnp	rs16977968	G	A	ALLELIC	9/13	5/45	9.318	1	0.002269
18	dbsnp	rs7245160	A	G	ALLELIC	9/13	5/45	9.318	1	0.002269
20	dbsnp	rs6016886	G	A	ALLELIC	9/13	5/45	9.318	1	0.002269
20	dbsnp	rs6018446	G	A	ALLELIC	9/13	5/45	9.318	1	0.002269
23	dbsnp	rs12687999	A	C	ALLELIC	9/13	5/45	9.318	1	0.002269
23	dbsnp	rs6640369	A	C	ALLELIC	9/13	5/45	9.318	1	0.002269
1	dbsnp	rs1507735	A	G	ALLELIC	13/7	13/37	9.307	1	0.002283
1	dbsnp	rs12021580	G	A	ALLELIC	13/7	13/37	9.307	1	0.002283
2	dbsnp	rs12469609	A	C	ALLELIC	13/7	13/37	9.307	1	0.002283
2	dbsnp	rs12622959	G	A	ALLELIC	13/7	13/37	9.307	1	0.002283
2	dbsnp	rs2943633	A	C	ALLELIC	13/7	13/37	9.307	1	0.002283
9	dbsnp	rs2778635	A	G	ALLELIC	13/7	13/37	9.307	1	0.002283
11	dbsnp	rs621310	A	G	ALLELIC	13/7	13/37	9.307	1	0.002283
12	dbsnp	rs2055731	G	A	ALLELIC	13/7	13/37	9.307	1	0.002283
16	dbsnp	rs1424254	A	G	ALLELIC	13/7	13/37	9.307	1	0.002283
19	dbsnp	rs1007161	A	G	ALLELIC	13/7	13/37	9.307	1	0.002283
19	dbsnp	rs1007160	A	C	ALLELIC	13/7	13/37	9.307	1	0.002283
19	dbsnp	rs11084673	A	G	ALLELIC	13/7	13/37	9.307	1	0.002283
19	dbsnp	rs8106108	C	A	ALLELIC	7/13	3/45	9.303	1	0.002288
1	dbsnp	rs4846400	G	A	ALLELIC	1/21	20/30	9.296	1	0.002297
2	dbsnp	rs6753618	A	C	ALLELIC	1/21	20/30	9.296	1	0.002297
2	dbsnp	rs17180327	G	A	ALLELIC	1/21	20/30	9.296	1	0.002297
2	dbsnp	rs590233	A	C	ALLELIC	1/21	20/30	9.296	1	0.002297
2	dbsnp	rs204249	A	G	ALLELIC	1/21	20/30	9.296	1	0.002297
4	dbsnp	rs12500107	G	A	ALLELIC	1/21	20/30	9.296	1	0.002297
5	dbsnp	rs1469101	G	A	ALLELIC	1/21	20/30	9.296	1	0.002297
5	dbsnp	rs28111	A	G	ALLELIC	1/21	20/30	9.296	1	0.002297
5	dbsnp	rs10060182	A	G	ALLELIC	1/21	20/30	9.296	1	0.002297
7	dbsnp	rs1528997	A	G	ALLELIC	1/21	20/30	9.296	1	0.002297
8	dbsnp	rs10098490	G	A	ALLELIC	1/21	20/30	9.296	1	0.002297
8	dbsnp	rs4625043	G	A	ALLELIC	1/21	20/30	9.296	1	0.002297
8	dbsnp	rs6999948	A	G	ALLELIC	1/21	20/30	9.296	1	0.002297
8	dbsnp	rs12678425	A	G	ALLELIC	1/21	20/30	9.296	1	0.002297
9	dbsnp	rs10780947	A	G	ALLELIC	1/21	20/30	9.296	1	0.002297
11	dbsnp	rs4758330	A	G	ALLELIC	1/21	20/30	9.296	1	0.002297
11	dbsnp	rs2129283	A	G	ALLELIC	1/21	20/30	9.296	1	0.002297
20	dbsnp	rs2423011	A	G	ALLELIC	1/21	20/30	9.296	1	0.002297
23	dbsnp	rs12014197	A	G	ALLELIC	1/21	20/30	9.296	1	0.002297
23	dbsnp	rs7051574	C	A	ALLELIC	1/21	20/30	9.296	1	0.002297
23	dbsnp	rs4829215	G	A	ALLELIC	1/21	20/30	9.296	1	0.002297
23	dbsnp	rs4829216	G	A	ALLELIC	1/21	20/30	9.296	1	0.002297

1	dbsnp	rs11591055	A	C	ALLELIC	3/19	25/23	9.291	1	0.002302
8	dbsnp	rs1002792	A	G	ALLELIC	3/19	25/23	9.291	1	0.002302
10	dbsnp	rs2264603	A	G	ALLELIC	10/12	6/42	9.291	1	0.002302
14	dbsnp	rs2416012	G	A	ALLELIC	14/4	18/32	9.272	1	0.002326
4	dbsnp	rs10024879	A	G	ALLELIC	4/18	0/48	9.256	1	0.002347
15	dbsnp	rs33931006	C	G	ALLELIC	4/18	0/48	9.256	1	0.002347
23	dbsnp	rs16990111	G	A	ALLELIC	4/18	0/48	9.256	1	0.002347
1	dbsnp	rs7551503	A	G	ALLELIC	9/11	6/44	9.24	1	0.002368
1	dbsnp	rs2236591	A	G	ALLELIC	9/11	6/44	9.24	1	0.002368
1	dbsnp	rs2686231	C	A	ALLELIC	9/11	6/44	9.24	1	0.002368
2	dbsnp	rs1542190	G	A	ALLELIC	9/11	6/44	9.24	1	0.002368
7	dbsnp	rs273136	A	G	ALLELIC	9/11	6/44	9.24	1	0.002368
8	dbsnp	rs10216950	G	A	ALLELIC	9/11	6/44	9.24	1	0.002368
10	dbsnp	rs1150018	G	A	ALLELIC	9/11	6/44	9.24	1	0.002368
11	dbsnp	rs1461630	G	A	ALLELIC	9/11	6/44	9.24	1	0.002368
11	dbsnp	rs585447	G	A	ALLELIC	9/11	6/44	9.24	1	0.002368
15	dbsnp	rs11630090	G	A	ALLELIC	9/11	6/44	9.24	1	0.002368
1	dbsnp	rs1295107	A	G	ALLELIC	14/8	13/37	9.233	1	0.002376
1	dbsnp	rs1041159	G	A	ALLELIC	14/8	13/37	9.233	1	0.002376
2	dbsnp	rs880564	G	A	ALLELIC	14/8	13/37	9.233	1	0.002376
2	dbsnp	rs9309641	G	A	ALLELIC	14/8	13/37	9.233	1	0.002376
2	dbsnp	rs1157873	G	A	ALLELIC	14/8	13/37	9.233	1	0.002376
3	dbsnp	rs9857394	A	G	ALLELIC	14/8	13/37	9.233	1	0.002376
5	dbsnp	rs10866712	A	G	ALLELIC	14/8	13/37	9.233	1	0.002376
8	dbsnp	rs4872714	G	A	ALLELIC	14/8	13/37	9.233	1	0.002376
8	dbsnp	rs2252553	A	C	ALLELIC	14/8	13/37	9.233	1	0.002376
9	dbsnp	rs2778636	A	G	ALLELIC	14/8	13/37	9.233	1	0.002376
10	dbsnp	rs7074678	G	A	ALLELIC	14/8	13/37	9.233	1	0.002376
10	dbsnp	rs10882888	A	G	ALLELIC	14/8	13/37	9.233	1	0.002376
15	dbsnp	rs1817955	A	C	ALLELIC	14/8	13/37	9.233	1	0.002376
15	dbsnp	rs12917608	G	A	ALLELIC	14/8	13/37	9.233	1	0.002376
19	dbsnp	rs12150889	G	A	ALLELIC	14/8	13/37	9.233	1	0.002376
23	dbsnp	rs2092039	A	C	ALLELIC	14/8	13/37	9.233	1	0.002376
23	dbsnp	rs5987668	A	G	ALLELIC	14/8	13/37	9.233	1	0.002376
1	dbsnp	rs17540656	G	A	ALLELIC	16/6	17/33	9.23	1	0.002381
1	dbsnp	rs10920278	C	A	ALLELIC	16/6	17/33	9.23	1	0.002381
2	dbsnp	rs1000087	G	A	ALLELIC	16/6	17/33	9.23	1	0.002381
3	dbsnp	rs4681161	A	G	ALLELIC	16/6	17/33	9.23	1	0.002381
5	dbsnp	rs1025296	G	A	ALLELIC	16/6	17/33	9.23	1	0.002381
5	dbsnp	rs11134252	A	G	ALLELIC	16/6	17/33	9.23	1	0.002381
7	dbsnp	rs6970608	A	G	ALLELIC	16/6	17/33	9.23	1	0.002381
7	dbsnp	rs2919435	A	G	ALLELIC	16/6	17/33	9.23	1	0.002381
8	dbsnp	rs11786728	G	A	ALLELIC	16/6	17/33	9.23	1	0.002381
10	dbsnp	rs4880567	G	A	ALLELIC	16/6	17/33	9.23	1	0.002381
10	dbsnp	rs2983854	G	A	ALLELIC	16/6	17/33	9.23	1	0.002381
10	dbsnp	rs12256543	A	C	ALLELIC	16/6	17/33	9.23	1	0.002381
10	dbsnp	rs4147059	A	G	ALLELIC	16/6	17/33	9.23	1	0.002381
10	dbsnp	rs11245106	A	G	ALLELIC	16/6	17/33	9.23	1	0.002381

13	dbsnp	rs1926502	A	C	ALLELIC	16/6	17/33	9.23	1	0.002381
14	dbsnp	rs811340	A	G	ALLELIC	16/6	17/33	9.23	1	0.002381
14	dbsnp	rs8016110	A	C	ALLELIC	16/6	17/33	9.23	1	0.002381
14	dbsnp	rs17703029	G	A	ALLELIC	16/6	17/33	9.23	1	0.002381
16	dbsnp	rs11117451	A	G	ALLELIC	16/6	17/33	9.23	1	0.002381
17	dbsnp	rs7224405	A	C	ALLELIC	16/6	17/33	9.23	1	0.002381
18	dbsnp	rs9959828	A	G	ALLELIC	16/6	17/33	9.23	1	0.002381
23	dbsnp	rs5933668	G	A	ALLELIC	16/6	17/33	9.23	1	0.002381
21	dbsnp	rs2838713	G	A	ALLELIC	5/15	1/47	9.216	1	0.002399
13	dbsnp	rs9557837	C	A	ALLELIC	8/12	4/42	9.183	1	0.002443
1	dbsnp	rs6588147	G	A	ALLELIC	2/20	23/27	9.182	1	0.002444
2	dbsnp	rs2610683	G	A	ALLELIC	2/20	23/27	9.182	1	0.002444
2	dbsnp	rs2601474	G	A	ALLELIC	2/20	23/27	9.182	1	0.002444
2	dbsnp	rs2441351	A	C	ALLELIC	2/20	23/27	9.182	1	0.002444
2	dbsnp	rs11679411	A	G	ALLELIC	2/20	23/27	9.182	1	0.002444
3	dbsnp	rs13070800	G	A	ALLELIC	2/20	23/27	9.182	1	0.002444
4	dbsnp	rs16874378	G	A	ALLELIC	2/20	23/27	9.182	1	0.002444
5	dbsnp	rs595117	G	A	ALLELIC	2/20	23/27	9.182	1	0.002444
6	dbsnp	rs2076189	A	G	ALLELIC	2/20	23/27	9.182	1	0.002444
6	dbsnp	rs472072	G	A	ALLELIC	2/20	23/27	9.182	1	0.002444
6	dbsnp	rs7763862	C	A	ALLELIC	2/20	23/27	9.182	1	0.002444
6	dbsnp	rs747866	G	A	ALLELIC	2/20	23/27	9.182	1	0.002444
9	dbsnp	rs954974	A	C	ALLELIC	2/20	23/27	9.182	1	0.002444
10	dbsnp	rs4747148	C	A	ALLELIC	2/20	23/27	9.182	1	0.002444
11	dbsnp	rs11029134	A	G	ALLELIC	2/20	23/27	9.182	1	0.002444
13	dbsnp	rs3007762	A	G	ALLELIC	2/20	23/27	9.182	1	0.002444
19	dbsnp	rs874232	G	A	ALLELIC	2/20	23/27	9.182	1	0.002444
21	dbsnp	rs965494	A	G	ALLELIC	2/20	23/27	9.182	1	0.002444
1	dbsnp	rs2843146	A	C	ALLELIC	15/7	15/35	9.164	1	0.002469
1	dbsnp	rs10798295	A	G	ALLELIC	15/7	15/35	9.164	1	0.002469
1	dbsnp	rs2253618	G	A	ALLELIC	15/7	15/35	9.164	1	0.002469
1	dbsnp	rs815344	A	G	ALLELIC	15/7	15/35	9.164	1	0.002469
3	dbsnp	rs424695	A	G	ALLELIC	15/7	15/35	9.164	1	0.002469
3	dbsnp	rs7633618	G	A	ALLELIC	15/7	15/35	9.164	1	0.002469
4	dbsnp	rs6834178	A	G	ALLELIC	15/7	15/35	9.164	1	0.002469
4	dbsnp	rs1400338	G	A	ALLELIC	15/7	15/35	9.164	1	0.002469
5	dbsnp	rs1863977	A	G	ALLELIC	15/7	15/35	9.164	1	0.002469
6	dbsnp	rs2814380	G	A	ALLELIC	15/7	15/35	9.164	1	0.002469
6	dbsnp	rs2812683	G	A	ALLELIC	15/7	15/35	9.164	1	0.002469
6	dbsnp	rs9362770	C	A	ALLELIC	15/7	15/35	9.164	1	0.002469
6	dbsnp	rs2895618	A	G	ALLELIC	15/7	15/35	9.164	1	0.002469
6	dbsnp	rs9386476	A	C	ALLELIC	15/7	15/35	9.164	1	0.002469
7	dbsnp	rs1440454	G	A	ALLELIC	15/7	15/35	9.164	1	0.002469
8	dbsnp	rs7825337	G	A	ALLELIC	15/7	15/35	9.164	1	0.002469
8	dbsnp	rs9643245	A	G	ALLELIC	15/7	15/35	9.164	1	0.002469
9	dbsnp	rs10733669	A	G	ALLELIC	15/7	15/35	9.164	1	0.002469
10	dbsnp	rs2050352	G	A	ALLELIC	15/7	15/35	9.164	1	0.002469
10	dbsnp	rs2892445	A	G	ALLELIC	15/7	15/35	9.164	1	0.002469

10	dbsnp	rs1914159	G	A	ALLELIC	15/7	15/35	9.164	1	0.002469
10	dbsnp	rs2486118	A	G	ALLELIC	15/7	15/35	9.164	1	0.002469
12	dbsnp	rs10846139	G	A	ALLELIC	15/7	15/35	9.164	1	0.002469
12	dbsnp	rs2611279	A	C	ALLELIC	15/7	15/35	9.164	1	0.002469
12	dbsnp	rs6606743	A	G	ALLELIC	15/7	15/35	9.164	1	0.002469
12	dbsnp	rs10774932	G	A	ALLELIC	15/7	15/35	9.164	1	0.002469
13	dbsnp	rs1926501	A	G	ALLELIC	15/7	15/35	9.164	1	0.002469
15	dbsnp	rs12440161	A	C	ALLELIC	15/7	15/35	9.164	1	0.002469
15	dbsnp	rs11072464	A	G	ALLELIC	15/7	15/35	9.164	1	0.002469
16	dbsnp	rs9940585	G	A	ALLELIC	15/7	15/35	9.164	1	0.002469
17	dbsnp	rs7224261	C	A	ALLELIC	15/7	15/35	9.164	1	0.002469
18	dbsnp	rs2048329	G	A	ALLELIC	15/7	15/35	9.164	1	0.002469
18	dbsnp	rs11151621	G	A	ALLELIC	15/7	15/35	9.164	1	0.002469
23	dbsnp	rs6621370	A	G	ALLELIC	15/7	15/35	9.164	1	0.002469
2	dbsnp	rs6433435	C	A	ALLELIC	4/16	30/20	9.15	1	0.002487
8	dbsnp	rs6983976	G	A	ALLELIC	4/16	30/20	9.15	1	0.002487
9	dbsnp	rs462537	G	A	ALLELIC	4/16	30/20	9.15	1	0.002487
10	dbsnp	rs2493655	A	G	ALLELIC	16/4	20/30	9.15	1	0.002487
11	dbsnp	rs488928	A	G	ALLELIC	16/4	20/30	9.15	1	0.002487
17	dbsnp	rs2058364	A	C	ALLELIC	4/16	30/20	9.15	1	0.002487
1	dbsnp	rs11581245	A	G	ALLELIC	11/11	8/42	9.092	1	0.002567
1	dbsnp	rs2970544	A	G	ALLELIC	11/11	8/42	9.092	1	0.002567
1	dbsnp	rs10923201	G	A	ALLELIC	11/11	8/42	9.092	1	0.002567
1	dbsnp	rs3767767	C	A	ALLELIC	11/11	8/42	9.092	1	0.002567
1	dbsnp	rs11120616	A	G	ALLELIC	11/11	8/42	9.092	1	0.002567
1	dbsnp	rs12141500	A	G	ALLELIC	11/11	8/42	9.092	1	0.002567
1	dbsnp	rs1341715	G	A	ALLELIC	11/11	8/42	9.092	1	0.002567
2	dbsnp	rs3738880	C	A	ALLELIC	11/11	8/42	9.092	1	0.002567
2	dbsnp	rs1878516	A	G	ALLELIC	11/11	8/42	9.092	1	0.002567
2	dbsnp	rs11693648	C	A	ALLELIC	11/11	8/42	9.092	1	0.002567
2	dbsnp	rs17008320	G	A	ALLELIC	11/11	8/42	9.092	1	0.002567
2	dbsnp	rs1373582	G	A	ALLELIC	11/11	8/42	9.092	1	0.002567
2	dbsnp	rs7595292	G	A	ALLELIC	11/11	8/42	9.092	1	0.002567
3	dbsnp	rs360833	A	G	ALLELIC	11/11	8/42	9.092	1	0.002567
3	dbsnp	rs4541352	C	A	ALLELIC	11/11	8/42	9.092	1	0.002567
3	dbsnp	rs6764014	A	G	ALLELIC	11/11	8/42	9.092	1	0.002567
4	dbsnp	rs1516666	G	A	ALLELIC	11/11	8/42	9.092	1	0.002567
5	dbsnp	rs1351403	A	G	ALLELIC	11/11	8/42	9.092	1	0.002567
5	dbsnp	rs10041333	G	A	ALLELIC	11/11	8/42	9.092	1	0.002567
6	dbsnp	rs9378430	G	A	ALLELIC	11/11	8/42	9.092	1	0.002567
6	dbsnp	rs9350215	A	C	ALLELIC	11/11	8/42	9.092	1	0.002567
6	dbsnp	rs652462	A	C	ALLELIC	11/11	8/42	9.092	1	0.002567
7	dbsnp	rs2159851	G	A	ALLELIC	11/11	8/42	9.092	1	0.002567
7	dbsnp	rs17645907	G	A	ALLELIC	11/11	8/42	9.092	1	0.002567
8	dbsnp	rs11782783	G	A	ALLELIC	11/11	8/42	9.092	1	0.002567
8	dbsnp	rs11784090	A	G	ALLELIC	11/11	8/42	9.092	1	0.002567
8	dbsnp	rs7015818	A	G	ALLELIC	11/11	8/42	9.092	1	0.002567
10	dbsnp	rs10883931	A	G	ALLELIC	11/11	8/42	9.092	1	0.002567

10	dbsnp	rs12414627	G	A	ALLELIC	11/11	8/42	9.092	1	0.002567
10	dbsnp	rs1710313	C	A	ALLELIC	11/11	8/42	9.092	1	0.002567
12	dbsnp	rs7136376	G	A	ALLELIC	11/11	8/42	9.092	1	0.002567
12	dbsnp	rs7978008	A	G	ALLELIC	11/11	8/42	9.092	1	0.002567
12	dbsnp	rs12579014	A	C	ALLELIC	11/11	8/42	9.092	1	0.002567
16	dbsnp	rs3111601	G	A	ALLELIC	11/11	8/42	9.092	1	0.002567
16	dbsnp	rs9939076	A	G	ALLELIC	11/11	8/42	9.092	1	0.002567
17	dbsnp	rs4796485	G	A	ALLELIC	11/11	8/42	9.092	1	0.002567
17	dbsnp	rs12451121	A	G	ALLELIC	11/11	8/42	9.092	1	0.002567
18	dbsnp	rs2322100	A	G	ALLELIC	11/11	8/42	9.092	1	0.002567
20	dbsnp	rs6077755	A	C	ALLELIC	11/11	8/42	9.092	1	0.002567
20	dbsnp	rs6013271	G	A	ALLELIC	11/11	8/42	9.092	1	0.002567
23	dbsnp	rs5979897	A	G	ALLELIC	11/11	8/42	9.092	1	0.002567
23	dbsnp	rs10283978	A	G	ALLELIC	11/11	8/42	9.092	1	0.002567
23	dbsnp	rs5990020	A	G	ALLELIC	11/11	8/42	9.092	1	0.002567
23	dbsnp	rs4969557	A	G	ALLELIC	11/11	8/42	9.092	1	0.002567
23	dbsnp	rs5928556	A	G	ALLELIC	11/11	8/42	9.092	1	0.002567
23	dbsnp	rs4824621	G	A	ALLELIC	11/11	8/42	9.092	1	0.002567
23	dbsnp	rs5953063	A	G	ALLELIC	11/11	8/42	9.092	1	0.002567
23	dbsnp	rs12687917	A	G	ALLELIC	11/11	8/42	9.092	1	0.002567
23	dbsnp	rs3924126	A	G	ALLELIC	11/11	8/42	9.092	1	0.002567
23	dbsnp	rs1494130	A	G	ALLELIC	11/11	8/42	9.092	1	0.002567
23	dbsnp	rs232368	A	G	ALLELIC	11/11	8/42	9.092	1	0.002567
23	dbsnp	rs12559898	A	G	ALLELIC	11/11	8/42	9.092	1	0.002567
23	dbsnp	rs6608163	A	G	ALLELIC	11/11	8/42	9.092	1	0.002567
23	dbsnp	rs6649176	A	G	ALLELIC	11/11	8/42	9.092	1	0.002567
1	dbsnp	rs535509	G	A	ALLELIC	6/14	2/46	9.076	1	0.00259
3	dbsnp	rs6793001	A	G	ALLELIC	6/14	2/46	9.076	1	0.00259
2	dbsnp	rs749285	G	A	ALLELIC	1/19	21/29	9.075	1	0.002592
2	dbsnp	rs11691600	A	G	ALLELIC	1/19	21/29	9.075	1	0.002592
6	dbsnp	rs12211229	A	G	ALLELIC	1/21	19/29	9.075	1	0.002592
6	dbsnp	rs2517554	G	A	ALLELIC	1/19	21/29	9.075	1	0.002592
6	dbsnp	rs1873337	C	A	ALLELIC	1/19	21/29	9.075	1	0.002592
10	dbsnp	rs12771818	A	G	ALLELIC	1/21	19/29	9.075	1	0.002592
1	dbsnp	rs2025579	A	G	ALLELIC	0/22	16/34	9.051	1	0.002625
3	dbsnp	rs6802030	A	G	ALLELIC	0/22	16/34	9.051	1	0.002625
4	dbsnp	rs629542	C	A	ALLELIC	0/22	16/34	9.051	1	0.002625
6	dbsnp	rs2328629	G	A	ALLELIC	0/22	16/34	9.051	1	0.002625
17	dbsnp	rs10902639	A	G	ALLELIC	0/22	16/34	9.051	1	0.002625
17	dbsnp	rs733099	A	G	ALLELIC	0/22	16/34	9.051	1	0.002625
17	dbsnp	rs2683134	A	G	ALLELIC	0/22	16/34	9.051	1	0.002625
23	dbsnp	rs12842492	G	A	ALLELIC	0/22	16/34	9.051	1	0.002625
1	dbsnp	rs12142130	A	G	ALLELIC	0/20	17/33	8.981	1	0.002728
2	dbsnp	rs16854131	C	A	ALLELIC	0/20	17/33	8.981	1	0.002728
6	dbsnp	rs94860	A	G	ALLELIC	0/20	17/33	8.981	1	0.002728
20	dbsnp	rs6045868	A	G	ALLELIC	0/20	17/33	8.981	1	0.002728
20	dbsnp	rs10470023	G	A	ALLELIC	0/20	17/33	8.981	1	0.002728
20	dbsnp	rs6010746	A	G	ALLELIC	0/20	17/33	8.981	1	0.002728

23	dbsnp	rs2188747	A	G	ALLELIC	0/20	17/33	8.981	1	0.002728
1	dbsnp	rs6690619	A	G	ALLELIC	3/17	27/23	8.872	1	0.002895
1	dbsnp	rs4341393	A	G	ALLELIC	3/17	27/23	8.872	1	0.002895
1	dbsnp	rs9426938	A	G	ALLELIC	3/17	27/23	8.872	1	0.002895
1	dbsnp	rs4845364	A	G	ALLELIC	3/17	27/23	8.872	1	0.002895
4	dbsnp	rs6535429	A	G	ALLELIC	3/17	27/23	8.872	1	0.002895
4	dbsnp	rs2298999	T	C	ALLELIC	3/17	27/23	8.872	1	0.002895
6	dbsnp	rs9442858	A	G	ALLELIC	3/17	27/23	8.872	1	0.002895
7	dbsnp	rs2450847	A	G	ALLELIC	3/17	27/23	8.872	1	0.002895
9	dbsnp	rs10970932	A	G	ALLELIC	3/17	27/23	8.872	1	0.002895
10	dbsnp	rs2447643	G	A	ALLELIC	3/17	27/23	8.872	1	0.002895
12	dbsnp	rs5027920	A	G	ALLELIC	3/17	27/23	8.872	1	0.002895
13	dbsnp	rs7327522	G	A	ALLELIC	3/17	27/23	8.872	1	0.002895
15	dbsnp	rs6493540	G	A	ALLELIC	3/17	27/23	8.872	1	0.002895
1	dbsnp	rs11120923	G	A	ALLELIC	8/14	4/46	8.849	1	0.002932
1	dbsnp	rs7542907	A	G	ALLELIC	8/14	4/46	8.849	1	0.002932
1	dbsnp	rs17509369	C	A	ALLELIC	8/14	4/46	8.849	1	0.002932
1	dbsnp	rs528629	A	C	ALLELIC	8/14	4/46	8.849	1	0.002932
1	dbsnp	rs6593669	G	A	ALLELIC	8/14	4/46	8.849	1	0.002932
1	dbsnp	rs10923228	A	G	ALLELIC	8/14	4/46	8.849	1	0.002932
1	dbsnp	rs6681289	A	G	ALLELIC	8/14	4/46	8.849	1	0.002932
1	dbsnp	rs2777962	A	G	ALLELIC	4/18	28/22	8.849	1	0.002932
1	dbsnp	rs856142	A	G	ALLELIC	8/14	4/46	8.849	1	0.002932
1	dbsnp	rs7556435	A	G	ALLELIC	8/14	4/46	8.849	1	0.002932
1	dbsnp	rs7540655	A	G	ALLELIC	8/14	4/46	8.849	1	0.002932
1	dbsnp	rs1361748	G	A	ALLELIC	4/18	28/22	8.849	1	0.002932
2	dbsnp	rs6738183	G	A	ALLELIC	8/14	4/46	8.849	1	0.002932
2	dbsnp	rs12991296	G	A	ALLELIC	8/14	4/46	8.849	1	0.002932
2	dbsnp	rs10174959	C	A	ALLELIC	4/18	28/22	8.849	1	0.002932
2	dbsnp	rs2358235	A	G	ALLELIC	4/18	28/22	8.849	1	0.002932
2	dbsnp	rs6711272	G	A	ALLELIC	8/14	4/46	8.849	1	0.002932
2	dbsnp	rs6714545	A	C	ALLELIC	8/14	4/46	8.849	1	0.002932
2	dbsnp	rs6748498	C	A	ALLELIC	8/14	4/46	8.849	1	0.002932
3	dbsnp	rs6778030	A	G	ALLELIC	4/18	28/22	8.849	1	0.002932
3	dbsnp	rs358597	G	A	ALLELIC	8/14	4/46	8.849	1	0.002932
3	dbsnp	rs358598	A	G	ALLELIC	8/14	4/46	8.849	1	0.002932
3	dbsnp	rs4687113	A	G	ALLELIC	8/14	4/46	8.849	1	0.002932
4	dbsnp	rs17500058	G	A	ALLELIC	8/14	4/46	8.849	1	0.002932
4	dbsnp	rs6835451	G	A	ALLELIC	8/14	4/46	8.849	1	0.002932
4	dbsnp	rs10857365	C	A	ALLELIC	8/14	4/46	8.849	1	0.002932
4	dbsnp	rs17258176	G	A	ALLELIC	8/14	4/46	8.849	1	0.002932
5	dbsnp	rs1011624	G	A	ALLELIC	8/14	4/46	8.849	1	0.002932
5	dbsnp	rs12516966	A	G	ALLELIC	8/14	4/46	8.849	1	0.002932
5	dbsnp	rs6595951	A	C	ALLELIC	8/14	4/46	8.849	1	0.002932
5	dbsnp	rs6874084	A	G	ALLELIC	8/14	4/46	8.849	1	0.002932
5	dbsnp	rs4976578	A	G	ALLELIC	8/14	4/46	8.849	1	0.002932
5	dbsnp	rs883150	C	A	ALLELIC	8/14	4/46	8.849	1	0.002932
6	dbsnp	rs6934209	A	G	ALLELIC	8/14	4/46	8.849	1	0.002932

6	dbsnp	rs16894557	A	G	ALLELIC	8/14	4/46	8.849	1	0.002932
6	dbsnp	rs3096691	A	G	ALLELIC	4/18	28/22	8.849	1	0.002932
6	dbsnp	rs6931103	G	A	ALLELIC	8/14	4/46	8.849	1	0.002932
6	dbsnp	rs12192606	G	A	ALLELIC	8/14	4/46	8.849	1	0.002932
6	dbsnp	rs11153361	A	G	ALLELIC	8/14	4/46	8.849	1	0.002932
6	dbsnp	rs11153362	A	C	ALLELIC	8/14	4/46	8.849	1	0.002932
6	dbsnp	rs12199067	G	A	ALLELIC	8/14	4/46	8.849	1	0.002932
6	dbsnp	rs7754221	C	A	ALLELIC	8/14	4/46	8.849	1	0.002932
7	dbsnp	rs6944332	A	G	ALLELIC	8/14	4/46	8.849	1	0.002932
7	dbsnp	rs7788676	G	A	ALLELIC	8/14	4/46	8.849	1	0.002932
7	dbsnp	rs34494536	G	A	ALLELIC	8/14	4/46	8.849	1	0.002932
7	dbsnp	rs10239000	A	G	ALLELIC	8/14	4/46	8.849	1	0.002932
8	dbsnp	rs7825784	A	G	ALLELIC	8/14	4/46	8.849	1	0.002932
8	dbsnp	rs6995710	A	G	ALLELIC	8/14	4/46	8.849	1	0.002932
8	dbsnp	rs2205258	A	G	ALLELIC	8/14	4/46	8.849	1	0.002932
8	dbsnp	rs2385295	C	A	ALLELIC	4/18	28/22	8.849	1	0.002932
8	dbsnp	rs12542467	C	A	ALLELIC	4/18	28/22	8.849	1	0.002932
9	dbsnp	rs7026536	G	A	ALLELIC	8/14	4/46	8.849	1	0.002932
9	dbsnp	rs11137521	G	A	ALLELIC	8/14	4/46	8.849	1	0.002932
9	dbsnp	rs11137533	A	G	ALLELIC	8/14	4/46	8.849	1	0.002932
9	dbsnp	rs11788194	A	G	ALLELIC	8/14	4/46	8.849	1	0.002932
9	dbsnp	rs7873896	A	G	ALLELIC	8/14	4/46	8.849	1	0.002932
9	dbsnp	rs12344392	A	G	ALLELIC	8/14	4/46	8.849	1	0.002932
9	dbsnp	rs12350202	G	A	ALLELIC	8/14	4/46	8.849	1	0.002932
10	dbsnp	rs1570844	A	G	ALLELIC	8/14	4/46	8.849	1	0.002932
10	dbsnp	rs10828101	A	G	ALLELIC	8/14	4/46	8.849	1	0.002932
10	dbsnp	rs10828105	A	G	ALLELIC	8/14	4/46	8.849	1	0.002932
10	dbsnp	rs2683548	G	A	ALLELIC	8/14	4/46	8.849	1	0.002932
10	dbsnp	rs11597420	A	G	ALLELIC	8/14	4/46	8.849	1	0.002932
11	dbsnp	rs1352524	A	G	ALLELIC	8/14	4/46	8.849	1	0.002932
12	dbsnp	rs34263	G	A	ALLELIC	8/14	4/46	8.849	1	0.002932
12	dbsnp	rs4766640	A	G	ALLELIC	4/18	28/22	8.849	1	0.002932
12	dbsnp	rs35471	G	A	ALLELIC	8/14	4/46	8.849	1	0.002932
12	dbsnp	rs10744185	G	A	ALLELIC	4/18	28/22	8.849	1	0.002932
12	dbsnp	rs11059209	A	G	ALLELIC	8/14	4/46	8.849	1	0.002932
12	dbsnp	rs1872520	C	A	ALLELIC	8/14	4/46	8.849	1	0.002932
13	dbsnp	rs9576175	C	A	ALLELIC	4/18	28/22	8.849	1	0.002932
13	dbsnp	rs7987512	A	C	ALLELIC	8/14	4/46	8.849	1	0.002932
13	dbsnp	rs4886202	A	G	ALLELIC	4/18	28/22	8.849	1	0.002932
13	dbsnp	rs169421	C	A	ALLELIC	4/18	28/22	8.849	1	0.002932
13	dbsnp	rs9525293	A	G	ALLELIC	8/14	4/46	8.849	1	0.002932
14	dbsnp	rs178485	G	A	ALLELIC	8/14	4/46	8.849	1	0.002932
15	dbsnp	rs4778893	A	G	ALLELIC	8/14	4/46	8.849	1	0.002932
15	dbsnp	rs7171722	C	A	ALLELIC	8/14	4/46	8.849	1	0.002932
16	dbsnp	rs7194733	G	A	ALLELIC	8/14	4/46	8.849	1	0.002932
16	dbsnp	rs2352931	G	A	ALLELIC	4/18	28/22	8.849	1	0.002932
17	dbsnp	rs7207359	A	G	ALLELIC	4/18	28/22	8.849	1	0.002932
17	dbsnp	rs277070	G	A	ALLELIC	8/14	4/46	8.849	1	0.002932

17	dbsnp	rs17687191	A	G	ALLELIC	8/14	4/46	8.849	1	0.002932
18	dbsnp	rs11877277	G	A	ALLELIC	4/18	28/22	8.849	1	0.002932
18	dbsnp	rs1241084	A	G	ALLELIC	8/14	4/46	8.849	1	0.002932
20	dbsnp	rs1699233	G	A	ALLELIC	4/18	28/22	8.849	1	0.002932
20	dbsnp	rs6058637	G	A	ALLELIC	8/14	4/46	8.849	1	0.002932
20	dbsnp	rs285207	C	A	ALLELIC	8/14	4/46	8.849	1	0.002932
20	dbsnp	rs285206	G	A	ALLELIC	8/14	4/46	8.849	1	0.002932
20	dbsnp	rs454255	A	C	ALLELIC	8/14	4/46	8.849	1	0.002932
21	dbsnp	rs2269127	A	G	ALLELIC	8/14	4/46	8.849	1	0.002932
22	dbsnp	rs5994129	G	A	ALLELIC	4/18	28/22	8.849	1	0.002932
22	dbsnp	rs400946	A	G	ALLELIC	8/14	4/46	8.849	1	0.002932
23	dbsnp	rs5979155	A	G	ALLELIC	8/14	4/46	8.849	1	0.002932
23	dbsnp	rs17322183	A	G	ALLELIC	8/14	4/46	8.849	1	0.002932
23	dbsnp	rs6633598	A	G	ALLELIC	4/18	28/22	8.849	1	0.002932
23	dbsnp	rs5970856	A	G	ALLELIC	4/18	28/22	8.849	1	0.002932
23	dbsnp	rs3935724	C	A	ALLELIC	4/18	28/22	8.849	1	0.002932
23	dbsnp	rs5927916	G	A	ALLELIC	8/14	4/46	8.849	1	0.002932
23	dbsnp	rs17282641	G	A	ALLELIC	8/14	4/46	8.849	1	0.002932
23	dbsnp	rs17331756	C	A	ALLELIC	8/14	4/46	8.849	1	0.002932
23	dbsnp	rs6528564	A	C	ALLELIC	8/14	4/46	8.849	1	0.002932
13	dbsnp	rs4344597	G	A	ALLELIC	6/2	6/24	8.842	1	0.002943
4	dbsnp	rs17440280	G	A	ALLELIC	2/18	24/26	8.836	1	0.002954
6	dbsnp	rs495610	A	G	ALLELIC	2/18	24/26	8.836	1	0.002954
8	dbsnp	rs328097	G	A	ALLELIC	2/18	24/26	8.836	1	0.002954
9	dbsnp	rs10781380	G	A	ALLELIC	2/18	24/26	8.836	1	0.002954
11	dbsnp	rs1551184	G	A	ALLELIC	2/18	24/26	8.836	1	0.002954
19	dbsnp	rs10405154	G	A	ALLELIC	2/18	24/26	8.836	1	0.002954
19	dbsnp	rs10421748	A	G	ALLELIC	2/18	24/26	8.836	1	0.002954
19	dbsnp	rs2091181	C	A	ALLELIC	2/18	24/26	8.836	1	0.002954
20	dbsnp	rs400735	G	A	ALLELIC	2/18	24/26	8.836	1	0.002954
1	dbsnp	rs11264736	G	A	ALLELIC	3/17	24/20	8.816	1	0.002985
2	dbsnp	rs1549020	C	A	ALLELIC	9/13	5/43	8.767	1	0.003068
11	dbsnp	rs505689	G	A	ALLELIC	13/9	11/37	8.762	1	0.003076
14	dbsnp	rs7147282	A	C	ALLELIC	2/18	23/25	8.73	1	0.00313
1	dbsnp	rs2224823	A	G	ALLELIC	15/5	18/32	8.72	1	0.003148
6	dbsnp	rs514769	G	A	ALLELIC	15/5	18/32	8.72	1	0.003148
6	dbsnp	rs1552855	G	A	ALLELIC	15/5	18/32	8.72	1	0.003148
9	dbsnp	rs2152647	G	A	ALLELIC	15/5	18/32	8.72	1	0.003148
9	dbsnp	rs4838177	G	A	ALLELIC	15/5	18/32	8.72	1	0.003148
10	dbsnp	rs2061071	G	A	ALLELIC	15/5	18/32	8.72	1	0.003148
11	dbsnp	rs7350545	G	A	ALLELIC	15/5	18/32	8.72	1	0.003148
12	dbsnp	rs7958583	A	G	ALLELIC	15/5	18/32	8.72	1	0.003148
20	dbsnp	rs749922	G	A	ALLELIC	15/5	18/32	8.72	1	0.003148
23	dbsnp	rs2858172	A	G	ALLELIC	5/15	32/18	8.72	1	0.003148
23	dbsnp	rs4828057	A	C	ALLELIC	5/15	32/18	8.72	1	0.003148
23	dbsnp	rs2022471	G	A	ALLELIC	5/15	32/18	8.72	1	0.003148
23	dbsnp	rs875450	A	G	ALLELIC	5/15	32/18	8.72	1	0.003148
22	dbsnp	rs5755039	A	G	ALLELIC	3/15	0/50	8.718	1	0.003151

14	dbsnp	rs4899016	C	A	ALLELIC	12/8	11/37	8.674	1	0.003229
23	dbsnp	rs5974854	A	G	ALLELIC	9/11	6/42	8.673	1	0.003229
1	dbsnp	rs10797961	G	A	ALLELIC	10/10	8/42	8.645	1	0.003279
5	dbsnp	rs10512645	G	A	ALLELIC	10/10	8/42	8.645	1	0.003279
5	dbsnp	rs248492	A	G	ALLELIC	10/10	8/42	8.645	1	0.003279
5	dbsnp	rs152276	G	A	ALLELIC	10/10	8/42	8.645	1	0.003279
6	dbsnp	rs2124006	G	A	ALLELIC	10/10	8/42	8.645	1	0.003279
7	dbsnp	rs4472405	G	A	ALLELIC	10/10	8/42	8.645	1	0.003279
7	dbsnp	rs6459686	G	A	ALLELIC	10/10	8/42	8.645	1	0.003279
8	dbsnp	rs2945861	A	G	ALLELIC	10/10	8/42	8.645	1	0.003279
9	dbsnp	rs16923195	C	A	ALLELIC	10/10	8/42	8.645	1	0.003279
10	dbsnp	rs11186048	G	A	ALLELIC	10/10	8/42	8.645	1	0.003279
14	dbsnp	rs2252267	A	G	ALLELIC	10/10	8/42	8.645	1	0.003279
14	dbsnp	rs10131278	A	G	ALLELIC	10/10	8/42	8.645	1	0.003279
15	dbsnp	rs625961	A	G	ALLELIC	10/10	8/42	8.645	1	0.003279
18	dbsnp	rs2358759	A	G	ALLELIC	10/10	8/42	8.645	1	0.003279
18	dbsnp	rs2031042	G	A	ALLELIC	10/10	8/42	8.645	1	0.003279
20	dbsnp	rs16997896	A	G	ALLELIC	10/10	8/42	8.645	1	0.003279
23	dbsnp	rs7878615	G	A	ALLELIC	6/12	3/47	8.611	1	0.003342
1	dbsnp	rs1291050	G	A	ALLELIC	8/12	1/23	8.609	1	0.003345
11	dbsnp	rs11029398	G	A	ALLELIC	16/6	16/30	8.601	1	0.00336
17	dbsnp	rs9903614	C	A	ALLELIC	13/7	13/35	8.594	1	0.003372
23	dbsnp	rs4907870	G	A	ALLELIC	13/7	13/35	8.594	1	0.003372
1	dbsnp	rs11805096	A	G	ALLELIC	5/17	1/49	8.592	1	0.003376
1	dbsnp	rs7540336	G	A	ALLELIC	5/17	1/49	8.592	1	0.003376
1	dbsnp	rs11264127	A	G	ALLELIC	5/17	1/49	8.592	1	0.003376
1	dbsnp	rs12075713	C	A	ALLELIC	5/17	1/49	8.592	1	0.003376
1	dbsnp	rs659215	A	G	ALLELIC	5/17	1/49	8.592	1	0.003376
1	dbsnp	rs6663920	A	G	ALLELIC	5/17	1/49	8.592	1	0.003376
1	dbsnp	rs12035525	A	C	ALLELIC	5/17	1/49	8.592	1	0.003376
1	dbsnp	rs11803470	A	G	ALLELIC	5/17	1/49	8.592	1	0.003376
1	dbsnp	rs1582123	A	G	ALLELIC	5/17	1/49	8.592	1	0.003376
1	dbsnp	rs12125717	A	G	ALLELIC	5/17	1/49	8.592	1	0.003376
1	dbsnp	rs1766803	G	A	ALLELIC	12/10	10/40	8.592	1	0.003376
1	dbsnp	rs3131310	A	G	ALLELIC	12/10	10/40	8.592	1	0.003376
1	dbsnp	rs607407	C	A	ALLELIC	12/10	10/40	8.592	1	0.003376
1	dbsnp	rs590360	G	A	ALLELIC	12/10	10/40	8.592	1	0.003376
1	dbsnp	rs2796160	A	C	ALLELIC	5/17	1/49	8.592	1	0.003376
1	dbsnp	rs2802944	G	A	ALLELIC	5/17	1/49	8.592	1	0.003376
1	dbsnp	rs11804678	A	C	ALLELIC	5/17	1/49	8.592	1	0.003376
1	dbsnp	rs4351686	G	A	ALLELIC	5/17	1/49	8.592	1	0.003376
1	dbsnp	rs7551871	A	G	ALLELIC	5/17	1/49	8.592	1	0.003376
1	dbsnp	rs17269923	A	C	ALLELIC	5/17	1/49	8.592	1	0.003376
1	dbsnp	rs10924968	A	G	ALLELIC	5/17	1/49	8.592	1	0.003376
2	dbsnp	rs1472780	A	C	ALLELIC	12/10	10/40	8.592	1	0.003376
2	dbsnp	rs2255177	G	A	ALLELIC	12/10	10/40	8.592	1	0.003376
2	dbsnp	rs6736412	A	G	ALLELIC	5/17	1/49	8.592	1	0.003376
2	dbsnp	rs1504262	A	G	ALLELIC	5/17	1/49	8.592	1	0.003376

2	dbsnp	rs1922092	A	G	ALLELIC	5/17	1/49	8.592	1	0.003376
2	dbsnp	rs934798	G	A	ALLELIC	5/17	1/49	8.592	1	0.003376
2	dbsnp	rs17760522	G	A	ALLELIC	5/17	1/49	8.592	1	0.003376
2	dbsnp	rs12618482	G	A	ALLELIC	5/17	1/49	8.592	1	0.003376
2	dbsnp	rs10203445	C	A	ALLELIC	5/17	1/49	8.592	1	0.003376
3	dbsnp	rs155834	A	G	ALLELIC	5/17	1/49	8.592	1	0.003376
3	dbsnp	rs12497831	A	G	ALLELIC	5/17	1/49	8.592	1	0.003376
3	dbsnp	rs1449688	G	A	ALLELIC	5/17	1/49	8.592	1	0.003376
3	dbsnp	rs12498069	A	C	ALLELIC	5/17	1/49	8.592	1	0.003376
3	dbsnp	rs3755733	C	A	ALLELIC	5/17	1/49	8.592	1	0.003376
3	dbsnp	rs6440177	A	G	ALLELIC	5/17	1/49	8.592	1	0.003376
3	dbsnp	rs9838353	A	G	ALLELIC	5/17	1/49	8.592	1	0.003376
3	dbsnp	rs7559	G	A	ALLELIC	5/17	1/49	8.592	1	0.003376
4	dbsnp	rs17164836	G	A	ALLELIC	5/17	1/49	8.592	1	0.003376
4	dbsnp	rs12186156	G	A	ALLELIC	5/17	1/49	8.592	1	0.003376
4	dbsnp	rs6599351	A	G	ALLELIC	5/17	1/49	8.592	1	0.003376
4	dbsnp	rs6533958	G	A	ALLELIC	5/17	1/49	8.592	1	0.003376
4	dbsnp	rs970943	A	G	ALLELIC	5/17	1/49	8.592	1	0.003376
4	dbsnp	rs6826566	A	G	ALLELIC	5/17	1/49	8.592	1	0.003376
4	dbsnp	rs10008795	A	G	ALLELIC	5/17	1/49	8.592	1	0.003376
5	dbsnp	rs13173735	C	A	ALLELIC	5/17	1/49	8.592	1	0.003376
5	dbsnp	rs10941473	G	A	ALLELIC	5/17	1/49	8.592	1	0.003376
5	dbsnp	rs3922417	G	A	ALLELIC	5/17	1/49	8.592	1	0.003376
5	dbsnp	rs2115766	C	A	ALLELIC	5/17	1/49	8.592	1	0.003376
5	dbsnp	rs7726250	A	G	ALLELIC	5/17	1/49	8.592	1	0.003376
5	dbsnp	rs13172414	C	A	ALLELIC	5/17	1/49	8.592	1	0.003376
5	dbsnp	rs6556104	A	G	ALLELIC	5/17	1/49	8.592	1	0.003376
5	dbsnp	rs359436	A	C	ALLELIC	5/17	1/49	8.592	1	0.003376
5	dbsnp	rs6869682	C	A	ALLELIC	5/17	1/49	8.592	1	0.003376
5	dbsnp	rs7726709	G	A	ALLELIC	5/17	1/49	8.592	1	0.003376
5	dbsnp	rs2909705	G	A	ALLELIC	12/10	10/40	8.592	1	0.003376
6	dbsnp	rs9378357	A	C	ALLELIC	5/17	1/49	8.592	1	0.003376
6	dbsnp	rs9262560	A	G	ALLELIC	5/17	1/49	8.592	1	0.003376
6	dbsnp	rs2045577	G	A	ALLELIC	5/17	1/49	8.592	1	0.003376
6	dbsnp	rs2253676	A	G	ALLELIC	5/17	1/49	8.592	1	0.003376
6	dbsnp	rs2803496	G	A	ALLELIC	5/17	1/49	8.592	1	0.003376
6	dbsnp	rs1982756	A	C	ALLELIC	5/17	1/49	8.592	1	0.003376
6	dbsnp	rs7757302	A	G	ALLELIC	12/10	10/40	8.592	1	0.003376
6	dbsnp	rs1782783	G	A	ALLELIC	5/17	1/49	8.592	1	0.003376
6	dbsnp	rs2497108	A	G	ALLELIC	12/10	10/40	8.592	1	0.003376
6	dbsnp	rs17729906	G	A	ALLELIC	12/10	10/40	8.592	1	0.003376
6	dbsnp	rs2497113	A	G	ALLELIC	12/10	10/40	8.592	1	0.003376
6	dbsnp	rs12190936	G	A	ALLELIC	12/10	10/40	8.592	1	0.003376
6	dbsnp	rs1969811	G	A	ALLELIC	5/17	1/49	8.592	1	0.003376
6	dbsnp	rs6903235	G	A	ALLELIC	12/10	10/40	8.592	1	0.003376
6	dbsnp	rs17749667	G	A	ALLELIC	12/10	10/40	8.592	1	0.003376
6	dbsnp	rs1570363	G	A	ALLELIC	12/10	10/40	8.592	1	0.003376
6	dbsnp	rs7451668	A	C	ALLELIC	5/17	1/49	8.592	1	0.003376

6	dbsnp	rs13215619	G	A	ALLELIC	5/17	1/49	8.592	1	0.003376
7	dbsnp	rs4991791	A	G	ALLELIC	5/17	1/49	8.592	1	0.003376
7	dbsnp	rs2163640	G	A	ALLELIC	5/17	1/49	8.592	1	0.003376
7	dbsnp	rs12701985	A	G	ALLELIC	5/17	1/49	8.592	1	0.003376
7	dbsnp	rs1880406	A	C	ALLELIC	5/17	1/49	8.592	1	0.003376
7	dbsnp	rs11768111	A	G	ALLELIC	5/17	1/49	8.592	1	0.003376
7	dbsnp	rs9690458	G	A	ALLELIC	5/17	1/49	8.592	1	0.003376
7	dbsnp	rs11766912	G	A	ALLELIC	5/17	1/49	8.592	1	0.003376
7	dbsnp	rs2620441	A	G	ALLELIC	5/17	1/49	8.592	1	0.003376
7	dbsnp	rs9655634	A	G	ALLELIC	12/10	10/40	8.592	1	0.003376
8	dbsnp	rs7013412	A	C	ALLELIC	5/17	1/49	8.592	1	0.003376
8	dbsnp	rs2705044	C	A	ALLELIC	5/17	1/49	8.592	1	0.003376
8	dbsnp	rs4406440	G	A	ALLELIC	5/17	1/49	8.592	1	0.003376
8	dbsnp	rs7821974	G	A	ALLELIC	5/17	1/49	8.592	1	0.003376
8	dbsnp	rs11136135	A	G	ALLELIC	12/10	10/40	8.592	1	0.003376
8	dbsnp	rs4872709	G	A	ALLELIC	12/10	10/40	8.592	1	0.003376
8	dbsnp	rs16921969	G	A	ALLELIC	5/17	1/49	8.592	1	0.003376
8	dbsnp	rs28572193	A	C	ALLELIC	5/17	1/49	8.592	1	0.003376
8	dbsnp	rs3098726	A	G	ALLELIC	5/17	1/49	8.592	1	0.003376
8	dbsnp	rs1400485	G	A	ALLELIC	5/17	1/49	8.592	1	0.003376
8	dbsnp	rs10112057	A	G	ALLELIC	5/17	1/49	8.592	1	0.003376
8	dbsnp	rs10102835	A	G	ALLELIC	5/17	1/49	8.592	1	0.003376
9	dbsnp	rs10815687	G	A	ALLELIC	5/17	1/49	8.592	1	0.003376
9	dbsnp	rs10976606	G	A	ALLELIC	5/17	1/49	8.592	1	0.003376
9	dbsnp	rs12683447	A	C	ALLELIC	5/17	1/49	8.592	1	0.003376
9	dbsnp	rs10966191	A	G	ALLELIC	5/17	1/49	8.592	1	0.003376
9	dbsnp	rs7031162	A	C	ALLELIC	5/17	1/49	8.592	1	0.003376
9	dbsnp	rs2383733	A	G	ALLELIC	5/17	1/49	8.592	1	0.003376
9	dbsnp	rs10738986	G	A	ALLELIC	12/10	10/40	8.592	1	0.003376
9	dbsnp	rs17180299	G	A	ALLELIC	5/17	1/49	8.592	1	0.003376
9	dbsnp	rs10984492	G	A	ALLELIC	5/17	1/49	8.592	1	0.003376
9	dbsnp	rs1888941	A	G	ALLELIC	5/17	1/49	8.592	1	0.003376
9	dbsnp	rs7020956	A	G	ALLELIC	5/17	1/49	8.592	1	0.003376
10	dbsnp	rs9733630	A	G	ALLELIC	12/10	10/40	8.592	1	0.003376
10	dbsnp	rs17155850	G	A	ALLELIC	5/17	1/49	8.592	1	0.003376
10	dbsnp	rs7896493	A	G	ALLELIC	5/17	1/49	8.592	1	0.003376
10	dbsnp	rs2749592	A	C	ALLELIC	12/10	10/40	8.592	1	0.003376
10	dbsnp	rs268309	A	G	ALLELIC	5/17	1/49	8.592	1	0.003376
10	dbsnp	rs17818833	A	G	ALLELIC	5/17	1/49	8.592	1	0.003376
10	dbsnp	rs1060373	A	G	ALLELIC	5/17	1/49	8.592	1	0.003376
10	dbsnp	rs868589	G	A	ALLELIC	12/10	10/40	8.592	1	0.003376
11	dbsnp	rs11024066	A	G	ALLELIC	12/10	10/40	8.592	1	0.003376
11	dbsnp	rs643902	A	G	ALLELIC	5/17	1/49	8.592	1	0.003376
11	dbsnp	rs1518566	A	G	ALLELIC	5/17	1/49	8.592	1	0.003376
11	dbsnp	rs3181328	A	G	ALLELIC	5/17	1/49	8.592	1	0.003376
11	dbsnp	rs1509729	A	G	ALLELIC	12/10	10/40	8.592	1	0.003376
11	dbsnp	rs11214938	A	G	ALLELIC	5/17	1/49	8.592	1	0.003376
11	dbsnp	rs12224823	A	C	ALLELIC	5/17	1/49	8.592	1	0.003376

12	dbsnp	rs11055018	A	G	ALLELIC	5/17	1/49	8.592	1	0.003376
12	dbsnp	rs11837280	G	A	ALLELIC	12/10	10/40	8.592	1	0.003376
12	dbsnp	rs11045044	A	G	ALLELIC	12/10	10/40	8.592	1	0.003376
12	dbsnp	rs4963701	G	A	ALLELIC	5/17	1/49	8.592	1	0.003376
12	dbsnp	rs3782131	G	A	ALLELIC	5/17	1/49	8.592	1	0.003376
12	dbsnp	rs2408184	A	G	ALLELIC	5/17	1/49	8.592	1	0.003376
12	dbsnp	rs2052603	A	C	ALLELIC	5/17	1/49	8.592	1	0.003376
12	dbsnp	rs11182949	C	A	ALLELIC	5/17	1/49	8.592	1	0.003376
12	dbsnp	rs12809717	A	C	ALLELIC	5/17	1/49	8.592	1	0.003376
12	dbsnp	rs17095872	A	C	ALLELIC	5/17	1/49	8.592	1	0.003376
12	dbsnp	rs342174	A	G	ALLELIC	5/17	1/49	8.592	1	0.003376
12	dbsnp	rs12424237	A	G	ALLELIC	5/17	1/49	8.592	1	0.003376
12	dbsnp	rs11175661	A	G	ALLELIC	5/17	1/49	8.592	1	0.003376
12	dbsnp	rs10219488	A	G	ALLELIC	12/10	10/40	8.592	1	0.003376
12	dbsnp	rs12172780	G	A	ALLELIC	12/10	10/40	8.592	1	0.003376
12	dbsnp	rs12824605	G	A	ALLELIC	12/10	10/40	8.592	1	0.003376
12	dbsnp	rs728084	A	G	ALLELIC	12/10	10/40	8.592	1	0.003376
12	dbsnp	rs2956278	G	A	ALLELIC	12/10	10/40	8.592	1	0.003376
12	dbsnp	rs12826320	A	G	ALLELIC	12/10	10/40	8.592	1	0.003376
12	dbsnp	rs7302806	A	G	ALLELIC	5/17	1/49	8.592	1	0.003376
12	dbsnp	rs12099456	G	A	ALLELIC	5/17	1/49	8.592	1	0.003376
12	dbsnp	rs10732648	G	A	ALLELIC	5/17	1/49	8.592	1	0.003376
12	dbsnp	rs4388939	G	A	ALLELIC	5/17	1/49	8.592	1	0.003376
12	dbsnp	rs11611074	A	G	ALLELIC	5/17	1/49	8.592	1	0.003376
12	dbsnp	rs12303276	A	G	ALLELIC	5/17	1/49	8.592	1	0.003376
12	dbsnp	rs3741571	A	G	ALLELIC	5/17	1/49	8.592	1	0.003376
13	dbsnp	rs9553868	A	G	ALLELIC	5/17	1/49	8.592	1	0.003376
13	dbsnp	rs9534271	A	G	ALLELIC	5/17	1/49	8.592	1	0.003376
13	dbsnp	rs9528250	A	G	ALLELIC	5/17	1/49	8.592	1	0.003376
13	dbsnp	rs4646213	A	G	ALLELIC	5/17	1/49	8.592	1	0.003376
13	dbsnp	rs2225160	G	A	ALLELIC	12/10	10/40	8.592	1	0.003376
13	dbsnp	rs9558203	A	G	ALLELIC	5/17	1/49	8.592	1	0.003376
13	dbsnp	rs7996751	A	G	ALLELIC	5/17	1/49	8.592	1	0.003376
13	dbsnp	rs8001296	A	G	ALLELIC	5/17	1/49	8.592	1	0.003376
13	dbsnp	rs11619183	C	A	ALLELIC	5/17	1/49	8.592	1	0.003376
13	dbsnp	rs7322775	A	C	ALLELIC	5/17	1/49	8.592	1	0.003376
13	dbsnp	rs9514510	G	A	ALLELIC	5/17	1/49	8.592	1	0.003376
13	dbsnp	rs1328249	A	G	ALLELIC	5/17	1/49	8.592	1	0.003376
14	dbsnp	rs2146628	A	G	ALLELIC	5/17	1/49	8.592	1	0.003376
14	dbsnp	rs1951762	A	G	ALLELIC	5/17	1/49	8.592	1	0.003376
14	dbsnp	rs1245395	G	A	ALLELIC	5/17	1/49	8.592	1	0.003376
14	dbsnp	rs4647899	A	T	ALLELIC	12/10	10/40	8.592	1	0.003376
14	dbsnp	rs10484092	G	A	ALLELIC	5/17	1/49	8.592	1	0.003376
14	dbsnp	rs1885397	C	A	ALLELIC	12/10	10/40	8.592	1	0.003376
14	dbsnp	rs12883769	A	G	ALLELIC	5/17	1/49	8.592	1	0.003376
14	dbsnp	rs17120810	A	G	ALLELIC	5/17	1/49	8.592	1	0.003376
14	dbsnp	rs1058903	A	G	ALLELIC	5/17	1/49	8.592	1	0.003376
14	dbsnp	rs4905967	A	C	ALLELIC	12/10	10/40	8.592	1	0.003376

15	dbsnp	rs8041435	A	G	ALLELIC	5/17	1/49	8.592	1	0.003376
15	dbsnp	rs7495265	G	A	ALLELIC	12/10	10/40	8.592	1	0.003376
16	dbsnp	rs12928665	G	A	ALLELIC	12/10	10/40	8.592	1	0.003376
16	dbsnp	rs4783954	C	A	ALLELIC	12/10	10/40	8.592	1	0.003376
16	dbsnp	rs17726834	A	G	ALLELIC	5/17	1/49	8.592	1	0.003376
16	dbsnp	rs10438600	A	C	ALLELIC	12/10	10/40	8.592	1	0.003376
17	dbsnp	rs7223088	A	G	ALLELIC	12/10	10/40	8.592	1	0.003376
17	dbsnp	rs9898180	G	A	ALLELIC	5/17	1/49	8.592	1	0.003376
17	dbsnp	rs16943470	G	A	ALLELIC	5/17	1/49	8.592	1	0.003376
17	dbsnp	rs2270524	A	G	ALLELIC	5/17	1/49	8.592	1	0.003376
17	dbsnp	rs17759657	C	A	ALLELIC	5/17	1/49	8.592	1	0.003376
17	dbsnp	rs10451281	G	A	ALLELIC	5/17	1/49	8.592	1	0.003376
17	dbsnp	rs17688743	G	A	ALLELIC	5/17	1/49	8.592	1	0.003376
18	dbsnp	rs6508378	A	G	ALLELIC	5/17	1/49	8.592	1	0.003376
19	dbsnp	rs10415609	A	G	ALLELIC	5/17	1/49	8.592	1	0.003376
20	dbsnp	rs4814650	G	A	ALLELIC	5/17	1/49	8.592	1	0.003376
20	dbsnp	rs2103985	A	G	ALLELIC	5/17	1/49	8.592	1	0.003376
20	dbsnp	rs2092469	A	G	ALLELIC	5/17	1/49	8.592	1	0.003376
20	dbsnp	rs6088353	A	C	ALLELIC	5/17	1/49	8.592	1	0.003376
20	dbsnp	rs6088358	A	G	ALLELIC	5/17	1/49	8.592	1	0.003376
22	dbsnp	rs130575	G	A	ALLELIC	5/17	1/49	8.592	1	0.003376
22	dbsnp	rs17751502	A	G	ALLELIC	5/17	1/49	8.592	1	0.003376
22	dbsnp	rs738483	A	G	ALLELIC	12/10	10/40	8.592	1	0.003376
22	dbsnp	rs6008618	A	G	ALLELIC	12/10	10/40	8.592	1	0.003376
23	dbsnp	rs7054190	A	C	ALLELIC	5/17	1/49	8.592	1	0.003376
23	dbsnp	rs1880848	A	C	ALLELIC	5/17	1/49	8.592	1	0.003376
23	dbsnp	rs7876357	G	A	ALLELIC	12/10	10/40	8.592	1	0.003376
23	dbsnp	rs7879807	A	C	ALLELIC	5/17	1/49	8.592	1	0.003376
23	dbsnp	rs6528009	G	A	ALLELIC	5/17	1/49	8.592	1	0.003376
23	dbsnp	rs6633421	G	A	ALLELIC	5/17	1/49	8.592	1	0.003376
23	dbsnp	rs6520555	A	C	ALLELIC	12/10	10/40	8.592	1	0.003376
23	dbsnp	rs2223159	G	A	ALLELIC	12/10	10/40	8.592	1	0.003376
23	dbsnp	rs7065630	G	A	ALLELIC	12/10	10/40	8.592	1	0.003376
23	dbsnp	rs4969636	A	G	ALLELIC	12/10	10/40	8.592	1	0.003376
23	dbsnp	rs5912017	A	C	ALLELIC	5/17	1/49	8.592	1	0.003376
23	dbsnp	rs5930285	G	A	ALLELIC	5/17	1/49	8.592	1	0.003376
23	dbsnp	rs12008050	G	A	ALLELIC	5/17	1/49	8.592	1	0.003376
23	dbsnp	rs4828389	G	A	ALLELIC	5/17	29/19	8.579	1	0.003401
1	dbsnp	rs897471	G	A	ALLELIC	1/21	19/31	8.523	1	0.003506
1	dbsnp	rs9436605	C	A	ALLELIC	1/21	19/31	8.523	1	0.003506
2	dbsnp	rs7598642	A	G	ALLELIC	1/21	19/31	8.523	1	0.003506
3	dbsnp	rs11717603	A	C	ALLELIC	1/21	19/31	8.523	1	0.003506
3	dbsnp	rs396426	A	G	ALLELIC	1/21	19/31	8.523	1	0.003506
3	dbsnp	rs6438697	G	A	ALLELIC	1/21	19/31	8.523	1	0.003506
4	dbsnp	rs13134269	G	A	ALLELIC	1/21	19/31	8.523	1	0.003506
4	dbsnp	rs7695069	G	A	ALLELIC	1/21	19/31	8.523	1	0.003506
4	dbsnp	rs12649767	G	A	ALLELIC	1/21	19/31	8.523	1	0.003506
5	dbsnp	rs2950026	G	A	ALLELIC	1/21	19/31	8.523	1	0.003506

6	dbsnp	rs1339898	A	G	ALLELIC	1/21	19/31	8.523	1	0.003506
6	dbsnp	rs3846816	A	G	ALLELIC	1/21	19/31	8.523	1	0.003506
7	dbsnp	rs39359	A	G	ALLELIC	1/21	19/31	8.523	1	0.003506
7	dbsnp	rs39399	A	G	ALLELIC	1/21	19/31	8.523	1	0.003506
7	dbsnp	rs9986961	C	A	ALLELIC	1/21	19/31	8.523	1	0.003506
8	dbsnp	rs9644032	A	C	ALLELIC	1/21	19/31	8.523	1	0.003506
8	dbsnp	rs6472189	G	A	ALLELIC	1/21	19/31	8.523	1	0.003506
9	dbsnp	rs12342106	A	G	ALLELIC	1/21	19/31	8.523	1	0.003506
12	dbsnp	rs7954735	G	A	ALLELIC	1/21	19/31	8.523	1	0.003506
12	dbsnp	rs7487519	G	A	ALLELIC	1/21	19/31	8.523	1	0.003506
12	dbsnp	rs28688596	G	A	ALLELIC	1/21	19/31	8.523	1	0.003506
12	dbsnp	rs4883606	A	G	ALLELIC	1/21	19/31	8.523	1	0.003506
15	dbsnp	rs7167360	C	A	ALLELIC	1/21	19/31	8.523	1	0.003506
17	dbsnp	rs758664	G	A	ALLELIC	1/21	19/31	8.523	1	0.003506
20	dbsnp	rs6066892	G	A	ALLELIC	1/21	19/31	8.523	1	0.003506
1	dbsnp	rs12045323	A	G	ALLELIC	7/15	3/47	8.515	1	0.003522
1	dbsnp	rs1408417	A	G	ALLELIC	7/15	3/47	8.515	1	0.003522
1	dbsnp	rs1342738	G	A	ALLELIC	7/15	3/47	8.515	1	0.003522
1	dbsnp	rs1442500	G	A	ALLELIC	7/15	3/47	8.515	1	0.003522
1	dbsnp	rs565210	G	A	ALLELIC	7/15	3/47	8.515	1	0.003522
1	dbsnp	rs12732891	A	C	ALLELIC	7/15	3/47	8.515	1	0.003522
1	dbsnp	rs10753410	G	A	ALLELIC	7/15	3/47	8.515	1	0.003522
1	dbsnp	rs1915877	A	G	ALLELIC	7/15	3/47	8.515	1	0.003522
2	dbsnp	rs4854272	G	A	ALLELIC	7/15	3/47	8.515	1	0.003522
2	dbsnp	rs10181834	A	G	ALLELIC	7/15	3/47	8.515	1	0.003522
2	dbsnp	rs13420936	A	G	ALLELIC	7/15	3/47	8.515	1	0.003522
2	dbsnp	rs6713911	A	G	ALLELIC	7/15	3/47	8.515	1	0.003522
2	dbsnp	rs6545910	A	G	ALLELIC	7/15	3/47	8.515	1	0.003522
2	dbsnp	rs11674245	A	C	ALLELIC	7/15	3/47	8.515	1	0.003522
2	dbsnp	rs7571700	A	G	ALLELIC	7/15	3/47	8.515	1	0.003522
2	dbsnp	rs4314035	G	A	ALLELIC	7/15	3/47	8.515	1	0.003522
2	dbsnp	rs10497191	A	G	ALLELIC	7/15	3/47	8.515	1	0.003522
2	dbsnp	rs7573365	A	G	ALLELIC	7/15	3/47	8.515	1	0.003522
2	dbsnp	rs917435	G	A	ALLELIC	7/15	3/47	8.515	1	0.003522
3	dbsnp	rs1178495	A	G	ALLELIC	7/15	3/47	8.515	1	0.003522
3	dbsnp	rs1626056	A	G	ALLELIC	7/15	3/47	8.515	1	0.003522
3	dbsnp	rs17033143	A	G	ALLELIC	7/15	3/47	8.515	1	0.003522
3	dbsnp	rs357136	A	G	ALLELIC	7/15	3/47	8.515	1	0.003522
3	dbsnp	rs903055	G	A	ALLELIC	7/15	3/47	8.515	1	0.003522
3	dbsnp	rs17272271	G	A	ALLELIC	7/15	3/47	8.515	1	0.003522
3	dbsnp	rs7623201	A	G	ALLELIC	7/15	3/47	8.515	1	0.003522
3	dbsnp	rs1658347	C	A	ALLELIC	7/15	3/47	8.515	1	0.003522
3	dbsnp	rs2367221	G	A	ALLELIC	7/15	3/47	8.515	1	0.003522
4	dbsnp	rs6855048	A	G	ALLELIC	7/15	3/47	8.515	1	0.003522
4	dbsnp	rs13133023	G	A	ALLELIC	7/15	3/47	8.515	1	0.003522
4	dbsnp	rs1501145	A	G	ALLELIC	7/15	3/47	8.515	1	0.003522
4	dbsnp	rs7683530	A	G	ALLELIC	7/15	3/47	8.515	1	0.003522
4	dbsnp	rs7668684	A	G	ALLELIC	7/15	3/47	8.515	1	0.003522

4	dbsnp	rs7657021	A	G	ALLELIC	7/15	3/47	8.515	1	0.003522
4	dbsnp	rs6856727	C	A	ALLELIC	7/15	3/47	8.515	1	0.003522
4	dbsnp	rs6841013	G	A	ALLELIC	7/15	3/47	8.515	1	0.003522
4	dbsnp	rs3811758	A	G	ALLELIC	7/15	3/47	8.515	1	0.003522
5	dbsnp	rs10069504	A	G	ALLELIC	7/15	3/47	8.515	1	0.003522
5	dbsnp	rs4865953	A	C	ALLELIC	7/15	3/47	8.515	1	0.003522
5	dbsnp	rs11747258	A	G	ALLELIC	7/15	3/47	8.515	1	0.003522
5	dbsnp	rs12521812	A	C	ALLELIC	7/15	3/47	8.515	1	0.003522
5	dbsnp	rs6890785	A	G	ALLELIC	7/15	3/47	8.515	1	0.003522
5	dbsnp	rs6450482	A	G	ALLELIC	7/15	3/47	8.515	1	0.003522
5	dbsnp	rs1876691	A	C	ALLELIC	7/15	3/47	8.515	1	0.003522
5	dbsnp	rs292201	A	C	ALLELIC	7/15	3/47	8.515	1	0.003522
5	dbsnp	rs10059648	A	G	ALLELIC	7/15	3/47	8.515	1	0.003522
5	dbsnp	rs12518735	A	G	ALLELIC	7/15	3/47	8.515	1	0.003522
5	dbsnp	rs10041715	G	A	ALLELIC	7/15	3/47	8.515	1	0.003522
5	dbsnp	rs10045335	G	A	ALLELIC	7/15	3/47	8.515	1	0.003522
5	dbsnp	rs7729239	G	A	ALLELIC	7/15	3/47	8.515	1	0.003522
5	dbsnp	rs7733786	A	G	ALLELIC	7/15	3/47	8.515	1	0.003522
5	dbsnp	rs1560323	A	C	ALLELIC	7/15	3/47	8.515	1	0.003522
5	dbsnp	rs10476724	G	A	ALLELIC	7/15	3/47	8.515	1	0.003522
5	dbsnp	rs1423315	G	A	ALLELIC	7/15	3/47	8.515	1	0.003522
5	dbsnp	rs17534957	A	G	ALLELIC	7/15	3/47	8.515	1	0.003522
5	dbsnp	rs1473134	G	A	ALLELIC	7/15	3/47	8.515	1	0.003522
6	dbsnp	rs11964945	G	A	ALLELIC	7/15	3/47	8.515	1	0.003522
6	dbsnp	rs3887266	A	G	ALLELIC	7/15	3/47	8.515	1	0.003522
6	dbsnp	rs740882	A	G	ALLELIC	7/15	3/47	8.515	1	0.003522
6	dbsnp	rs881284	A	G	ALLELIC	7/15	3/47	8.515	1	0.003522
6	dbsnp	rs29262	G	A	ALLELIC	7/15	3/47	8.515	1	0.003522
6	dbsnp	rs29258	A	G	ALLELIC	7/15	3/47	8.515	1	0.003522
6	dbsnp	rs29257	G	A	ALLELIC	7/15	3/47	8.515	1	0.003522
6	dbsnp	rs29253	G	A	ALLELIC	7/15	3/47	8.515	1	0.003522
6	dbsnp	rs29226	C	A	ALLELIC	7/15	3/47	8.515	1	0.003522
6	dbsnp	rs29225	G	A	ALLELIC	7/15	3/47	8.515	1	0.003522
6	dbsnp	rs6919973	A	G	ALLELIC	7/15	3/47	8.515	1	0.003522
6	dbsnp	rs29223	C	A	ALLELIC	7/15	3/47	8.515	1	0.003522
6	dbsnp	rs2021749	A	G	ALLELIC	7/15	3/47	8.515	1	0.003522
6	dbsnp	rs9366754	G	C	ALLELIC	7/15	3/47	8.515	1	0.003522
6	dbsnp	rs28730510	C	G	ALLELIC	7/15	3/47	8.515	1	0.003522
6	dbsnp	rs2094596	A	G	ALLELIC	7/15	3/47	8.515	1	0.003522
6	dbsnp	rs9372328	A	G	ALLELIC	7/15	3/47	8.515	1	0.003522
6	dbsnp	rs9401098	G	A	ALLELIC	7/15	3/47	8.515	1	0.003522
6	dbsnp	rs9493891	A	G	ALLELIC	7/15	3/47	8.515	1	0.003522
7	dbsnp	rs2159017	A	C	ALLELIC	7/15	3/47	8.515	1	0.003522
7	dbsnp	rs2189052	G	A	ALLELIC	7/15	3/47	8.515	1	0.003522
7	dbsnp	rs12706565	G	A	ALLELIC	7/15	3/47	8.515	1	0.003522
8	dbsnp	rs10503972	G	A	ALLELIC	7/15	3/47	8.515	1	0.003522
8	dbsnp	rs17380635	A	C	ALLELIC	7/15	3/47	8.515	1	0.003522
9	dbsnp	rs7868009	A	G	ALLELIC	7/15	3/47	8.515	1	0.003522

9	dbsnp	rs10283761	G	A	ALLELIC	7/15	3/47	8.515	1	0.003522
9	dbsnp	rs11789781	G	A	ALLELIC	7/15	3/47	8.515	1	0.003522
10	dbsnp	rs10508307	A	G	ALLELIC	7/15	3/47	8.515	1	0.003522
10	dbsnp	rs1610163	A	G	ALLELIC	7/15	3/47	8.515	1	0.003522
10	dbsnp	rs12240404	A	G	ALLELIC	7/15	3/47	8.515	1	0.003522
10	dbsnp	rs10826802	A	G	ALLELIC	7/15	3/47	8.515	1	0.003522
10	dbsnp	rs1878166	G	A	ALLELIC	7/15	3/47	8.515	1	0.003522
10	dbsnp	rs12265793	G	A	ALLELIC	7/15	3/47	8.515	1	0.003522
11	dbsnp	rs3763820	A	G	ALLELIC	7/15	3/47	8.515	1	0.003522
11	dbsnp	rs12575607	A	G	ALLELIC	7/15	3/47	8.515	1	0.003522
11	dbsnp	rs589289	A	G	ALLELIC	7/15	3/47	8.515	1	0.003522
11	dbsnp	rs10897545	G	A	ALLELIC	7/15	3/47	8.515	1	0.003522
11	dbsnp	rs4753437	A	C	ALLELIC	7/15	3/47	8.515	1	0.003522
11	dbsnp	rs2511380	C	A	ALLELIC	7/15	3/47	8.515	1	0.003522
11	dbsnp	rs2511403	G	A	ALLELIC	7/15	3/47	8.515	1	0.003522
12	dbsnp	rs10492090	A	G	ALLELIC	7/15	3/47	8.515	1	0.003522
12	dbsnp	rs2350663	G	A	ALLELIC	7/15	3/47	8.515	1	0.003522
12	dbsnp	rs12230192	C	A	ALLELIC	7/15	3/47	8.515	1	0.003522
12	dbsnp	rs11177364	G	A	ALLELIC	7/15	3/47	8.515	1	0.003522
12	dbsnp	rs11177624	A	G	ALLELIC	7/15	3/47	8.515	1	0.003522
12	dbsnp	rs2522276	G	A	ALLELIC	7/15	3/47	8.515	1	0.003522
12	dbsnp	rs775451	G	A	ALLELIC	7/15	3/47	8.515	1	0.003522
12	dbsnp	rs6539482	A	G	ALLELIC	7/15	3/47	8.515	1	0.003522
12	dbsnp	rs17807835	G	A	ALLELIC	7/15	3/47	8.515	1	0.003522
12	dbsnp	rs17811547	A	G	ALLELIC	7/15	3/47	8.515	1	0.003522
14	dbsnp	rs6575049	A	G	ALLELIC	7/15	3/47	8.515	1	0.003522
15	dbsnp	rs7173271	A	G	ALLELIC	7/15	3/47	8.515	1	0.003522
15	dbsnp	rs11853179	A	G	ALLELIC	7/15	3/47	8.515	1	0.003522
15	dbsnp	rs4594240	G	A	ALLELIC	7/15	3/47	8.515	1	0.003522
16	dbsnp	rs1557811	G	A	ALLELIC	7/15	3/47	8.515	1	0.003522
16	dbsnp	rs1008154	A	G	ALLELIC	7/15	3/47	8.515	1	0.003522
16	dbsnp	rs11865882	A	G	ALLELIC	7/15	3/47	8.515	1	0.003522
16	dbsnp	rs7350878	G	A	ALLELIC	7/15	3/47	8.515	1	0.003522
17	dbsnp	rs11870140	G	A	ALLELIC	7/15	3/47	8.515	1	0.003522
17	dbsnp	rs8070414	C	A	ALLELIC	7/15	3/47	8.515	1	0.003522
17	dbsnp	rs4889911	A	G	ALLELIC	7/15	3/47	8.515	1	0.003522
19	dbsnp	rs1842221	A	C	ALLELIC	7/15	3/47	8.515	1	0.003522
19	dbsnp	rs17601885	G	A	ALLELIC	7/15	3/47	8.515	1	0.003522
20	dbsnp	rs6077914	G	A	ALLELIC	7/15	3/47	8.515	1	0.003522
20	dbsnp	rs1049679	G	A	ALLELIC	7/15	3/47	8.515	1	0.003522
20	dbsnp	rs6125888	C	A	ALLELIC	7/15	3/47	8.515	1	0.003522
20	dbsnp	rs2073053	A	C	ALLELIC	7/15	3/47	8.515	1	0.003522
20	dbsnp	rs4811253	A	G	ALLELIC	7/15	3/47	8.515	1	0.003522
23	dbsnp	rs7061250	G	A	ALLELIC	7/15	3/47	8.515	1	0.003522
23	dbsnp	rs11796927	G	A	ALLELIC	7/15	3/47	8.515	1	0.003522
23	dbsnp	rs1462578	A	G	ALLELIC	7/15	3/47	8.515	1	0.003522
23	dbsnp	rs5929798	G	A	ALLELIC	7/15	3/47	8.515	1	0.003522
23	dbsnp	rs7053295	A	G	ALLELIC	7/15	3/47	8.515	1	0.003522

23	dbsnp	rs4829864	G	A	ALLELIC	7/15	3/47	8.515	1	0.003522
23	dbsnp	rs5931124	A	G	ALLELIC	7/15	3/47	8.515	1	0.003522
23	dbsnp	rs11095691	G	A	ALLELIC	7/15	3/47	8.515	1	0.003522
23	dbsnp	rs5931135	G	A	ALLELIC	7/15	3/47	8.515	1	0.003522
1	dbsnp	rs3935570	A	C	ALLELIC	8/12	5/45	8.502	1	0.003548
1	dbsnp	rs693260	A	G	ALLELIC	8/12	5/45	8.502	1	0.003548
1	dbsnp	rs699521	G	A	ALLELIC	8/12	5/45	8.502	1	0.003548
1	dbsnp	rs12058868	A	G	ALLELIC	8/12	5/45	8.502	1	0.003548
2	dbsnp	rs1838899	A	G	ALLELIC	8/12	5/45	8.502	1	0.003548
3	dbsnp	rs2067819	A	G	ALLELIC	8/12	5/45	8.502	1	0.003548
5	dbsnp	rs298380	G	A	ALLELIC	8/12	5/45	8.502	1	0.003548
5	dbsnp	rs298372	G	A	ALLELIC	8/12	5/45	8.502	1	0.003548
5	dbsnp	rs10068957	G	A	ALLELIC	8/12	5/45	8.502	1	0.003548
5	dbsnp	rs13165241	G	A	ALLELIC	8/12	5/45	8.502	1	0.003548
6	dbsnp	rs4708042	G	A	ALLELIC	8/12	5/45	8.502	1	0.003548
8	dbsnp	rs932267	A	G	ALLELIC	8/12	5/45	8.502	1	0.003548
8	dbsnp	rs7014419	A	C	ALLELIC	8/12	5/45	8.502	1	0.003548
10	dbsnp	rs11191820	G	A	ALLELIC	8/12	5/45	8.502	1	0.003548
10	dbsnp	rs17693936	G	A	ALLELIC	8/12	5/45	8.502	1	0.003548
11	dbsnp	rs16910187	G	A	ALLELIC	8/12	5/45	8.502	1	0.003548
14	dbsnp	rs217671	G	A	ALLELIC	8/12	5/45	8.502	1	0.003548
14	dbsnp	rs10141319	G	A	ALLELIC	8/12	5/45	8.502	1	0.003548
16	dbsnp	rs1585528	G	A	ALLELIC	8/12	5/45	8.502	1	0.003548
20	dbsnp	rs6030456	A	G	ALLELIC	8/12	5/45	8.502	1	0.003548
23	dbsnp	rs5990415	A	G	ALLELIC	8/12	5/45	8.502	1	0.003548
3	dbsnp	rs4510355	A	G	ALLELIC	3/19	25/25	8.501	1	0.00355
3	dbsnp	rs4073897	G	A	ALLELIC	3/19	25/25	8.501	1	0.00355
3	dbsnp	rs4683428	A	G	ALLELIC	3/19	25/25	8.501	1	0.00355
4	dbsnp	rs6446700	G	A	ALLELIC	3/19	25/25	8.501	1	0.00355
4	dbsnp	rs6836081	A	G	ALLELIC	3/19	25/25	8.501	1	0.00355
5	dbsnp	rs877353	C	A	ALLELIC	3/19	25/25	8.501	1	0.00355
5	dbsnp	rs1349687	A	G	ALLELIC	3/19	25/25	8.501	1	0.00355
7	dbsnp	rs9641615	A	G	ALLELIC	3/19	25/25	8.501	1	0.00355
8	dbsnp	rs891570	G	A	ALLELIC	3/19	25/25	8.501	1	0.00355
9	dbsnp	rs1889817	G	A	ALLELIC	3/19	25/25	8.501	1	0.00355
14	dbsnp	rs1889802	A	G	ALLELIC	3/19	25/25	8.501	1	0.00355
14	dbsnp	rs3742665	A	G	ALLELIC	3/19	25/25	8.501	1	0.00355
16	dbsnp	rs1559393	G	A	ALLELIC	3/19	25/25	8.501	1	0.00355
18	dbsnp	rs2957128	A	G	ALLELIC	3/19	25/25	8.501	1	0.00355
20	dbsnp	rs6040477	A	G	ALLELIC	3/19	25/25	8.501	1	0.00355
23	dbsnp	rs2694727	A	G	ALLELIC	3/19	25/25	8.501	1	0.00355
23	dbsnp	rs7881435	A	G	ALLELIC	3/19	25/25	8.501	1	0.00355
1	dbsnp	rs1418808	G	A	ALLELIC	5/17	30/20	8.496	1	0.003558
1	dbsnp	rs2686240	G	A	ALLELIC	5/17	30/20	8.496	1	0.003558
2	dbsnp	rs1517682	A	G	ALLELIC	5/17	30/20	8.496	1	0.003558
2	dbsnp	rs10930407	A	G	ALLELIC	17/5	20/30	8.496	1	0.003558
2	dbsnp	rs10176594	G	A	ALLELIC	5/17	30/20	8.496	1	0.003558
3	dbsnp	rs11710715	G	A	ALLELIC	5/17	30/20	8.496	1	0.003558

3	dbsnp	rs4678953	G	A	ALLELIC	5/17	30/20	8.496	1	0.003558
4	dbsnp	rs768695	A	G	ALLELIC	5/17	30/20	8.496	1	0.003558
4	dbsnp	rs282706	G	A	ALLELIC	17/5	20/30	8.496	1	0.003558
4	dbsnp	rs978402	G	A	ALLELIC	5/17	30/20	8.496	1	0.003558
5	dbsnp	rs3117740	G	A	ALLELIC	5/17	30/20	8.496	1	0.003558
7	dbsnp	rs2107888	A	G	ALLELIC	17/5	20/30	8.496	1	0.003558
7	dbsnp	rs10231911	G	A	ALLELIC	17/5	20/30	8.496	1	0.003558
9	dbsnp	rs1416505	A	G	ALLELIC	5/17	30/20	8.496	1	0.003558
9	dbsnp	rs620420	G	A	ALLELIC	5/17	30/20	8.496	1	0.003558
9	dbsnp	rs665889	A	G	ALLELIC	5/17	30/20	8.496	1	0.003558
10	dbsnp	rs4917787	A	C	ALLELIC	5/17	30/20	8.496	1	0.003558
11	dbsnp	rs2511798	C	A	ALLELIC	17/5	20/30	8.496	1	0.003558
11	dbsnp	rs495997	G	A	ALLELIC	17/5	20/30	8.496	1	0.003558
12	dbsnp	rs1148427	A	G	ALLELIC	17/5	20/30	8.496	1	0.003558
13	dbsnp	rs1198237	G	A	ALLELIC	5/17	30/20	8.496	1	0.003558
13	dbsnp	rs12867753	A	G	ALLELIC	5/17	30/20	8.496	1	0.003558
15	dbsnp	rs4776862	G	A	ALLELIC	5/17	30/20	8.496	1	0.003558
16	dbsnp	rs2745137	A	G	ALLELIC	5/17	30/20	8.496	1	0.003558
16	dbsnp	rs1054645	G	A	ALLELIC	5/17	30/20	8.496	1	0.003558
17	dbsnp	rs6505375	A	G	ALLELIC	5/17	30/20	8.496	1	0.003558
18	dbsnp	rs12959039	A	G	ALLELIC	17/5	20/30	8.496	1	0.003558
18	dbsnp	rs2438415	A	G	ALLELIC	5/17	30/20	8.496	1	0.003558
20	dbsnp	rs2424794	A	G	ALLELIC	5/17	30/20	8.496	1	0.003558
20	dbsnp	rs6141536	A	G	ALLELIC	17/5	20/30	8.496	1	0.003558
20	dbsnp	rs6088901	G	A	ALLELIC	17/5	20/30	8.496	1	0.003558
20	dbsnp	rs1018405	A	G	ALLELIC	17/5	20/30	8.496	1	0.003558
21	dbsnp	rs2837507	A	G	ALLELIC	17/5	20/30	8.496	1	0.003558
21	dbsnp	rs2837518	G	A	ALLELIC	17/5	20/30	8.496	1	0.003558
22	dbsnp	rs5750348	A	G	ALLELIC	5/17	30/20	8.496	1	0.003558
23	dbsnp	rs331347	A	G	ALLELIC	5/17	30/20	8.496	1	0.003558
23	dbsnp	rs1448865	G	A	ALLELIC	5/17	30/20	8.496	1	0.003558
2	dbsnp	rs6431885	G	A	ALLELIC	14/6	16/34	8.423	1	0.003704
2	dbsnp	rs1542665	G	A	ALLELIC	14/6	16/34	8.423	1	0.003704
2	dbsnp	rs571111	G	A	ALLELIC	14/6	16/34	8.423	1	0.003704
2	dbsnp	rs6728027	G	A	ALLELIC	14/6	16/34	8.423	1	0.003704
2	dbsnp	rs7590429	A	G	ALLELIC	14/6	16/34	8.423	1	0.003704
4	dbsnp	rs6847750	A	G	ALLELIC	14/6	16/34	8.423	1	0.003704
7	dbsnp	rs10267433	G	A	ALLELIC	14/6	16/34	8.423	1	0.003704
7	dbsnp	rs9692321	G	A	ALLELIC	14/6	16/34	8.423	1	0.003704
9	dbsnp	rs10816927	C	A	ALLELIC	14/6	16/34	8.423	1	0.003704
10	dbsnp	rs7895398	G	A	ALLELIC	14/6	16/34	8.423	1	0.003704
11	dbsnp	rs1599459	G	A	ALLELIC	14/6	16/34	8.423	1	0.003704
12	dbsnp	rs1554986	A	G	ALLELIC	14/6	16/34	8.423	1	0.003704
12	dbsnp	rs1973869	A	C	ALLELIC	14/6	16/34	8.423	1	0.003704
14	dbsnp	rs8008072	C	A	ALLELIC	14/6	16/34	8.423	1	0.003704
14	dbsnp	rs6574294	C	A	ALLELIC	14/6	16/34	8.423	1	0.003704
15	dbsnp	rs1908196	G	A	ALLELIC	14/6	16/34	8.423	1	0.003704
18	dbsnp	rs11151610	G	A	ALLELIC	14/6	16/34	8.423	1	0.003704

1	dbsnp	rs6605081	A	G	ALLELIC	10/12	7/43	8.381	1	0.003792
1	dbsnp	rs6695900	G	A	ALLELIC	10/12	7/43	8.381	1	0.003792
1	dbsnp	rs1008495	A	C	ALLELIC	10/12	7/43	8.381	1	0.003792
1	dbsnp	rs6658545	G	A	ALLELIC	10/12	7/43	8.381	1	0.003792
1	dbsnp	rs1053093	A	G	ALLELIC	10/12	7/43	8.381	1	0.003792
1	dbsnp	rs6684332	A	G	ALLELIC	10/12	7/43	8.381	1	0.003792
1	dbsnp	rs2200122	A	C	ALLELIC	10/12	7/43	8.381	1	0.003792
2	dbsnp	rs16858314	G	A	ALLELIC	10/12	7/43	8.381	1	0.003792
2	dbsnp	rs10432425	A	G	ALLELIC	10/12	7/43	8.381	1	0.003792
2	dbsnp	rs1439235	A	G	ALLELIC	10/12	7/43	8.381	1	0.003792
2	dbsnp	rs13028597	G	A	ALLELIC	10/12	7/43	8.381	1	0.003792
3	dbsnp	rs4858483	A	C	ALLELIC	10/12	7/43	8.381	1	0.003792
3	dbsnp	rs6550748	A	G	ALLELIC	10/12	7/43	8.381	1	0.003792
3	dbsnp	rs1562487	A	C	ALLELIC	10/12	7/43	8.381	1	0.003792
3	dbsnp	rs12696497	G	A	ALLELIC	10/12	7/43	8.381	1	0.003792
3	dbsnp	rs10937103	G	A	ALLELIC	10/12	7/43	8.381	1	0.003792
3	dbsnp	rs11710878	G	A	ALLELIC	10/12	7/43	8.381	1	0.003792
4	dbsnp	rs10805281	G	A	ALLELIC	10/12	7/43	8.381	1	0.003792
4	dbsnp	rs2585909	G	A	ALLELIC	10/12	7/43	8.381	1	0.003792
4	dbsnp	rs1295450	A	G	ALLELIC	10/12	7/43	8.381	1	0.003792
4	dbsnp	rs17048339	G	A	ALLELIC	10/12	7/43	8.381	1	0.003792
5	dbsnp	rs3810859	A	G	ALLELIC	10/12	7/43	8.381	1	0.003792
5	dbsnp	rs1388120	A	G	ALLELIC	10/12	7/43	8.381	1	0.003792
5	dbsnp	rs10038754	G	A	ALLELIC	10/12	7/43	8.381	1	0.003792
5	dbsnp	rs6556145	G	A	ALLELIC	10/12	7/43	8.381	1	0.003792
5	dbsnp	rs4704749	G	A	ALLELIC	10/12	7/43	8.381	1	0.003792
5	dbsnp	rs1348523	G	A	ALLELIC	10/12	7/43	8.381	1	0.003792
6	dbsnp	rs2050289	A	G	ALLELIC	10/12	7/43	8.381	1	0.003792
6	dbsnp	rs29230	G	A	ALLELIC	10/12	7/43	8.381	1	0.003792
6	dbsnp	rs2239705	A	G	ALLELIC	10/12	7/43	8.381	1	0.003792
6	dbsnp	rs493768	A	G	ALLELIC	10/12	7/43	8.381	1	0.003792
6	dbsnp	rs4706061	A	G	ALLELIC	10/12	7/43	8.381	1	0.003792
6	dbsnp	rs1403926	A	C	ALLELIC	10/12	7/43	8.381	1	0.003792
6	dbsnp	rs11961866	G	A	ALLELIC	10/12	7/43	8.381	1	0.003792
6	dbsnp	rs9373237	C	A	ALLELIC	10/12	7/43	8.381	1	0.003792
7	dbsnp	rs11770305	G	A	ALLELIC	10/12	7/43	8.381	1	0.003792
7	dbsnp	rs6955135	A	C	ALLELIC	10/12	7/43	8.381	1	0.003792
7	dbsnp	rs1897815	A	G	ALLELIC	10/12	7/43	8.381	1	0.003792
7	dbsnp	rs13232363	A	G	ALLELIC	10/12	7/43	8.381	1	0.003792
8	dbsnp	rs11784870	A	G	ALLELIC	10/12	7/43	8.381	1	0.003792
8	dbsnp	rs6471171	A	C	ALLELIC	10/12	7/43	8.381	1	0.003792
9	dbsnp	rs10756003	A	G	ALLELIC	10/12	7/43	8.381	1	0.003792
9	dbsnp	rs4878112	A	G	ALLELIC	10/12	7/43	8.381	1	0.003792
9	dbsnp	rs2472495	G	A	ALLELIC	10/12	7/43	8.381	1	0.003792
10	dbsnp	rs11598477	A	G	ALLELIC	10/12	7/43	8.381	1	0.003792
10	dbsnp	rs1360117	G	A	ALLELIC	10/12	7/43	8.381	1	0.003792
10	dbsnp	rs11189378	A	G	ALLELIC	10/12	7/43	8.381	1	0.003792
11	dbsnp	rs2755145	A	G	ALLELIC	10/12	7/43	8.381	1	0.003792

11	dbsnp	rs489412	A	G	ALLELIC	10/12	7/43	8.381	1	0.003792
11	dbsnp	rs10501996	G	A	ALLELIC	10/12	7/43	8.381	1	0.003792
11	dbsnp	rs4938344	G	A	ALLELIC	10/12	7/43	8.381	1	0.003792
11	dbsnp	rs10892079	A	C	ALLELIC	10/12	7/43	8.381	1	0.003792
11	dbsnp	rs356269	G	A	ALLELIC	10/12	7/43	8.381	1	0.003792
11	dbsnp	rs7108112	A	G	ALLELIC	10/12	7/43	8.381	1	0.003792
12	dbsnp	rs2728571	G	A	ALLELIC	10/12	7/43	8.381	1	0.003792
12	dbsnp	rs2417833	G	A	ALLELIC	10/12	7/43	8.381	1	0.003792
12	dbsnp	rs2728565	G	A	ALLELIC	10/12	7/43	8.381	1	0.003792
12	dbsnp	rs1663470	C	A	ALLELIC	10/12	7/43	8.381	1	0.003792
12	dbsnp	rs1012225	C	A	ALLELIC	10/12	7/43	8.381	1	0.003792
13	dbsnp	rs765606	A	G	ALLELIC	10/12	7/43	8.381	1	0.003792
13	dbsnp	rs12584527	A	G	ALLELIC	10/12	7/43	8.381	1	0.003792
13	dbsnp	rs9566162	A	G	ALLELIC	10/12	7/43	8.381	1	0.003792
13	dbsnp	rs1407976	A	G	ALLELIC	10/12	7/43	8.381	1	0.003792
13	dbsnp	rs9572286	A	G	ALLELIC	10/12	7/43	8.381	1	0.003792
13	dbsnp	rs4483719	A	G	ALLELIC	10/12	7/43	8.381	1	0.003792
17	dbsnp	rs12603906	A	G	ALLELIC	10/12	7/43	8.381	1	0.003792
17	dbsnp	rs11079020	A	G	ALLELIC	10/12	7/43	8.381	1	0.003792
17	dbsnp	rs1003313	A	G	ALLELIC	10/12	7/43	8.381	1	0.003792
17	dbsnp	rs2620052	G	A	ALLELIC	10/12	7/43	8.381	1	0.003792
18	dbsnp	rs9949696	A	G	ALLELIC	10/12	7/43	8.381	1	0.003792
18	dbsnp	rs6417086	G	A	ALLELIC	10/12	7/43	8.381	1	0.003792
18	dbsnp	rs6507662	G	A	ALLELIC	10/12	7/43	8.381	1	0.003792
20	dbsnp	rs6087091	A	G	ALLELIC	10/12	7/43	8.381	1	0.003792
20	dbsnp	rs6118268	C	A	ALLELIC	10/12	7/43	8.381	1	0.003792
20	dbsnp	rs735050	G	A	ALLELIC	10/12	7/43	8.381	1	0.003792
22	dbsnp	rs5995155	A	G	ALLELIC	10/12	7/43	8.381	1	0.003792
22	dbsnp	rs139096	A	G	ALLELIC	10/12	7/43	8.381	1	0.003792
23	dbsnp	rs221396	C	A	ALLELIC	10/12	7/43	8.381	1	0.003792
23	dbsnp	rs6616323	G	A	ALLELIC	10/12	7/43	8.381	1	0.003792
23	dbsnp	rs2194898	G	A	ALLELIC	10/12	7/43	8.381	1	0.003792
1	dbsnp	rs9328948	A	C	ALLELIC	6/16	2/48	8.378	1	0.003798
1	dbsnp	rs509261	A	G	ALLELIC	6/16	2/48	8.378	1	0.003798
1	dbsnp	rs6685296	G	A	ALLELIC	6/16	2/48	8.378	1	0.003798
1	dbsnp	rs11579575	A	C	ALLELIC	6/16	2/48	8.378	1	0.003798
1	dbsnp	rs12409558	A	C	ALLELIC	6/16	2/48	8.378	1	0.003798
1	dbsnp	rs509414	C	A	ALLELIC	6/16	2/48	8.378	1	0.003798
1	dbsnp	rs3753661	C	A	ALLELIC	6/16	2/48	8.378	1	0.003798
1	dbsnp	rs3766934	A	C	ALLELIC	6/16	2/48	8.378	1	0.003798
1	dbsnp	rs3738043	G	A	ALLELIC	6/16	2/48	8.378	1	0.003798
2	dbsnp	rs219542	C	A	ALLELIC	6/16	2/48	8.378	1	0.003798
2	dbsnp	rs6736937	G	A	ALLELIC	6/16	2/48	8.378	1	0.003798
2	dbsnp	rs11680307	A	G	ALLELIC	2/20	22/28	8.378	1	0.003798
2	dbsnp	rs772788	A	G	ALLELIC	2/20	22/28	8.378	1	0.003798
3	dbsnp	rs6776332	G	A	ALLELIC	2/20	22/28	8.378	1	0.003798
3	dbsnp	rs17036160	A	G	ALLELIC	6/16	2/48	8.378	1	0.003798
3	dbsnp	rs13064760	A	G	ALLELIC	6/16	2/48	8.378	1	0.003798

3	dbsnp	rs6793666	A	G	ALLELIC	6/16	2/48	8.378	1	0.003798
3	dbsnp	rs3774487	G	A	ALLELIC	6/16	2/48	8.378	1	0.003798
3	dbsnp	rs9880768	A	G	ALLELIC	6/16	2/48	8.378	1	0.003798
3	dbsnp	rs646315	A	C	ALLELIC	6/16	2/48	8.378	1	0.003798
3	dbsnp	rs875870	G	A	ALLELIC	6/16	2/48	8.378	1	0.003798
3	dbsnp	rs976052	A	G	ALLELIC	6/16	2/48	8.378	1	0.003798
3	dbsnp	rs6444980	G	A	ALLELIC	2/20	22/28	8.378	1	0.003798
4	dbsnp	rs1400773	A	G	ALLELIC	6/16	2/48	8.378	1	0.003798
4	dbsnp	rs11946196	A	G	ALLELIC	6/16	2/48	8.378	1	0.003798
4	dbsnp	rs6851075	G	A	ALLELIC	2/20	22/28	8.378	1	0.003798
4	dbsnp	rs1898848	A	G	ALLELIC	6/16	2/48	8.378	1	0.003798
4	dbsnp	rs1439294	C	A	ALLELIC	6/16	2/48	8.378	1	0.003798
4	dbsnp	rs4535361	A	G	ALLELIC	6/16	2/48	8.378	1	0.003798
4	dbsnp	rs13132177	G	A	ALLELIC	6/16	2/48	8.378	1	0.003798
4	dbsnp	rs11725332	G	A	ALLELIC	6/16	2/48	8.378	1	0.003798
4	dbsnp	rs2625247	A	G	ALLELIC	6/16	2/48	8.378	1	0.003798
4	dbsnp	rs7659108	A	G	ALLELIC	6/16	2/48	8.378	1	0.003798
5	dbsnp	rs1106136	A	C	ALLELIC	6/16	2/48	8.378	1	0.003798
5	dbsnp	rs6878043	C	A	ALLELIC	6/16	2/48	8.378	1	0.003798
5	dbsnp	rs461425	G	A	ALLELIC	6/16	2/48	8.378	1	0.003798
5	dbsnp	rs10062244	A	G	ALLELIC	6/16	2/48	8.378	1	0.003798
5	dbsnp	rs10474277	G	A	ALLELIC	6/16	2/48	8.378	1	0.003798
5	dbsnp	rs36791	G	A	ALLELIC	6/16	2/48	8.378	1	0.003798
5	dbsnp	rs1494826	A	G	ALLELIC	6/16	2/48	8.378	1	0.003798
5	dbsnp	rs10052236	C	A	ALLELIC	6/16	2/48	8.378	1	0.003798
5	dbsnp	rs3797851	A	C	ALLELIC	6/16	2/48	8.378	1	0.003798
5	dbsnp	rs9007	G	A	ALLELIC	6/16	2/48	8.378	1	0.003798
5	dbsnp	rs10515861	G	A	ALLELIC	2/20	22/28	8.378	1	0.003798
5	dbsnp	rs279416	A	C	ALLELIC	2/20	22/28	8.378	1	0.003798
5	dbsnp	rs17738444	G	A	ALLELIC	6/16	2/48	8.378	1	0.003798
5	dbsnp	rs11249632	A	G	ALLELIC	6/16	2/48	8.378	1	0.003798
6	dbsnp	rs12665228	A	G	ALLELIC	6/16	2/48	8.378	1	0.003798
6	dbsnp	rs9381042	A	G	ALLELIC	6/16	2/48	8.378	1	0.003798
6	dbsnp	rs911982	C	A	ALLELIC	6/16	2/48	8.378	1	0.003798
6	dbsnp	rs1155750	A	G	ALLELIC	6/16	2/48	8.378	1	0.003798
6	dbsnp	rs10943983	A	G	ALLELIC	6/16	2/48	8.378	1	0.003798
6	dbsnp	rs17074746	G	A	ALLELIC	6/16	2/48	8.378	1	0.003798
6	dbsnp	rs1934226	A	G	ALLELIC	6/16	2/48	8.378	1	0.003798
6	dbsnp	rs17182988	A	G	ALLELIC	6/16	2/48	8.378	1	0.003798
7	dbsnp	rs6967345	G	A	ALLELIC	2/20	22/28	8.378	1	0.003798
7	dbsnp	rs10085751	A	G	ALLELIC	2/20	22/28	8.378	1	0.003798
7	dbsnp	rs11980921	G	A	ALLELIC	2/20	22/28	8.378	1	0.003798
7	dbsnp	rs10265155	G	A	ALLELIC	2/20	22/28	8.378	1	0.003798
7	dbsnp	rs17641278	A	G	ALLELIC	6/16	2/48	8.378	1	0.003798
8	dbsnp	rs10505301	A	G	ALLELIC	6/16	2/48	8.378	1	0.003798
9	dbsnp	rs1398433	G	A	ALLELIC	2/20	22/28	8.378	1	0.003798
9	dbsnp	rs17606608	A	G	ALLELIC	6/16	2/48	8.378	1	0.003798
9	dbsnp	rs4372056	G	A	ALLELIC	6/16	2/48	8.378	1	0.003798

9	dbsnp	rs4977816	A	C	ALLELIC	2/20	22/28	8.378	1	0.003798
9	dbsnp	rs1668962	A	G	ALLELIC	6/16	2/48	8.378	1	0.003798
9	dbsnp	rs12336869	A	G	ALLELIC	6/16	2/48	8.378	1	0.003798
10	dbsnp	rs2386709	C	A	ALLELIC	6/16	2/48	8.378	1	0.003798
10	dbsnp	rs703256	G	A	ALLELIC	2/20	22/28	8.378	1	0.003798
10	dbsnp	rs983086	A	C	ALLELIC	2/20	22/28	8.378	1	0.003798
10	dbsnp	rs2289964	A	G	ALLELIC	6/16	2/48	8.378	1	0.003798
10	dbsnp	rs1278272	G	A	ALLELIC	6/16	2/48	8.378	1	0.003798
10	dbsnp	rs2944462	A	G	ALLELIC	6/16	2/48	8.378	1	0.003798
11	dbsnp	rs389646	A	G	ALLELIC	6/16	2/48	8.378	1	0.003798
11	dbsnp	rs451443	A	G	ALLELIC	6/16	2/48	8.378	1	0.003798
11	dbsnp	rs16924294	G	A	ALLELIC	6/16	2/48	8.378	1	0.003798
11	dbsnp	rs16927077	A	G	ALLELIC	6/16	2/48	8.378	1	0.003798
11	dbsnp	rs3741364	A	G	ALLELIC	6/16	2/48	8.378	1	0.003798
11	dbsnp	rs1509714	A	G	ALLELIC	6/16	2/48	8.378	1	0.003798
11	dbsnp	rs11217175	A	G	ALLELIC	6/16	2/48	8.378	1	0.003798
12	dbsnp	rs35006259	A	G	ALLELIC	6/16	2/48	8.378	1	0.003798
12	dbsnp	rs710708	A	G	ALLELIC	6/16	2/48	8.378	1	0.003798
12	dbsnp	rs766256	G	A	ALLELIC	6/16	2/48	8.378	1	0.003798
12	dbsnp	rs2491343	A	C	ALLELIC	6/16	2/48	8.378	1	0.003798
12	dbsnp	rs7303464	C	A	ALLELIC	6/16	2/48	8.378	1	0.003798
12	dbsnp	rs2204743	A	G	ALLELIC	6/16	2/48	8.378	1	0.003798
12	dbsnp	rs11104794	A	G	ALLELIC	6/16	2/48	8.378	1	0.003798
12	dbsnp	rs1426463	G	A	ALLELIC	6/16	2/48	8.378	1	0.003798
12	dbsnp	rs11067544	A	G	ALLELIC	6/16	2/48	8.378	1	0.003798
12	dbsnp	rs1077410	G	A	ALLELIC	6/16	2/48	8.378	1	0.003798
13	dbsnp	rs11148350	A	G	ALLELIC	6/16	2/48	8.378	1	0.003798
13	dbsnp	rs4771007	A	G	ALLELIC	6/16	2/48	8.378	1	0.003798
13	dbsnp	rs674286	A	G	ALLELIC	6/16	2/48	8.378	1	0.003798
13	dbsnp	rs7336267	A	C	ALLELIC	6/16	2/48	8.378	1	0.003798
13	dbsnp	rs7989245	G	A	ALLELIC	2/20	22/28	8.378	1	0.003798
13	dbsnp	rs9593571	G	A	ALLELIC	2/20	22/28	8.378	1	0.003798
13	dbsnp	rs8001225	C	A	ALLELIC	2/20	22/28	8.378	1	0.003798
13	dbsnp	rs1936004	A	C	ALLELIC	2/20	22/28	8.378	1	0.003798
13	dbsnp	rs9531258	G	A	ALLELIC	2/20	22/28	8.378	1	0.003798
13	dbsnp	rs301678	A	C	ALLELIC	6/16	2/48	8.378	1	0.003798
13	dbsnp	rs301661	A	G	ALLELIC	6/16	2/48	8.378	1	0.003798
13	dbsnp	rs159891	A	G	ALLELIC	6/16	2/48	8.378	1	0.003798
13	dbsnp	rs214121	A	G	ALLELIC	6/16	2/48	8.378	1	0.003798
13	dbsnp	rs17505147	G	A	ALLELIC	6/16	2/48	8.378	1	0.003798
13	dbsnp	rs8001831	A	G	ALLELIC	6/16	2/48	8.378	1	0.003798
13	dbsnp	rs12050037	A	G	ALLELIC	6/16	2/48	8.378	1	0.003798
13	dbsnp	rs17069858	G	A	ALLELIC	6/16	2/48	8.378	1	0.003798
13	dbsnp	rs2031565	A	G	ALLELIC	6/16	2/48	8.378	1	0.003798
13	dbsnp	rs7322582	G	A	ALLELIC	2/20	22/28	8.378	1	0.003798
14	dbsnp	rs2242539	A	C	ALLELIC	6/16	2/48	8.378	1	0.003798
14	dbsnp	rs11624654	G	A	ALLELIC	6/16	2/48	8.378	1	0.003798
14	dbsnp	rs9888543	A	G	ALLELIC	2/20	22/28	8.378	1	0.003798

14	dbsnp	rs7144433	G	A	ALLELIC	6/16	2/48	8.378	1	0.003798
14	dbsnp	rs2300502	A	G	ALLELIC	6/16	2/48	8.378	1	0.003798
15	dbsnp	rs690399	A	C	ALLELIC	6/16	2/48	8.378	1	0.003798
15	dbsnp	rs11070964	G	A	ALLELIC	6/16	2/48	8.378	1	0.003798
15	dbsnp	rs566855	A	C	ALLELIC	6/16	2/48	8.378	1	0.003798
15	dbsnp	rs690449	G	A	ALLELIC	6/16	2/48	8.378	1	0.003798
15	dbsnp	rs9284297	A	G	ALLELIC	6/16	2/48	8.378	1	0.003798
15	dbsnp	rs553000	G	A	ALLELIC	6/16	2/48	8.378	1	0.003798
15	dbsnp	rs8037291	G	A	ALLELIC	6/16	2/48	8.378	1	0.003798
15	dbsnp	rs11631170	G	A	ALLELIC	6/16	2/48	8.378	1	0.003798
16	dbsnp	rs11861487	A	G	ALLELIC	6/16	2/48	8.378	1	0.003798
16	dbsnp	rs2075639	A	G	ALLELIC	6/16	2/48	8.378	1	0.003798
16	dbsnp	rs9938283	A	G	ALLELIC	6/16	2/48	8.378	1	0.003798
16	dbsnp	rs7197526	A	G	ALLELIC	2/20	22/28	8.378	1	0.003798
17	dbsnp	rs4359493	G	A	ALLELIC	6/16	2/48	8.378	1	0.003798
17	dbsnp	rs4598945	G	A	ALLELIC	6/16	2/48	8.378	1	0.003798
17	dbsnp	rs11649752	A	G	ALLELIC	6/16	2/48	8.378	1	0.003798
17	dbsnp	rs16976669	G	A	ALLELIC	6/16	2/48	8.378	1	0.003798
18	dbsnp	rs1034486	A	G	ALLELIC	6/16	2/48	8.378	1	0.003798
18	dbsnp	rs17816780	A	G	ALLELIC	6/16	2/48	8.378	1	0.003798
18	dbsnp	rs9958493	A	G	ALLELIC	6/16	2/48	8.378	1	0.003798
18	dbsnp	rs8093617	A	G	ALLELIC	6/16	2/48	8.378	1	0.003798
18	dbsnp	rs4940802	A	G	ALLELIC	6/16	2/48	8.378	1	0.003798
18	dbsnp	rs9960318	A	G	ALLELIC	6/16	2/48	8.378	1	0.003798
19	dbsnp	rs453679	G	A	ALLELIC	6/16	2/48	8.378	1	0.003798
19	dbsnp	rs11667458	A	G	ALLELIC	6/16	2/48	8.378	1	0.003798
19	dbsnp	rs7253228	G	A	ALLELIC	6/16	2/48	8.378	1	0.003798
20	dbsnp	rs221007	A	C	ALLELIC	6/16	2/48	8.378	1	0.003798
20	dbsnp	rs6035231	A	G	ALLELIC	2/20	22/28	8.378	1	0.003798
20	dbsnp	rs12479885	A	G	ALLELIC	6/16	2/48	8.378	1	0.003798
20	dbsnp	rs13043248	A	G	ALLELIC	6/16	2/48	8.378	1	0.003798
21	dbsnp	rs638268	G	A	ALLELIC	6/16	2/48	8.378	1	0.003798
21	dbsnp	rs2838869	A	G	ALLELIC	6/16	2/48	8.378	1	0.003798
22	dbsnp	rs5997026	G	A	ALLELIC	6/16	2/48	8.378	1	0.003798
22	dbsnp	rs8141254	G	A	ALLELIC	6/16	2/48	8.378	1	0.003798
22	dbsnp	rs5999196	A	G	ALLELIC	6/16	2/48	8.378	1	0.003798
22	dbsnp	rs2858237	G	A	ALLELIC	2/20	22/28	8.378	1	0.003798
22	dbsnp	rs5764573	A	G	ALLELIC	6/16	2/48	8.378	1	0.003798
23	dbsnp	rs6638764	A	G	ALLELIC	6/16	2/48	8.378	1	0.003798
23	dbsnp	rs4568774	A	G	ALLELIC	6/16	2/48	8.378	1	0.003798
23	dbsnp	rs9887650	G	A	ALLELIC	6/16	2/48	8.378	1	0.003798
23	dbsnp	rs3761590	A	C	ALLELIC	6/16	2/48	8.378	1	0.003798
23	dbsnp	rs5967045	G	A	ALLELIC	6/16	2/48	8.378	1	0.003798
23	dbsnp	rs5911602	G	A	ALLELIC	6/16	2/48	8.378	1	0.003798
23	dbsnp	rs12387435	A	C	ALLELIC	6/16	2/48	8.378	1	0.003798
23	dbsnp	rs6608141	A	G	ALLELIC	6/16	2/48	8.378	1	0.003798
23	dbsnp	rs4288491	A	G	ALLELIC	6/16	2/48	8.378	1	0.003798
23	dbsnp	rs6418944	A	G	ALLELIC	2/20	22/28	8.378	1	0.003798

2	dbsnp	rs13015524	G	A	ALLELIC	8/14	4/44	8.345	1	0.003868
2	dbsnp	rs6704601	G	A	ALLELIC	8/14	4/44	8.345	1	0.003868
1	dbsnp	rs860554	A	G	ALLELIC	0/22	15/35	8.337	1	0.003885
2	dbsnp	rs9989821	G	A	ALLELIC	0/22	15/35	8.337	1	0.003885
2	dbsnp	rs3754770	A	G	ALLELIC	0/22	15/35	8.337	1	0.003885
2	dbsnp	rs1434999	A	G	ALLELIC	0/22	15/35	8.337	1	0.003885
2	dbsnp	rs1522802	A	C	ALLELIC	0/22	15/35	8.337	1	0.003885
2	dbsnp	rs6431547	G	A	ALLELIC	0/22	15/35	8.337	1	0.003885
4	dbsnp	rs1450242	A	G	ALLELIC	0/22	15/35	8.337	1	0.003885
6	dbsnp	rs6925912	A	G	ALLELIC	0/22	15/35	8.337	1	0.003885
6	dbsnp	rs9459695	A	C	ALLELIC	0/22	15/35	8.337	1	0.003885
7	dbsnp	rs11772019	G	A	ALLELIC	0/22	15/35	8.337	1	0.003885
7	dbsnp	rs1852796	G	A	ALLELIC	0/22	15/35	8.337	1	0.003885
7	dbsnp	rs43002	G	A	ALLELIC	0/22	15/35	8.337	1	0.003885
8	dbsnp	rs4612351	G	A	ALLELIC	0/22	15/35	8.337	1	0.003885
10	dbsnp	rs2002950	G	A	ALLELIC	0/22	15/35	8.337	1	0.003885
10	dbsnp	rs11245085	A	G	ALLELIC	0/22	15/35	8.337	1	0.003885
15	dbsnp	rs2899517	A	C	ALLELIC	0/22	15/35	8.337	1	0.003885
15	dbsnp	rs6496239	G	A	ALLELIC	0/22	15/35	8.337	1	0.003885
18	dbsnp	rs10163707	A	G	ALLELIC	0/22	15/35	8.337	1	0.003885
18	dbsnp	rs931356	G	A	ALLELIC	0/22	15/35	8.337	1	0.003885
20	dbsnp	rs4813792	A	C	ALLELIC	0/22	15/35	8.337	1	0.003885
20	dbsnp	rs761684	A	G	ALLELIC	0/22	15/35	8.337	1	0.003885
20	dbsnp	rs6074962	A	G	ALLELIC	0/22	15/35	8.337	1	0.003885
23	dbsnp	rs5930019	G	A	ALLELIC	0/22	15/35	8.337	1	0.003885
23	dbsnp	rs5931703	A	G	ALLELIC	0/22	15/35	8.337	1	0.003885
1	dbsnp	rs11261073	A	G	ALLELIC	1/19	20/30	8.333	1	0.003892
1	dbsnp	rs1323816	A	G	ALLELIC	11/9	10/40	8.333	1	0.003892
1	dbsnp	rs12078288	A	G	ALLELIC	11/9	10/40	8.333	1	0.003892
1	dbsnp	rs7539202	G	A	ALLELIC	1/19	20/30	8.333	1	0.003892
1	dbsnp	rs10753990	A	C	ALLELIC	1/19	20/30	8.333	1	0.003892
1	dbsnp	rs12745924	G	A	ALLELIC	1/19	20/30	8.333	1	0.003892
1	dbsnp	rs1256603	G	A	ALLELIC	1/19	20/30	8.333	1	0.003892
2	dbsnp	rs10931477	G	A	ALLELIC	11/9	10/40	8.333	1	0.003892
2	dbsnp	rs10205602	G	A	ALLELIC	11/9	10/40	8.333	1	0.003892
4	dbsnp	rs4833993	G	A	ALLELIC	11/9	10/40	8.333	1	0.003892
4	dbsnp	rs11132138	G	A	ALLELIC	1/19	20/30	8.333	1	0.003892
4	dbsnp	rs11934546	G	A	ALLELIC	1/19	20/30	8.333	1	0.003892
7	dbsnp	rs17151997	G	A	ALLELIC	1/19	20/30	8.333	1	0.003892
8	dbsnp	rs1478892	A	C	ALLELIC	1/19	20/30	8.333	1	0.003892
8	dbsnp	rs436760	G	A	ALLELIC	1/19	20/30	8.333	1	0.003892
8	dbsnp	rs7836486	A	C	ALLELIC	11/9	10/40	8.333	1	0.003892
8	dbsnp	rs10441622	A	G	ALLELIC	11/9	10/40	8.333	1	0.003892
8	dbsnp	rs4873076	G	A	ALLELIC	11/9	10/40	8.333	1	0.003892
8	dbsnp	rs11136156	A	C	ALLELIC	11/9	10/40	8.333	1	0.003892
8	dbsnp	rs187219	G	A	ALLELIC	11/9	10/40	8.333	1	0.003892
10	dbsnp	rs927915	G	A	ALLELIC	11/9	10/40	8.333	1	0.003892
12	dbsnp	rs10860794	A	C	ALLELIC	1/19	20/30	8.333	1	0.003892

12	dbsnp	rs4764937	C	A	ALLELIC	11/9	10/40	8.333	1	0.003892
13	dbsnp	rs7322299	G	A	ALLELIC	11/9	10/40	8.333	1	0.003892
14	dbsnp	rs10135768	A	C	ALLELIC	1/19	20/30	8.333	1	0.003892
15	dbsnp	rs2683086	C	A	ALLELIC	11/9	10/40	8.333	1	0.003892
22	dbsnp	rs134128	G	A	ALLELIC	11/9	10/40	8.333	1	0.003892
23	dbsnp	rs5945157	G	A	ALLELIC	11/9	10/40	8.333	1	0.003892
18	dbsnp	rs1149356	A	G	ALLELIC	4/16	28/20	8.327	1	0.003906
1	dbsnp	rs2404990	G	A	ALLELIC	3/13	0/42	8.305	1	0.003955
1	dbsnp	rs12088178	G	A	ALLELIC	13/9	12/38	8.3	1	0.003965
1	dbsnp	rs5878	G	A	ALLELIC	13/9	12/38	8.3	1	0.003965
1	dbsnp	rs5877	G	A	ALLELIC	13/9	12/38	8.3	1	0.003965
1	dbsnp	rs10803066	G	A	ALLELIC	13/9	12/38	8.3	1	0.003965
2	dbsnp	rs12623348	G	A	ALLELIC	13/9	12/38	8.3	1	0.003965
2	dbsnp	rs6713577	G	A	ALLELIC	13/9	12/38	8.3	1	0.003965
2	dbsnp	rs7579033	G	A	ALLELIC	13/9	12/38	8.3	1	0.003965
3	dbsnp	rs778506	G	A	ALLELIC	13/9	12/38	8.3	1	0.003965
3	dbsnp	rs704533	A	G	ALLELIC	13/9	12/38	8.3	1	0.003965
3	dbsnp	rs4683702	C	A	ALLELIC	13/9	12/38	8.3	1	0.003965
3	dbsnp	rs1470121	A	G	ALLELIC	13/9	12/38	8.3	1	0.003965
3	dbsnp	rs7646258	C	A	ALLELIC	13/9	12/38	8.3	1	0.003965
3	dbsnp	rs11713634	A	G	ALLELIC	13/9	12/38	8.3	1	0.003965
3	dbsnp	rs11711157	A	G	ALLELIC	13/9	12/38	8.3	1	0.003965
3	dbsnp	rs3732477	A	G	ALLELIC	13/9	12/38	8.3	1	0.003965
3	dbsnp	rs11924930	A	G	ALLELIC	13/9	12/38	8.3	1	0.003965
4	dbsnp	rs9997830	C	A	ALLELIC	13/9	12/38	8.3	1	0.003965
4	dbsnp	rs2865763	A	C	ALLELIC	13/9	12/38	8.3	1	0.003965
4	dbsnp	rs17548053	A	G	ALLELIC	13/9	12/38	8.3	1	0.003965
5	dbsnp	rs2019251	C	A	ALLELIC	13/9	12/38	8.3	1	0.003965
5	dbsnp	rs26979	A	G	ALLELIC	13/9	12/38	8.3	1	0.003965
5	dbsnp	rs2548599	C	A	ALLELIC	13/9	12/38	8.3	1	0.003965
5	dbsnp	rs2927630	A	G	ALLELIC	13/9	12/38	8.3	1	0.003965
5	dbsnp	rs1427884	C	A	ALLELIC	13/9	12/38	8.3	1	0.003965
5	dbsnp	rs6885522	A	G	ALLELIC	13/9	12/38	8.3	1	0.003965
6	dbsnp	rs3799732	G	A	ALLELIC	13/9	12/38	8.3	1	0.003965
6	dbsnp	rs9364421	C	A	ALLELIC	13/9	12/38	8.3	1	0.003965
7	dbsnp	rs17166902	A	C	ALLELIC	13/9	12/38	8.3	1	0.003965
7	dbsnp	rs6597451	A	G	ALLELIC	13/9	12/38	8.3	1	0.003965
7	dbsnp	rs1561602	A	G	ALLELIC	13/9	12/38	8.3	1	0.003965
8	dbsnp	rs1028777	C	A	ALLELIC	13/9	12/38	8.3	1	0.003965
10	dbsnp	rs10998336	A	G	ALLELIC	13/9	12/38	8.3	1	0.003965
11	dbsnp	rs573549	A	G	ALLELIC	13/9	12/38	8.3	1	0.003965
11	dbsnp	rs3019797	G	A	ALLELIC	13/9	12/38	8.3	1	0.003965
11	dbsnp	rs2703761	G	A	ALLELIC	13/9	12/38	8.3	1	0.003965
11	dbsnp	rs1496248	G	A	ALLELIC	13/9	12/38	8.3	1	0.003965
12	dbsnp	rs2728554	C	A	ALLELIC	13/9	12/38	8.3	1	0.003965
12	dbsnp	rs10784294	A	G	ALLELIC	13/9	12/38	8.3	1	0.003965
12	dbsnp	rs10848374	G	A	ALLELIC	13/9	12/38	8.3	1	0.003965
13	dbsnp	rs942348	G	A	ALLELIC	13/9	12/38	8.3	1	0.003965

13	dbsnp	rs7991808	G	A	ALLELIC	13/9	12/38	8.3	1	0.003965
13	dbsnp	rs931004	A	G	ALLELIC	13/9	12/38	8.3	1	0.003965
14	dbsnp	rs6573329	A	G	ALLELIC	13/9	12/38	8.3	1	0.003965
14	dbsnp	rs10137188	A	G	ALLELIC	13/9	12/38	8.3	1	0.003965
14	dbsnp	rs1475022	A	G	ALLELIC	13/9	12/38	8.3	1	0.003965
14	dbsnp	rs2351271	G	A	ALLELIC	13/9	12/38	8.3	1	0.003965
14	dbsnp	rs3742636	A	C	ALLELIC	13/9	12/38	8.3	1	0.003965
20	dbsnp	rs8125417	A	G	ALLELIC	13/9	12/38	8.3	1	0.003965
21	dbsnp	rs2823128	A	G	ALLELIC	13/9	12/38	8.3	1	0.003965
23	dbsnp	rs233214	A	G	ALLELIC	13/9	12/38	8.3	1	0.003965
23	dbsnp	rs5907705	G	A	ALLELIC	13/9	12/38	8.3	1	0.003965
23	dbsnp	rs4825002	A	C	ALLELIC	13/9	12/38	8.3	1	0.003965
3	dbsnp	rs898411	G	A	ALLELIC	0/20	16/34	8.296	1	0.003973
3	dbsnp	rs11130377	G	A	ALLELIC	0/20	16/34	8.296	1	0.003973
3	dbsnp	rs9839536	A	G	ALLELIC	0/20	16/34	8.296	1	0.003973
4	dbsnp	rs4577620	A	G	ALLELIC	0/20	16/34	8.296	1	0.003973
7	dbsnp	rs4730462	G	A	ALLELIC	0/20	16/34	8.296	1	0.003973
7	dbsnp	rs1358434	A	G	ALLELIC	0/20	16/34	8.296	1	0.003973
15	dbsnp	rs7180196	G	A	ALLELIC	0/20	16/34	8.296	1	0.003973
16	dbsnp	rs7205337	G	A	ALLELIC	0/20	16/34	8.296	1	0.003973
15	dbsnp	rs16958561	G	A	ALLELIC	7/9	3/31	8.295	1	0.003975
14	dbsnp	rs12883738	G	A	ALLELIC	1/21	18/30	8.285	1	0.003998
2	dbsnp	rs12476858	A	G	ALLELIC	4/16	29/21	8.278	1	0.004012
2	dbsnp	rs7560771	A	G	ALLELIC	4/16	29/21	8.278	1	0.004012
5	dbsnp	rs7725393	A	C	ALLELIC	4/16	29/21	8.278	1	0.004012
11	dbsnp	rs2703752	A	C	ALLELIC	4/16	29/21	8.278	1	0.004012
12	dbsnp	rs11171856	A	G	ALLELIC	4/16	29/21	8.278	1	0.004012
15	dbsnp	rs8039405	A	G	ALLELIC	4/16	29/21	8.278	1	0.004012
18	dbsnp	rs9646545	G	A	ALLELIC	4/16	29/21	8.278	1	0.004012
25	dbsnp	rs5946367	G	A	ALLELIC	16/4	21/29	8.278	1	0.004012
1	dbsnp	rs2272908	A	G	ALLELIC	6/16	32/18	8.269	1	0.004033
1	dbsnp	rs10919132	G	A	ALLELIC	6/16	32/18	8.269	1	0.004033
1	dbsnp	rs10753786	A	G	ALLELIC	6/16	32/18	8.269	1	0.004033
2	dbsnp	rs6746740	A	G	ALLELIC	16/6	18/32	8.269	1	0.004033
2	dbsnp	rs1550316	A	G	ALLELIC	6/16	32/18	8.269	1	0.004033
2	dbsnp	rs7562518	G	A	ALLELIC	16/6	18/32	8.269	1	0.004033
3	dbsnp	rs9843391	A	G	ALLELIC	16/6	18/32	8.269	1	0.004033
3	dbsnp	rs9825772	A	G	ALLELIC	16/6	18/32	8.269	1	0.004033
4	dbsnp	rs13109943	C	A	ALLELIC	16/6	18/32	8.269	1	0.004033
4	dbsnp	rs1426936	G	A	ALLELIC	16/6	18/32	8.269	1	0.004033
4	dbsnp	rs10213203	A	G	ALLELIC	16/6	18/32	8.269	1	0.004033
5	dbsnp	rs734986	G	A	ALLELIC	16/6	18/32	8.269	1	0.004033
5	dbsnp	rs7700874	G	A	ALLELIC	16/6	18/32	8.269	1	0.004033
6	dbsnp	rs2182997	A	C	ALLELIC	16/6	18/32	8.269	1	0.004033
6	dbsnp	rs2277083	A	G	ALLELIC	16/6	18/32	8.269	1	0.004033
8	dbsnp	rs991359	G	A	ALLELIC	16/6	18/32	8.269	1	0.004033
8	dbsnp	rs478360	A	G	ALLELIC	16/6	18/32	8.269	1	0.004033
8	dbsnp	rs11135963	A	G	ALLELIC	16/6	18/32	8.269	1	0.004033

8	dbsnp	rs1985958	A	G	ALLELIC	16/6	18/32	8.269	1	0.004033
9	dbsnp	rs2034764	G	A	ALLELIC	16/6	18/32	8.269	1	0.004033
9	dbsnp	rs1378062	G	A	ALLELIC	16/6	18/32	8.269	1	0.004033
9	dbsnp	rs10814274	A	G	ALLELIC	16/6	18/32	8.269	1	0.004033
9	dbsnp	rs1570246	A	C	ALLELIC	16/6	18/32	8.269	1	0.004033
9	dbsnp	rs1570249	A	G	ALLELIC	16/6	18/32	8.269	1	0.004033
10	dbsnp	rs7915072	A	C	ALLELIC	16/6	18/32	8.269	1	0.004033
10	dbsnp	rs4255455	C	A	ALLELIC	16/6	18/32	8.269	1	0.004033
11	dbsnp	rs658573	A	G	ALLELIC	16/6	18/32	8.269	1	0.004033
11	dbsnp	rs1461687	A	G	ALLELIC	16/6	18/32	8.269	1	0.004033
11	dbsnp	rs1793322	A	C	ALLELIC	16/6	18/32	8.269	1	0.004033
12	dbsnp	rs651083	A	C	ALLELIC	16/6	18/32	8.269	1	0.004033
13	dbsnp	rs1218877	A	C	ALLELIC	6/16	32/18	8.269	1	0.004033
13	dbsnp	rs285081	G	A	ALLELIC	16/6	18/32	8.269	1	0.004033
13	dbsnp	rs4772979	G	A	ALLELIC	16/6	18/32	8.269	1	0.004033
13	dbsnp	rs9514878	G	A	ALLELIC	16/6	18/32	8.269	1	0.004033
14	dbsnp	rs7148400	G	A	ALLELIC	16/6	18/32	8.269	1	0.004033
15	dbsnp	rs1840300	A	G	ALLELIC	16/6	18/32	8.269	1	0.004033
15	dbsnp	rs2581332	A	G	ALLELIC	16/6	18/32	8.269	1	0.004033
16	dbsnp	rs904195	A	G	ALLELIC	16/6	18/32	8.269	1	0.004033
17	dbsnp	rs11651964	A	G	ALLELIC	16/6	18/32	8.269	1	0.004033
18	dbsnp	rs4493141	A	C	ALLELIC	16/6	18/32	8.269	1	0.004033
18	dbsnp	rs4800205	A	C	ALLELIC	16/6	18/32	8.269	1	0.004033
18	dbsnp	rs330295	C	A	ALLELIC	16/6	18/32	8.269	1	0.004033
20	dbsnp	rs6063073	A	G	ALLELIC	6/16	32/18	8.269	1	0.004033
20	dbsnp	rs1739592	A	G	ALLELIC	16/6	18/32	8.269	1	0.004033
22	dbsnp	rs2246092	A	G	ALLELIC	16/6	18/32	8.269	1	0.004033
23	dbsnp	rs2249047	A	G	ALLELIC	16/6	18/32	8.269	1	0.004033
23	dbsnp	rs1024429	A	G	ALLELIC	16/6	18/32	8.269	1	0.004033
1	dbsnp	rs1408854	G	A	ALLELIC	13/7	14/36	8.254	1	0.004066
1	dbsnp	rs607007	G	A	ALLELIC	13/7	14/36	8.254	1	0.004066
2	dbsnp	rs6736664	A	G	ALLELIC	13/7	14/36	8.254	1	0.004066
2	dbsnp	rs7591274	A	G	ALLELIC	13/7	14/36	8.254	1	0.004066
2	dbsnp	rs4277473	G	A	ALLELIC	13/7	14/36	8.254	1	0.004066
4	dbsnp	rs11731078	G	A	ALLELIC	13/7	14/36	8.254	1	0.004066
5	dbsnp	rs4521479	G	A	ALLELIC	13/7	14/36	8.254	1	0.004066
5	dbsnp	rs4602632	A	G	ALLELIC	13/7	14/36	8.254	1	0.004066
6	dbsnp	rs3799585	A	G	ALLELIC	13/7	14/36	8.254	1	0.004066
8	dbsnp	rs1788190	A	G	ALLELIC	13/7	14/36	8.254	1	0.004066
9	dbsnp	rs10984782	A	G	ALLELIC	13/7	14/36	8.254	1	0.004066
10	dbsnp	rs1962474	G	A	ALLELIC	13/7	14/36	8.254	1	0.004066
11	dbsnp	rs10768246	G	A	ALLELIC	13/7	14/36	8.254	1	0.004066
12	dbsnp	rs12313584	A	G	ALLELIC	13/7	14/36	8.254	1	0.004066
15	dbsnp	rs1107113	A	C	ALLELIC	13/7	14/36	8.254	1	0.004066
17	dbsnp	rs12452084	A	G	ALLELIC	13/7	14/36	8.254	1	0.004066
18	dbsnp	rs9965051	G	A	ALLELIC	13/7	14/36	8.254	1	0.004066
23	dbsnp	rs6638917	A	G	ALLELIC	13/7	14/36	8.254	1	0.004066
23	dbsnp	rs2239490	C	A	ALLELIC	13/7	14/36	8.254	1	0.004066

23	dbsnp	rs6641108	A	G	ALLELIC	13/7	14/36	8.254	1	0.004066
23	dbsnp	rs5928564	G	A	ALLELIC	13/7	14/36	8.254	1	0.004066
1	dbsnp	rs6656554	C	A	ALLELIC	12/8	12/38	8.217	1	0.004149
4	dbsnp	rs7655988	A	G	ALLELIC	12/8	12/38	8.217	1	0.004149
5	dbsnp	rs151915	G	A	ALLELIC	12/8	12/38	8.217	1	0.004149
6	dbsnp	rs1572265	G	A	ALLELIC	12/8	12/38	8.217	1	0.004149
8	dbsnp	rs10958798	G	A	ALLELIC	12/8	12/38	8.217	1	0.004149
8	dbsnp	rs1603681	G	A	ALLELIC	12/8	12/38	8.217	1	0.004149
8	dbsnp	rs11136174	G	A	ALLELIC	12/8	12/38	8.217	1	0.004149
9	dbsnp	rs639949	C	A	ALLELIC	12/8	12/38	8.217	1	0.004149
10	dbsnp	rs12358164	C	A	ALLELIC	12/8	12/38	8.217	1	0.004149
11	dbsnp	rs10742809	C	A	ALLELIC	12/8	12/38	8.217	1	0.004149
14	dbsnp	rs12893435	A	G	ALLELIC	12/8	12/38	8.217	1	0.004149
15	dbsnp	rs1994714	A	G	ALLELIC	12/8	12/38	8.217	1	0.004149
17	dbsnp	rs12941198	A	C	ALLELIC	12/8	12/38	8.217	1	0.004149
18	dbsnp	rs1567038	A	C	ALLELIC	12/8	12/38	8.217	1	0.004149
18	dbsnp	rs1828037	A	G	ALLELIC	12/8	12/38	8.217	1	0.004149
19	dbsnp	rs1529482	G	A	ALLELIC	12/8	12/38	8.217	1	0.004149
4	dbsnp	rs11132556	A	G	ALLELIC	2/20	21/27	8.214	1	0.004156
2	dbsnp	rs17655123	A	G	ALLELIC	5/17	1/47	8.204	1	0.00418
9	dbsnp	rs1064560	A	G	ALLELIC	5/17	1/47	8.204	1	0.00418
13	dbsnp	rs2277440	A	G	ALLELIC	5/17	1/47	8.204	1	0.00418
13	dbsnp	rs12865469	G	A	ALLELIC	5/17	1/47	8.204	1	0.00418
23	dbsnp	rs5956720	G	A	ALLELIC	5/17	1/47	8.204	1	0.00418
23	dbsnp	rs12832847	G	A	ALLELIC	5/17	1/47	8.204	1	0.00418
2	dbsnp	rs10187143	A	G	ALLELIC	14/8	14/36	8.164	1	0.004273
2	dbsnp	rs10865404	A	G	ALLELIC	14/8	14/36	8.164	1	0.004273
2	dbsnp	rs11687402	G	A	ALLELIC	14/8	14/36	8.164	1	0.004273
2	dbsnp	rs7587426	G	A	ALLELIC	14/8	14/36	8.164	1	0.004273
2	dbsnp	rs13411041	A	G	ALLELIC	14/8	14/36	8.164	1	0.004273
2	dbsnp	rs6435396	G	A	ALLELIC	14/8	14/36	8.164	1	0.004273
2	dbsnp	rs16863916	A	G	ALLELIC	14/8	14/36	8.164	1	0.004273
3	dbsnp	rs536127	A	G	ALLELIC	14/8	14/36	8.164	1	0.004273
4	dbsnp	rs4699523	G	A	ALLELIC	14/8	14/36	8.164	1	0.004273
4	dbsnp	rs2324	A	G	ALLELIC	14/8	14/36	8.164	1	0.004273
4	dbsnp	rs419764	A	G	ALLELIC	14/8	14/36	8.164	1	0.004273
4	dbsnp	rs12502197	G	A	ALLELIC	14/8	14/36	8.164	1	0.004273
4	dbsnp	rs17378274	C	A	ALLELIC	14/8	14/36	8.164	1	0.004273
5	dbsnp	rs959662	G	A	ALLELIC	14/8	14/36	8.164	1	0.004273
6	dbsnp	rs1871428	A	G	ALLELIC	14/8	14/36	8.164	1	0.004273
8	dbsnp	rs2617076	A	C	ALLELIC	14/8	14/36	8.164	1	0.004273
8	dbsnp	rs4301434	G	A	ALLELIC	14/8	14/36	8.164	1	0.004273
9	dbsnp	rs10739615	G	A	ALLELIC	14/8	14/36	8.164	1	0.004273
10	dbsnp	rs2454803	G	A	ALLELIC	14/8	14/36	8.164	1	0.004273
10	dbsnp	rs2505176	A	G	ALLELIC	14/8	14/36	8.164	1	0.004273
11	dbsnp	rs7931228	A	G	ALLELIC	14/8	14/36	8.164	1	0.004273
11	dbsnp	rs1506981	G	A	ALLELIC	14/8	14/36	8.164	1	0.004273
12	dbsnp	rs11616084	A	G	ALLELIC	14/8	14/36	8.164	1	0.004273

13	dbsnp	rs2803219	G	A	ALLELIC	14/8	14/36	8.164	1	0.004273
13	dbsnp	rs7984646	C	A	ALLELIC	14/8	14/36	8.164	1	0.004273
14	dbsnp	rs13379337	A	C	ALLELIC	14/8	14/36	8.164	1	0.004273
14	dbsnp	rs1467529	C	A	ALLELIC	14/8	14/36	8.164	1	0.004273
14	dbsnp	rs1628501	G	A	ALLELIC	14/8	14/36	8.164	1	0.004273
15	dbsnp	rs876383	A	G	ALLELIC	14/8	14/36	8.164	1	0.004273
16	dbsnp	rs12930148	A	C	ALLELIC	14/8	14/36	8.164	1	0.004273
17	dbsnp	rs2058259	A	C	ALLELIC	14/8	14/36	8.164	1	0.004273
18	dbsnp	rs1785113	G	A	ALLELIC	14/8	14/36	8.164	1	0.004273
19	dbsnp	rs3745129	A	G	ALLELIC	14/8	14/36	8.164	1	0.004273
20	dbsnp	rs1555141	A	G	ALLELIC	14/8	14/36	8.164	1	0.004273
20	dbsnp	rs913664	G	A	ALLELIC	14/8	14/36	8.164	1	0.004273
21	dbsnp	rs7280789	A	C	ALLELIC	14/8	14/36	8.164	1	0.004273
23	dbsnp	rs2405113	A	C	ALLELIC	14/8	14/36	8.164	1	0.004273
23	dbsnp	rs829802	A	G	ALLELIC	14/8	14/36	8.164	1	0.004273
1	dbsnp	rs17374565	G	A	ALLELIC	15/7	16/34	8.158	1	0.004288
1	dbsnp	rs10920424	A	G	ALLELIC	15/7	16/34	8.158	1	0.004288
1	dbsnp	rs7516991	G	A	ALLELIC	15/7	16/34	8.158	1	0.004288
1	dbsnp	rs1856294	C	A	ALLELIC	15/7	16/34	8.158	1	0.004288
1	dbsnp	rs1340202	G	A	ALLELIC	15/7	16/34	8.158	1	0.004288
1	dbsnp	rs11119547	A	G	ALLELIC	15/7	16/34	8.158	1	0.004288
2	dbsnp	rs3771408	A	G	ALLELIC	15/7	16/34	8.158	1	0.004288
2	dbsnp	rs11684234	G	A	ALLELIC	15/7	16/34	8.158	1	0.004288
3	dbsnp	rs437555	A	G	ALLELIC	15/7	16/34	8.158	1	0.004288
3	dbsnp	rs588801	A	G	ALLELIC	15/7	16/34	8.158	1	0.004288
3	dbsnp	rs435698	G	A	ALLELIC	15/7	16/34	8.158	1	0.004288
3	dbsnp	rs988397	A	G	ALLELIC	15/7	16/34	8.158	1	0.004288
4	dbsnp	rs9998340	A	G	ALLELIC	15/7	16/34	8.158	1	0.004288
4	dbsnp	rs1951235	G	A	ALLELIC	15/7	16/34	8.158	1	0.004288
4	dbsnp	rs2079112	G	A	ALLELIC	15/7	16/34	8.158	1	0.004288
4	dbsnp	rs1077767	G	A	ALLELIC	15/7	16/34	8.158	1	0.004288
5	dbsnp	rs6595456	G	A	ALLELIC	15/7	16/34	8.158	1	0.004288
5	dbsnp	rs740750	G	A	ALLELIC	15/7	16/34	8.158	1	0.004288
7	dbsnp	rs2106303	C	A	ALLELIC	15/7	16/34	8.158	1	0.004288
7	dbsnp	rs1440459	G	A	ALLELIC	15/7	16/34	8.158	1	0.004288
8	dbsnp	rs17676811	A	G	ALLELIC	15/7	16/34	8.158	1	0.004288
8	dbsnp	rs7844297	G	A	ALLELIC	15/7	16/34	8.158	1	0.004288
8	dbsnp	rs12550697	A	G	ALLELIC	15/7	16/34	8.158	1	0.004288
8	dbsnp	rs7823113	G	A	ALLELIC	15/7	16/34	8.158	1	0.004288
8	dbsnp	rs7833637	A	G	ALLELIC	15/7	16/34	8.158	1	0.004288
9	dbsnp	rs2375957	A	G	ALLELIC	15/7	16/34	8.158	1	0.004288
9	dbsnp	rs1336336	A	G	ALLELIC	15/7	16/34	8.158	1	0.004288
9	dbsnp	rs7862236	C	A	ALLELIC	15/7	16/34	8.158	1	0.004288
9	dbsnp	rs605576	C	A	ALLELIC	15/7	16/34	8.158	1	0.004288
10	dbsnp	rs691005	A	G	ALLELIC	15/7	16/34	8.158	1	0.004288
10	dbsnp	rs11245344	G	A	ALLELIC	15/7	16/34	8.158	1	0.004288
10	dbsnp	rs10901818	G	A	ALLELIC	15/7	16/34	8.158	1	0.004288
10	dbsnp	rs11245366	G	A	ALLELIC	15/7	16/34	8.158	1	0.004288

10	dbsnp	rs2303611	G	A	ALLELIC	15/7	16/34	8.158	1	0.004288
11	dbsnp	rs7940419	C	A	ALLELIC	15/7	16/34	8.158	1	0.004288
12	dbsnp	rs6539845	A	G	ALLELIC	15/7	16/34	8.158	1	0.004288
12	dbsnp	rs10847323	A	G	ALLELIC	15/7	16/34	8.158	1	0.004288
13	dbsnp	rs1343607	A	G	ALLELIC	15/7	16/34	8.158	1	0.004288
13	dbsnp	rs7323372	G	A	ALLELIC	15/7	16/34	8.158	1	0.004288
13	dbsnp	rs9577907	C	A	ALLELIC	15/7	16/34	8.158	1	0.004288
15	dbsnp	rs9972361	A	G	ALLELIC	15/7	16/34	8.158	1	0.004288
15	dbsnp	rs11072458	A	G	ALLELIC	15/7	16/34	8.158	1	0.004288
15	dbsnp	rs10162627	A	G	ALLELIC	15/7	16/34	8.158	1	0.004288
15	dbsnp	rs2168351	G	A	ALLELIC	15/7	16/34	8.158	1	0.004288
18	dbsnp	rs3861284	A	G	ALLELIC	15/7	16/34	8.158	1	0.004288
20	dbsnp	rs2297434	A	G	ALLELIC	15/7	16/34	8.158	1	0.004288
23	dbsnp	rs12687986	A	G	ALLELIC	15/7	16/34	8.158	1	0.004288
23	dbsnp	rs12852636	A	G	ALLELIC	15/7	16/34	8.158	1	0.004288
23	dbsnp	rs5929448	A	G	ALLELIC	15/7	16/34	8.158	1	0.004288
12	dbsnp	rs7301895	A	G	ALLELIC	4/18	0/42	8.145	1	0.004317
23	dbsnp	rs5983595	G	A	ALLELIC	11/7	12/38	8.144	1	0.004321
12	dbsnp	rs10778226	C	A	ALLELIC	1/19	19/29	8.133	1	0.004347
5	dbsnp	rs7719818	C	A	ALLELIC	5/13	2/48	8.103	1	0.004418
19	dbsnp	rs10410012	A	G	ALLELIC	5/13	2/48	8.103	1	0.004418
13	dbsnp	rs2325234	A	G	ALLELIC	10/12	6/38	8.085	1	0.004463
1	dbsnp	rs1327121	G	A	ALLELIC	2/18	23/27	8.064	1	0.004515
1	dbsnp	rs11208654	G	A	ALLELIC	2/18	23/27	8.064	1	0.004515
2	dbsnp	rs357977	A	G	ALLELIC	2/18	23/27	8.064	1	0.004515
3	dbsnp	rs953201	G	A	ALLELIC	2/18	23/27	8.064	1	0.004515
9	dbsnp	rs4548247	G	A	ALLELIC	2/18	23/27	8.064	1	0.004515
9	dbsnp	rs10780936	A	G	ALLELIC	2/18	23/27	8.064	1	0.004515
12	dbsnp	rs7297062	G	A	ALLELIC	2/18	23/27	8.064	1	0.004515
13	dbsnp	rs2984848	A	C	ALLELIC	2/18	23/27	8.064	1	0.004515
14	dbsnp	rs10148982	G	A	ALLELIC	2/18	23/27	8.064	1	0.004515
15	dbsnp	rs907396	C	A	ALLELIC	2/18	23/27	8.064	1	0.004515
19	dbsnp	rs4802602	A	G	ALLELIC	2/18	23/27	8.064	1	0.004515
11	dbsnp	rs7447	G	A	ALLELIC	8/12	4/38	8.062	1	0.00452
2	dbsnp	rs11684742	C	A	ALLELIC	3/17	26/24	8.06	1	0.004526
6	dbsnp	rs311686	A	G	ALLELIC	3/17	26/24	8.06	1	0.004526
6	dbsnp	rs311685	A	G	ALLELIC	3/17	26/24	8.06	1	0.004526
9	dbsnp	rs6476690	A	C	ALLELIC	3/17	26/24	8.06	1	0.004526
9	dbsnp	rs10868444	G	A	ALLELIC	3/17	26/24	8.06	1	0.004526
9	dbsnp	rs1537190	G	A	ALLELIC	3/17	26/24	8.06	1	0.004526
9	dbsnp	rs7865902	A	C	ALLELIC	3/17	26/24	8.06	1	0.004526
13	dbsnp	rs1322939	G	A	ALLELIC	3/17	26/24	8.06	1	0.004526
16	dbsnp	rs8056792	A	G	ALLELIC	3/17	26/24	8.06	1	0.004526
18	dbsnp	rs9960331	C	A	ALLELIC	3/17	26/24	8.06	1	0.004526
22	dbsnp	rs713964	G	A	ALLELIC	3/17	26/24	8.06	1	0.004526
5	dbsnp	rs7729880	G	A	ALLELIC	10/10	8/40	8.059	1	0.004527
1	dbsnp	rs7546487	A	G	ALLELIC	7/15	3/45	8.054	1	0.00454
5	dbsnp	rs3814066	G	A	ALLELIC	7/15	3/45	8.054	1	0.00454

5	dbsnp	rs12519614	G	A	ALLELIC	7/15	3/45	8.054	1	0.00454
10	dbsnp	rs3814219	A	G	ALLELIC	7/15	3/45	8.054	1	0.00454
12	dbsnp	rs4762367	A	G	ALLELIC	7/15	3/45	8.054	1	0.00454
23	dbsnp	rs5931130	G	A	ALLELIC	7/15	3/45	8.054	1	0.00454
22	dbsnp	rs9611577	C	A	ALLELIC	11/11	6/32	8.031	1	0.004599
6	dbsnp	rs2051538	A	G	ALLELIC	0/22	14/34	8.021	1	0.004624
3	dbsnp	rs11714766	A	C	ALLELIC	3/17	25/23	8.015	1	0.004638
19	dbsnp	rs7254323	G	A	ALLELIC	12/8	11/35	7.995	1	0.00469
1	dbsnp	rs11580863	A	G	ALLELIC	4/18	27/23	7.994	1	0.004692
1	dbsnp	rs6694114	A	G	ALLELIC	4/18	27/23	7.994	1	0.004692
1	dbsnp	rs1770691	A	G	ALLELIC	4/18	27/23	7.994	1	0.004692
1	dbsnp	rs10908751	G	A	ALLELIC	4/18	27/23	7.994	1	0.004692
1	dbsnp	rs4393149	G	A	ALLELIC	4/18	27/23	7.994	1	0.004692
1	dbsnp	rs1194604	C	A	ALLELIC	4/18	27/23	7.994	1	0.004692
5	dbsnp	rs400155	G	A	ALLELIC	4/18	27/23	7.994	1	0.004692
5	dbsnp	rs10059467	A	G	ALLELIC	4/18	27/23	7.994	1	0.004692
5	dbsnp	rs13188843	G	A	ALLELIC	4/18	27/23	7.994	1	0.004692
5	dbsnp	rs740477	G	A	ALLELIC	4/18	27/23	7.994	1	0.004692
5	dbsnp	rs12655091	A	G	ALLELIC	4/18	27/23	7.994	1	0.004692
5	dbsnp	rs6860134	G	A	ALLELIC	4/18	27/23	7.994	1	0.004692
5	dbsnp	rs966180	A	G	ALLELIC	4/18	27/23	7.994	1	0.004692
6	dbsnp	rs1206917	A	G	ALLELIC	4/18	27/23	7.994	1	0.004692
7	dbsnp	rs12704876	G	A	ALLELIC	4/18	27/23	7.994	1	0.004692
8	dbsnp	rs10956139	A	G	ALLELIC	4/18	27/23	7.994	1	0.004692
8	dbsnp	rs3812436	A	G	ALLELIC	4/18	27/23	7.994	1	0.004692
9	dbsnp	rs1006698	A	C	ALLELIC	4/18	27/23	7.994	1	0.004692
9	dbsnp	rs1927973	G	A	ALLELIC	4/18	27/23	7.994	1	0.004692
10	dbsnp	rs9423540	A	C	ALLELIC	4/18	27/23	7.994	1	0.004692
10	dbsnp	rs1155447	A	G	ALLELIC	4/18	27/23	7.994	1	0.004692
10	dbsnp	rs4500401	G	A	ALLELIC	4/18	27/23	7.994	1	0.004692
11	dbsnp	rs661348	G	A	ALLELIC	4/18	27/23	7.994	1	0.004692
11	dbsnp	rs1945410	A	C	ALLELIC	4/18	27/23	7.994	1	0.004692
11	dbsnp	rs1820453	A	C	ALLELIC	4/18	27/23	7.994	1	0.004692
11	dbsnp	rs2033083	G	A	ALLELIC	4/18	27/23	7.994	1	0.004692
12	dbsnp	rs12309211	G	A	ALLELIC	4/18	27/23	7.994	1	0.004692
12	dbsnp	rs10507115	A	C	ALLELIC	4/18	27/23	7.994	1	0.004692
12	dbsnp	rs7136537	A	C	ALLELIC	4/18	27/23	7.994	1	0.004692
13	dbsnp	rs7317235	G	A	ALLELIC	4/18	27/23	7.994	1	0.004692
13	dbsnp	rs12876395	G	A	ALLELIC	4/18	27/23	7.994	1	0.004692
14	dbsnp	rs10135113	A	C	ALLELIC	4/18	27/23	7.994	1	0.004692
14	dbsnp	rs2416021	A	C	ALLELIC	4/18	27/23	7.994	1	0.004692
15	dbsnp	rs11635223	A	G	ALLELIC	4/18	27/23	7.994	1	0.004692
15	dbsnp	rs10518966	A	G	ALLELIC	4/18	27/23	7.994	1	0.004692
17	dbsnp	rs11658790	C	A	ALLELIC	4/18	27/23	7.994	1	0.004692
17	dbsnp	rs917054	A	G	ALLELIC	4/18	27/23	7.994	1	0.004692
17	dbsnp	rs9891238	G	A	ALLELIC	4/18	27/23	7.994	1	0.004692
23	dbsnp	rs929217	G	A	ALLELIC	4/18	27/23	7.994	1	0.004692
23	dbsnp	rs6528239	G	A	ALLELIC	4/18	27/23	7.994	1	0.004692

14	dbsnp	rs17253633	G	A	ALLELIC	8/12	5/43	7.99	1	0.004703
5	dbsnp	rs718927	A	G	ALLELIC	4/18	26/22	7.977	1	0.004738
13	dbsnp	rs1932298	G	A	ALLELIC	4/18	26/22	7.977	1	0.004738
2	dbsnp	rs1016062	A	G	ALLELIC	6/16	2/46	7.957	1	0.004791
3	dbsnp	rs13083375	A	C	ALLELIC	6/16	2/46	7.957	1	0.004791
5	dbsnp	rs4326178	A	G	ALLELIC	6/16	2/46	7.957	1	0.004791
6	dbsnp	rs538905	A	C	ALLELIC	6/16	2/46	7.957	1	0.004791
10	dbsnp	rs4881298	A	C	ALLELIC	6/16	2/46	7.957	1	0.004791
11	dbsnp	rs11234218	A	G	ALLELIC	6/16	2/46	7.957	1	0.004791
23	dbsnp	rs4424440	G	A	ALLELIC	6/16	2/46	7.957	1	0.004791
23	dbsnp	rs1381730	G	A	ALLELIC	6/16	2/46	7.957	1	0.004791
1	dbsnp	rs6604725	A	G	ALLELIC	12/10	10/38	7.956	1	0.004794
9	dbsnp	rs715119	A	G	ALLELIC	12/10	10/38	7.956	1	0.004794
20	dbsnp	rs6136630	G	A	ALLELIC	12/10	10/38	7.956	1	0.004794
9	dbsnp	rs2590498	G	A	ALLELIC	14/6	15/31	7.912	1	0.004911
2	dbsnp	rs35896030	G	A	ALLELIC	7/13	4/46	7.863	1	0.005046
3	dbsnp	rs6785153	C	A	ALLELIC	7/13	4/46	7.863	1	0.005046
4	dbsnp	rs1573494	G	A	ALLELIC	7/13	4/46	7.863	1	0.005046
4	dbsnp	rs17586724	G	A	ALLELIC	7/13	4/46	7.863	1	0.005046
5	dbsnp	rs6871810	G	A	ALLELIC	7/13	4/46	7.863	1	0.005046
5	dbsnp	rs4835997	A	C	ALLELIC	7/13	4/46	7.863	1	0.005046
5	dbsnp	rs7718731	G	A	ALLELIC	7/13	4/46	7.863	1	0.005046
5	dbsnp	rs6880509	G	A	ALLELIC	7/13	4/46	7.863	1	0.005046
5	dbsnp	rs1975525	G	A	ALLELIC	7/13	4/46	7.863	1	0.005046
5	dbsnp	rs12516521	A	G	ALLELIC	7/13	4/46	7.863	1	0.005046
6	dbsnp	rs2033705	G	A	ALLELIC	7/13	4/46	7.863	1	0.005046
7	dbsnp	rs3211956	C	A	ALLELIC	7/13	4/46	7.863	1	0.005046
8	dbsnp	rs1365008	C	A	ALLELIC	7/13	4/46	7.863	1	0.005046
9	dbsnp	rs2273715	G	A	ALLELIC	7/13	4/46	7.863	1	0.005046
9	dbsnp	rs4604546	G	A	ALLELIC	7/13	4/46	7.863	1	0.005046
10	dbsnp	rs596642	G	A	ALLELIC	7/13	4/46	7.863	1	0.005046
10	dbsnp	rs4329592	G	A	ALLELIC	7/13	4/46	7.863	1	0.005046
10	dbsnp	rs11596104	A	C	ALLELIC	7/13	4/46	7.863	1	0.005046
10	dbsnp	rs2001515	A	C	ALLELIC	7/13	4/46	7.863	1	0.005046
11	dbsnp	rs11037204	C	A	ALLELIC	7/13	4/46	7.863	1	0.005046
11	dbsnp	rs1785827	C	A	ALLELIC	7/13	4/46	7.863	1	0.005046
11	dbsnp	rs1540124	A	G	ALLELIC	7/13	4/46	7.863	1	0.005046
11	dbsnp	rs1060211	G	A	ALLELIC	7/13	4/46	7.863	1	0.005046
12	dbsnp	rs10506808	G	A	ALLELIC	7/13	4/46	7.863	1	0.005046
12	dbsnp	rs2160428	A	G	ALLELIC	7/13	4/46	7.863	1	0.005046
14	dbsnp	rs4898957	A	G	ALLELIC	7/13	4/46	7.863	1	0.005046
14	dbsnp	rs2255146	A	G	ALLELIC	7/13	4/46	7.863	1	0.005046
14	dbsnp	rs6575045	G	A	ALLELIC	7/13	4/46	7.863	1	0.005046
14	dbsnp	rs7148212	G	A	ALLELIC	7/13	4/46	7.863	1	0.005046
14	dbsnp	rs2295133	A	G	ALLELIC	7/13	4/46	7.863	1	0.005046
15	dbsnp	rs1996671	A	G	ALLELIC	7/13	4/46	7.863	1	0.005046
20	dbsnp	rs442143	A	G	ALLELIC	7/13	4/46	7.863	1	0.005046
23	dbsnp	rs5916208	G	A	ALLELIC	7/13	4/46	7.863	1	0.005046

2	dbsnp	rs6721089	G	A	ALLELIC	13/5	17/33	7.843	1	0.005102
1	dbsnp	rs12094935	A	G	ALLELIC	3/17	0/50	7.836	1	0.005122
1	dbsnp	rs872595	G	A	ALLELIC	3/17	0/50	7.836	1	0.005122
1	dbsnp	rs17111206	A	G	ALLELIC	3/17	0/50	7.836	1	0.005122
2	dbsnp	rs2971884	A	G	ALLELIC	3/17	0/50	7.836	1	0.005122
2	dbsnp	rs13387850	A	G	ALLELIC	3/17	0/50	7.836	1	0.005122
3	dbsnp	rs6783389	A	G	ALLELIC	3/17	0/50	7.836	1	0.005122
4	dbsnp	rs1451634	G	A	ALLELIC	3/17	0/50	7.836	1	0.005122
4	dbsnp	rs7660712	C	A	ALLELIC	3/17	0/50	7.836	1	0.005122
5	dbsnp	rs1870658	G	A	ALLELIC	3/17	0/50	7.836	1	0.005122
5	dbsnp	rs624943	G	A	ALLELIC	3/17	0/50	7.836	1	0.005122
5	dbsnp	rs153118	G	A	ALLELIC	3/17	0/50	7.836	1	0.005122
6	dbsnp	rs13208734	G	A	ALLELIC	3/17	0/50	7.836	1	0.005122
7	dbsnp	rs17595402	A	C	ALLELIC	3/17	0/50	7.836	1	0.005122
8	dbsnp	rs12545323	A	G	ALLELIC	3/17	0/50	7.836	1	0.005122
8	dbsnp	rs11691	A	G	ALLELIC	3/17	0/50	7.836	1	0.005122
8	dbsnp	rs2272722	G	A	ALLELIC	3/17	0/50	7.836	1	0.005122
10	dbsnp	rs10508374	C	A	ALLELIC	3/17	0/50	7.836	1	0.005122
10	dbsnp	rs7089847	G	A	ALLELIC	3/17	0/50	7.836	1	0.005122
10	dbsnp	rs6585391	A	G	ALLELIC	3/17	0/50	7.836	1	0.005122
10	dbsnp	rs4751086	A	G	ALLELIC	3/17	0/50	7.836	1	0.005122
12	dbsnp	rs1990639	G	A	ALLELIC	3/17	0/50	7.836	1	0.005122
14	dbsnp	rs7159288	G	A	ALLELIC	3/17	0/50	7.836	1	0.005122
17	dbsnp	rs1806269	A	C	ALLELIC	3/17	0/50	7.836	1	0.005122
17	dbsnp	rs874640	A	C	ALLELIC	3/17	0/50	7.836	1	0.005122
17	dbsnp	rs4792952	A	G	ALLELIC	3/17	0/50	7.836	1	0.005122
17	dbsnp	rs3922318	G	A	ALLELIC	3/17	0/50	7.836	1	0.005122
19	dbsnp	rs3764573	A	G	ALLELIC	3/17	0/50	7.836	1	0.005122
20	dbsnp	rs4635580	A	G	ALLELIC	3/17	0/50	7.836	1	0.005122
22	dbsnp	rs5992169	A	G	ALLELIC	3/17	0/50	7.836	1	0.005122
22	dbsnp	rs467998	C	A	ALLELIC	3/17	0/50	7.836	1	0.005122
22	dbsnp	rs135868	A	G	ALLELIC	3/17	0/50	7.836	1	0.005122
23	dbsnp	rs5991839	A	G	ALLELIC	3/17	0/50	7.836	1	0.005122
23	dbsnp	rs5968690	G	A	ALLELIC	3/17	0/50	7.836	1	0.005122
23	dbsnp	rs9306818	C	A	ALLELIC	3/17	0/50	7.836	1	0.005122
1	dbsnp	rs4648845	A	G	ALLELIC	15/5	19/31	7.829	1	0.005141
1	dbsnp	rs6427268	G	A	ALLELIC	15/5	19/31	7.829	1	0.005141
1	dbsnp	rs12144096	G	A	ALLELIC	15/5	19/31	7.829	1	0.005141
1	dbsnp	rs2050664	G	A	ALLELIC	15/5	19/31	7.829	1	0.005141
2	dbsnp	rs13390757	G	A	ALLELIC	5/15	31/19	7.829	1	0.005141
3	dbsnp	rs4624520	G	A	ALLELIC	5/15	31/19	7.829	1	0.005141
4	dbsnp	rs1381737	C	A	ALLELIC	15/5	19/31	7.829	1	0.005141
4	dbsnp	rs1560982	G	A	ALLELIC	15/5	19/31	7.829	1	0.005141
5	dbsnp	rs11746179	G	A	ALLELIC	15/5	19/31	7.829	1	0.005141
6	dbsnp	rs516473	C	A	ALLELIC	15/5	19/31	7.829	1	0.005141
7	dbsnp	rs10270076	G	A	ALLELIC	5/15	31/19	7.829	1	0.005141
9	dbsnp	rs4524899	G	A	ALLELIC	15/5	19/31	7.829	1	0.005141
11	dbsnp	rs10895000	A	G	ALLELIC	15/5	19/31	7.829	1	0.005141

11	dbsnp	rs10789746	A	G	ALLELIC	5/15	31/19	7.829	1	0.005141
14	dbsnp	rs8003133	G	A	ALLELIC	15/5	19/31	7.829	1	0.005141
14	dbsnp	rs8020137	G	A	ALLELIC	15/5	19/31	7.829	1	0.005141
14	dbsnp	rs7492545	G	A	ALLELIC	5/15	31/19	7.829	1	0.005141
14	dbsnp	rs2022771	G	A	ALLELIC	15/5	19/31	7.829	1	0.005141
16	dbsnp	rs2352934	A	G	ALLELIC	15/5	19/31	7.829	1	0.005141
21	dbsnp	rs2142090	G	A	ALLELIC	5/15	31/19	7.829	1	0.005141
23	dbsnp	rs7053243	G	A	ALLELIC	15/5	19/31	7.829	1	0.005141
3	dbsnp	rs492310	A	G	ALLELIC	10/12	7/41	7.819	1	0.00517
11	dbsnp	rs11600121	C	A	ALLELIC	10/12	7/41	7.819	1	0.00517
23	dbsnp	rs5986271	A	G	ALLELIC	10/12	7/41	7.819	1	0.00517
1	dbsnp	rs12563394	G	A	ALLELIC	11/11	9/41	7.798	1	0.00523
1	dbsnp	rs2301460	C	A	ALLELIC	11/11	9/41	7.798	1	0.00523
1	dbsnp	rs10889070	A	G	ALLELIC	11/11	9/41	7.798	1	0.00523
1	dbsnp	rs12565558	G	A	ALLELIC	11/11	9/41	7.798	1	0.00523
1	dbsnp	rs6667611	A	G	ALLELIC	11/11	9/41	7.798	1	0.00523
1	dbsnp	rs6687300	C	A	ALLELIC	11/11	9/41	7.798	1	0.00523
1	dbsnp	rs17163203	G	A	ALLELIC	11/11	9/41	7.798	1	0.00523
2	dbsnp	rs4594412	A	G	ALLELIC	11/11	9/41	7.798	1	0.00523
2	dbsnp	rs4954585	G	A	ALLELIC	11/11	9/41	7.798	1	0.00523
2	dbsnp	rs4954599	G	A	ALLELIC	11/11	9/41	7.798	1	0.00523
2	dbsnp	rs10931792	G	A	ALLELIC	11/11	9/41	7.798	1	0.00523
3	dbsnp	rs2657606	G	A	ALLELIC	11/11	9/41	7.798	1	0.00523
3	dbsnp	rs9809150	A	G	ALLELIC	11/11	9/41	7.798	1	0.00523
3	dbsnp	rs9854304	A	G	ALLELIC	11/11	9/41	7.798	1	0.00523
3	dbsnp	rs2292130	G	A	ALLELIC	11/11	9/41	7.798	1	0.00523
4	dbsnp	rs10011864	A	G	ALLELIC	11/11	9/41	7.798	1	0.00523
4	dbsnp	rs6816525	A	G	ALLELIC	11/11	9/41	7.798	1	0.00523
5	dbsnp	rs3110984	G	A	ALLELIC	11/11	9/41	7.798	1	0.00523
5	dbsnp	rs4547878	G	A	ALLELIC	11/11	9/41	7.798	1	0.00523
5	dbsnp	rs964986	A	G	ALLELIC	11/11	9/41	7.798	1	0.00523
5	dbsnp	rs17387218	G	A	ALLELIC	11/11	9/41	7.798	1	0.00523
5	dbsnp	rs4921496	A	G	ALLELIC	11/11	9/41	7.798	1	0.00523
6	dbsnp	rs2844509	G	A	ALLELIC	11/11	9/41	7.798	1	0.00523
6	dbsnp	rs13214308	G	A	ALLELIC	11/11	9/41	7.798	1	0.00523
6	dbsnp	rs12523848	A	G	ALLELIC	11/11	9/41	7.798	1	0.00523
6	dbsnp	rs2803358	A	G	ALLELIC	11/11	9/41	7.798	1	0.00523
6	dbsnp	rs569919	A	G	ALLELIC	11/11	9/41	7.798	1	0.00523
7	dbsnp	rs9638987	A	G	ALLELIC	11/11	9/41	7.798	1	0.00523
7	dbsnp	rs13224928	G	A	ALLELIC	11/11	9/41	7.798	1	0.00523
7	dbsnp	rs10953315	G	A	ALLELIC	11/11	9/41	7.798	1	0.00523
7	dbsnp	rs17829622	C	A	ALLELIC	11/11	9/41	7.798	1	0.00523
8	dbsnp	rs10091907	A	G	ALLELIC	11/11	9/41	7.798	1	0.00523
8	dbsnp	rs883227	A	G	ALLELIC	11/11	9/41	7.798	1	0.00523
8	dbsnp	rs4571700	A	G	ALLELIC	11/11	9/41	7.798	1	0.00523
8	dbsnp	rs7012851	A	G	ALLELIC	11/11	9/41	7.798	1	0.00523
8	dbsnp	rs7013213	A	C	ALLELIC	11/11	9/41	7.798	1	0.00523
9	dbsnp	rs2381871	A	G	ALLELIC	11/11	9/41	7.798	1	0.00523

9	dbsnp	rs879857	A	G	ALLELIC	11/11	9/41	7.798	1	0.00523
9	dbsnp	rs7856532	C	A	ALLELIC	11/11	9/41	7.798	1	0.00523
9	dbsnp	rs1323433	A	G	ALLELIC	11/11	9/41	7.798	1	0.00523
9	dbsnp	rs7858998	A	G	ALLELIC	11/11	9/41	7.798	1	0.00523
10	dbsnp	rs7903338	A	C	ALLELIC	11/11	9/41	7.798	1	0.00523
10	dbsnp	rs1394495	G	A	ALLELIC	11/11	9/41	7.798	1	0.00523
10	dbsnp	rs4357606	G	A	ALLELIC	11/11	9/41	7.798	1	0.00523
11	dbsnp	rs1714338	A	G	ALLELIC	11/11	9/41	7.798	1	0.00523
12	dbsnp	rs2887780	G	A	ALLELIC	11/11	9/41	7.798	1	0.00523
12	dbsnp	rs10841939	A	C	ALLELIC	11/11	9/41	7.798	1	0.00523
12	dbsnp	rs1432113	G	A	ALLELIC	11/11	9/41	7.798	1	0.00523
12	dbsnp	rs2651882	A	G	ALLELIC	11/11	9/41	7.798	1	0.00523
13	dbsnp	rs9572262	G	A	ALLELIC	11/11	9/41	7.798	1	0.00523
13	dbsnp	rs9317845	G	A	ALLELIC	11/11	9/41	7.798	1	0.00523
13	dbsnp	rs1218307	G	A	ALLELIC	11/11	9/41	7.798	1	0.00523
13	dbsnp	rs1927364	G	A	ALLELIC	11/11	9/41	7.798	1	0.00523
13	dbsnp	rs7336772	G	A	ALLELIC	11/11	9/41	7.798	1	0.00523
13	dbsnp	rs7987644	G	A	ALLELIC	11/11	9/41	7.798	1	0.00523
14	dbsnp	rs10498358	A	G	ALLELIC	11/11	9/41	7.798	1	0.00523
15	dbsnp	rs687209	A	G	ALLELIC	11/11	9/41	7.798	1	0.00523
15	dbsnp	rs11072035	A	G	ALLELIC	11/11	9/41	7.798	1	0.00523
16	dbsnp	rs1532167	A	C	ALLELIC	11/11	9/41	7.798	1	0.00523
17	dbsnp	rs11654578	A	C	ALLELIC	11/11	9/41	7.798	1	0.00523
18	dbsnp	rs4798363	A	G	ALLELIC	11/11	9/41	7.798	1	0.00523
18	dbsnp	rs9320006	A	G	ALLELIC	11/11	9/41	7.798	1	0.00523
19	dbsnp	rs2918294	A	G	ALLELIC	11/11	9/41	7.798	1	0.00523
19	dbsnp	rs2910344	A	G	ALLELIC	11/11	9/41	7.798	1	0.00523
20	dbsnp	rs28698842	A	G	ALLELIC	11/11	9/41	7.798	1	0.00523
22	dbsnp	rs134609	G	A	ALLELIC	11/11	9/41	7.798	1	0.00523
22	dbsnp	rs2111833	A	G	ALLELIC	11/11	9/41	7.798	1	0.00523
22	dbsnp	rs9612080	A	G	ALLELIC	11/11	9/41	7.798	1	0.00523
23	dbsnp	rs11798513	C	A	ALLELIC	11/11	9/41	7.798	1	0.00523
23	dbsnp	rs7055256	C	A	ALLELIC	11/11	9/41	7.798	1	0.00523
23	dbsnp	rs2158035	G	A	ALLELIC	11/11	9/41	7.798	1	0.00523
23	dbsnp	rs6653670	G	A	ALLELIC	11/11	9/41	7.798	1	0.00523
23	dbsnp	rs5925968	C	A	ALLELIC	11/11	9/41	7.798	1	0.00523
23	dbsnp	rs3813165	G	A	ALLELIC	11/11	9/41	7.798	1	0.00523
23	dbsnp	rs4828786	A	C	ALLELIC	11/11	9/41	7.798	1	0.00523
1	dbsnp	rs7547836	C	A	ALLELIC	9/11	7/43	7.786	1	0.005266
1	dbsnp	rs2143583	C	A	ALLELIC	9/11	7/43	7.786	1	0.005266
1	dbsnp	rs6427193	G	A	ALLELIC	9/11	7/43	7.786	1	0.005266
1	dbsnp	rs6427832	A	G	ALLELIC	9/11	7/43	7.786	1	0.005266
1	dbsnp	rs10863529	G	A	ALLELIC	9/11	7/43	7.786	1	0.005266
4	dbsnp	rs7694207	A	G	ALLELIC	9/11	7/43	7.786	1	0.005266
5	dbsnp	rs10075239	A	C	ALLELIC	9/11	7/43	7.786	1	0.005266
5	dbsnp	rs1458513	A	G	ALLELIC	9/11	7/43	7.786	1	0.005266
6	dbsnp	rs10944649	A	G	ALLELIC	9/11	7/43	7.786	1	0.005266
7	dbsnp	rs10270418	A	G	ALLELIC	9/11	7/43	7.786	1	0.005266

7	dbsnp	rs6951864	A	G	ALLELIC	9/11	7/43	7.786	1	0.005266
8	dbsnp	rs2012106	C	A	ALLELIC	9/11	7/43	7.786	1	0.005266
10	dbsnp	rs12357686	A	G	ALLELIC	9/11	7/43	7.786	1	0.005266
10	dbsnp	rs7909809	A	G	ALLELIC	9/11	7/43	7.786	1	0.005266
10	dbsnp	rs6571242	G	A	ALLELIC	9/11	7/43	7.786	1	0.005266
11	dbsnp	rs6762	G	A	ALLELIC	9/11	7/43	7.786	1	0.005266
11	dbsnp	rs10902227	A	G	ALLELIC	9/11	7/43	7.786	1	0.005266
11	dbsnp	rs11024797	G	A	ALLELIC	9/11	7/43	7.786	1	0.005266
11	dbsnp	rs10790175	A	G	ALLELIC	9/11	7/43	7.786	1	0.005266
11	dbsnp	rs8521	G	A	ALLELIC	9/11	7/43	7.786	1	0.005266
12	dbsnp	rs1386488	C	A	ALLELIC	9/11	7/43	7.786	1	0.005266
12	dbsnp	rs1406620	A	G	ALLELIC	9/11	7/43	7.786	1	0.005266
12	dbsnp	rs2701810	A	G	ALLELIC	9/11	7/43	7.786	1	0.005266
13	dbsnp	rs12430011	G	A	ALLELIC	9/11	7/43	7.786	1	0.005266
18	dbsnp	rs6508395	C	A	ALLELIC	9/11	7/43	7.786	1	0.005266
18	dbsnp	rs4891051	A	G	ALLELIC	9/11	7/43	7.786	1	0.005266
23	dbsnp	rs766775	T	A	ALLELIC	9/11	7/43	7.786	1	0.005266
2	dbsnp	rs7603003	G	A	ALLELIC	1/21	18/32	7.781	1	0.005278
2	dbsnp	rs298259	A	G	ALLELIC	1/21	18/32	7.781	1	0.005278
2	dbsnp	rs602291	C	A	ALLELIC	1/21	18/32	7.781	1	0.005278
3	dbsnp	rs10513840	C	A	ALLELIC	1/21	18/32	7.781	1	0.005278
4	dbsnp	rs4698773	A	C	ALLELIC	1/21	18/32	7.781	1	0.005278
5	dbsnp	rs26436	A	G	ALLELIC	1/21	18/32	7.781	1	0.005278
6	dbsnp	rs1361385	G	A	ALLELIC	1/21	18/32	7.781	1	0.005278
6	dbsnp	rs6907950	A	G	ALLELIC	1/21	18/32	7.781	1	0.005278
6	dbsnp	rs6899389	A	C	ALLELIC	1/21	18/32	7.781	1	0.005278
6	dbsnp	rs2071965	A	G	ALLELIC	1/21	18/32	7.781	1	0.005278
6	dbsnp	rs6931820	A	G	ALLELIC	1/21	18/32	7.781	1	0.005278
7	dbsnp	rs890635	A	G	ALLELIC	1/21	18/32	7.781	1	0.005278
7	dbsnp	rs1729851	C	A	ALLELIC	1/21	18/32	7.781	1	0.005278
7	dbsnp	rs1529001	A	G	ALLELIC	1/21	18/32	7.781	1	0.005278
7	dbsnp	rs1649704	A	G	ALLELIC	1/21	18/32	7.781	1	0.005278
8	dbsnp	rs6981912	G	A	ALLELIC	1/21	18/32	7.781	1	0.005278
9	dbsnp	rs11141716	C	A	ALLELIC	1/21	18/32	7.781	1	0.005278
9	dbsnp	rs16916507	A	G	ALLELIC	1/21	18/32	7.781	1	0.005278
10	dbsnp	rs7906088	A	G	ALLELIC	1/21	18/32	7.781	1	0.005278
10	dbsnp	rs7908745	G	A	ALLELIC	1/21	18/32	7.781	1	0.005278
10	dbsnp	rs10900222	A	C	ALLELIC	1/21	18/32	7.781	1	0.005278
10	dbsnp	rs7894232	A	G	ALLELIC	1/21	18/32	7.781	1	0.005278
10	dbsnp	rs12778761	A	C	ALLELIC	1/21	18/32	7.781	1	0.005278
11	dbsnp	rs1501466	G	A	ALLELIC	1/21	18/32	7.781	1	0.005278
12	dbsnp	rs12578472	A	G	ALLELIC	1/21	18/32	7.781	1	0.005278
14	dbsnp	rs1977131	A	G	ALLELIC	1/21	18/32	7.781	1	0.005278
14	dbsnp	rs17110896	A	G	ALLELIC	1/21	18/32	7.781	1	0.005278
14	dbsnp	rs12882548	G	A	ALLELIC	1/21	18/32	7.781	1	0.005278
14	dbsnp	rs1609699	A	G	ALLELIC	1/21	18/32	7.781	1	0.005278
18	dbsnp	rs6566993	C	A	ALLELIC	1/21	18/32	7.781	1	0.005278
19	dbsnp	rs34463842	C	A	ALLELIC	1/21	18/32	7.781	1	0.005278

20	dbsnp	rs2423013	A	G	ALLELIC	1/21	18/32	7.781	1	0.005278
1	dbsnp	rs10492930	G	A	ALLELIC	9/13	6/44	7.742	1	0.005396
1	dbsnp	rs924088	G	A	ALLELIC	9/13	6/44	7.742	1	0.005396
1	dbsnp	rs10912467	G	A	ALLELIC	9/13	6/44	7.742	1	0.005396
1	dbsnp	rs12078733	G	A	ALLELIC	9/13	6/44	7.742	1	0.005396
2	dbsnp	rs13414125	A	G	ALLELIC	9/13	6/44	7.742	1	0.005396
2	dbsnp	rs4953640	A	G	ALLELIC	9/13	6/44	7.742	1	0.005396
2	dbsnp	rs6761675	A	G	ALLELIC	9/13	6/44	7.742	1	0.005396
2	dbsnp	rs3770016	A	G	ALLELIC	9/13	6/44	7.742	1	0.005396
2	dbsnp	rs262283	G	A	ALLELIC	9/13	6/44	7.742	1	0.005396
2	dbsnp	rs6715945	G	A	ALLELIC	9/13	6/44	7.742	1	0.005396
2	dbsnp	rs7574670	G	A	ALLELIC	9/13	6/44	7.742	1	0.005396
3	dbsnp	rs2067745	A	G	ALLELIC	9/13	6/44	7.742	1	0.005396
3	dbsnp	rs7627013	A	G	ALLELIC	9/13	6/44	7.742	1	0.005396
3	dbsnp	rs622205	A	G	ALLELIC	9/13	6/44	7.742	1	0.005396
4	dbsnp	rs10938939	A	G	ALLELIC	9/13	6/44	7.742	1	0.005396
4	dbsnp	rs12507992	A	G	ALLELIC	9/13	6/44	7.742	1	0.005396
4	dbsnp	rs7697688	A	G	ALLELIC	9/13	6/44	7.742	1	0.005396
5	dbsnp	rs6884756	A	G	ALLELIC	9/13	6/44	7.742	1	0.005396
5	dbsnp	rs9292273	G	A	ALLELIC	9/13	6/44	7.742	1	0.005396
5	dbsnp	rs11242026	A	G	ALLELIC	9/13	6/44	7.742	1	0.005396
5	dbsnp	rs2071156	A	G	ALLELIC	9/13	6/44	7.742	1	0.005396
5	dbsnp	rs949845	A	C	ALLELIC	9/13	6/44	7.742	1	0.005396
6	dbsnp	rs478791	A	G	ALLELIC	9/13	6/44	7.742	1	0.005396
6	dbsnp	rs1872241	A	G	ALLELIC	9/13	6/44	7.742	1	0.005396
6	dbsnp	rs7769759	G	A	ALLELIC	9/13	6/44	7.742	1	0.005396
6	dbsnp	rs2022212	G	A	ALLELIC	9/13	6/44	7.742	1	0.005396
6	dbsnp	rs4707265	A	C	ALLELIC	9/13	6/44	7.742	1	0.005396
6	dbsnp	rs2211493	C	A	ALLELIC	9/13	6/44	7.742	1	0.005396
6	dbsnp	rs12190656	G	A	ALLELIC	9/13	6/44	7.742	1	0.005396
6	dbsnp	rs9356236	G	A	ALLELIC	9/13	6/44	7.742	1	0.005396
7	dbsnp	rs2190066	G	A	ALLELIC	9/13	6/44	7.742	1	0.005396
7	dbsnp	rs12537544	G	A	ALLELIC	9/13	6/44	7.742	1	0.005396
7	dbsnp	rs677014	C	A	ALLELIC	9/13	6/44	7.742	1	0.005396
7	dbsnp	rs1242778	A	G	ALLELIC	9/13	6/44	7.742	1	0.005396
9	dbsnp	rs10978077	G	A	ALLELIC	9/13	6/44	7.742	1	0.005396
9	dbsnp	rs10759100	G	A	ALLELIC	9/13	6/44	7.742	1	0.005396
9	dbsnp	rs10759101	A	G	ALLELIC	9/13	6/44	7.742	1	0.005396
9	dbsnp	rs10978079	A	G	ALLELIC	9/13	6/44	7.742	1	0.005396
9	dbsnp	rs7047163	G	A	ALLELIC	9/13	6/44	7.742	1	0.005396
9	dbsnp	rs4977746	G	A	ALLELIC	9/13	6/44	7.742	1	0.005396
9	dbsnp	rs13288748	A	G	ALLELIC	9/13	6/44	7.742	1	0.005396
9	dbsnp	rs4877115	A	C	ALLELIC	9/13	6/44	7.742	1	0.005396
9	dbsnp	rs3847304	G	A	ALLELIC	9/13	6/44	7.742	1	0.005396
10	dbsnp	rs10826682	A	G	ALLELIC	9/13	6/44	7.742	1	0.005396
10	dbsnp	rs6480917	A	G	ALLELIC	9/13	6/44	7.742	1	0.005396
11	dbsnp	rs16910122	A	G	ALLELIC	9/13	6/44	7.742	1	0.005396
11	dbsnp	rs11037545	A	G	ALLELIC	9/13	6/44	7.742	1	0.005396

11	dbsnp	rs11037567	G	A	ALLELIC	9/13	6/44	7.742	1	0.005396
11	dbsnp	rs1042838	A	C	ALLELIC	9/13	6/44	7.742	1	0.005396
12	dbsnp	rs2058349	G	A	ALLELIC	9/13	6/44	7.742	1	0.005396
12	dbsnp	rs10492111	A	G	ALLELIC	9/13	6/44	7.742	1	0.005396
12	dbsnp	rs2728609	A	G	ALLELIC	9/13	6/44	7.742	1	0.005396
12	dbsnp	rs10083205	G	A	ALLELIC	9/13	6/44	7.742	1	0.005396
12	dbsnp	rs7975712	G	A	ALLELIC	9/13	6/44	7.742	1	0.005396
12	dbsnp	rs11169063	G	A	ALLELIC	9/13	6/44	7.742	1	0.005396
12	dbsnp	rs11107531	A	G	ALLELIC	9/13	6/44	7.742	1	0.005396
12	dbsnp	rs12307812	A	G	ALLELIC	9/13	6/44	7.742	1	0.005396
13	dbsnp	rs7995390	A	G	ALLELIC	9/13	6/44	7.742	1	0.005396
13	dbsnp	rs9540775	G	A	ALLELIC	9/13	6/44	7.742	1	0.005396
13	dbsnp	rs9518279	A	C	ALLELIC	9/13	6/44	7.742	1	0.005396
14	dbsnp	rs9635224	C	A	ALLELIC	9/13	6/44	7.742	1	0.005396
14	dbsnp	rs10140556	A	G	ALLELIC	9/13	6/44	7.742	1	0.005396
14	dbsnp	rs881471	A	G	ALLELIC	9/13	6/44	7.742	1	0.005396
14	dbsnp	rs7149810	G	A	ALLELIC	9/13	6/44	7.742	1	0.005396
14	dbsnp	rs2057368	A	G	ALLELIC	9/13	6/44	7.742	1	0.005396
14	dbsnp	rs17128004	A	C	ALLELIC	9/13	6/44	7.742	1	0.005396
14	dbsnp	rs2878168	A	G	ALLELIC	9/13	6/44	7.742	1	0.005396
14	dbsnp	rs2623142	A	G	ALLELIC	9/13	6/44	7.742	1	0.005396
15	dbsnp	rs11629630	G	A	ALLELIC	9/13	6/44	7.742	1	0.005396
15	dbsnp	rs11636318	G	A	ALLELIC	9/13	6/44	7.742	1	0.005396
15	dbsnp	rs4278705	G	A	ALLELIC	9/13	6/44	7.742	1	0.005396
15	dbsnp	rs7170663	A	G	ALLELIC	9/13	6/44	7.742	1	0.005396
16	dbsnp	rs11642055	A	G	ALLELIC	9/13	6/44	7.742	1	0.005396
16	dbsnp	rs4473203	A	G	ALLELIC	9/13	6/44	7.742	1	0.005396
17	dbsnp	rs228285	A	G	ALLELIC	9/13	6/44	7.742	1	0.005396
17	dbsnp	rs228289	C	A	ALLELIC	9/13	6/44	7.742	1	0.005396
17	dbsnp	rs2586233	A	G	ALLELIC	9/13	6/44	7.742	1	0.005396
17	dbsnp	rs744667	G	A	ALLELIC	9/13	6/44	7.742	1	0.005396
17	dbsnp	rs4789155	A	G	ALLELIC	9/13	6/44	7.742	1	0.005396
17	dbsnp	rs877874	G	A	ALLELIC	9/13	6/44	7.742	1	0.005396
18	dbsnp	rs717183	A	G	ALLELIC	9/13	6/44	7.742	1	0.005396
18	dbsnp	rs28370576	G	A	ALLELIC	9/13	6/44	7.742	1	0.005396
20	dbsnp	rs2284374	A	G	ALLELIC	9/13	6/44	7.742	1	0.005396
20	dbsnp	rs376852	A	G	ALLELIC	9/13	6/44	7.742	1	0.005396
21	dbsnp	rs2836436	G	A	ALLELIC	9/13	6/44	7.742	1	0.005396
22	dbsnp	rs1997739	G	A	ALLELIC	9/13	6/44	7.742	1	0.005396
22	dbsnp	rs5999368	G	A	ALLELIC	9/13	6/44	7.742	1	0.005396
22	dbsnp	rs910570	G	A	ALLELIC	9/13	6/44	7.742	1	0.005396
23	dbsnp	rs4826792	G	A	ALLELIC	9/13	6/44	7.742	1	0.005396
23	dbsnp	rs5922584	G	A	ALLELIC	9/13	6/44	7.742	1	0.005396
23	dbsnp	rs4548319	C	A	ALLELIC	9/13	6/44	7.742	1	0.005396
2	dbsnp	rs10194544	A	G	ALLELIC	5/17	27/19	7.728	1	0.005436
2	dbsnp	rs1515110	C	A	ALLELIC	11/9	10/38	7.721	1	0.005459
8	dbsnp	rs2570668	G	A	ALLELIC	11/9	10/38	7.721	1	0.005459
23	dbsnp	rs5977586	A	G	ALLELIC	3/15	0/44	7.706	1	0.005503

1	dbsnp	rs1295085	A	G	ALLELIC	3/19	24/26	7.697	1	0.00553
1	dbsnp	rs2811314	G	A	ALLELIC	3/19	24/26	7.697	1	0.00553
1	dbsnp	rs7538400	A	G	ALLELIC	3/19	24/26	7.697	1	0.00553
2	dbsnp	rs3755166	A	G	ALLELIC	3/19	24/26	7.697	1	0.00553
2	dbsnp	rs12616969	G	A	ALLELIC	3/19	24/26	7.697	1	0.00553
4	dbsnp	rs28590539	A	G	ALLELIC	3/19	24/26	7.697	1	0.00553
4	dbsnp	rs10518139	G	A	ALLELIC	3/19	24/26	7.697	1	0.00553
4	dbsnp	rs10020976	A	G	ALLELIC	3/19	24/26	7.697	1	0.00553
4	dbsnp	rs17051169	A	G	ALLELIC	3/19	24/26	7.697	1	0.00553
6	dbsnp	rs9465608	A	G	ALLELIC	3/19	24/26	7.697	1	0.00553
6	dbsnp	rs9368359	C	A	ALLELIC	3/19	24/26	7.697	1	0.00553
6	dbsnp	rs1936019	A	G	ALLELIC	3/19	24/26	7.697	1	0.00553
6	dbsnp	rs2142642	G	A	ALLELIC	3/19	24/26	7.697	1	0.00553
6	dbsnp	rs1345990	A	G	ALLELIC	3/19	24/26	7.697	1	0.00553
6	dbsnp	rs7747720	A	G	ALLELIC	3/19	24/26	7.697	1	0.00553
6	dbsnp	rs4708431	G	A	ALLELIC	3/19	24/26	7.697	1	0.00553
7	dbsnp	rs1174864	G	A	ALLELIC	3/19	24/26	7.697	1	0.00553
7	dbsnp	rs1474383	A	G	ALLELIC	3/19	24/26	7.697	1	0.00553
9	dbsnp	rs7872188	A	G	ALLELIC	3/19	24/26	7.697	1	0.00553
10	dbsnp	rs4462243	G	A	ALLELIC	3/19	24/26	7.697	1	0.00553
10	dbsnp	rs7905167	A	G	ALLELIC	3/19	24/26	7.697	1	0.00553
10	dbsnp	rs2398215	A	G	ALLELIC	3/19	24/26	7.697	1	0.00553
11	dbsnp	rs374544	G	A	ALLELIC	3/19	24/26	7.697	1	0.00553
11	dbsnp	rs11606370	C	A	ALLELIC	3/19	24/26	7.697	1	0.00553
11	dbsnp	rs10897526	G	A	ALLELIC	3/19	24/26	7.697	1	0.00553
11	dbsnp	rs10894727	A	G	ALLELIC	3/19	24/26	7.697	1	0.00553
12	dbsnp	rs11116456	A	G	ALLELIC	3/19	24/26	7.697	1	0.00553
12	dbsnp	rs7959985	A	G	ALLELIC	3/19	24/26	7.697	1	0.00553
12	dbsnp	rs10745722	A	G	ALLELIC	3/19	24/26	7.697	1	0.00553
12	dbsnp	rs4525299	A	G	ALLELIC	3/19	24/26	7.697	1	0.00553
13	dbsnp	rs7328493	C	A	ALLELIC	3/19	24/26	7.697	1	0.00553
13	dbsnp	rs3910315	A	G	ALLELIC	3/19	24/26	7.697	1	0.00553
14	dbsnp	rs1255884	A	G	ALLELIC	3/19	24/26	7.697	1	0.00553
15	dbsnp	rs16961209	A	G	ALLELIC	3/19	24/26	7.697	1	0.00553
16	dbsnp	rs299993	C	A	ALLELIC	3/19	24/26	7.697	1	0.00553
17	dbsnp	rs9896540	A	G	ALLELIC	3/19	24/26	7.697	1	0.00553
18	dbsnp	rs12959692	G	A	ALLELIC	3/19	24/26	7.697	1	0.00553
20	dbsnp	rs6040881	G	A	ALLELIC	3/19	24/26	7.697	1	0.00553
20	dbsnp	rs6040901	A	G	ALLELIC	3/19	24/26	7.697	1	0.00553
20	dbsnp	rs6089354	G	A	ALLELIC	3/19	24/26	7.697	1	0.00553
21	dbsnp	rs2839084	A	G	ALLELIC	3/19	24/26	7.697	1	0.00553
21	dbsnp	rs2070426	G	C	ALLELIC	3/19	24/26	7.697	1	0.00553
22	dbsnp	rs6000213	G	A	ALLELIC	3/19	24/26	7.697	1	0.00553
23	dbsnp	rs3087965	T	A	ALLELIC	3/19	24/26	7.697	1	0.00553
23	dbsnp	rs5935953	A	G	ALLELIC	3/19	24/26	7.697	1	0.00553
23	dbsnp	rs6527487	A	G	ALLELIC	3/19	24/26	7.697	1	0.00553
1	dbsnp	rs4907133	A	G	ALLELIC	0/22	14/36	7.647	1	0.005687
1	dbsnp	rs7511717	A	G	ALLELIC	0/22	14/36	7.647	1	0.005687

2	dbsnp	rs1009024	C	A	ALLELIC	0/22	14/36	7.647	1	0.005687
2	dbsnp	rs2541194	A	G	ALLELIC	0/22	14/36	7.647	1	0.005687
2	dbsnp	rs6745103	G	A	ALLELIC	0/22	14/36	7.647	1	0.005687
2	dbsnp	rs12996082	A	G	ALLELIC	0/22	14/36	7.647	1	0.005687
2	dbsnp	rs1430112	G	A	ALLELIC	0/22	14/36	7.647	1	0.005687
2	dbsnp	rs6738538	A	G	ALLELIC	0/22	14/36	7.647	1	0.005687
2	dbsnp	rs4663563	A	G	ALLELIC	0/22	14/36	7.647	1	0.005687
3	dbsnp	rs9847827	A	G	ALLELIC	0/22	14/36	7.647	1	0.005687
3	dbsnp	rs9819444	C	A	ALLELIC	0/22	14/36	7.647	1	0.005687
3	dbsnp	rs16849947	G	A	ALLELIC	0/22	14/36	7.647	1	0.005687
4	dbsnp	rs11733542	A	G	ALLELIC	0/22	14/36	7.647	1	0.005687
4	dbsnp	rs12499997	A	G	ALLELIC	0/22	14/36	7.647	1	0.005687
4	dbsnp	rs3866838	A	G	ALLELIC	0/22	14/36	7.647	1	0.005687
5	dbsnp	rs7713448	A	C	ALLELIC	0/22	14/36	7.647	1	0.005687
5	dbsnp	rs2306826	A	G	ALLELIC	0/22	14/36	7.647	1	0.005687
6	dbsnp	rs2230654	A	G	ALLELIC	0/22	14/36	7.647	1	0.005687
6	dbsnp	rs11751732	A	G	ALLELIC	0/22	14/36	7.647	1	0.005687
6	dbsnp	rs9353681	A	G	ALLELIC	0/22	14/36	7.647	1	0.005687
6	dbsnp	rs9376889	G	A	ALLELIC	0/22	14/36	7.647	1	0.005687
7	dbsnp	rs4721197	G	A	ALLELIC	0/22	14/36	7.647	1	0.005687
7	dbsnp	rs2068399	A	G	ALLELIC	0/22	14/36	7.647	1	0.005687
7	dbsnp	rs4520105	G	A	ALLELIC	0/22	14/36	7.647	1	0.005687
7	dbsnp	rs17164727	A	C	ALLELIC	0/22	14/36	7.647	1	0.005687
8	dbsnp	rs1607910	G	A	ALLELIC	0/22	14/36	7.647	1	0.005687
11	dbsnp	rs2397351	A	C	ALLELIC	0/22	14/36	7.647	1	0.005687
12	dbsnp	rs7134252	G	A	ALLELIC	0/22	14/36	7.647	1	0.005687
12	dbsnp	rs1155392	C	A	ALLELIC	0/22	14/36	7.647	1	0.005687
12	dbsnp	rs4931393	A	C	ALLELIC	0/22	14/36	7.647	1	0.005687
12	dbsnp	rs2352616	A	G	ALLELIC	0/22	14/36	7.647	1	0.005687
13	dbsnp	rs12020349	C	A	ALLELIC	0/22	14/36	7.647	1	0.005687
14	dbsnp	rs222717	G	A	ALLELIC	0/22	14/36	7.647	1	0.005687
14	dbsnp	rs1968577	A	G	ALLELIC	0/22	14/36	7.647	1	0.005687
15	dbsnp	rs16947804	C	A	ALLELIC	0/22	14/36	7.647	1	0.005687
17	dbsnp	rs4794318	A	G	ALLELIC	0/22	14/36	7.647	1	0.005687
17	dbsnp	rs11652334	G	A	ALLELIC	0/22	14/36	7.647	1	0.005687
20	dbsnp	rs6039012	C	A	ALLELIC	0/22	14/36	7.647	1	0.005687
23	dbsnp	rs5968924	G	A	ALLELIC	0/22	14/36	7.647	1	0.005687
23	dbsnp	rs5968931	G	A	ALLELIC	0/22	14/36	7.647	1	0.005687
23	dbsnp	rs6623736	G	A	ALLELIC	0/22	14/36	7.647	1	0.005687
23	dbsnp	rs12011482	A	C	ALLELIC	0/22	14/36	7.647	1	0.005687
11	dbsnp	rs11603047	C	A	ALLELIC	15/7	15/31	7.639	1	0.005712
2	dbsnp	rs7607360	G	A	ALLELIC	0/20	15/35	7.636	1	0.00572
2	dbsnp	rs17610130	G	A	ALLELIC	0/20	15/35	7.636	1	0.00572
4	dbsnp	rs4148295	C	A	ALLELIC	13/9	12/36	7.636	1	0.00572
4	dbsnp	rs13143261	G	A	ALLELIC	0/20	15/35	7.636	1	0.00572
5	dbsnp	rs2956581	A	G	ALLELIC	0/20	15/35	7.636	1	0.00572
7	dbsnp	rs953143	G	A	ALLELIC	0/20	15/35	7.636	1	0.00572
8	dbsnp	rs10755941	G	A	ALLELIC	13/9	12/36	7.636	1	0.00572

9	dbsnp	rs653030	C	A	ALLELIC	0/20	15/35	7.636	1	0.00572
10	dbsnp	rs11245315	A	G	ALLELIC	13/9	12/36	7.636	1	0.00572
11	dbsnp	rs1258854	A	G	ALLELIC	13/9	12/36	7.636	1	0.00572
12	dbsnp	rs6487504	G	A	ALLELIC	0/20	15/35	7.636	1	0.00572
14	dbsnp	rs12432461	A	G	ALLELIC	0/20	15/35	7.636	1	0.00572
14	dbsnp	rs11158828	G	A	ALLELIC	0/20	15/35	7.636	1	0.00572
17	dbsnp	rs16942199	C	A	ALLELIC	13/9	12/36	7.636	1	0.00572
1	dbsnp	rs875807	A	G	ALLELIC	5/17	29/21	7.627	1	0.005751
1	dbsnp	rs6700115	A	G	ALLELIC	5/17	29/21	7.627	1	0.005751
1	dbsnp	rs1970625	A	G	ALLELIC	5/17	29/21	7.627	1	0.005751
2	dbsnp	rs6705765	G	A	ALLELIC	5/17	29/21	7.627	1	0.005751
2	dbsnp	rs207946	G	A	ALLELIC	5/17	29/21	7.627	1	0.005751
3	dbsnp	rs930567	A	G	ALLELIC	5/17	29/21	7.627	1	0.005751
4	dbsnp	rs1027473	A	C	ALLELIC	5/17	29/21	7.627	1	0.005751
4	dbsnp	rs10030678	G	A	ALLELIC	5/17	29/21	7.627	1	0.005751
4	dbsnp	rs1706145	A	C	ALLELIC	5/17	29/21	7.627	1	0.005751
5	dbsnp	rs833344	A	G	ALLELIC	5/17	29/21	7.627	1	0.005751
5	dbsnp	rs286734	C	A	ALLELIC	5/17	29/21	7.627	1	0.005751
5	dbsnp	rs2909707	A	G	ALLELIC	5/17	29/21	7.627	1	0.005751
6	dbsnp	rs394657	G	A	ALLELIC	17/5	21/29	7.627	1	0.005751
6	dbsnp	rs1744493	G	A	ALLELIC	5/17	29/21	7.627	1	0.005751
8	dbsnp	rs2740939	A	C	ALLELIC	5/17	29/21	7.627	1	0.005751
8	dbsnp	rs1964328	G	A	ALLELIC	5/17	29/21	7.627	1	0.005751
8	dbsnp	rs6999447	G	A	ALLELIC	5/17	29/21	7.627	1	0.005751
8	dbsnp	rs13275988	A	G	ALLELIC	5/17	29/21	7.627	1	0.005751
8	dbsnp	rs10090960	G	A	ALLELIC	5/17	29/21	7.627	1	0.005751
9	dbsnp	rs10967657	G	A	ALLELIC	5/17	29/21	7.627	1	0.005751
9	dbsnp	rs10868854	A	G	ALLELIC	17/5	21/29	7.627	1	0.005751
9	dbsnp	rs10781013	A	G	ALLELIC	17/5	21/29	7.627	1	0.005751
9	dbsnp	rs6415829	A	G	ALLELIC	5/17	29/21	7.627	1	0.005751
10	dbsnp	rs1750726	A	G	ALLELIC	5/17	29/21	7.627	1	0.005751
10	dbsnp	rs1329173	G	A	ALLELIC	5/17	29/21	7.627	1	0.005751
11	dbsnp	rs10766807	A	G	ALLELIC	5/17	29/21	7.627	1	0.005751
11	dbsnp	rs7102381	G	A	ALLELIC	5/17	29/21	7.627	1	0.005751
11	dbsnp	rs4756265	A	G	ALLELIC	5/17	29/21	7.627	1	0.005751
11	dbsnp	rs1499511	G	A	ALLELIC	5/17	29/21	7.627	1	0.005751
11	dbsnp	rs1461688	A	G	ALLELIC	5/17	29/21	7.627	1	0.005751
12	dbsnp	rs4483672	G	A	ALLELIC	5/17	29/21	7.627	1	0.005751
12	dbsnp	rs4964604	A	G	ALLELIC	5/17	29/21	7.627	1	0.005751
13	dbsnp	rs9536354	A	G	ALLELIC	5/17	29/21	7.627	1	0.005751
14	dbsnp	rs1191555	G	A	ALLELIC	5/17	29/21	7.627	1	0.005751
14	dbsnp	rs7159758	A	C	ALLELIC	5/17	29/21	7.627	1	0.005751
14	dbsnp	rs8015328	A	G	ALLELIC	5/17	29/21	7.627	1	0.005751
14	dbsnp	rs7140309	A	G	ALLELIC	5/17	29/21	7.627	1	0.005751
15	dbsnp	rs11070236	A	C	ALLELIC	5/17	29/21	7.627	1	0.005751
15	dbsnp	rs17465692	G	A	ALLELIC	5/17	29/21	7.627	1	0.005751
17	dbsnp	rs3760410	A	G	ALLELIC	5/17	29/21	7.627	1	0.005751
20	dbsnp	rs2876233	G	A	ALLELIC	5/17	29/21	7.627	1	0.005751

20	dbsnp	rs2423610	G	A	ALLELIC	5/17	29/21	7.627	1	0.005751
20	dbsnp	rs458332	G	A	ALLELIC	5/17	29/21	7.627	1	0.005751
20	dbsnp	rs4142484	A	G	ALLELIC	5/17	29/21	7.627	1	0.005751
23	dbsnp	rs719499	A	G	ALLELIC	5/17	29/21	7.627	1	0.005751
23	dbsnp	rs5928136	A	G	ALLELIC	5/17	29/21	7.627	1	0.005751
23	dbsnp	rs5929690	A	G	ALLELIC	5/17	29/21	7.627	1	0.005751
2	dbsnp	rs3087873	A	G	ALLELIC	1/19	19/31	7.623	1	0.005763
2	dbsnp	rs1586627	G	A	ALLELIC	1/19	19/31	7.623	1	0.005763
2	dbsnp	rs10490692	G	A	ALLELIC	1/19	19/31	7.623	1	0.005763
3	dbsnp	rs1545771	G	A	ALLELIC	1/19	19/31	7.623	1	0.005763
4	dbsnp	rs10517227	G	A	ALLELIC	1/19	19/31	7.623	1	0.005763
6	dbsnp	rs1558205	A	C	ALLELIC	1/19	19/31	7.623	1	0.005763
9	dbsnp	rs373546	G	A	ALLELIC	1/19	19/31	7.623	1	0.005763
9	dbsnp	rs9410624	G	A	ALLELIC	1/19	19/31	7.623	1	0.005763
12	dbsnp	rs1381802	G	A	ALLELIC	1/19	19/31	7.623	1	0.005763
12	dbsnp	rs11116045	G	A	ALLELIC	1/19	19/31	7.623	1	0.005763
13	dbsnp	rs7330056	G	A	ALLELIC	1/19	19/31	7.623	1	0.005763
13	dbsnp	rs916593	A	G	ALLELIC	1/19	19/31	7.623	1	0.005763
13	dbsnp	rs2793700	A	C	ALLELIC	1/19	19/31	7.623	1	0.005763
14	dbsnp	rs914073	A	G	ALLELIC	1/19	19/31	7.623	1	0.005763
14	dbsnp	rs17097317	G	A	ALLELIC	1/19	19/31	7.623	1	0.005763
15	dbsnp	rs10519078	G	A	ALLELIC	1/19	19/31	7.623	1	0.005763
16	dbsnp	rs223812	G	A	ALLELIC	1/19	19/31	7.623	1	0.005763
18	dbsnp	rs11662309	G	A	ALLELIC	1/19	19/31	7.623	1	0.005763
23	dbsnp	rs11096210	G	A	ALLELIC	1/19	19/31	7.623	1	0.005763
1	dbsnp	rs7548900	A	C	ALLELIC	2/20	21/29	7.611	1	0.005802
2	dbsnp	rs3770809	A	C	ALLELIC	2/20	21/29	7.611	1	0.005802
2	dbsnp	rs2072535	C	A	ALLELIC	2/20	21/29	7.611	1	0.005802
2	dbsnp	rs780012	G	A	ALLELIC	2/20	21/29	7.611	1	0.005802
2	dbsnp	rs386549	A	G	ALLELIC	2/20	21/29	7.611	1	0.005802
2	dbsnp	rs281527	A	G	ALLELIC	2/20	21/29	7.611	1	0.005802
3	dbsnp	rs4685707	A	G	ALLELIC	2/20	21/29	7.611	1	0.005802
3	dbsnp	rs4688605	G	A	ALLELIC	2/20	21/29	7.611	1	0.005802
5	dbsnp	rs2561231	G	A	ALLELIC	2/20	21/29	7.611	1	0.005802
6	dbsnp	rs2531804	G	A	ALLELIC	2/20	21/29	7.611	1	0.005802
6	dbsnp	rs976471	G	A	ALLELIC	2/20	21/29	7.611	1	0.005802
6	dbsnp	rs9493627	A	G	ALLELIC	2/20	21/29	7.611	1	0.005802
6	dbsnp	rs1022514	A	G	ALLELIC	2/20	21/29	7.611	1	0.005802
6	dbsnp	rs4896774	G	A	ALLELIC	2/20	21/29	7.611	1	0.005802
7	dbsnp	rs6951952	A	G	ALLELIC	2/20	21/29	7.611	1	0.005802
7	dbsnp	rs4717839	G	A	ALLELIC	2/20	21/29	7.611	1	0.005802
7	dbsnp	rs7785642	A	C	ALLELIC	2/20	21/29	7.611	1	0.005802
8	dbsnp	rs13254494	G	A	ALLELIC	2/20	21/29	7.611	1	0.005802
8	dbsnp	rs7818607	A	C	ALLELIC	2/20	21/29	7.611	1	0.005802
8	dbsnp	rs6998848	A	G	ALLELIC	2/20	21/29	7.611	1	0.005802
9	dbsnp	rs12380325	G	A	ALLELIC	2/20	21/29	7.611	1	0.005802
10	dbsnp	rs3812656	G	A	ALLELIC	2/20	21/29	7.611	1	0.005802
10	dbsnp	rs2579174	G	A	ALLELIC	2/20	21/29	7.611	1	0.005802

10	dbsnp	rs1248635	A	G	ALLELIC	2/20	21/29	7.611	1	0.005802
11	dbsnp	rs4758332	G	A	ALLELIC	2/20	21/29	7.611	1	0.005802
12	dbsnp	rs7301431	A	G	ALLELIC	2/20	21/29	7.611	1	0.005802
13	dbsnp	rs12869772	G	A	ALLELIC	2/20	21/29	7.611	1	0.005802
16	dbsnp	rs11641981	A	G	ALLELIC	2/20	21/29	7.611	1	0.005802
17	dbsnp	rs8070953	A	G	ALLELIC	2/20	21/29	7.611	1	0.005802
20	dbsnp	rs6043626	A	G	ALLELIC	2/20	21/29	7.611	1	0.005802
21	dbsnp	rs2837926	A	C	ALLELIC	2/20	21/29	7.611	1	0.005802
23	dbsnp	rs6633624	G	A	ALLELIC	2/20	21/29	7.611	1	0.005802
23	dbsnp	rs12556777	A	C	ALLELIC	2/20	21/29	7.611	1	0.005802
1	dbsnp	rs654072	A	G	ALLELIC	7/11	5/45	7.601	1	0.005835
5	dbsnp	rs10070707	G	A	ALLELIC	3/19	23/25	7.593	1	0.005859
9	dbsnp	rs4742004	G	A	ALLELIC	3/19	23/25	7.593	1	0.005859
7	dbsnp	rs11561890	A	G	ALLELIC	12/4	17/31	7.588	1	0.005877
13	dbsnp	rs9576177	G	A	ALLELIC	4/14	30/20	7.556	1	0.005983
8	dbsnp	rs1825011	A	G	ALLELIC	3/17	0/48	7.532	1	0.00606
16	dbsnp	rs12921986	G	A	ALLELIC	3/17	0/48	7.532	1	0.00606
22	dbsnp	rs13055216	G	A	ALLELIC	3/17	0/48	7.532	1	0.00606
10	dbsnp	rs2588948	A	G	ALLELIC	1/21	17/31	7.527	1	0.00608
19	dbsnp	rs9967619	A	C	ALLELIC	1/21	17/31	7.527	1	0.00608
22	dbsnp	rs5748449	A	G	ALLELIC	1/21	17/31	7.527	1	0.00608
1	dbsnp	rs997985	A	G	ALLELIC	14/6	17/33	7.504	1	0.006157
1	dbsnp	rs609384	A	G	ALLELIC	14/6	17/33	7.504	1	0.006157
2	dbsnp	rs930767	C	A	ALLELIC	14/6	17/33	7.504	1	0.006157
2	dbsnp	rs12472756	G	A	ALLELIC	14/6	17/33	7.504	1	0.006157
2	dbsnp	rs6542747	A	G	ALLELIC	14/6	17/33	7.504	1	0.006157
2	dbsnp	rs10200294	G	A	ALLELIC	14/6	17/33	7.504	1	0.006157
3	dbsnp	rs399821	A	G	ALLELIC	14/6	17/33	7.504	1	0.006157
3	dbsnp	rs2272486	A	G	ALLELIC	14/6	17/33	7.504	1	0.006157
5	dbsnp	rs11134251	A	C	ALLELIC	14/6	17/33	7.504	1	0.006157
5	dbsnp	rs1382302	A	G	ALLELIC	14/6	17/33	7.504	1	0.006157
6	dbsnp	rs1538340	A	G	ALLELIC	14/6	17/33	7.504	1	0.006157
7	dbsnp	rs4623321	A	G	ALLELIC	14/6	17/33	7.504	1	0.006157
8	dbsnp	rs1027266	G	A	ALLELIC	14/6	17/33	7.504	1	0.006157
11	dbsnp	rs12274692	G	A	ALLELIC	14/6	17/33	7.504	1	0.006157
12	dbsnp	rs7314842	A	G	ALLELIC	14/6	17/33	7.504	1	0.006157
13	dbsnp	rs7328598	A	C	ALLELIC	14/6	17/33	7.504	1	0.006157
15	dbsnp	rs8030562	G	A	ALLELIC	14/6	17/33	7.504	1	0.006157
15	dbsnp	rs8027781	C	A	ALLELIC	14/6	17/33	7.504	1	0.006157
18	dbsnp	rs8090956	G	A	ALLELIC	14/6	17/33	7.504	1	0.006157
18	dbsnp	rs1705465	A	C	ALLELIC	14/6	17/33	7.504	1	0.006157
18	dbsnp	rs4344834	A	G	ALLELIC	14/6	17/33	7.504	1	0.006157
19	dbsnp	rs10423902	G	A	ALLELIC	14/6	17/33	7.504	1	0.006157
23	dbsnp	rs4338301	A	G	ALLELIC	14/6	17/33	7.504	1	0.006157
23	dbsnp	rs1997737	G	A	ALLELIC	14/6	17/33	7.504	1	0.006157
23	dbsnp	rs7061149	G	A	ALLELIC	14/6	17/33	7.504	1	0.006157
23	dbsnp	rs12557898	G	A	ALLELIC	6/16	30/18	7.494	1	0.006189
12	dbsnp	rs11050396	C	A	ALLELIC	3/19	22/24	7.483	1	0.006229

18	dbsnp	rs28455052	A	G	ALLELIC	10/10	8/38	7.473	1	0.006264
1	dbsnp	rs7552428	G	A	ALLELIC	4/16	28/22	7.461	1	0.006307
2	dbsnp	rs2067564	G	A	ALLELIC	4/16	28/22	7.461	1	0.006307
4	dbsnp	rs2237051	A	G	ALLELIC	4/16	28/22	7.461	1	0.006307
5	dbsnp	rs2591933	A	G	ALLELIC	4/16	28/22	7.461	1	0.006307
5	dbsnp	rs2913496	A	C	ALLELIC	4/16	28/22	7.461	1	0.006307
6	dbsnp	rs9267845	T	A	ALLELIC	4/16	28/22	7.461	1	0.006307
8	dbsnp	rs2617086	A	C	ALLELIC	4/16	28/22	7.461	1	0.006307
8	dbsnp	rs7831711	A	C	ALLELIC	4/16	28/22	7.461	1	0.006307
12	dbsnp	rs2371455	A	C	ALLELIC	4/16	28/22	7.461	1	0.006307
12	dbsnp	rs11116423	G	A	ALLELIC	4/16	28/22	7.461	1	0.006307
12	dbsnp	rs2926474	A	G	ALLELIC	4/16	28/22	7.461	1	0.006307
13	dbsnp	rs9511097	A	G	ALLELIC	4/16	28/22	7.461	1	0.006307
13	dbsnp	rs990780	G	A	ALLELIC	4/16	28/22	7.461	1	0.006307
19	dbsnp	rs2431835	A	G	ALLELIC	4/16	28/22	7.461	1	0.006307
20	dbsnp	rs873544	G	A	ALLELIC	4/16	28/22	7.461	1	0.006307
1	dbsnp	rs4531246	A	C	ALLELIC	12/10	11/39	7.444	1	0.006366
1	dbsnp	rs2494641	A	G	ALLELIC	12/10	11/39	7.444	1	0.006366
1	dbsnp	rs6686674	A	G	ALLELIC	12/10	11/39	7.444	1	0.006366
1	dbsnp	rs6676838	G	A	ALLELIC	12/10	11/39	7.444	1	0.006366
1	dbsnp	rs4539125	G	A	ALLELIC	12/10	11/39	7.444	1	0.006366
1	dbsnp	rs12127281	A	G	ALLELIC	12/10	11/39	7.444	1	0.006366
1	dbsnp	rs7551606	A	G	ALLELIC	12/10	11/39	7.444	1	0.006366
2	dbsnp	rs9287739	A	G	ALLELIC	12/10	11/39	7.444	1	0.006366
2	dbsnp	rs10202489	G	A	ALLELIC	12/10	11/39	7.444	1	0.006366
2	dbsnp	rs1438307	C	A	ALLELIC	12/10	11/39	7.444	1	0.006366
2	dbsnp	rs1990760	G	A	ALLELIC	12/10	11/39	7.444	1	0.006366
2	dbsnp	rs4853646	A	C	ALLELIC	12/10	11/39	7.444	1	0.006366
2	dbsnp	rs4853978	A	G	ALLELIC	12/10	11/39	7.444	1	0.006366
2	dbsnp	rs4149558	G	A	ALLELIC	12/10	11/39	7.444	1	0.006366
3	dbsnp	rs1344815	A	G	ALLELIC	12/10	11/39	7.444	1	0.006366
3	dbsnp	rs2339934	G	A	ALLELIC	12/10	11/39	7.444	1	0.006366
4	dbsnp	rs17509725	A	G	ALLELIC	12/10	11/39	7.444	1	0.006366
4	dbsnp	rs6820523	A	G	ALLELIC	12/10	11/39	7.444	1	0.006366
4	dbsnp	rs11132117	G	A	ALLELIC	12/10	11/39	7.444	1	0.006366
5	dbsnp	rs275502	A	G	ALLELIC	12/10	11/39	7.444	1	0.006366
5	dbsnp	rs3909431	C	A	ALLELIC	12/10	11/39	7.444	1	0.006366
5	dbsnp	rs17342682	A	G	ALLELIC	12/10	11/39	7.444	1	0.006366
5	dbsnp	rs888817	G	A	ALLELIC	12/10	11/39	7.444	1	0.006366
5	dbsnp	rs2544702	A	C	ALLELIC	12/10	11/39	7.444	1	0.006366
6	dbsnp	rs843319	G	A	ALLELIC	12/10	11/39	7.444	1	0.006366
6	dbsnp	rs506398	G	A	ALLELIC	12/10	11/39	7.444	1	0.006366
6	dbsnp	rs2497115	A	C	ALLELIC	12/10	11/39	7.444	1	0.006366
6	dbsnp	rs1338715	A	C	ALLELIC	12/10	11/39	7.444	1	0.006366
6	dbsnp	rs2024971	G	A	ALLELIC	12/10	11/39	7.444	1	0.006366
6	dbsnp	rs10499250	A	G	ALLELIC	12/10	11/39	7.444	1	0.006366
7	dbsnp	rs849742	A	G	ALLELIC	12/10	11/39	7.444	1	0.006366
7	dbsnp	rs39416	A	G	ALLELIC	12/10	11/39	7.444	1	0.006366

7	dbsnp	rs318572	G	A	ALLELIC	12/10	11/39	7.444	1	0.006366
7	dbsnp	rs7794818	G	A	ALLELIC	12/10	11/39	7.444	1	0.006366
7	dbsnp	rs9639924	A	C	ALLELIC	12/10	11/39	7.444	1	0.006366
7	dbsnp	rs992805	A	G	ALLELIC	12/10	11/39	7.444	1	0.006366
7	dbsnp	rs2241859	A	C	ALLELIC	12/10	11/39	7.444	1	0.006366
7	dbsnp	rs1631327	A	G	ALLELIC	12/10	11/39	7.444	1	0.006366
7	dbsnp	rs1730209	A	G	ALLELIC	12/10	11/39	7.444	1	0.006366
8	dbsnp	rs4921697	A	G	ALLELIC	12/10	11/39	7.444	1	0.006366
8	dbsnp	rs7846693	A	G	ALLELIC	12/10	11/39	7.444	1	0.006366
8	dbsnp	rs1357818	A	G	ALLELIC	12/10	11/39	7.444	1	0.006366
8	dbsnp	rs642074	C	A	ALLELIC	12/10	11/39	7.444	1	0.006366
9	dbsnp	rs2111554	G	A	ALLELIC	12/10	11/39	7.444	1	0.006366
9	dbsnp	rs7865138	C	A	ALLELIC	12/10	11/39	7.444	1	0.006366
10	dbsnp	rs4881551	A	G	ALLELIC	12/10	11/39	7.444	1	0.006366
10	dbsnp	rs4750093	A	G	ALLELIC	12/10	11/39	7.444	1	0.006366
10	dbsnp	rs1810205	G	A	ALLELIC	12/10	11/39	7.444	1	0.006366
10	dbsnp	rs10825072	G	A	ALLELIC	12/10	11/39	7.444	1	0.006366
10	dbsnp	rs1046778	G	A	ALLELIC	12/10	11/39	7.444	1	0.006366
10	dbsnp	rs7897654	G	A	ALLELIC	12/10	11/39	7.444	1	0.006366
10	dbsnp	rs3802701	A	G	ALLELIC	12/10	11/39	7.444	1	0.006366
10	dbsnp	rs7097254	A	C	ALLELIC	12/10	11/39	7.444	1	0.006366
10	dbsnp	rs10829862	G	A	ALLELIC	12/10	11/39	7.444	1	0.006366
11	dbsnp	rs1792565	C	A	ALLELIC	12/10	11/39	7.444	1	0.006366
11	dbsnp	rs7109209	A	G	ALLELIC	12/10	11/39	7.444	1	0.006366
11	dbsnp	rs2729881	C	A	ALLELIC	12/10	11/39	7.444	1	0.006366
11	dbsnp	rs7924766	G	A	ALLELIC	12/10	11/39	7.444	1	0.006366
11	dbsnp	rs4922828	G	A	ALLELIC	12/10	11/39	7.444	1	0.006366
11	dbsnp	rs6484002	A	G	ALLELIC	12/10	11/39	7.444	1	0.006366
11	dbsnp	rs1949541	G	A	ALLELIC	12/10	11/39	7.444	1	0.006366
11	dbsnp	rs12274346	G	A	ALLELIC	12/10	11/39	7.444	1	0.006366
11	dbsnp	rs11236839	A	G	ALLELIC	12/10	11/39	7.444	1	0.006366
11	dbsnp	rs518181	C	A	ALLELIC	12/10	11/39	7.444	1	0.006366
13	dbsnp	rs880624	G	A	ALLELIC	12/10	11/39	7.444	1	0.006366
13	dbsnp	rs1323951	G	A	ALLELIC	12/10	11/39	7.444	1	0.006366
13	dbsnp	rs9564716	G	A	ALLELIC	12/10	11/39	7.444	1	0.006366
13	dbsnp	rs9518764	G	A	ALLELIC	12/10	11/39	7.444	1	0.006366
13	dbsnp	rs622911	G	A	ALLELIC	12/10	11/39	7.444	1	0.006366
14	dbsnp	rs227395	A	G	ALLELIC	12/10	11/39	7.444	1	0.006366
14	dbsnp	rs4577003	G	A	ALLELIC	12/10	11/39	7.444	1	0.006366
14	dbsnp	rs10150134	G	A	ALLELIC	12/10	11/39	7.444	1	0.006366
14	dbsnp	rs7151475	G	A	ALLELIC	12/10	11/39	7.444	1	0.006366
15	dbsnp	rs515341	A	G	ALLELIC	12/10	11/39	7.444	1	0.006366
15	dbsnp	rs4778861	G	A	ALLELIC	12/10	11/39	7.444	1	0.006366
16	dbsnp	rs6498114	C	A	ALLELIC	12/10	11/39	7.444	1	0.006366
16	dbsnp	rs3087456	G	A	ALLELIC	12/10	11/39	7.444	1	0.006366
17	dbsnp	rs8069926	A	G	ALLELIC	12/10	11/39	7.444	1	0.006366
18	dbsnp	rs299243	G	A	ALLELIC	12/10	11/39	7.444	1	0.006366
18	dbsnp	rs1521920	A	C	ALLELIC	12/10	11/39	7.444	1	0.006366

20	dbsnp	rs6021474	G	A	ALLELIC	12/10	11/39	7.444	1	0.006366
20	dbsnp	rs2268689	G	A	ALLELIC	12/10	11/39	7.444	1	0.006366
20	dbsnp	rs6027132	G	A	ALLELIC	12/10	11/39	7.444	1	0.006366
22	dbsnp	rs3171692	A	G	ALLELIC	12/10	11/39	7.444	1	0.006366
22	dbsnp	rs2024566	G	A	ALLELIC	12/10	11/39	7.444	1	0.006366
22	dbsnp	rs5770651	G	A	ALLELIC	12/10	11/39	7.444	1	0.006366
23	dbsnp	rs2188454	G	A	ALLELIC	12/10	11/39	7.444	1	0.006366
23	dbsnp	rs2311430	G	A	ALLELIC	12/10	11/39	7.444	1	0.006366
23	dbsnp	rs933383	A	G	ALLELIC	12/10	11/39	7.444	1	0.006366
23	dbsnp	rs6571284	G	A	ALLELIC	12/10	11/39	7.444	1	0.006366
3	dbsnp	rs212050	A	C	ALLELIC	3/15	18/14	7.41	1	0.006487
18	dbsnp	rs7236165	G	A	ALLELIC	1/19	18/30	7.406	1	0.0065
1	dbsnp	rs900895	A	G	ALLELIC	7/13	4/44	7.404	1	0.006509
9	dbsnp	rs493126	A	G	ALLELIC	7/13	4/44	7.404	1	0.006509
1	dbsnp	rs1890449	A	C	ALLELIC	10/10	9/41	7.397	1	0.006532
1	dbsnp	rs3890756	G	A	ALLELIC	10/10	9/41	7.397	1	0.006532
1	dbsnp	rs10903106	G	A	ALLELIC	10/10	9/41	7.397	1	0.006532
1	dbsnp	rs12439	G	A	ALLELIC	10/10	9/41	7.397	1	0.006532
1	dbsnp	rs11584669	C	A	ALLELIC	10/10	9/41	7.397	1	0.006532
2	dbsnp	rs4670135	A	G	ALLELIC	10/10	9/41	7.397	1	0.006532
2	dbsnp	rs12692010	A	G	ALLELIC	10/10	9/41	7.397	1	0.006532
2	dbsnp	rs13405357	G	A	ALLELIC	10/10	9/41	7.397	1	0.006532
2	dbsnp	rs2943645	G	A	ALLELIC	10/10	9/41	7.397	1	0.006532
3	dbsnp	rs6793635	A	G	ALLELIC	10/10	9/41	7.397	1	0.006532
4	dbsnp	rs7690552	C	A	ALLELIC	10/10	9/41	7.397	1	0.006532
4	dbsnp	rs11732672	G	A	ALLELIC	10/10	9/41	7.397	1	0.006532
4	dbsnp	rs4859456	A	G	ALLELIC	10/10	9/41	7.397	1	0.006532
5	dbsnp	rs6869920	G	A	ALLELIC	10/10	9/41	7.397	1	0.006532
5	dbsnp	rs2059217	G	A	ALLELIC	10/10	9/41	7.397	1	0.006532
5	dbsnp	rs11745118	G	A	ALLELIC	10/10	9/41	7.397	1	0.006532
6	dbsnp	rs2627239	C	A	ALLELIC	10/10	9/41	7.397	1	0.006532
7	dbsnp	rs2195894	A	C	ALLELIC	10/10	9/41	7.397	1	0.006532
7	dbsnp	rs6959554	G	A	ALLELIC	10/10	9/41	7.397	1	0.006532
7	dbsnp	rs12672674	C	A	ALLELIC	10/10	9/41	7.397	1	0.006532
9	dbsnp	rs7026407	C	A	ALLELIC	10/10	9/41	7.397	1	0.006532
9	dbsnp	rs621277	G	A	ALLELIC	10/10	9/41	7.397	1	0.006532
10	dbsnp	rs12766994	G	A	ALLELIC	10/10	9/41	7.397	1	0.006532
10	dbsnp	rs16919426	C	A	ALLELIC	10/10	9/41	7.397	1	0.006532
10	dbsnp	rs9417245	A	G	ALLELIC	10/10	9/41	7.397	1	0.006532
11	dbsnp	rs3858526	C	A	ALLELIC	10/10	9/41	7.397	1	0.006532
12	dbsnp	rs2448063	A	C	ALLELIC	10/10	9/41	7.397	1	0.006532
12	dbsnp	rs7958404	A	G	ALLELIC	10/10	9/41	7.397	1	0.006532
13	dbsnp	rs17553284	A	G	ALLELIC	10/10	9/41	7.397	1	0.006532
13	dbsnp	rs11617079	G	A	ALLELIC	10/10	9/41	7.397	1	0.006532
14	dbsnp	rs1951244	G	A	ALLELIC	10/10	9/41	7.397	1	0.006532
15	dbsnp	rs12900387	A	G	ALLELIC	10/10	9/41	7.397	1	0.006532
16	dbsnp	rs6500099	A	G	ALLELIC	10/10	9/41	7.397	1	0.006532
18	dbsnp	rs8091251	G	A	ALLELIC	10/10	9/41	7.397	1	0.006532

19	dbsnp	rs400058	A	G	ALLELIC	10/10	9/41	7.397	1	0.006532
19	dbsnp	rs8110582	A	G	ALLELIC	10/10	9/41	7.397	1	0.006532
19	dbsnp	rs1036238	A	C	ALLELIC	10/10	9/41	7.397	1	0.006532
22	dbsnp	rs10470277	A	G	ALLELIC	10/10	9/41	7.397	1	0.006532
1	dbsnp	rs2474460	A	G	ALLELIC	6/16	31/19	7.376	1	0.006612
1	dbsnp	rs6577393	G	A	ALLELIC	16/6	19/31	7.376	1	0.006612
1	dbsnp	rs6674628	G	A	ALLELIC	16/6	19/31	7.376	1	0.006612
1	dbsnp	rs2095726	A	G	ALLELIC	16/6	19/31	7.376	1	0.006612
1	dbsnp	rs4620530	A	C	ALLELIC	6/16	31/19	7.376	1	0.006612
1	dbsnp	rs2184185	G	A	ALLELIC	16/6	19/31	7.376	1	0.006612
2	dbsnp	rs16864123	G	A	ALLELIC	16/6	19/31	7.376	1	0.006612
2	dbsnp	rs7581689	A	G	ALLELIC	16/6	19/31	7.376	1	0.006612
2	dbsnp	rs6547479	C	A	ALLELIC	16/6	19/31	7.376	1	0.006612
2	dbsnp	rs10184962	C	A	ALLELIC	6/16	31/19	7.376	1	0.006612
2	dbsnp	rs12327971	G	A	ALLELIC	6/16	31/19	7.376	1	0.006612
2	dbsnp	rs12691759	A	G	ALLELIC	6/16	31/19	7.376	1	0.006612
2	dbsnp	rs6730381	G	A	ALLELIC	6/16	31/19	7.376	1	0.006612
2	dbsnp	rs10084181	C	A	ALLELIC	6/16	31/19	7.376	1	0.006612
2	dbsnp	rs7599348	G	A	ALLELIC	16/6	19/31	7.376	1	0.006612
2	dbsnp	rs10195178	A	C	ALLELIC	6/16	31/19	7.376	1	0.006612
3	dbsnp	rs9875041	C	A	ALLELIC	16/6	19/31	7.376	1	0.006612
4	dbsnp	rs3775031	A	G	ALLELIC	16/6	19/31	7.376	1	0.006612
4	dbsnp	rs12500473	A	G	ALLELIC	16/6	19/31	7.376	1	0.006612
4	dbsnp	rs6833223	G	A	ALLELIC	16/6	19/31	7.376	1	0.006612
4	dbsnp	rs3113687	G	A	ALLELIC	16/6	19/31	7.376	1	0.006612
4	dbsnp	rs4597781	A	G	ALLELIC	16/6	19/31	7.376	1	0.006612
4	dbsnp	rs4862122	A	G	ALLELIC	16/6	19/31	7.376	1	0.006612
5	dbsnp	rs269853	G	A	ALLELIC	16/6	19/31	7.376	1	0.006612
5	dbsnp	rs12658302	A	G	ALLELIC	16/6	19/31	7.376	1	0.006612
6	dbsnp	rs9462209	C	A	ALLELIC	6/16	31/19	7.376	1	0.006612
6	dbsnp	rs6905394	A	G	ALLELIC	6/16	31/19	7.376	1	0.006612
6	dbsnp	rs910532	A	G	ALLELIC	16/6	19/31	7.376	1	0.006612
6	dbsnp	rs533183	G	A	ALLELIC	6/16	31/19	7.376	1	0.006612
6	dbsnp	rs6570821	A	G	ALLELIC	16/6	19/31	7.376	1	0.006612
7	dbsnp	rs799234	A	G	ALLELIC	16/6	19/31	7.376	1	0.006612
7	dbsnp	rs4723537	G	A	ALLELIC	16/6	19/31	7.376	1	0.006612
7	dbsnp	rs2286117	G	A	ALLELIC	6/16	31/19	7.376	1	0.006612
7	dbsnp	rs2032582	C	A	ALLELIC	16/6	19/31	7.376	1	0.006612
7	dbsnp	rs11761128	G	A	ALLELIC	16/6	19/31	7.376	1	0.006612
7	dbsnp	rs7779135	G	A	ALLELIC	16/6	19/31	7.376	1	0.006612
8	dbsnp	rs3752670	A	G	ALLELIC	16/6	19/31	7.376	1	0.006612
8	dbsnp	rs2700714	A	G	ALLELIC	16/6	19/31	7.376	1	0.006612
8	dbsnp	rs2979823	G	A	ALLELIC	16/6	19/31	7.376	1	0.006612
8	dbsnp	rs3110150	G	A	ALLELIC	16/6	19/31	7.376	1	0.006612
9	dbsnp	rs277742	C	A	ALLELIC	16/6	19/31	7.376	1	0.006612
9	dbsnp	rs277763	A	G	ALLELIC	16/6	19/31	7.376	1	0.006612
10	dbsnp	rs2384148	A	G	ALLELIC	16/6	19/31	7.376	1	0.006612
10	dbsnp	rs7902653	G	A	ALLELIC	16/6	19/31	7.376	1	0.006612

10	dbsnp	rs4403749	G	A	ALLELIC	16/6	19/31	7.376	1	0.006612
12	dbsnp	rs2088809	A	G	ALLELIC	16/6	19/31	7.376	1	0.006612
12	dbsnp	rs7968902	A	C	ALLELIC	16/6	19/31	7.376	1	0.006612
12	dbsnp	rs10847968	C	A	ALLELIC	16/6	19/31	7.376	1	0.006612
13	dbsnp	rs176059	G	A	ALLELIC	16/6	19/31	7.376	1	0.006612
13	dbsnp	rs11069237	A	G	ALLELIC	16/6	19/31	7.376	1	0.006612
13	dbsnp	rs9556582	G	A	ALLELIC	16/6	19/31	7.376	1	0.006612
13	dbsnp	rs874199	G	A	ALLELIC	16/6	19/31	7.376	1	0.006612
14	dbsnp	rs7141573	G	A	ALLELIC	16/6	19/31	7.376	1	0.006612
15	dbsnp	rs6494623	G	A	ALLELIC	6/16	31/19	7.376	1	0.006612
16	dbsnp	rs4785367	A	G	ALLELIC	16/6	19/31	7.376	1	0.006612
16	dbsnp	rs7197843	G	A	ALLELIC	16/6	19/31	7.376	1	0.006612
17	dbsnp	rs1005321	G	A	ALLELIC	16/6	19/31	7.376	1	0.006612
17	dbsnp	rs9904092	A	C	ALLELIC	6/16	31/19	7.376	1	0.006612
17	dbsnp	rs2958875	A	G	ALLELIC	16/6	19/31	7.376	1	0.006612
17	dbsnp	rs2159041	A	G	ALLELIC	16/6	19/31	7.376	1	0.006612
17	dbsnp	rs9911758	G	A	ALLELIC	16/6	19/31	7.376	1	0.006612
18	dbsnp	rs8085678	C	A	ALLELIC	16/6	19/31	7.376	1	0.006612
18	dbsnp	rs10502849	A	G	ALLELIC	16/6	19/31	7.376	1	0.006612
20	dbsnp	rs765736	A	G	ALLELIC	16/6	19/31	7.376	1	0.006612
20	dbsnp	rs11204467	C	A	ALLELIC	16/6	19/31	7.376	1	0.006612
20	dbsnp	rs6089695	A	G	ALLELIC	16/6	19/31	7.376	1	0.006612
21	dbsnp	rs2223743	G	A	ALLELIC	16/6	19/31	7.376	1	0.006612
23	dbsnp	rs1795595	A	G	ALLELIC	16/6	19/31	7.376	1	0.006612
23	dbsnp	rs5942915	G	A	ALLELIC	16/6	19/31	7.376	1	0.006612
2	dbsnp	rs17522496	A	G	ALLELIC	6/14	3/47	7.344	1	0.006728
2	dbsnp	rs13428321	A	C	ALLELIC	6/14	3/47	7.344	1	0.006728
2	dbsnp	rs4663990	G	A	ALLELIC	6/14	3/47	7.344	1	0.006728
3	dbsnp	rs1447659	A	C	ALLELIC	6/14	3/47	7.344	1	0.006728
4	dbsnp	rs986240	C	A	ALLELIC	6/14	3/47	7.344	1	0.006728
4	dbsnp	rs2903701	A	G	ALLELIC	6/14	3/47	7.344	1	0.006728
4	dbsnp	rs13135838	A	G	ALLELIC	6/14	3/47	7.344	1	0.006728
5	dbsnp	rs6450480	G	A	ALLELIC	6/14	3/47	7.344	1	0.006728
5	dbsnp	rs583195	A	G	ALLELIC	6/14	3/47	7.344	1	0.006728
5	dbsnp	rs2178270	G	A	ALLELIC	6/14	3/47	7.344	1	0.006728
6	dbsnp	rs10946483	G	A	ALLELIC	6/14	3/47	7.344	1	0.006728
6	dbsnp	rs29227	A	C	ALLELIC	6/14	3/47	7.344	1	0.006728
6	dbsnp	rs11759652	A	G	ALLELIC	6/14	3/47	7.344	1	0.006728
6	dbsnp	rs11155418	A	G	ALLELIC	6/14	3/47	7.344	1	0.006728
6	dbsnp	rs12197582	A	G	ALLELIC	6/14	3/47	7.344	1	0.006728
8	dbsnp	rs4872380	A	G	ALLELIC	6/14	3/47	7.344	1	0.006728
8	dbsnp	rs11781930	A	G	ALLELIC	6/14	3/47	7.344	1	0.006728
8	dbsnp	rs1452796	A	G	ALLELIC	6/14	3/47	7.344	1	0.006728
8	dbsnp	rs17430533	A	C	ALLELIC	6/14	3/47	7.344	1	0.006728
8	dbsnp	rs11166618	A	G	ALLELIC	6/14	3/47	7.344	1	0.006728
9	dbsnp	rs12378054	G	A	ALLELIC	6/14	3/47	7.344	1	0.006728
9	dbsnp	rs1576836	G	A	ALLELIC	6/14	3/47	7.344	1	0.006728
11	dbsnp	rs2860519	G	A	ALLELIC	6/14	3/47	7.344	1	0.006728

12	dbsnp	rs12307532	C	A	ALLELIC	6/14	3/47	7.344	1	0.006728
12	dbsnp	rs17116524	G	A	ALLELIC	6/14	3/47	7.344	1	0.006728
12	dbsnp	rs16944947	A	G	ALLELIC	6/14	3/47	7.344	1	0.006728
12	dbsnp	rs16944966	C	A	ALLELIC	6/14	3/47	7.344	1	0.006728
12	dbsnp	rs7313297	A	G	ALLELIC	6/14	3/47	7.344	1	0.006728
12	dbsnp	rs7299358	G	A	ALLELIC	6/14	3/47	7.344	1	0.006728
12	dbsnp	rs12321965	A	G	ALLELIC	6/14	3/47	7.344	1	0.006728
13	dbsnp	rs7320437	C	A	ALLELIC	6/14	3/47	7.344	1	0.006728
13	dbsnp	rs17074792	A	G	ALLELIC	6/14	3/47	7.344	1	0.006728
14	dbsnp	rs4982322	A	C	ALLELIC	6/14	3/47	7.344	1	0.006728
14	dbsnp	rs10138951	G	A	ALLELIC	6/14	3/47	7.344	1	0.006728
14	dbsnp	rs10132162	G	A	ALLELIC	6/14	3/47	7.344	1	0.006728
14	dbsnp	rs8005433	A	G	ALLELIC	6/14	3/47	7.344	1	0.006728
15	dbsnp	rs7163599	G	A	ALLELIC	6/14	3/47	7.344	1	0.006728
16	dbsnp	rs17772954	A	G	ALLELIC	6/14	3/47	7.344	1	0.006728
17	dbsnp	rs8082037	A	G	ALLELIC	6/14	3/47	7.344	1	0.006728
17	dbsnp	rs9914211	C	A	ALLELIC	6/14	3/47	7.344	1	0.006728
18	dbsnp	rs4798731	A	G	ALLELIC	6/14	3/47	7.344	1	0.006728
20	dbsnp	rs6133513	A	G	ALLELIC	6/14	3/47	7.344	1	0.006728
20	dbsnp	rs3746482	G	A	ALLELIC	6/14	3/47	7.344	1	0.006728
20	dbsnp	rs11696180	G	A	ALLELIC	6/14	3/47	7.344	1	0.006728
20	dbsnp	rs7268399	G	A	ALLELIC	6/14	3/47	7.344	1	0.006728
20	dbsnp	rs747063	A	G	ALLELIC	6/14	3/47	7.344	1	0.006728
20	dbsnp	rs6021236	C	A	ALLELIC	6/14	3/47	7.344	1	0.006728
20	dbsnp	rs6013509	A	G	ALLELIC	6/14	3/47	7.344	1	0.006728
23	dbsnp	rs7876886	C	A	ALLELIC	6/14	3/47	7.344	1	0.006728
23	dbsnp	rs36047913	A	G	ALLELIC	6/14	3/47	7.344	1	0.006728
5	dbsnp	rs28096	A	G	ALLELIC	2/16	21/23	7.34	1	0.006745
1	dbsnp	rs12724252	G	A	ALLELIC	2/18	22/28	7.33	1	0.006782
1	dbsnp	rs1933631	C	A	ALLELIC	2/18	22/28	7.33	1	0.006782
1	dbsnp	rs12754490	G	A	ALLELIC	2/18	22/28	7.33	1	0.006782
2	dbsnp	rs14291	G	A	ALLELIC	2/18	22/28	7.33	1	0.006782
5	dbsnp	rs396769	G	A	ALLELIC	2/18	22/28	7.33	1	0.006782
5	dbsnp	rs279403	G	A	ALLELIC	2/18	22/28	7.33	1	0.006782
6	dbsnp	rs6938042	C	A	ALLELIC	2/18	22/28	7.33	1	0.006782
6	dbsnp	rs1114644	A	G	ALLELIC	2/18	22/28	7.33	1	0.006782
6	dbsnp	rs7765680	A	G	ALLELIC	2/18	22/28	7.33	1	0.006782
8	dbsnp	rs6558462	C	A	ALLELIC	2/18	22/28	7.33	1	0.006782
8	dbsnp	rs8534	A	G	ALLELIC	2/18	22/28	7.33	1	0.006782
8	dbsnp	rs11998308	A	G	ALLELIC	2/18	22/28	7.33	1	0.006782
8	dbsnp	rs10102945	A	G	ALLELIC	2/18	22/28	7.33	1	0.006782
10	dbsnp	rs10884989	A	G	ALLELIC	2/18	22/28	7.33	1	0.006782
10	dbsnp	rs7083245	G	A	ALLELIC	2/18	22/28	7.33	1	0.006782
10	dbsnp	rs10736109	G	A	ALLELIC	2/18	22/28	7.33	1	0.006782
11	dbsnp	rs674946	A	G	ALLELIC	2/18	22/28	7.33	1	0.006782
13	dbsnp	rs9285110	G	A	ALLELIC	2/18	22/28	7.33	1	0.006782
13	dbsnp	rs2873372	G	A	ALLELIC	2/18	22/28	7.33	1	0.006782
16	dbsnp	rs729533	C	A	ALLELIC	2/18	22/28	7.33	1	0.006782

16	dbsnp	rs2965831	A	C	ALLELIC	2/18	22/28	7.33	1	0.006782
16	dbsnp	rs4782152	G	A	ALLELIC	2/18	22/28	7.33	1	0.006782
18	dbsnp	rs2874688	C	A	ALLELIC	2/18	22/28	7.33	1	0.006782
22	dbsnp	rs10854598	A	G	ALLELIC	2/18	22/28	7.33	1	0.006782
12	dbsnp	rs12822740	G	A	ALLELIC	12/10	10/36	7.319	1	0.006825
23	dbsnp	rs6603535	G	A	ALLELIC	0/22	13/35	7.317	1	0.00683
1	dbsnp	rs3795299	C	G	ALLELIC	13/7	15/35	7.292	1	0.006928
2	dbsnp	rs6743753	C	A	ALLELIC	13/7	15/35	7.292	1	0.006928
2	dbsnp	rs11694460	A	G	ALLELIC	3/17	25/25	7.292	1	0.006928
2	dbsnp	rs10171686	G	A	ALLELIC	3/17	25/25	7.292	1	0.006928
2	dbsnp	rs10194641	G	A	ALLELIC	13/7	15/35	7.292	1	0.006928
3	dbsnp	rs7650098	A	G	ALLELIC	13/7	15/35	7.292	1	0.006928
3	dbsnp	rs6549098	G	A	ALLELIC	13/7	15/35	7.292	1	0.006928
4	dbsnp	rs10030044	C	A	ALLELIC	3/17	25/25	7.292	1	0.006928
5	dbsnp	rs890825	G	A	ALLELIC	13/7	15/35	7.292	1	0.006928
5	dbsnp	rs2652210	A	G	ALLELIC	3/17	25/25	7.292	1	0.006928
6	dbsnp	rs311684	G	A	ALLELIC	3/17	25/25	7.292	1	0.006928
6	dbsnp	rs2325312	A	G	ALLELIC	13/7	15/35	7.292	1	0.006928
6	dbsnp	rs713155	A	C	ALLELIC	13/7	15/35	7.292	1	0.006928
7	dbsnp	rs4341113	A	C	ALLELIC	3/17	25/25	7.292	1	0.006928
7	dbsnp	rs6966980	A	G	ALLELIC	13/7	15/35	7.292	1	0.006928
8	dbsnp	rs11136516	C	A	ALLELIC	3/17	25/25	7.292	1	0.006928
8	dbsnp	rs2977124	A	G	ALLELIC	3/17	25/25	7.292	1	0.006928
8	dbsnp	rs7820620	G	A	ALLELIC	3/17	25/25	7.292	1	0.006928
9	dbsnp	rs12348066	G	A	ALLELIC	13/7	15/35	7.292	1	0.006928
9	dbsnp	rs7045330	A	G	ALLELIC	13/7	15/35	7.292	1	0.006928
9	dbsnp	rs7867205	A	G	ALLELIC	13/7	15/35	7.292	1	0.006928
10	dbsnp	rs10786769	G	A	ALLELIC	3/17	25/25	7.292	1	0.006928
10	dbsnp	rs2864000	G	A	ALLELIC	3/17	25/25	7.292	1	0.006928
10	dbsnp	rs10764730	G	A	ALLELIC	13/7	15/35	7.292	1	0.006928
11	dbsnp	rs10833542	A	C	ALLELIC	3/17	25/25	7.292	1	0.006928
12	dbsnp	rs10844227	G	A	ALLELIC	13/7	15/35	7.292	1	0.006928
12	dbsnp	rs11116482	A	G	ALLELIC	3/17	25/25	7.292	1	0.006928
13	dbsnp	rs9539890	A	G	ALLELIC	13/7	15/35	7.292	1	0.006928
13	dbsnp	rs2806940	G	A	ALLELIC	13/7	15/35	7.292	1	0.006928
14	dbsnp	rs4904523	G	A	ALLELIC	13/7	15/35	7.292	1	0.006928
15	dbsnp	rs6493535	A	G	ALLELIC	3/17	25/25	7.292	1	0.006928
16	dbsnp	rs368679	A	G	ALLELIC	3/17	25/25	7.292	1	0.006928
16	dbsnp	rs1895498	G	A	ALLELIC	3/17	25/25	7.292	1	0.006928
23	dbsnp	rs3897937	G	A	ALLELIC	13/7	15/35	7.292	1	0.006928
13	dbsnp	rs9554776	G	A	ALLELIC	5/13	2/44	7.291	1	0.006931
10	dbsnp	rs7090934	G	A	ALLELIC	9/11	7/41	7.259	1	0.007054
1	dbsnp	rs10864479	A	C	ALLELIC	13/9	13/37	7.251	1	0.007085
1	dbsnp	rs10753261	A	G	ALLELIC	13/9	13/37	7.251	1	0.007085
1	dbsnp	rs7533927	A	G	ALLELIC	13/9	13/37	7.251	1	0.007085
1	dbsnp	rs815343	A	G	ALLELIC	13/9	13/37	7.251	1	0.007085
1	dbsnp	rs4915342	G	A	ALLELIC	13/9	13/37	7.251	1	0.007085
1	dbsnp	rs1538027	A	C	ALLELIC	13/9	13/37	7.251	1	0.007085

1	dbsnp	rs600379	A	G	ALLELIC	13/9	13/37	7.251	1	0.007085
1	dbsnp	rs6674642	A	G	ALLELIC	13/9	13/37	7.251	1	0.007085
2	dbsnp	rs6712088	G	A	ALLELIC	13/9	13/37	7.251	1	0.007085
2	dbsnp	rs1370548	A	G	ALLELIC	13/9	13/37	7.251	1	0.007085
2	dbsnp	rs1078150	A	G	ALLELIC	13/9	13/37	7.251	1	0.007085
2	dbsnp	rs12465314	A	G	ALLELIC	13/9	13/37	7.251	1	0.007085
2	dbsnp	rs13017739	G	A	ALLELIC	13/9	13/37	7.251	1	0.007085
2	dbsnp	rs1437434	A	G	ALLELIC	13/9	13/37	7.251	1	0.007085
2	dbsnp	rs10193946	G	A	ALLELIC	13/9	13/37	7.251	1	0.007085
2	dbsnp	rs2894593	A	G	ALLELIC	13/9	13/37	7.251	1	0.007085
3	dbsnp	rs9825778	C	A	ALLELIC	13/9	13/37	7.251	1	0.007085
3	dbsnp	rs6796840	G	A	ALLELIC	13/9	13/37	7.251	1	0.007085
3	dbsnp	rs7640308	G	A	ALLELIC	13/9	13/37	7.251	1	0.007085
4	dbsnp	rs13434430	G	A	ALLELIC	13/9	13/37	7.251	1	0.007085
4	dbsnp	rs13122849	A	G	ALLELIC	13/9	13/37	7.251	1	0.007085
4	dbsnp	rs10034770	A	C	ALLELIC	13/9	13/37	7.251	1	0.007085
4	dbsnp	rs957196	A	G	ALLELIC	13/9	13/37	7.251	1	0.007085
4	dbsnp	rs1513572	G	A	ALLELIC	13/9	13/37	7.251	1	0.007085
4	dbsnp	rs10002552	G	A	ALLELIC	13/9	13/37	7.251	1	0.007085
4	dbsnp	rs2352593	G	A	ALLELIC	13/9	13/37	7.251	1	0.007085
4	dbsnp	rs11728546	A	C	ALLELIC	13/9	13/37	7.251	1	0.007085
4	dbsnp	rs6600793	A	G	ALLELIC	13/9	13/37	7.251	1	0.007085
4	dbsnp	rs6600794	A	G	ALLELIC	13/9	13/37	7.251	1	0.007085
4	dbsnp	rs11732124	A	G	ALLELIC	13/9	13/37	7.251	1	0.007085
4	dbsnp	rs1863652	A	G	ALLELIC	13/9	13/37	7.251	1	0.007085
6	dbsnp	rs1556547	G	A	ALLELIC	13/9	13/37	7.251	1	0.007085
6	dbsnp	rs12208969	G	A	ALLELIC	13/9	13/37	7.251	1	0.007085
6	dbsnp	rs6569368	G	A	ALLELIC	13/9	13/37	7.251	1	0.007085
7	dbsnp	rs4717992	G	A	ALLELIC	13/9	13/37	7.251	1	0.007085
7	dbsnp	rs2709922	A	G	ALLELIC	13/9	13/37	7.251	1	0.007085
7	dbsnp	rs4327776	G	A	ALLELIC	13/9	13/37	7.251	1	0.007085
7	dbsnp	rs6955612	G	A	ALLELIC	13/9	13/37	7.251	1	0.007085
7	dbsnp	rs1979600	A	G	ALLELIC	13/9	13/37	7.251	1	0.007085
9	dbsnp	rs2164560	G	A	ALLELIC	13/9	13/37	7.251	1	0.007085
9	dbsnp	rs4397467	G	A	ALLELIC	13/9	13/37	7.251	1	0.007085
10	dbsnp	rs1227785	A	G	ALLELIC	13/9	13/37	7.251	1	0.007085
10	dbsnp	rs10762364	A	G	ALLELIC	13/9	13/37	7.251	1	0.007085
11	dbsnp	rs2200568	G	A	ALLELIC	13/9	13/37	7.251	1	0.007085
11	dbsnp	rs10835829	G	A	ALLELIC	13/9	13/37	7.251	1	0.007085
11	dbsnp	rs675482	G	A	ALLELIC	13/9	13/37	7.251	1	0.007085
13	dbsnp	rs9590923	A	C	ALLELIC	13/9	13/37	7.251	1	0.007085
13	dbsnp	rs1932300	A	G	ALLELIC	13/9	13/37	7.251	1	0.007085
13	dbsnp	rs9520960	C	A	ALLELIC	13/9	13/37	7.251	1	0.007085
14	dbsnp	rs4151178	G	A	ALLELIC	13/9	13/37	7.251	1	0.007085
14	dbsnp	rs2025967	G	A	ALLELIC	13/9	13/37	7.251	1	0.007085
15	dbsnp	rs8036176	G	A	ALLELIC	13/9	13/37	7.251	1	0.007085
15	dbsnp	rs4886857	G	A	ALLELIC	13/9	13/37	7.251	1	0.007085
15	dbsnp	rs17385059	G	A	ALLELIC	13/9	13/37	7.251	1	0.007085

16	dbsnp	rs1868689	A	G	ALLELIC	13/9	13/37	7.251	1	0.007085
16	dbsnp	rs7184693	A	G	ALLELIC	13/9	13/37	7.251	1	0.007085
17	dbsnp	rs9915268	A	G	ALLELIC	13/9	13/37	7.251	1	0.007085
17	dbsnp	rs8069275	A	G	ALLELIC	13/9	13/37	7.251	1	0.007085
21	dbsnp	rs9979316	G	A	ALLELIC	13/9	13/37	7.251	1	0.007085
21	dbsnp	rs2094877	C	A	ALLELIC	13/9	13/37	7.251	1	0.007085
22	dbsnp	rs5756444	G	A	ALLELIC	13/9	13/37	7.251	1	0.007085
23	dbsnp	rs1815919	A	G	ALLELIC	13/9	13/37	7.251	1	0.007085
3	dbsnp	rs12629240	G	A	ALLELIC	9/13	6/42	7.231	1	0.007164
3	dbsnp	rs4145574	G	A	ALLELIC	9/13	6/42	7.231	1	0.007164
12	dbsnp	rs10784705	C	A	ALLELIC	9/13	6/42	7.231	1	0.007164
14	dbsnp	rs841	A	G	ALLELIC	9/13	6/42	7.231	1	0.007164
20	dbsnp	rs6042425	G	A	ALLELIC	9/13	6/42	7.231	1	0.007164
1	dbsnp	rs502918	G	A	ALLELIC	15/7	17/33	7.229	1	0.007172
1	dbsnp	rs7547072	G	A	ALLELIC	15/7	17/33	7.229	1	0.007172
1	dbsnp	rs7521304	A	C	ALLELIC	15/7	17/33	7.229	1	0.007172
1	dbsnp	rs9427802	G	A	ALLELIC	15/7	17/33	7.229	1	0.007172
1	dbsnp	rs6701181	G	A	ALLELIC	15/7	17/33	7.229	1	0.007172
1	dbsnp	rs1776139	C	A	ALLELIC	15/7	17/33	7.229	1	0.007172
1	dbsnp	rs1776140	A	G	ALLELIC	15/7	17/33	7.229	1	0.007172
2	dbsnp	rs6749462	A	G	ALLELIC	15/7	17/33	7.229	1	0.007172
2	dbsnp	rs6547892	A	G	ALLELIC	15/7	17/33	7.229	1	0.007172
2	dbsnp	rs4440018	A	G	ALLELIC	15/7	17/33	7.229	1	0.007172
3	dbsnp	rs4398451	G	A	ALLELIC	15/7	17/33	7.229	1	0.007172
3	dbsnp	rs11706098	C	A	ALLELIC	15/7	17/33	7.229	1	0.007172
3	dbsnp	rs1918966	A	G	ALLELIC	15/7	17/33	7.229	1	0.007172
4	dbsnp	rs6855368	A	G	ALLELIC	15/7	17/33	7.229	1	0.007172
4	dbsnp	rs11937354	G	A	ALLELIC	15/7	17/33	7.229	1	0.007172
4	dbsnp	rs151378	G	A	ALLELIC	15/7	17/33	7.229	1	0.007172
5	dbsnp	rs177108	G	A	ALLELIC	15/7	17/33	7.229	1	0.007172
5	dbsnp	rs2548331	C	A	ALLELIC	15/7	17/33	7.229	1	0.007172
5	dbsnp	rs255888	G	A	ALLELIC	15/7	17/33	7.229	1	0.007172
5	dbsnp	rs51777	G	A	ALLELIC	15/7	17/33	7.229	1	0.007172
5	dbsnp	rs10051893	A	G	ALLELIC	15/7	17/33	7.229	1	0.007172
5	dbsnp	rs17616436	A	G	ALLELIC	15/7	17/33	7.229	1	0.007172
5	dbsnp	rs32461	A	G	ALLELIC	15/7	17/33	7.229	1	0.007172
6	dbsnp	rs6921610	G	A	ALLELIC	15/7	17/33	7.229	1	0.007172
6	dbsnp	rs1319584	G	A	ALLELIC	15/7	17/33	7.229	1	0.007172
6	dbsnp	rs937327	A	G	ALLELIC	15/7	17/33	7.229	1	0.007172
7	dbsnp	rs11770502	G	A	ALLELIC	15/7	17/33	7.229	1	0.007172
7	dbsnp	rs4731007	G	A	ALLELIC	15/7	17/33	7.229	1	0.007172
7	dbsnp	rs3823523	C	A	ALLELIC	15/7	17/33	7.229	1	0.007172
8	dbsnp	rs7012076	A	G	ALLELIC	15/7	17/33	7.229	1	0.007172
8	dbsnp	rs2051274	G	A	ALLELIC	15/7	17/33	7.229	1	0.007172
8	dbsnp	rs1593501	A	C	ALLELIC	15/7	17/33	7.229	1	0.007172
9	dbsnp	rs2025557	A	G	ALLELIC	15/7	17/33	7.229	1	0.007172
9	dbsnp	rs2789876	G	A	ALLELIC	15/7	17/33	7.229	1	0.007172
9	dbsnp	rs3124782	A	G	ALLELIC	15/7	17/33	7.229	1	0.007172

9	dbsnp	rs10858121	A	G	ALLELIC	15/7	17/33	7.229	1	0.007172
10	dbsnp	rs10904927	G	A	ALLELIC	15/7	17/33	7.229	1	0.007172
10	dbsnp	rs10786719	G	A	ALLELIC	15/7	17/33	7.229	1	0.007172
10	dbsnp	rs2275271	G	A	ALLELIC	15/7	17/33	7.229	1	0.007172
10	dbsnp	rs8139	A	G	ALLELIC	15/7	17/33	7.229	1	0.007172
10	dbsnp	rs1926030	G	A	ALLELIC	15/7	17/33	7.229	1	0.007172
10	dbsnp	rs1999414	A	C	ALLELIC	15/7	17/33	7.229	1	0.007172
12	dbsnp	rs978813	A	G	ALLELIC	15/7	17/33	7.229	1	0.007172
13	dbsnp	rs9540520	A	C	ALLELIC	15/7	17/33	7.229	1	0.007172
13	dbsnp	rs11839787	A	G	ALLELIC	15/7	17/33	7.229	1	0.007172
13	dbsnp	rs9796234	G	A	ALLELIC	15/7	17/33	7.229	1	0.007172
14	dbsnp	rs2888426	C	A	ALLELIC	15/7	17/33	7.229	1	0.007172
15	dbsnp	rs2624271	G	A	ALLELIC	15/7	17/33	7.229	1	0.007172
15	dbsnp	rs11071065	A	G	ALLELIC	15/7	17/33	7.229	1	0.007172
15	dbsnp	rs4366668	A	G	ALLELIC	15/7	17/33	7.229	1	0.007172
15	dbsnp	rs2974282	A	C	ALLELIC	15/7	17/33	7.229	1	0.007172
15	dbsnp	rs12442211	G	A	ALLELIC	15/7	17/33	7.229	1	0.007172
15	dbsnp	rs8031104	G	A	ALLELIC	15/7	17/33	7.229	1	0.007172
15	dbsnp	rs10468183	G	A	ALLELIC	15/7	17/33	7.229	1	0.007172
16	dbsnp	rs237142	G	A	ALLELIC	15/7	17/33	7.229	1	0.007172
16	dbsnp	rs2875853	A	G	ALLELIC	15/7	17/33	7.229	1	0.007172
17	dbsnp	rs11079871	G	A	ALLELIC	15/7	17/33	7.229	1	0.007172
18	dbsnp	rs2584739	A	G	ALLELIC	15/7	17/33	7.229	1	0.007172
18	dbsnp	rs4941232	A	G	ALLELIC	15/7	17/33	7.229	1	0.007172
18	dbsnp	rs4940604	A	G	ALLELIC	15/7	17/33	7.229	1	0.007172
18	dbsnp	rs154880	A	G	ALLELIC	15/7	17/33	7.229	1	0.007172
19	dbsnp	rs7251403	C	A	ALLELIC	15/7	17/33	7.229	1	0.007172
19	dbsnp	rs12983988	A	G	ALLELIC	15/7	17/33	7.229	1	0.007172
21	dbsnp	rs2831507	A	C	ALLELIC	15/7	17/33	7.229	1	0.007172
21	dbsnp	rs879894	A	C	ALLELIC	15/7	17/33	7.229	1	0.007172
21	dbsnp	rs879893	A	G	ALLELIC	15/7	17/33	7.229	1	0.007172
21	dbsnp	rs4816521	G	A	ALLELIC	15/7	17/33	7.229	1	0.007172
21	dbsnp	rs8126939	A	G	ALLELIC	15/7	17/33	7.229	1	0.007172
21	dbsnp	rs4817782	C	A	ALLELIC	15/7	17/33	7.229	1	0.007172
21	dbsnp	rs1056892	A	G	ALLELIC	15/7	17/33	7.229	1	0.007172
21	dbsnp	rs2835289	A	G	ALLELIC	15/7	17/33	7.229	1	0.007172
22	dbsnp	rs134583	G	A	ALLELIC	15/7	17/33	7.229	1	0.007172
22	dbsnp	rs134651	G	A	ALLELIC	15/7	17/33	7.229	1	0.007172
22	dbsnp	rs126077	G	A	ALLELIC	15/7	17/33	7.229	1	0.007172
22	dbsnp	rs2097919	G	A	ALLELIC	15/7	17/33	7.229	1	0.007172
23	dbsnp	rs4638004	A	G	ALLELIC	15/7	17/33	7.229	1	0.007172
23	dbsnp	rs3012653	A	C	ALLELIC	15/7	17/33	7.229	1	0.007172
23	dbsnp	rs11092141	A	G	ALLELIC	15/7	17/33	7.229	1	0.007172
23	dbsnp	rs5911595	G	A	ALLELIC	15/7	17/33	7.229	1	0.007172
6	dbsnp	rs1342313	C	A	ALLELIC	3/17	0/46	7.229	1	0.007175
9	dbsnp	rs12350333	A	G	ALLELIC	3/17	0/46	7.229	1	0.007175
21	dbsnp	rs2178914	G	A	ALLELIC	3/17	24/24	7.224	1	0.007194
1	dbsnp	rs11161732	A	G	ALLELIC	11/9	11/39	7.219	1	0.007215

1	dbsnp	rs10489637	A	G	ALLELIC	11/9	11/39	7.219	1	0.007215
1	dbsnp	rs6691318	A	C	ALLELIC	11/9	11/39	7.219	1	0.007215
2	dbsnp	rs4362588	A	G	ALLELIC	11/9	11/39	7.219	1	0.007215
4	dbsnp	rs10022990	G	A	ALLELIC	11/9	11/39	7.219	1	0.007215
5	dbsnp	rs2303711	A	G	ALLELIC	11/9	11/39	7.219	1	0.007215
5	dbsnp	rs10060813	A	G	ALLELIC	11/9	11/39	7.219	1	0.007215
5	dbsnp	rs11746188	G	A	ALLELIC	11/11	9/39	7.219	1	0.007215
5	dbsnp	rs1862134	G	A	ALLELIC	11/9	11/39	7.219	1	0.007215
6	dbsnp	rs443578	A	G	ALLELIC	11/9	11/39	7.219	1	0.007215
7	dbsnp	rs6952753	A	C	ALLELIC	11/11	9/39	7.219	1	0.007215
7	dbsnp	rs2519523	A	G	ALLELIC	11/9	11/39	7.219	1	0.007215
7	dbsnp	rs4728764	A	G	ALLELIC	11/9	11/39	7.219	1	0.007215
7	dbsnp	rs2718315	A	G	ALLELIC	11/9	11/39	7.219	1	0.007215
8	dbsnp	rs6651412	G	A	ALLELIC	11/11	9/39	7.219	1	0.007215
10	dbsnp	rs2252783	A	C	ALLELIC	11/9	11/39	7.219	1	0.007215
11	dbsnp	rs553351	G	A	ALLELIC	11/9	11/39	7.219	1	0.007215
11	dbsnp	rs7120706	A	G	ALLELIC	11/9	11/39	7.219	1	0.007215
11	dbsnp	rs7120963	A	G	ALLELIC	11/9	11/39	7.219	1	0.007215
11	dbsnp	rs10892063	A	C	ALLELIC	11/9	11/39	7.219	1	0.007215
11	dbsnp	rs7120515	A	G	ALLELIC	11/9	11/39	7.219	1	0.007215
12	dbsnp	rs11177375	A	G	ALLELIC	11/9	11/39	7.219	1	0.007215
12	dbsnp	rs3730581	A	G	ALLELIC	11/9	11/39	7.219	1	0.007215
12	dbsnp	rs10859338	G	A	ALLELIC	11/9	11/39	7.219	1	0.007215
14	dbsnp	rs1958375	G	A	ALLELIC	11/9	11/39	7.219	1	0.007215
17	dbsnp	rs4792239	C	A	ALLELIC	11/9	11/39	7.219	1	0.007215
17	dbsnp	rs17514650	A	G	ALLELIC	11/11	9/39	7.219	1	0.007215
18	dbsnp	rs11665183	C	A	ALLELIC	11/9	11/39	7.219	1	0.007215
21	dbsnp	rs9976987	G	A	ALLELIC	11/9	11/39	7.219	1	0.007215
21	dbsnp	rs12482310	A	G	ALLELIC	11/9	11/39	7.219	1	0.007215
23	dbsnp	rs5958250	A	G	ALLELIC	11/9	11/39	7.219	1	0.007215
23	dbsnp	rs4824848	A	G	ALLELIC	11/11	9/39	7.219	1	0.007215
1	dbsnp	rs1360903	A	C	ALLELIC	12/8	13/37	7.193	1	0.007319
1	dbsnp	rs3856060	A	G	ALLELIC	12/8	13/37	7.193	1	0.007319
2	dbsnp	rs6750287	C	A	ALLELIC	12/8	13/37	7.193	1	0.007319
2	dbsnp	rs985697	A	G	ALLELIC	12/8	13/37	7.193	1	0.007319
2	dbsnp	rs2582974	A	G	ALLELIC	12/8	13/37	7.193	1	0.007319
2	dbsnp	rs2306276	A	G	ALLELIC	12/8	13/37	7.193	1	0.007319
2	dbsnp	rs1678160	G	A	ALLELIC	12/8	13/37	7.193	1	0.007319
2	dbsnp	rs1649883	A	G	ALLELIC	12/8	13/37	7.193	1	0.007319
2	dbsnp	rs1678168	G	A	ALLELIC	12/8	13/37	7.193	1	0.007319
3	dbsnp	rs11128627	A	G	ALLELIC	12/8	13/37	7.193	1	0.007319
5	dbsnp	rs315893	C	A	ALLELIC	12/8	13/37	7.193	1	0.007319
5	dbsnp	rs9326979	G	A	ALLELIC	12/8	13/37	7.193	1	0.007319
5	dbsnp	rs3797102	G	A	ALLELIC	12/8	13/37	7.193	1	0.007319
6	dbsnp	rs1333652	A	G	ALLELIC	12/8	13/37	7.193	1	0.007319
7	dbsnp	rs2140890	A	G	ALLELIC	12/8	13/37	7.193	1	0.007319
7	dbsnp	rs605586	A	G	ALLELIC	12/8	13/37	7.193	1	0.007319
7	dbsnp	rs1548763	G	A	ALLELIC	12/8	13/37	7.193	1	0.007319

7	dbsnp	rs4726403	A	G	ALLELIC	12/8	13/37	7.193	1	0.007319
8	dbsnp	rs13248759	A	G	ALLELIC	12/8	13/37	7.193	1	0.007319
8	dbsnp	rs2978257	A	G	ALLELIC	12/8	13/37	7.193	1	0.007319
8	dbsnp	rs6984991	C	A	ALLELIC	12/8	13/37	7.193	1	0.007319
9	dbsnp	rs7028992	G	A	ALLELIC	12/8	13/37	7.193	1	0.007319
9	dbsnp	rs7028820	G	A	ALLELIC	12/8	13/37	7.193	1	0.007319
11	dbsnp	rs11026102	A	C	ALLELIC	12/8	13/37	7.193	1	0.007319
12	dbsnp	rs1566318	A	G	ALLELIC	12/8	13/37	7.193	1	0.007319
13	dbsnp	rs537967	G	A	ALLELIC	12/8	13/37	7.193	1	0.007319
13	dbsnp	rs6563501	G	A	ALLELIC	12/8	13/37	7.193	1	0.007319
13	dbsnp	rs4943431	A	G	ALLELIC	12/8	13/37	7.193	1	0.007319
13	dbsnp	rs4482174	G	A	ALLELIC	12/8	13/37	7.193	1	0.007319
13	dbsnp	rs4772139	G	A	ALLELIC	12/8	13/37	7.193	1	0.007319
16	dbsnp	rs996091	G	A	ALLELIC	12/8	13/37	7.193	1	0.007319
18	dbsnp	rs1610037	G	A	ALLELIC	12/8	13/37	7.193	1	0.007319
18	dbsnp	rs2404869	A	G	ALLELIC	12/8	13/37	7.193	1	0.007319
21	dbsnp	rs2838616	G	A	ALLELIC	12/8	13/37	7.193	1	0.007319
23	dbsnp	rs4907820	G	A	ALLELIC	12/8	13/37	7.193	1	0.007319
1	dbsnp	rs7512495	A	G	ALLELIC	4/18	26/24	7.189	1	0.007336
1	dbsnp	rs12078899	G	A	ALLELIC	4/18	26/24	7.189	1	0.007336
1	dbsnp	rs3001365	C	A	ALLELIC	4/18	26/24	7.189	1	0.007336
1	dbsnp	rs14335	A	G	ALLELIC	4/18	26/24	7.189	1	0.007336
2	dbsnp	rs722705	G	A	ALLELIC	4/18	26/24	7.189	1	0.007336
3	dbsnp	rs295433	A	G	ALLELIC	4/18	26/24	7.189	1	0.007336
3	dbsnp	rs6787964	C	A	ALLELIC	4/18	26/24	7.189	1	0.007336
4	dbsnp	rs4689801	A	G	ALLELIC	4/18	26/24	7.189	1	0.007336
4	dbsnp	rs7680832	G	A	ALLELIC	4/18	26/24	7.189	1	0.007336
4	dbsnp	rs6534832	G	A	ALLELIC	4/18	26/24	7.189	1	0.007336
5	dbsnp	rs6876143	G	A	ALLELIC	4/18	26/24	7.189	1	0.007336
5	dbsnp	rs1393114	G	A	ALLELIC	4/18	26/24	7.189	1	0.007336
5	dbsnp	rs28474955	A	G	ALLELIC	4/18	26/24	7.189	1	0.007336
5	dbsnp	rs4704844	G	A	ALLELIC	4/18	26/24	7.189	1	0.007336
6	dbsnp	rs952571	G	A	ALLELIC	4/18	26/24	7.189	1	0.007336
6	dbsnp	rs9452333	A	G	ALLELIC	4/18	26/24	7.189	1	0.007336
6	dbsnp	rs2859307	G	A	ALLELIC	4/18	26/24	7.189	1	0.007336
6	dbsnp	rs4512241	A	C	ALLELIC	4/18	26/24	7.189	1	0.007336
6	dbsnp	rs9321951	A	G	ALLELIC	4/18	26/24	7.189	1	0.007336
6	dbsnp	rs3819812	G	A	ALLELIC	4/18	26/24	7.189	1	0.007336
8	dbsnp	rs2977078	A	G	ALLELIC	4/18	26/24	7.189	1	0.007336
8	dbsnp	rs2001721	G	A	ALLELIC	4/18	26/24	7.189	1	0.007336
8	dbsnp	rs3134216	G	A	ALLELIC	4/18	26/24	7.189	1	0.007336
8	dbsnp	rs2853172	A	G	ALLELIC	4/18	26/24	7.189	1	0.007336
9	dbsnp	rs4744600	G	A	ALLELIC	4/18	26/24	7.189	1	0.007336
9	dbsnp	rs10984725	G	A	ALLELIC	4/18	26/24	7.189	1	0.007336
9	dbsnp	rs3124781	A	G	ALLELIC	4/18	26/24	7.189	1	0.007336
10	dbsnp	rs9419445	A	G	ALLELIC	4/18	26/24	7.189	1	0.007336
10	dbsnp	rs1149994	A	G	ALLELIC	4/18	26/24	7.189	1	0.007336
10	dbsnp	rs10785943	A	C	ALLELIC	4/18	26/24	7.189	1	0.007336

11	dbsnp	rs7941517	A	G	ALLELIC	4/18	26/24	7.189	1	0.007336
11	dbsnp	rs10160665	A	C	ALLELIC	4/18	26/24	7.189	1	0.007336
11	dbsnp	rs7114555	G	A	ALLELIC	4/18	26/24	7.189	1	0.007336
11	dbsnp	rs10894384	G	A	ALLELIC	4/18	26/24	7.189	1	0.007336
13	dbsnp	rs7337666	A	C	ALLELIC	4/18	26/24	7.189	1	0.007336
16	dbsnp	rs11077313	G	A	ALLELIC	4/18	26/24	7.189	1	0.007336
16	dbsnp	rs907022	G	A	ALLELIC	4/18	26/24	7.189	1	0.007336
17	dbsnp	rs6502670	C	A	ALLELIC	4/18	26/24	7.189	1	0.007336
17	dbsnp	rs1058381	A	G	ALLELIC	4/18	26/24	7.189	1	0.007336
17	dbsnp	rs1058398	G	A	ALLELIC	4/18	26/24	7.189	1	0.007336
17	dbsnp	rs739768	A	G	ALLELIC	4/18	26/24	7.189	1	0.007336
17	dbsnp	rs1806239	G	A	ALLELIC	4/18	26/24	7.189	1	0.007336
17	dbsnp	rs757351	A	C	ALLELIC	4/18	26/24	7.189	1	0.007336
17	dbsnp	rs9900353	G	A	ALLELIC	4/18	26/24	7.189	1	0.007336
17	dbsnp	rs8080721	G	A	ALLELIC	4/18	26/24	7.189	1	0.007336
19	dbsnp	rs730427	G	A	ALLELIC	4/18	26/24	7.189	1	0.007336
23	dbsnp	rs4570314	G	A	ALLELIC	4/18	26/24	7.189	1	0.007336
23	dbsnp	rs5930921	A	C	ALLELIC	4/18	26/24	7.189	1	0.007336
1	dbsnp	rs9424310	A	G	ALLELIC	14/8	15/35	7.186	1	0.007348
1	dbsnp	rs10794665	A	G	ALLELIC	14/8	15/35	7.186	1	0.007348
1	dbsnp	rs3795302	A	G	ALLELIC	14/8	15/35	7.186	1	0.007348
1	dbsnp	rs834350	G	A	ALLELIC	14/8	15/35	7.186	1	0.007348
1	dbsnp	rs12726057	A	G	ALLELIC	14/8	15/35	7.186	1	0.007348
2	dbsnp	rs992725	G	A	ALLELIC	14/8	15/35	7.186	1	0.007348
2	dbsnp	rs13398058	G	A	ALLELIC	14/8	15/35	7.186	1	0.007348
3	dbsnp	rs2358930	G	A	ALLELIC	14/8	15/35	7.186	1	0.007348
3	dbsnp	rs2839770	A	C	ALLELIC	14/8	15/35	7.186	1	0.007348
4	dbsnp	rs7672573	A	G	ALLELIC	14/8	15/35	7.186	1	0.007348
4	dbsnp	rs2008438	C	A	ALLELIC	14/8	15/35	7.186	1	0.007348
4	dbsnp	rs4356965	A	G	ALLELIC	14/8	15/35	7.186	1	0.007348
4	dbsnp	rs2593079	A	G	ALLELIC	14/8	15/35	7.186	1	0.007348
4	dbsnp	rs706341	C	A	ALLELIC	14/8	15/35	7.186	1	0.007348
5	dbsnp	rs13357659	G	A	ALLELIC	14/8	15/35	7.186	1	0.007348
5	dbsnp	rs10223052	A	G	ALLELIC	14/8	15/35	7.186	1	0.007348
5	dbsnp	rs34309	A	G	ALLELIC	14/8	15/35	7.186	1	0.007348
5	dbsnp	rs930875	A	G	ALLELIC	14/8	15/35	7.186	1	0.007348
6	dbsnp	rs9392322	G	A	ALLELIC	14/8	15/35	7.186	1	0.007348
6	dbsnp	rs1025534	A	G	ALLELIC	14/8	15/35	7.186	1	0.007348
6	dbsnp	rs9384526	A	G	ALLELIC	14/8	15/35	7.186	1	0.007348
6	dbsnp	rs1764059	A	G	ALLELIC	14/8	15/35	7.186	1	0.007348
6	dbsnp	rs6911413	A	C	ALLELIC	14/8	15/35	7.186	1	0.007348
7	dbsnp	rs7806030	G	A	ALLELIC	14/8	15/35	7.186	1	0.007348
7	dbsnp	rs1881722	G	A	ALLELIC	14/8	15/35	7.186	1	0.007348
9	dbsnp	rs1407977	A	G	ALLELIC	14/8	15/35	7.186	1	0.007348
9	dbsnp	rs3808707	A	G	ALLELIC	14/8	15/35	7.186	1	0.007348
10	dbsnp	rs3125037	G	A	ALLELIC	14/8	15/35	7.186	1	0.007348
10	dbsnp	rs10795415	A	G	ALLELIC	14/8	15/35	7.186	1	0.007348
10	dbsnp	rs1992821	C	A	ALLELIC	14/8	15/35	7.186	1	0.007348

11	dbsnp	rs3750992	C	A	ALLELIC	14/8	15/35	7.186	1	0.007348
11	dbsnp	rs17314159	A	C	ALLELIC	14/8	15/35	7.186	1	0.007348
11	dbsnp	rs16937906	A	C	ALLELIC	14/8	15/35	7.186	1	0.007348
11	dbsnp	rs224604	C	A	ALLELIC	14/8	15/35	7.186	1	0.007348
11	dbsnp	rs4754604	G	A	ALLELIC	14/8	15/35	7.186	1	0.007348
11	dbsnp	rs11219590	G	A	ALLELIC	14/8	15/35	7.186	1	0.007348
11	dbsnp	rs7930825	G	A	ALLELIC	14/8	15/35	7.186	1	0.007348
11	dbsnp	rs588472	A	G	ALLELIC	14/8	15/35	7.186	1	0.007348
11	dbsnp	rs7925428	G	A	ALLELIC	14/8	15/35	7.186	1	0.007348
12	dbsnp	rs7306438	A	G	ALLELIC	14/8	15/35	7.186	1	0.007348
12	dbsnp	rs6580967	A	C	ALLELIC	14/8	15/35	7.186	1	0.007348
12	dbsnp	rs3843640	C	A	ALLELIC	14/8	15/35	7.186	1	0.007348
13	dbsnp	rs2265775	A	C	ALLELIC	14/8	15/35	7.186	1	0.007348
13	dbsnp	rs7319981	A	G	ALLELIC	14/8	15/35	7.186	1	0.007348
13	dbsnp	rs7324840	G	A	ALLELIC	14/8	15/35	7.186	1	0.007348
14	dbsnp	rs2207243	G	A	ALLELIC	14/8	15/35	7.186	1	0.007348
14	dbsnp	rs3007133	A	G	ALLELIC	14/8	15/35	7.186	1	0.007348
14	dbsnp	rs1147437	C	A	ALLELIC	14/8	15/35	7.186	1	0.007348
15	dbsnp	rs3784562	G	A	ALLELIC	14/8	15/35	7.186	1	0.007348
15	dbsnp	rs11858435	A	G	ALLELIC	14/8	15/35	7.186	1	0.007348
15	dbsnp	rs1159895	A	G	ALLELIC	14/8	15/35	7.186	1	0.007348
15	dbsnp	rs903971	G	A	ALLELIC	14/8	15/35	7.186	1	0.007348
16	dbsnp	rs1014126	A	G	ALLELIC	14/8	15/35	7.186	1	0.007348
16	dbsnp	rs11643080	G	A	ALLELIC	14/8	15/35	7.186	1	0.007348
16	dbsnp	rs1110470	A	G	ALLELIC	14/8	15/35	7.186	1	0.007348
17	dbsnp	rs175391	A	C	ALLELIC	14/8	15/35	7.186	1	0.007348
17	dbsnp	rs1034923	A	G	ALLELIC	14/8	15/35	7.186	1	0.007348
17	dbsnp	rs4969170	A	G	ALLELIC	14/8	15/35	7.186	1	0.007348
18	dbsnp	rs8091927	C	A	ALLELIC	14/8	15/35	7.186	1	0.007348
18	dbsnp	rs4366786	A	C	ALLELIC	14/8	15/35	7.186	1	0.007348
18	dbsnp	rs1945475	A	G	ALLELIC	14/8	15/35	7.186	1	0.007348
19	dbsnp	rs1529729	A	G	ALLELIC	14/8	15/35	7.186	1	0.007348
19	dbsnp	rs50871	C	A	ALLELIC	14/8	15/35	7.186	1	0.007348
20	dbsnp	rs4642012	A	C	ALLELIC	14/8	15/35	7.186	1	0.007348
20	dbsnp	rs761267	C	A	ALLELIC	14/8	15/35	7.186	1	0.007348
21	dbsnp	rs2409758	A	G	ALLELIC	14/8	15/35	7.186	1	0.007348
22	dbsnp	rs11090644	C	A	ALLELIC	14/8	15/35	7.186	1	0.007348
23	dbsnp	rs1279795	A	G	ALLELIC	14/8	15/35	7.186	1	0.007348
23	dbsnp	rs3900169	C	A	ALLELIC	14/8	15/35	7.186	1	0.007348
12	dbsnp	rs4767259	A	G	ALLELIC	2/18	21/27	7.184	1	0.007354
1	dbsnp	rs2275819	A	G	ALLELIC	8/14	5/45	7.177	1	0.007385
1	dbsnp	rs11184747	G	A	ALLELIC	8/14	5/45	7.177	1	0.007385
1	dbsnp	rs8453	A	C	ALLELIC	8/14	5/45	7.177	1	0.007385
1	dbsnp	rs10494322	G	A	ALLELIC	8/14	5/45	7.177	1	0.007385
1	dbsnp	rs767053	A	G	ALLELIC	8/14	5/45	7.177	1	0.007385
1	dbsnp	rs6703547	A	G	ALLELIC	8/14	5/45	7.177	1	0.007385
1	dbsnp	rs16846667	A	G	ALLELIC	8/14	5/45	7.177	1	0.007385
1	dbsnp	rs12565430	A	G	ALLELIC	8/14	5/45	7.177	1	0.007385

1	dbsnp	rs16847271	A	C	ALLELIC	8/14	5/45	7.177	1	0.007385
1	dbsnp	rs2500499	A	G	ALLELIC	8/14	5/45	7.177	1	0.007385
2	dbsnp	rs1439964	A	G	ALLELIC	8/14	5/45	7.177	1	0.007385
2	dbsnp	rs16863159	C	A	ALLELIC	8/14	5/45	7.177	1	0.007385
2	dbsnp	rs13385952	G	A	ALLELIC	8/14	5/45	7.177	1	0.007385
2	dbsnp	rs4538235	G	A	ALLELIC	8/14	5/45	7.177	1	0.007385
2	dbsnp	rs4662692	A	G	ALLELIC	8/14	5/45	7.177	1	0.007385
2	dbsnp	rs11887389	C	A	ALLELIC	8/14	5/45	7.177	1	0.007385
2	dbsnp	rs2111720	G	A	ALLELIC	8/14	5/45	7.177	1	0.007385
2	dbsnp	rs10490749	A	G	ALLELIC	8/14	5/45	7.177	1	0.007385
2	dbsnp	rs4585026	A	G	ALLELIC	8/14	5/45	7.177	1	0.007385
2	dbsnp	rs1574030	A	G	ALLELIC	8/14	5/45	7.177	1	0.007385
2	dbsnp	rs6543586	C	A	ALLELIC	8/14	5/45	7.177	1	0.007385
3	dbsnp	rs2892212	G	A	ALLELIC	8/14	5/45	7.177	1	0.007385
3	dbsnp	rs1073657	A	G	ALLELIC	8/14	5/45	7.177	1	0.007385
3	dbsnp	rs2600268	G	A	ALLELIC	8/14	5/45	7.177	1	0.007385
3	dbsnp	rs373827	G	A	ALLELIC	8/14	5/45	7.177	1	0.007385
3	dbsnp	rs6770055	G	A	ALLELIC	8/14	5/45	7.177	1	0.007385
3	dbsnp	rs6445588	A	G	ALLELIC	8/14	5/45	7.177	1	0.007385
3	dbsnp	rs11130409	C	A	ALLELIC	8/14	5/45	7.177	1	0.007385
3	dbsnp	rs10439957	A	G	ALLELIC	8/14	5/45	7.177	1	0.007385
3	dbsnp	rs12488945	G	A	ALLELIC	8/14	5/45	7.177	1	0.007385
3	dbsnp	rs12486909	A	G	ALLELIC	8/14	5/45	7.177	1	0.007385
3	dbsnp	rs11914342	A	C	ALLELIC	8/14	5/45	7.177	1	0.007385
4	dbsnp	rs10025855	A	G	ALLELIC	8/14	5/45	7.177	1	0.007385
4	dbsnp	rs1513935	A	G	ALLELIC	8/14	5/45	7.177	1	0.007385
4	dbsnp	rs13150445	C	A	ALLELIC	8/14	5/45	7.177	1	0.007385
4	dbsnp	rs4447889	C	A	ALLELIC	8/14	5/45	7.177	1	0.007385
4	dbsnp	rs11100509	A	G	ALLELIC	8/14	5/45	7.177	1	0.007385
5	dbsnp	rs462906	A	G	ALLELIC	8/14	5/45	7.177	1	0.007385
5	dbsnp	rs458006	A	C	ALLELIC	8/14	5/45	7.177	1	0.007385
5	dbsnp	rs10060537	A	G	ALLELIC	8/14	5/45	7.177	1	0.007385
5	dbsnp	rs2624435	A	G	ALLELIC	8/14	5/45	7.177	1	0.007385
5	dbsnp	rs400042	A	G	ALLELIC	8/14	5/45	7.177	1	0.007385
5	dbsnp	rs10074805	A	G	ALLELIC	8/14	5/45	7.177	1	0.007385
5	dbsnp	rs7736339	C	A	ALLELIC	8/14	5/45	7.177	1	0.007385
5	dbsnp	rs1472357	G	A	ALLELIC	8/14	5/45	7.177	1	0.007385
5	dbsnp	rs1472356	A	G	ALLELIC	8/14	5/45	7.177	1	0.007385
5	dbsnp	rs7734214	A	G	ALLELIC	8/14	5/45	7.177	1	0.007385
5	dbsnp	rs7727165	A	C	ALLELIC	8/14	5/45	7.177	1	0.007385
6	dbsnp	rs6905506	G	A	ALLELIC	8/14	5/45	7.177	1	0.007385
6	dbsnp	rs501948	C	A	ALLELIC	8/14	5/45	7.177	1	0.007385
6	dbsnp	rs126405	A	G	ALLELIC	8/14	5/45	7.177	1	0.007385
6	dbsnp	rs4715959	A	G	ALLELIC	8/14	5/45	7.177	1	0.007385
6	dbsnp	rs4712446	A	G	ALLELIC	8/14	5/45	7.177	1	0.007385
6	dbsnp	rs7747549	A	G	ALLELIC	8/14	5/45	7.177	1	0.007385
6	dbsnp	rs10945147	A	G	ALLELIC	8/14	5/45	7.177	1	0.007385
6	dbsnp	rs2342763	A	G	ALLELIC	8/14	5/45	7.177	1	0.007385

6	dbsnp	rs1573190	A	G	ALLELIC	8/14	5/45	7.177	1	0.007385
6	dbsnp	rs7772098	A	G	ALLELIC	8/14	5/45	7.177	1	0.007385
6	dbsnp	rs12207886	A	G	ALLELIC	8/14	5/45	7.177	1	0.007385
6	dbsnp	rs2069128	A	G	ALLELIC	8/14	5/45	7.177	1	0.007385
6	dbsnp	rs12198739	A	G	ALLELIC	8/14	5/45	7.177	1	0.007385
7	dbsnp	rs3750012	G	A	ALLELIC	8/14	5/45	7.177	1	0.007385
7	dbsnp	rs11983337	A	G	ALLELIC	8/14	5/45	7.177	1	0.007385
7	dbsnp	rs3793263	A	C	ALLELIC	8/14	5/45	7.177	1	0.007385
7	dbsnp	rs6463265	A	G	ALLELIC	8/14	5/45	7.177	1	0.007385
7	dbsnp	rs156966	C	A	ALLELIC	8/14	5/45	7.177	1	0.007385
7	dbsnp	rs156969	A	C	ALLELIC	8/14	5/45	7.177	1	0.007385
7	dbsnp	rs2536076	G	A	ALLELIC	8/14	5/45	7.177	1	0.007385
8	dbsnp	rs492083	G	A	ALLELIC	8/14	5/45	7.177	1	0.007385
8	dbsnp	rs939341	C	A	ALLELIC	8/14	5/45	7.177	1	0.007385
8	dbsnp	rs2611367	A	G	ALLELIC	8/14	5/45	7.177	1	0.007385
8	dbsnp	rs4140855	A	G	ALLELIC	8/14	5/45	7.177	1	0.007385
8	dbsnp	rs12156416	A	G	ALLELIC	8/14	5/45	7.177	1	0.007385
8	dbsnp	rs11776528	A	G	ALLELIC	8/14	5/45	7.177	1	0.007385
8	dbsnp	rs12541052	A	G	ALLELIC	8/14	5/45	7.177	1	0.007385
8	dbsnp	rs4354346	A	G	ALLELIC	8/14	5/45	7.177	1	0.007385
8	dbsnp	rs6982188	A	G	ALLELIC	8/14	5/45	7.177	1	0.007385
9	dbsnp	rs11791131	A	G	ALLELIC	8/14	5/45	7.177	1	0.007385
9	dbsnp	rs1923431	G	A	ALLELIC	8/14	5/45	7.177	1	0.007385
9	dbsnp	rs1616208	A	G	ALLELIC	8/14	5/45	7.177	1	0.007385
10	dbsnp	rs1779384	A	C	ALLELIC	8/14	5/45	7.177	1	0.007385
10	dbsnp	rs997066	A	G	ALLELIC	8/14	5/45	7.177	1	0.007385
10	dbsnp	rs1758816	G	A	ALLELIC	8/14	5/45	7.177	1	0.007385
10	dbsnp	rs7916611	C	A	ALLELIC	8/14	5/45	7.177	1	0.007385
10	dbsnp	rs7920207	A	C	ALLELIC	8/14	5/45	7.177	1	0.007385
11	dbsnp	rs2238009	A	G	ALLELIC	8/14	5/45	7.177	1	0.007385
12	dbsnp	rs9668454	G	A	ALLELIC	8/14	5/45	7.177	1	0.007385
12	dbsnp	rs3852540	G	A	ALLELIC	8/14	5/45	7.177	1	0.007385
12	dbsnp	rs11838060	A	C	ALLELIC	8/14	5/45	7.177	1	0.007385
12	dbsnp	rs1544396	G	A	ALLELIC	8/14	5/45	7.177	1	0.007385
12	dbsnp	rs2285727	A	C	ALLELIC	8/14	5/45	7.177	1	0.007385
12	dbsnp	rs609230	A	G	ALLELIC	8/14	5/45	7.177	1	0.007385
12	dbsnp	rs640783	G	A	ALLELIC	8/14	5/45	7.177	1	0.007385
12	dbsnp	rs7962138	G	A	ALLELIC	8/14	5/45	7.177	1	0.007385
12	dbsnp	rs12812413	A	G	ALLELIC	8/14	5/45	7.177	1	0.007385
12	dbsnp	rs7968444	C	A	ALLELIC	8/14	5/45	7.177	1	0.007385
13	dbsnp	rs9511662	A	G	ALLELIC	8/14	5/45	7.177	1	0.007385
13	dbsnp	rs4943748	A	C	ALLELIC	8/14	5/45	7.177	1	0.007385
13	dbsnp	rs3783045	G	A	ALLELIC	8/14	5/45	7.177	1	0.007385
13	dbsnp	rs9564596	C	A	ALLELIC	8/14	5/45	7.177	1	0.007385
14	dbsnp	rs11158028	A	G	ALLELIC	8/14	5/45	7.177	1	0.007385
14	dbsnp	rs761951	C	A	ALLELIC	8/14	5/45	7.177	1	0.007385
14	dbsnp	rs2372243	A	G	ALLELIC	8/14	5/45	7.177	1	0.007385
14	dbsnp	rs7158484	A	G	ALLELIC	8/14	5/45	7.177	1	0.007385

14	dbsnp	rs807732	G	A	ALLELIC	8/14	5/45	7.177	1	0.007385
15	dbsnp	rs11161189	A	G	ALLELIC	8/14	5/45	7.177	1	0.007385
15	dbsnp	rs602302	A	G	ALLELIC	8/14	5/45	7.177	1	0.007385
15	dbsnp	rs6495442	G	A	ALLELIC	8/14	5/45	7.177	1	0.007385
15	dbsnp	rs12443084	A	C	ALLELIC	8/14	5/45	7.177	1	0.007385
15	dbsnp	rs6493090	A	C	ALLELIC	8/14	5/45	7.177	1	0.007385
15	dbsnp	rs4454949	A	G	ALLELIC	8/14	5/45	7.177	1	0.007385
15	dbsnp	rs10438437	A	G	ALLELIC	8/14	5/45	7.177	1	0.007385
15	dbsnp	rs9972576	G	A	ALLELIC	8/14	5/45	7.177	1	0.007385
15	dbsnp	rs4777936	G	A	ALLELIC	8/14	5/45	7.177	1	0.007385
16	dbsnp	rs16940835	G	A	ALLELIC	8/14	5/45	7.177	1	0.007385
17	dbsnp	rs12449606	A	C	ALLELIC	8/14	5/45	7.177	1	0.007385
17	dbsnp	rs16975266	G	A	ALLELIC	8/14	5/45	7.177	1	0.007385
18	dbsnp	rs1941137	A	G	ALLELIC	8/14	5/45	7.177	1	0.007385
18	dbsnp	rs786019	A	G	ALLELIC	8/14	5/45	7.177	1	0.007385
18	dbsnp	rs9955645	G	A	ALLELIC	8/14	5/45	7.177	1	0.007385
18	dbsnp	rs4890615	A	G	ALLELIC	8/14	5/45	7.177	1	0.007385
18	dbsnp	rs4243311	G	A	ALLELIC	8/14	5/45	7.177	1	0.007385
19	dbsnp	rs7251857	G	A	ALLELIC	8/14	5/45	7.177	1	0.007385
20	dbsnp	rs6108160	A	G	ALLELIC	8/14	5/45	7.177	1	0.007385
20	dbsnp	rs6057603	A	G	ALLELIC	8/14	5/45	7.177	1	0.007385
21	dbsnp	rs2836447	A	G	ALLELIC	8/14	5/45	7.177	1	0.007385
23	dbsnp	rs5949952	G	A	ALLELIC	8/14	5/45	7.177	1	0.007385
23	dbsnp	rs6523307	G	A	ALLELIC	8/14	5/45	7.177	1	0.007385
23	dbsnp	rs2061662	A	G	ALLELIC	8/14	5/45	7.177	1	0.007385
23	dbsnp	rs2061663	G	A	ALLELIC	8/14	5/45	7.177	1	0.007385
23	dbsnp	rs5987626	A	G	ALLELIC	8/14	5/45	7.177	1	0.007385
23	dbsnp	rs5987497	G	A	ALLELIC	8/14	5/45	7.177	1	0.007385
23	dbsnp	rs585602	A	G	ALLELIC	8/14	5/45	7.177	1	0.007385
23	dbsnp	rs5905023	A	C	ALLELIC	8/14	5/45	7.177	1	0.007385
7	dbsnp	rs4577890	C	A	ALLELIC	15/5	18/28	7.174	1	0.007397
19	dbsnp	rs745831	A	C	ALLELIC	9/7	9/35	7.159	1	0.007458
13	dbsnp	rs4770114	A	G	ALLELIC	3/17	23/23	7.152	1	0.007488
6	dbsnp	rs2268447	A	G	ALLELIC	4/18	25/23	7.145	1	0.007516
13	dbsnp	rs2806939	A	G	ALLELIC	4/18	25/23	7.145	1	0.007516
23	dbsnp	rs6625563	A	G	ALLELIC	4/18	25/23	7.145	1	0.007516
20	dbsnp	rs11905011	C	A	ALLELIC	8/10	7/43	7.135	1	0.007559
1	dbsnp	rs4908637	A	G	ALLELIC	3/19	0/50	7.115	1	0.007646
1	dbsnp	rs6429675	A	C	ALLELIC	3/19	0/50	7.115	1	0.007646
1	dbsnp	rs12065087	A	C	ALLELIC	3/19	0/50	7.115	1	0.007646
1	dbsnp	rs1188641	C	A	ALLELIC	3/19	0/50	7.115	1	0.007646
1	dbsnp	rs10157108	C	A	ALLELIC	3/19	0/50	7.115	1	0.007646
1	dbsnp	rs658147	G	A	ALLELIC	3/19	0/50	7.115	1	0.007646
1	dbsnp	rs11263833	A	C	ALLELIC	3/19	0/50	7.115	1	0.007646
1	dbsnp	rs2296470	G	A	ALLELIC	3/19	0/50	7.115	1	0.007646
1	dbsnp	rs11263839	C	A	ALLELIC	3/19	0/50	7.115	1	0.007646
1	dbsnp	rs617673	C	A	ALLELIC	3/19	0/50	7.115	1	0.007646
1	dbsnp	rs12026699	C	A	ALLELIC	3/19	0/50	7.115	1	0.007646

1	dbsnp	rs274740	A	G	ALLELIC	3/19	0/50	7.115	1	0.007646
1	dbsnp	rs274741	G	A	ALLELIC	3/19	0/50	7.115	1	0.007646
1	dbsnp	rs274750	A	C	ALLELIC	3/19	0/50	7.115	1	0.007646
1	dbsnp	rs274751	G	A	ALLELIC	3/19	0/50	7.115	1	0.007646
1	dbsnp	rs274752	G	A	ALLELIC	3/19	0/50	7.115	1	0.007646
1	dbsnp	rs272817	G	A	ALLELIC	3/19	0/50	7.115	1	0.007646
1	dbsnp	rs17099885	G	A	ALLELIC	3/19	0/50	7.115	1	0.007646
1	dbsnp	rs17100075	G	A	ALLELIC	3/19	0/50	7.115	1	0.007646
1	dbsnp	rs7534033	G	A	ALLELIC	3/19	0/50	7.115	1	0.007646
1	dbsnp	rs1721165	A	G	ALLELIC	3/19	0/50	7.115	1	0.007646
1	dbsnp	rs1779177	G	A	ALLELIC	3/19	0/50	7.115	1	0.007646
1	dbsnp	rs1167198	G	A	ALLELIC	3/19	0/50	7.115	1	0.007646
1	dbsnp	rs11162319	G	A	ALLELIC	3/19	0/50	7.115	1	0.007646
1	dbsnp	rs3000112	A	C	ALLELIC	3/19	0/50	7.115	1	0.007646
1	dbsnp	rs3000156	A	G	ALLELIC	3/19	0/50	7.115	1	0.007646
1	dbsnp	rs3015077	G	A	ALLELIC	3/19	0/50	7.115	1	0.007646
1	dbsnp	rs1353287	G	A	ALLELIC	3/19	0/50	7.115	1	0.007646
1	dbsnp	rs10518537	A	G	ALLELIC	3/19	0/50	7.115	1	0.007646
1	dbsnp	rs17113362	A	G	ALLELIC	3/19	0/50	7.115	1	0.007646
1	dbsnp	rs6658632	A	G	ALLELIC	3/19	0/50	7.115	1	0.007646
1	dbsnp	rs7540424	G	A	ALLELIC	3/19	0/50	7.115	1	0.007646
1	dbsnp	rs10923243	G	A	ALLELIC	3/19	0/50	7.115	1	0.007646
1	dbsnp	rs16835600	G	A	ALLELIC	3/19	0/50	7.115	1	0.007646
1	dbsnp	rs11264446	A	G	ALLELIC	3/19	0/50	7.115	1	0.007646
1	dbsnp	rs10908561	G	A	ALLELIC	3/19	0/50	7.115	1	0.007646
1	dbsnp	rs11264712	G	A	ALLELIC	3/19	0/50	7.115	1	0.007646
1	dbsnp	rs10908563	G	A	ALLELIC	3/19	0/50	7.115	1	0.007646
1	dbsnp	rs1387353	G	A	ALLELIC	3/19	0/50	7.115	1	0.007646
1	dbsnp	rs4652591	G	A	ALLELIC	3/19	0/50	7.115	1	0.007646
1	dbsnp	rs6687494	C	A	ALLELIC	3/19	0/50	7.115	1	0.007646
1	dbsnp	rs3753990	A	G	ALLELIC	3/19	0/50	7.115	1	0.007646
1	dbsnp	rs1999132	G	A	ALLELIC	3/19	0/50	7.115	1	0.007646
1	dbsnp	rs17018414	C	A	ALLELIC	3/19	0/50	7.115	1	0.007646
2	dbsnp	rs4669361	A	G	ALLELIC	3/19	0/50	7.115	1	0.007646
2	dbsnp	rs1429410	C	A	ALLELIC	3/19	0/50	7.115	1	0.007646
2	dbsnp	rs16982805	G	A	ALLELIC	3/19	0/50	7.115	1	0.007646
2	dbsnp	rs1465832	G	A	ALLELIC	3/19	0/50	7.115	1	0.007646
2	dbsnp	rs6725784	A	G	ALLELIC	3/19	0/50	7.115	1	0.007646
2	dbsnp	rs11898704	G	A	ALLELIC	3/19	0/50	7.115	1	0.007646
2	dbsnp	rs6712399	A	G	ALLELIC	3/19	0/50	7.115	1	0.007646
2	dbsnp	rs11695220	G	A	ALLELIC	3/19	0/50	7.115	1	0.007646
2	dbsnp	rs11685825	G	A	ALLELIC	3/19	0/50	7.115	1	0.007646
2	dbsnp	rs1105595	A	G	ALLELIC	3/19	0/50	7.115	1	0.007646
2	dbsnp	rs10469935	C	A	ALLELIC	3/19	0/50	7.115	1	0.007646
2	dbsnp	rs7570117	C	A	ALLELIC	3/19	0/50	7.115	1	0.007646
2	dbsnp	rs6751871	G	A	ALLELIC	3/19	0/50	7.115	1	0.007646
2	dbsnp	rs1105865	G	A	ALLELIC	3/19	0/50	7.115	1	0.007646
2	dbsnp	rs7423827	G	A	ALLELIC	3/19	0/50	7.115	1	0.007646

2	dbsnp	rs12472370	G	A	ALLELIC	3/19	0/50	7.115	1	0.007646
2	dbsnp	rs12470170	A	C	ALLELIC	3/19	0/50	7.115	1	0.007646
2	dbsnp	rs2090806	A	G	ALLELIC	3/19	0/50	7.115	1	0.007646
2	dbsnp	rs1719079	G	A	ALLELIC	3/19	0/50	7.115	1	0.007646
2	dbsnp	rs16854770	A	C	ALLELIC	3/19	0/50	7.115	1	0.007646
2	dbsnp	rs698258	A	G	ALLELIC	3/19	0/50	7.115	1	0.007646
2	dbsnp	rs16866737	G	A	ALLELIC	3/19	0/50	7.115	1	0.007646
2	dbsnp	rs7593100	A	G	ALLELIC	3/19	0/50	7.115	1	0.007646
2	dbsnp	rs16823799	A	G	ALLELIC	3/19	0/50	7.115	1	0.007646
2	dbsnp	rs10804072	C	A	ALLELIC	3/19	0/50	7.115	1	0.007646
2	dbsnp	rs16866988	A	G	ALLELIC	3/19	0/50	7.115	1	0.007646
2	dbsnp	rs6704649	G	A	ALLELIC	3/19	0/50	7.115	1	0.007646
2	dbsnp	rs1013243	A	C	ALLELIC	3/19	0/50	7.115	1	0.007646
2	dbsnp	rs17868387	G	A	ALLELIC	3/19	0/50	7.115	1	0.007646
2	dbsnp	rs28948671	A	C	ALLELIC	3/19	0/50	7.115	1	0.007646
2	dbsnp	rs2042831	A	G	ALLELIC	3/19	0/50	7.115	1	0.007646
3	dbsnp	rs908603	A	G	ALLELIC	3/19	0/50	7.115	1	0.007646
3	dbsnp	rs7614027	G	A	ALLELIC	3/19	0/50	7.115	1	0.007646
3	dbsnp	rs2010265	G	A	ALLELIC	3/19	0/50	7.115	1	0.007646
3	dbsnp	rs17041032	A	G	ALLELIC	3/19	0/50	7.115	1	0.007646
3	dbsnp	rs9310823	G	A	ALLELIC	3/19	0/50	7.115	1	0.007646
3	dbsnp	rs33480	G	A	ALLELIC	3/19	0/50	7.115	1	0.007646
3	dbsnp	rs1814906	G	A	ALLELIC	3/19	0/50	7.115	1	0.007646
3	dbsnp	rs4974142	A	G	ALLELIC	3/19	0/50	7.115	1	0.007646
3	dbsnp	rs921162	G	A	ALLELIC	3/19	0/50	7.115	1	0.007646
3	dbsnp	rs9848161	A	G	ALLELIC	3/19	0/50	7.115	1	0.007646
3	dbsnp	rs9813741	A	G	ALLELIC	3/19	0/50	7.115	1	0.007646
3	dbsnp	rs9841232	A	G	ALLELIC	3/19	0/50	7.115	1	0.007646
3	dbsnp	rs17016763	G	A	ALLELIC	3/19	0/50	7.115	1	0.007646
3	dbsnp	rs1492869	G	A	ALLELIC	3/19	0/50	7.115	1	0.007646
3	dbsnp	rs1492865	G	A	ALLELIC	3/19	0/50	7.115	1	0.007646
3	dbsnp	rs3853594	A	G	ALLELIC	3/19	0/50	7.115	1	0.007646
3	dbsnp	rs16825084	G	A	ALLELIC	3/19	0/50	7.115	1	0.007646
3	dbsnp	rs3851384	A	G	ALLELIC	3/19	0/50	7.115	1	0.007646
4	dbsnp	rs1179042	A	G	ALLELIC	3/19	0/50	7.115	1	0.007646
4	dbsnp	rs13109196	A	G	ALLELIC	3/19	0/50	7.115	1	0.007646
4	dbsnp	rs6811040	A	G	ALLELIC	3/19	0/50	7.115	1	0.007646
4	dbsnp	rs1473094	A	G	ALLELIC	3/19	0/50	7.115	1	0.007646
4	dbsnp	rs1512160	A	C	ALLELIC	3/19	0/50	7.115	1	0.007646
4	dbsnp	rs10019706	G	A	ALLELIC	3/19	0/50	7.115	1	0.007646
4	dbsnp	rs9994236	A	G	ALLELIC	3/19	0/50	7.115	1	0.007646
4	dbsnp	rs13115999	G	A	ALLELIC	3/19	0/50	7.115	1	0.007646
4	dbsnp	rs6448713	A	G	ALLELIC	3/19	0/50	7.115	1	0.007646
4	dbsnp	rs7671654	G	A	ALLELIC	3/19	0/50	7.115	1	0.007646
4	dbsnp	rs11726010	A	G	ALLELIC	3/19	0/50	7.115	1	0.007646
4	dbsnp	rs10007812	A	G	ALLELIC	3/19	0/50	7.115	1	0.007646
4	dbsnp	rs3849020	A	G	ALLELIC	3/19	0/50	7.115	1	0.007646
4	dbsnp	rs9968502	A	G	ALLELIC	3/19	0/50	7.115	1	0.007646

4	dbsnp	rs11946541	A	G	ALLELIC	3/19	0/50	7.115	1	0.007646
4	dbsnp	rs10010155	A	G	ALLELIC	3/19	0/50	7.115	1	0.007646
4	dbsnp	rs13103779	A	G	ALLELIC	3/19	0/50	7.115	1	0.007646
4	dbsnp	rs2702331	G	A	ALLELIC	3/19	0/50	7.115	1	0.007646
4	dbsnp	rs2593087	G	A	ALLELIC	3/19	0/50	7.115	1	0.007646
4	dbsnp	rs16847840	C	A	ALLELIC	3/19	0/50	7.115	1	0.007646
4	dbsnp	rs1369093	G	A	ALLELIC	3/19	0/50	7.115	1	0.007646
4	dbsnp	rs10006753	A	G	ALLELIC	3/19	0/50	7.115	1	0.007646
4	dbsnp	rs12506540	A	C	ALLELIC	3/19	0/50	7.115	1	0.007646
4	dbsnp	rs17008568	G	A	ALLELIC	3/19	0/50	7.115	1	0.007646
4	dbsnp	rs12648061	G	A	ALLELIC	3/19	0/50	7.115	1	0.007646
4	dbsnp	rs9986027	C	A	ALLELIC	3/19	0/50	7.115	1	0.007646
4	dbsnp	rs4147586	G	A	ALLELIC	3/19	0/50	7.115	1	0.007646
4	dbsnp	rs2602239	A	C	ALLELIC	3/19	0/50	7.115	1	0.007646
4	dbsnp	rs1782360	G	C	ALLELIC	3/19	0/50	7.115	1	0.007646
4	dbsnp	rs1201208	G	A	ALLELIC	3/19	0/50	7.115	1	0.007646
4	dbsnp	rs6839352	A	G	ALLELIC	3/19	0/50	7.115	1	0.007646
4	dbsnp	rs4696648	G	A	ALLELIC	3/19	0/50	7.115	1	0.007646
4	dbsnp	rs6819599	G	A	ALLELIC	3/19	0/50	7.115	1	0.007646
4	dbsnp	rs7697992	C	A	ALLELIC	3/19	0/50	7.115	1	0.007646
4	dbsnp	rs4696226	A	G	ALLELIC	3/19	0/50	7.115	1	0.007646
4	dbsnp	rs11734586	G	A	ALLELIC	3/19	0/50	7.115	1	0.007646
4	dbsnp	rs17030220	C	A	ALLELIC	3/19	0/50	7.115	1	0.007646
4	dbsnp	rs4431170	G	A	ALLELIC	3/19	0/50	7.115	1	0.007646
4	dbsnp	rs6826711	G	A	ALLELIC	3/19	0/50	7.115	1	0.007646
4	dbsnp	rs6819917	G	A	ALLELIC	3/19	0/50	7.115	1	0.007646
4	dbsnp	rs355196	A	G	ALLELIC	3/19	0/50	7.115	1	0.007646
4	dbsnp	rs10018611	G	A	ALLELIC	3/19	0/50	7.115	1	0.007646
5	dbsnp	rs13160629	G	A	ALLELIC	3/19	0/50	7.115	1	0.007646
5	dbsnp	rs10512742	A	C	ALLELIC	3/19	0/50	7.115	1	0.007646
5	dbsnp	rs10512833	A	G	ALLELIC	3/19	0/50	7.115	1	0.007646
5	dbsnp	rs6894626	A	G	ALLELIC	3/19	0/50	7.115	1	0.007646
5	dbsnp	rs16903801	A	G	ALLELIC	3/19	0/50	7.115	1	0.007646
5	dbsnp	rs1446037	A	G	ALLELIC	3/19	0/50	7.115	1	0.007646
5	dbsnp	rs16894704	A	C	ALLELIC	3/19	0/50	7.115	1	0.007646
5	dbsnp	rs7731629	G	A	ALLELIC	3/19	0/50	7.115	1	0.007646
5	dbsnp	rs2964209	G	A	ALLELIC	3/19	0/50	7.115	1	0.007646
5	dbsnp	rs2964173	A	G	ALLELIC	3/19	0/50	7.115	1	0.007646
5	dbsnp	rs2203822	G	A	ALLELIC	3/19	0/50	7.115	1	0.007646
5	dbsnp	rs870054	G	A	ALLELIC	3/19	0/50	7.115	1	0.007646
5	dbsnp	rs1862555	A	G	ALLELIC	3/19	0/50	7.115	1	0.007646
5	dbsnp	rs2290675	A	G	ALLELIC	3/19	0/50	7.115	1	0.007646
5	dbsnp	rs6557015	G	A	ALLELIC	3/19	0/50	7.115	1	0.007646
5	dbsnp	rs12521805	G	A	ALLELIC	3/19	0/50	7.115	1	0.007646
5	dbsnp	rs17150847	G	A	ALLELIC	3/19	0/50	7.115	1	0.007646
5	dbsnp	rs1048957	C	A	ALLELIC	3/19	0/50	7.115	1	0.007646
5	dbsnp	rs28994879	A	G	ALLELIC	3/19	0/50	7.115	1	0.007646
5	dbsnp	rs6897560	A	G	ALLELIC	3/19	0/50	7.115	1	0.007646

5	dbsnp	rs10074081	A	G	ALLELIC	3/19	0/50	7.115	1	0.007646
6	dbsnp	rs3823133	G	A	ALLELIC	3/19	0/50	7.115	1	0.007646
6	dbsnp	rs1747593	A	C	ALLELIC	3/19	0/50	7.115	1	0.007646
6	dbsnp	rs3863219	G	A	ALLELIC	3/19	0/50	7.115	1	0.007646
6	dbsnp	rs1737556	A	G	ALLELIC	3/19	0/50	7.115	1	0.007646
6	dbsnp	rs2294670	C	A	ALLELIC	3/19	0/50	7.115	1	0.007646
6	dbsnp	rs110284	G	A	ALLELIC	3/19	0/50	7.115	1	0.007646
6	dbsnp	rs17142699	G	A	ALLELIC	3/19	0/50	7.115	1	0.007646
6	dbsnp	rs9380830	G	A	ALLELIC	3/19	0/50	7.115	1	0.007646
6	dbsnp	rs16880565	C	A	ALLELIC	3/19	0/50	7.115	1	0.007646
6	dbsnp	rs16890551	A	G	ALLELIC	3/19	0/50	7.115	1	0.007646
6	dbsnp	rs6920787	C	A	ALLELIC	3/19	0/50	7.115	1	0.007646
6	dbsnp	rs28365995	A	G	ALLELIC	3/19	0/50	7.115	1	0.007646
6	dbsnp	rs17583915	G	A	ALLELIC	3/19	0/50	7.115	1	0.007646
6	dbsnp	rs17617515	A	C	ALLELIC	3/19	0/50	7.115	1	0.007646
6	dbsnp	rs10484568	G	A	ALLELIC	3/19	0/50	7.115	1	0.007646
6	dbsnp	rs3918143	A	G	ALLELIC	3/19	0/50	7.115	1	0.007646
6	dbsnp	rs17220500	C	G	ALLELIC	3/19	0/50	7.115	1	0.007646
6	dbsnp	rs2273063	A	G	ALLELIC	3/19	0/50	7.115	1	0.007646
6	dbsnp	rs16882561	A	G	ALLELIC	3/19	0/50	7.115	1	0.007646
6	dbsnp	rs1266787	G	A	ALLELIC	3/19	0/50	7.115	1	0.007646
6	dbsnp	rs2397091	G	A	ALLELIC	3/19	0/50	7.115	1	0.007646
6	dbsnp	rs3003495	A	C	ALLELIC	3/19	0/50	7.115	1	0.007646
6	dbsnp	rs16881749	A	G	ALLELIC	3/19	0/50	7.115	1	0.007646
6	dbsnp	rs2503718	A	G	ALLELIC	3/19	0/50	7.115	1	0.007646
6	dbsnp	rs17501289	A	G	ALLELIC	3/19	0/50	7.115	1	0.007646
6	dbsnp	rs7775945	G	A	ALLELIC	3/19	0/50	7.115	1	0.007646
6	dbsnp	rs195077	C	A	ALLELIC	3/19	0/50	7.115	1	0.007646
6	dbsnp	rs9372528	A	G	ALLELIC	3/19	0/50	7.115	1	0.007646
6	dbsnp	rs9398511	G	A	ALLELIC	3/19	0/50	7.115	1	0.007646
6	dbsnp	rs7768419	G	A	ALLELIC	3/19	0/50	7.115	1	0.007646
6	dbsnp	rs10457047	G	A	ALLELIC	3/19	0/50	7.115	1	0.007646
6	dbsnp	rs2297367	A	G	ALLELIC	3/19	0/50	7.115	1	0.007646
6	dbsnp	rs3798174	A	G	ALLELIC	3/19	0/50	7.115	1	0.007646
7	dbsnp	rs10265031	C	A	ALLELIC	3/19	0/50	7.115	1	0.007646
7	dbsnp	rs10085783	A	G	ALLELIC	3/19	0/50	7.115	1	0.007646
7	dbsnp	rs6977880	A	G	ALLELIC	3/19	0/50	7.115	1	0.007646
7	dbsnp	rs6979650	A	G	ALLELIC	3/19	0/50	7.115	1	0.007646
7	dbsnp	rs12670562	A	G	ALLELIC	3/19	0/50	7.115	1	0.007646
7	dbsnp	rs9649808	C	A	ALLELIC	3/19	0/50	7.115	1	0.007646
7	dbsnp	rs10228124	A	C	ALLELIC	3/19	0/50	7.115	1	0.007646
7	dbsnp	rs3751565	A	G	ALLELIC	3/19	0/50	7.115	1	0.007646
7	dbsnp	rs17840380	A	G	ALLELIC	3/19	0/50	7.115	1	0.007646
7	dbsnp	rs12534498	A	G	ALLELIC	3/19	0/50	7.115	1	0.007646
7	dbsnp	rs17163541	G	A	ALLELIC	3/19	0/50	7.115	1	0.007646
7	dbsnp	rs7807511	G	A	ALLELIC	3/19	0/50	7.115	1	0.007646
7	dbsnp	rs1115265	A	G	ALLELIC	3/19	0/50	7.115	1	0.007646
7	dbsnp	rs10248220	G	A	ALLELIC	3/19	0/50	7.115	1	0.007646

7	dbsnp	rs4148843	A	G	ALLELIC	3/19	0/50	7.115	1	0.007646
7	dbsnp	rs7787974	G	A	ALLELIC	3/19	0/50	7.115	1	0.007646
7	dbsnp	rs842446	G	A	ALLELIC	3/19	0/50	7.115	1	0.007646
8	dbsnp	rs11777803	G	A	ALLELIC	3/19	0/50	7.115	1	0.007646
8	dbsnp	rs2617097	A	G	ALLELIC	3/19	0/50	7.115	1	0.007646
8	dbsnp	rs12682166	A	G	ALLELIC	3/19	0/50	7.115	1	0.007646
8	dbsnp	rs10108954	A	G	ALLELIC	3/19	0/50	7.115	1	0.007646
8	dbsnp	rs4841435	A	G	ALLELIC	3/19	0/50	7.115	1	0.007646
8	dbsnp	rs17632013	A	G	ALLELIC	3/19	0/50	7.115	1	0.007646
8	dbsnp	rs17054639	G	A	ALLELIC	3/19	0/50	7.115	1	0.007646
8	dbsnp	rs17056112	A	G	ALLELIC	3/19	0/50	7.115	1	0.007646
8	dbsnp	rs4272427	A	C	ALLELIC	3/19	0/50	7.115	1	0.007646
8	dbsnp	rs7815490	A	G	ALLELIC	3/19	0/50	7.115	1	0.007646
8	dbsnp	rs11136098	A	G	ALLELIC	3/19	0/50	7.115	1	0.007646
8	dbsnp	rs4735757	A	C	ALLELIC	3/19	0/50	7.115	1	0.007646
8	dbsnp	rs3018862	G	A	ALLELIC	3/19	0/50	7.115	1	0.007646
8	dbsnp	rs542253	A	G	ALLELIC	3/19	0/50	7.115	1	0.007646
8	dbsnp	rs546474	G	A	ALLELIC	3/19	0/50	7.115	1	0.007646
8	dbsnp	rs10101743	C	A	ALLELIC	3/19	0/50	7.115	1	0.007646
8	dbsnp	rs3897874	A	G	ALLELIC	3/19	0/50	7.115	1	0.007646
8	dbsnp	rs16886401	A	G	ALLELIC	3/19	0/50	7.115	1	0.007646
8	dbsnp	rs16901432	G	A	ALLELIC	3/19	0/50	7.115	1	0.007646
8	dbsnp	rs10505503	A	G	ALLELIC	3/19	0/50	7.115	1	0.007646
8	dbsnp	rs7816583	G	A	ALLELIC	3/19	0/50	7.115	1	0.007646
8	dbsnp	rs12541799	A	G	ALLELIC	3/19	0/50	7.115	1	0.007646
8	dbsnp	rs16906787	A	G	ALLELIC	3/19	0/50	7.115	1	0.007646
8	dbsnp	rs10282908	A	G	ALLELIC	3/19	0/50	7.115	1	0.007646
9	dbsnp	rs12005362	A	G	ALLELIC	3/19	0/50	7.115	1	0.007646
9	dbsnp	rs7868180	G	A	ALLELIC	3/19	0/50	7.115	1	0.007646
9	dbsnp	rs34307867	A	G	ALLELIC	3/19	0/50	7.115	1	0.007646
9	dbsnp	rs7873050	C	A	ALLELIC	3/19	0/50	7.115	1	0.007646
9	dbsnp	rs7868480	A	C	ALLELIC	3/19	0/50	7.115	1	0.007646
9	dbsnp	rs7040199	G	A	ALLELIC	3/19	0/50	7.115	1	0.007646
9	dbsnp	rs7864652	A	G	ALLELIC	3/19	0/50	7.115	1	0.007646
9	dbsnp	rs12376522	A	G	ALLELIC	3/19	0/50	7.115	1	0.007646
9	dbsnp	rs994975	A	C	ALLELIC	3/19	0/50	7.115	1	0.007646
9	dbsnp	rs10967194	G	A	ALLELIC	3/19	0/50	7.115	1	0.007646
9	dbsnp	rs16910176	G	A	ALLELIC	3/19	0/50	7.115	1	0.007646
9	dbsnp	rs7035912	A	G	ALLELIC	3/19	0/50	7.115	1	0.007646
9	dbsnp	rs1836463	A	G	ALLELIC	3/19	0/50	7.115	1	0.007646
9	dbsnp	rs2571519	A	G	ALLELIC	3/19	0/50	7.115	1	0.007646
9	dbsnp	rs12553273	G	A	ALLELIC	3/19	0/50	7.115	1	0.007646
9	dbsnp	rs2297343	A	G	ALLELIC	3/19	0/50	7.115	1	0.007646
9	dbsnp	rs7023719	G	A	ALLELIC	3/19	0/50	7.115	1	0.007646
9	dbsnp	rs12238437	A	G	ALLELIC	3/19	0/50	7.115	1	0.007646
9	dbsnp	rs4838118	A	C	ALLELIC	3/19	0/50	7.115	1	0.007646
9	dbsnp	rs7860461	G	A	ALLELIC	3/19	0/50	7.115	1	0.007646
9	dbsnp	rs4836535	A	C	ALLELIC	3/19	0/50	7.115	1	0.007646

9	dbsnp	rs7025596	G	A	ALLELIC	3/19	0/50	7.115	1	0.007646
9	dbsnp	rs28488438	A	G	ALLELIC	3/19	0/50	7.115	1	0.007646
9	dbsnp	rs7390551	A	G	ALLELIC	3/19	0/50	7.115	1	0.007646
9	dbsnp	rs3125782	A	G	ALLELIC	3/19	0/50	7.115	1	0.007646
10	dbsnp	rs11251367	A	G	ALLELIC	3/19	0/50	7.115	1	0.007646
10	dbsnp	rs10751917	A	G	ALLELIC	3/19	0/50	7.115	1	0.007646
10	dbsnp	rs2765654	A	G	ALLELIC	3/19	0/50	7.115	1	0.007646
10	dbsnp	rs7079031	A	G	ALLELIC	3/19	0/50	7.115	1	0.007646
10	dbsnp	rs612766	C	A	ALLELIC	3/19	0/50	7.115	1	0.007646
10	dbsnp	rs641297	A	G	ALLELIC	3/19	0/50	7.115	1	0.007646
10	dbsnp	rs4747019	A	G	ALLELIC	3/19	0/50	7.115	1	0.007646
10	dbsnp	rs10999435	A	G	ALLELIC	3/19	0/50	7.115	1	0.007646
10	dbsnp	rs7909650	A	G	ALLELIC	3/19	0/50	7.115	1	0.007646
10	dbsnp	rs17111253	A	G	ALLELIC	3/19	0/50	7.115	1	0.007646
10	dbsnp	rs17112654	G	A	ALLELIC	3/19	0/50	7.115	1	0.007646
10	dbsnp	rs4919428	C	A	ALLELIC	3/19	0/50	7.115	1	0.007646
10	dbsnp	rs12262005	A	C	ALLELIC	3/19	0/50	7.115	1	0.007646
10	dbsnp	rs12269345	A	G	ALLELIC	3/19	0/50	7.115	1	0.007646
10	dbsnp	rs3750710	G	A	ALLELIC	3/19	0/50	7.115	1	0.007646
10	dbsnp	rs4917452	A	G	ALLELIC	3/19	0/50	7.115	1	0.007646
10	dbsnp	rs12217301	A	C	ALLELIC	3/19	0/50	7.115	1	0.007646
10	dbsnp	rs11813708	A	C	ALLELIC	3/19	0/50	7.115	1	0.007646
10	dbsnp	rs2185261	A	C	ALLELIC	3/19	0/50	7.115	1	0.007646
11	dbsnp	rs11608118	A	G	ALLELIC	3/19	0/50	7.115	1	0.007646
11	dbsnp	rs1397591	A	C	ALLELIC	3/19	0/50	7.115	1	0.007646
11	dbsnp	rs7481121	A	G	ALLELIC	3/19	0/50	7.115	1	0.007646
11	dbsnp	rs4758291	C	A	ALLELIC	3/19	0/50	7.115	1	0.007646
11	dbsnp	rs10769880	G	A	ALLELIC	3/19	0/50	7.115	1	0.007646
11	dbsnp	rs3741043	A	G	ALLELIC	3/19	0/50	7.115	1	0.007646
11	dbsnp	rs7948666	A	G	ALLELIC	3/19	0/50	7.115	1	0.007646
11	dbsnp	rs16908075	G	A	ALLELIC	3/19	0/50	7.115	1	0.007646
11	dbsnp	rs12418180	A	G	ALLELIC	3/19	0/50	7.115	1	0.007646
11	dbsnp	rs831464	A	C	ALLELIC	3/19	0/50	7.115	1	0.007646
11	dbsnp	rs11030406	A	G	ALLELIC	3/19	0/50	7.115	1	0.007646
11	dbsnp	rs1374494	G	A	ALLELIC	3/19	0/50	7.115	1	0.007646
11	dbsnp	rs11608110	A	C	ALLELIC	3/19	0/50	7.115	1	0.007646
11	dbsnp	rs11605263	A	G	ALLELIC	3/19	0/50	7.115	1	0.007646
11	dbsnp	rs4980673	G	A	ALLELIC	3/19	0/50	7.115	1	0.007646
11	dbsnp	rs17136727	A	G	ALLELIC	3/19	0/50	7.115	1	0.007646
11	dbsnp	rs341093	C	A	ALLELIC	3/19	0/50	7.115	1	0.007646
11	dbsnp	rs11826354	A	C	ALLELIC	3/19	0/50	7.115	1	0.007646
11	dbsnp	rs2010606	A	C	ALLELIC	3/19	0/50	7.115	1	0.007646
11	dbsnp	rs12419815	A	G	ALLELIC	3/19	0/50	7.115	1	0.007646
11	dbsnp	rs6589574	A	G	ALLELIC	3/19	0/50	7.115	1	0.007646
11	dbsnp	rs2115925	G	A	ALLELIC	3/19	0/50	7.115	1	0.007646
11	dbsnp	rs3802807	A	G	ALLELIC	3/19	0/50	7.115	1	0.007646
11	dbsnp	rs7936592	G	A	ALLELIC	3/19	0/50	7.115	1	0.007646
11	dbsnp	rs34780562	A	G	ALLELIC	3/19	0/50	7.115	1	0.007646

11	dbsnp	rs7112898	A	G	ALLELIC	3/19	0/50	7.115	1	0.007646
12	dbsnp	rs2058032	A	G	ALLELIC	3/19	0/50	7.115	1	0.007646
12	dbsnp	rs12820601	A	G	ALLELIC	3/19	0/50	7.115	1	0.007646
12	dbsnp	rs6582600	A	C	ALLELIC	3/19	0/50	7.115	1	0.007646
12	dbsnp	rs11183045	A	C	ALLELIC	3/19	0/50	7.115	1	0.007646
12	dbsnp	rs11168983	A	G	ALLELIC	3/19	0/50	7.115	1	0.007646
12	dbsnp	rs773651	A	G	ALLELIC	3/19	0/50	7.115	1	0.007646
12	dbsnp	rs2122694	A	G	ALLELIC	3/19	0/50	7.115	1	0.007646
12	dbsnp	rs11176712	A	G	ALLELIC	3/19	0/50	7.115	1	0.007646
12	dbsnp	rs17111115	A	G	ALLELIC	3/19	0/50	7.115	1	0.007646
12	dbsnp	rs10735984	G	A	ALLELIC	3/19	0/50	7.115	1	0.007646
12	dbsnp	rs17005500	G	A	ALLELIC	3/19	0/50	7.115	1	0.007646
12	dbsnp	rs10735321	A	G	ALLELIC	3/19	0/50	7.115	1	0.007646
12	dbsnp	rs5742612	G	A	ALLELIC	3/19	0/50	7.115	1	0.007646
12	dbsnp	rs6539151	G	A	ALLELIC	3/19	0/50	7.115	1	0.007646
12	dbsnp	rs17036205	A	G	ALLELIC	3/19	0/50	7.115	1	0.007646
12	dbsnp	rs4766793	A	G	ALLELIC	3/19	0/50	7.115	1	0.007646
12	dbsnp	rs4765424	A	G	ALLELIC	3/19	0/50	7.115	1	0.007646
13	dbsnp	rs9552958	A	G	ALLELIC	3/19	0/50	7.115	1	0.007646
13	dbsnp	rs7985561	A	G	ALLELIC	3/19	0/50	7.115	1	0.007646
13	dbsnp	rs7333424	A	G	ALLELIC	3/19	0/50	7.115	1	0.007646
13	dbsnp	rs12853449	A	G	ALLELIC	3/19	0/50	7.115	1	0.007646
13	dbsnp	rs7986530	A	G	ALLELIC	3/19	0/50	7.115	1	0.007646
13	dbsnp	rs12427503	A	G	ALLELIC	3/19	0/50	7.115	1	0.007646
13	dbsnp	rs12430612	G	A	ALLELIC	3/19	0/50	7.115	1	0.007646
13	dbsnp	rs9542299	G	A	ALLELIC	3/19	0/50	7.115	1	0.007646
13	dbsnp	rs2225376	A	G	ALLELIC	3/19	0/50	7.115	1	0.007646
13	dbsnp	rs7998158	G	A	ALLELIC	3/19	0/50	7.115	1	0.007646
13	dbsnp	rs2031387	G	A	ALLELIC	3/19	0/50	7.115	1	0.007646
13	dbsnp	rs9559025	A	G	ALLELIC	3/19	0/50	7.115	1	0.007646
13	dbsnp	rs16972960	A	G	ALLELIC	3/19	0/50	7.115	1	0.007646
14	dbsnp	rs3748366	G	A	ALLELIC	3/19	0/50	7.115	1	0.007646
14	dbsnp	rs12434054	G	A	ALLELIC	3/19	0/50	7.115	1	0.007646
14	dbsnp	rs848049	A	G	ALLELIC	3/19	0/50	7.115	1	0.007646
14	dbsnp	rs7156095	C	A	ALLELIC	3/19	0/50	7.115	1	0.007646
14	dbsnp	rs10483829	G	A	ALLELIC	3/19	0/50	7.115	1	0.007646
14	dbsnp	rs17108204	A	G	ALLELIC	3/19	0/50	7.115	1	0.007646
14	dbsnp	rs7157907	G	A	ALLELIC	3/19	0/50	7.115	1	0.007646
14	dbsnp	rs17117682	A	G	ALLELIC	3/19	0/50	7.115	1	0.007646
14	dbsnp	rs229833	G	A	ALLELIC	3/19	0/50	7.115	1	0.007646
14	dbsnp	rs1885750	A	G	ALLELIC	3/19	0/50	7.115	1	0.007646
14	dbsnp	rs7148434	G	A	ALLELIC	3/19	0/50	7.115	1	0.007646
14	dbsnp	rs10136276	A	G	ALLELIC	3/19	0/50	7.115	1	0.007646
14	dbsnp	rs10148231	G	A	ALLELIC	3/19	0/50	7.115	1	0.007646
14	dbsnp	rs10134473	A	G	ALLELIC	3/19	0/50	7.115	1	0.007646
14	dbsnp	rs11847931	G	A	ALLELIC	3/19	0/50	7.115	1	0.007646
15	dbsnp	rs2873026	G	A	ALLELIC	3/19	0/50	7.115	1	0.007646
15	dbsnp	rs1399079	A	G	ALLELIC	3/19	0/50	7.115	1	0.007646

15	dbsnp	rs4780141	A	G	ALLELIC	3/19	0/50	7.115	1	0.007646
15	dbsnp	rs4780144	G	A	ALLELIC	3/19	0/50	7.115	1	0.007646
15	dbsnp	rs658752	A	G	ALLELIC	3/19	0/50	7.115	1	0.007646
15	dbsnp	rs690136	A	G	ALLELIC	3/19	0/50	7.115	1	0.007646
15	dbsnp	rs690365	A	G	ALLELIC	3/19	0/50	7.115	1	0.007646
15	dbsnp	rs675262	G	A	ALLELIC	3/19	0/50	7.115	1	0.007646
15	dbsnp	rs676180	A	G	ALLELIC	3/19	0/50	7.115	1	0.007646
15	dbsnp	rs677273	G	A	ALLELIC	3/19	0/50	7.115	1	0.007646
15	dbsnp	rs694213	G	A	ALLELIC	3/19	0/50	7.115	1	0.007646
15	dbsnp	rs16971186	G	A	ALLELIC	3/19	0/50	7.115	1	0.007646
15	dbsnp	rs686038	G	A	ALLELIC	3/19	0/50	7.115	1	0.007646
15	dbsnp	rs491274	G	A	ALLELIC	3/19	0/50	7.115	1	0.007646
15	dbsnp	rs8029656	G	A	ALLELIC	3/19	0/50	7.115	1	0.007646
15	dbsnp	rs12148122	A	C	ALLELIC	3/19	0/50	7.115	1	0.007646
15	dbsnp	rs3743205	A	G	ALLELIC	3/19	0/50	7.115	1	0.007646
15	dbsnp	rs2930313	G	A	ALLELIC	3/19	0/50	7.115	1	0.007646
15	dbsnp	rs17552981	G	A	ALLELIC	3/19	0/50	7.115	1	0.007646
16	dbsnp	rs2939960	A	G	ALLELIC	3/19	0/50	7.115	1	0.007646
16	dbsnp	rs17139716	A	G	ALLELIC	3/19	0/50	7.115	1	0.007646
16	dbsnp	rs1159167	A	C	ALLELIC	3/19	0/50	7.115	1	0.007646
16	dbsnp	rs1475968	A	G	ALLELIC	3/19	0/50	7.115	1	0.007646
16	dbsnp	rs2941256	G	A	ALLELIC	3/19	0/50	7.115	1	0.007646
16	dbsnp	rs11860071	A	G	ALLELIC	3/19	0/50	7.115	1	0.007646
17	dbsnp	rs8074959	A	G	ALLELIC	3/19	0/50	7.115	1	0.007646
17	dbsnp	rs16954373	A	C	ALLELIC	3/19	0/50	7.115	1	0.007646
17	dbsnp	rs11571365	A	G	ALLELIC	3/19	0/50	7.115	1	0.007646
17	dbsnp	rs3182380	A	G	ALLELIC	3/19	0/50	7.115	1	0.007646
17	dbsnp	rs4795430	A	G	ALLELIC	3/19	0/50	7.115	1	0.007646
17	dbsnp	rs7220074	G	A	ALLELIC	3/19	0/50	7.115	1	0.007646
17	dbsnp	rs12603921	G	A	ALLELIC	3/19	0/50	7.115	1	0.007646
17	dbsnp	rs12451779	A	G	ALLELIC	3/19	0/50	7.115	1	0.007646
17	dbsnp	rs16960228	A	G	ALLELIC	3/19	0/50	7.115	1	0.007646
17	dbsnp	rs1468472	A	G	ALLELIC	3/19	0/50	7.115	1	0.007646
17	dbsnp	rs8076407	G	A	ALLELIC	3/19	0/50	7.115	1	0.007646
17	dbsnp	rs4789937	A	G	ALLELIC	3/19	0/50	7.115	1	0.007646
17	dbsnp	rs12601947	A	G	ALLELIC	3/19	0/50	7.115	1	0.007646
18	dbsnp	rs7244607	A	G	ALLELIC	3/19	0/50	7.115	1	0.007646
18	dbsnp	rs9947868	A	C	ALLELIC	3/19	0/50	7.115	1	0.007646
18	dbsnp	rs12956849	A	C	ALLELIC	3/19	0/50	7.115	1	0.007646
18	dbsnp	rs2011391	A	G	ALLELIC	3/19	0/50	7.115	1	0.007646
18	dbsnp	rs273730	G	A	ALLELIC	3/19	0/50	7.115	1	0.007646
18	dbsnp	rs9947145	A	G	ALLELIC	3/19	0/50	7.115	1	0.007646
18	dbsnp	rs9963266	A	G	ALLELIC	3/19	0/50	7.115	1	0.007646
18	dbsnp	rs7244666	C	A	ALLELIC	3/19	0/50	7.115	1	0.007646
18	dbsnp	rs4239380	G	A	ALLELIC	3/19	0/50	7.115	1	0.007646
18	dbsnp	rs8083143	G	A	ALLELIC	3/19	0/50	7.115	1	0.007646
18	dbsnp	rs1348336	C	A	ALLELIC	3/19	0/50	7.115	1	0.007646
18	dbsnp	rs17744022	A	C	ALLELIC	3/19	0/50	7.115	1	0.007646

18	dbsnp	rs12954409	A	G	ALLELIC	3/19	0/50	7.115	1	0.007646
18	dbsnp	rs12709705	A	G	ALLELIC	3/19	0/50	7.115	1	0.007646
18	dbsnp	rs7233521	A	G	ALLELIC	3/19	0/50	7.115	1	0.007646
18	dbsnp	rs16951370	G	A	ALLELIC	3/19	0/50	7.115	1	0.007646
18	dbsnp	rs17064003	A	G	ALLELIC	3/19	0/50	7.115	1	0.007646
18	dbsnp	rs12965811	A	C	ALLELIC	3/19	0/50	7.115	1	0.007646
18	dbsnp	rs9952419	A	G	ALLELIC	3/19	0/50	7.115	1	0.007646
19	dbsnp	rs10417988	A	G	ALLELIC	3/19	0/50	7.115	1	0.007646
19	dbsnp	rs10518260	A	G	ALLELIC	3/19	0/50	7.115	1	0.007646
19	dbsnp	rs16967815	A	G	ALLELIC	3/19	0/50	7.115	1	0.007646
19	dbsnp	rs8109447	G	A	ALLELIC	3/19	0/50	7.115	1	0.007646
19	dbsnp	rs7255180	A	G	ALLELIC	3/19	0/50	7.115	1	0.007646
19	dbsnp	rs1049339	A	G	ALLELIC	3/19	0/50	7.115	1	0.007646
19	dbsnp	rs11575002	C	A	ALLELIC	3/19	0/50	7.115	1	0.007646
19	dbsnp	rs7254951	A	G	ALLELIC	3/19	0/50	7.115	1	0.007646
19	dbsnp	rs16988665	G	A	ALLELIC	3/19	0/50	7.115	1	0.007646
19	dbsnp	rs1268538	A	G	ALLELIC	3/19	0/50	7.115	1	0.007646
19	dbsnp	rs893184	A	G	ALLELIC	3/19	0/50	7.115	1	0.007646
20	dbsnp	rs1739573	G	A	ALLELIC	3/19	0/50	7.115	1	0.007646
20	dbsnp	rs6081676	G	A	ALLELIC	3/19	0/50	7.115	1	0.007646
20	dbsnp	rs4589832	A	C	ALLELIC	3/19	0/50	7.115	1	0.007646
20	dbsnp	rs6129888	A	G	ALLELIC	3/19	0/50	7.115	1	0.007646
20	dbsnp	rs6103385	A	G	ALLELIC	3/19	0/50	7.115	1	0.007646
20	dbsnp	rs292116	A	G	ALLELIC	3/19	0/50	7.115	1	0.007646
20	dbsnp	rs7262424	G	A	ALLELIC	3/19	0/50	7.115	1	0.007646
20	dbsnp	rs6098415	G	A	ALLELIC	3/19	0/50	7.115	1	0.007646
20	dbsnp	rs6023860	A	G	ALLELIC	3/19	0/50	7.115	1	0.007646
21	dbsnp	rs16985745	A	G	ALLELIC	3/19	0/50	7.115	1	0.007646
21	dbsnp	rs2836421	A	G	ALLELIC	3/19	0/50	7.115	1	0.007646
22	dbsnp	rs12157958	C	A	ALLELIC	3/19	0/50	7.115	1	0.007646
22	dbsnp	rs375115	A	C	ALLELIC	3/19	0/50	7.115	1	0.007646
22	dbsnp	rs17821572	G	A	ALLELIC	3/19	0/50	7.115	1	0.007646
22	dbsnp	rs5996898	A	G	ALLELIC	3/19	0/50	7.115	1	0.007646
22	dbsnp	rs7288872	A	G	ALLELIC	3/19	0/50	7.115	1	0.007646
22	dbsnp	rs8139746	G	A	ALLELIC	3/19	0/50	7.115	1	0.007646
22	dbsnp	rs4822622	A	G	ALLELIC	3/19	0/50	7.115	1	0.007646
22	dbsnp	rs8136102	G	A	ALLELIC	3/19	0/50	7.115	1	0.007646
22	dbsnp	rs7292706	A	G	ALLELIC	3/19	0/50	7.115	1	0.007646
22	dbsnp	rs12167259	A	C	ALLELIC	3/19	0/50	7.115	1	0.007646
22	dbsnp	rs12167472	A	G	ALLELIC	3/19	0/50	7.115	1	0.007646
22	dbsnp	rs3788560	A	G	ALLELIC	3/19	0/50	7.115	1	0.007646
22	dbsnp	rs6009718	G	A	ALLELIC	3/19	0/50	7.115	1	0.007646
22	dbsnp	rs12159805	A	G	ALLELIC	3/19	0/50	7.115	1	0.007646
22	dbsnp	rs28372448	A	G	ALLELIC	3/19	0/50	7.115	1	0.007646
23	dbsnp	rs6530179	A	G	ALLELIC	3/19	0/50	7.115	1	0.007646
23	dbsnp	rs11796001	C	A	ALLELIC	3/19	0/50	7.115	1	0.007646
23	dbsnp	rs7056599	G	A	ALLELIC	3/19	0/50	7.115	1	0.007646
23	dbsnp	rs5926809	A	G	ALLELIC	3/19	0/50	7.115	1	0.007646

23	dbsnp	rs17474852	G	A	ALLELIC	3/19	0/50	7.115	1	0.007646
23	dbsnp	rs17145643	G	A	ALLELIC	3/19	0/50	7.115	1	0.007646
23	dbsnp	rs717689	A	G	ALLELIC	3/19	0/50	7.115	1	0.007646
23	dbsnp	rs4363320	G	A	ALLELIC	3/19	0/50	7.115	1	0.007646
23	dbsnp	rs17313135	A	G	ALLELIC	3/19	0/50	7.115	1	0.007646
23	dbsnp	rs7876455	A	G	ALLELIC	3/19	0/50	7.115	1	0.007646
23	dbsnp	rs7064462	G	A	ALLELIC	3/19	0/50	7.115	1	0.007646
23	dbsnp	rs1323577	C	A	ALLELIC	3/19	0/50	7.115	1	0.007646
23	dbsnp	rs5945974	G	A	ALLELIC	3/19	0/50	7.115	1	0.007646
23	dbsnp	rs17329546	A	C	ALLELIC	3/19	0/50	7.115	1	0.007646
23	dbsnp	rs12689389	A	G	ALLELIC	3/19	0/50	7.115	1	0.007646
23	dbsnp	rs16993397	G	A	ALLELIC	3/19	0/50	7.115	1	0.007646
23	dbsnp	rs392959	A	G	ALLELIC	3/19	0/50	7.115	1	0.007646
23	dbsnp	rs4149759	G	A	ALLELIC	3/19	0/50	7.115	1	0.007646
23	dbsnp	rs10521841	A	C	ALLELIC	3/19	0/50	7.115	1	0.007646
23	dbsnp	rs6649728	A	G	ALLELIC	3/19	0/50	7.115	1	0.007646
23	dbsnp	rs6649722	A	G	ALLELIC	3/19	0/50	7.115	1	0.007646
23	dbsnp	rs1339599	A	C	ALLELIC	3/19	0/50	7.115	1	0.007646
23	dbsnp	rs12011741	G	A	ALLELIC	3/19	0/50	7.115	1	0.007646
23	dbsnp	rs10449049	A	G	ALLELIC	3/19	0/50	7.115	1	0.007646
23	dbsnp	rs6608304	G	A	ALLELIC	3/19	0/50	7.115	1	0.007646
23	dbsnp	rs6413703	A	G	ALLELIC	3/19	0/50	7.115	1	0.007646
23	dbsnp	rs3895379	A	G	ALLELIC	3/19	0/50	7.115	1	0.007646
2	dbsnp	rs1822770	C	A	ALLELIC	9/7	8/32	7.103	1	0.007693
14	dbsnp	rs2803947	A	G	ALLELIC	4/18	24/22	7.099	1	0.007711
3	dbsnp	rs10849	G	A	ALLELIC	15/5	19/29	7.083	1	0.00778
3	dbsnp	rs11129756	A	G	ALLELIC	5/15	29/19	7.083	1	0.00778
20	dbsnp	rs709045	A	G	ALLELIC	15/5	19/29	7.083	1	0.00778
1	dbsnp	rs4648432	C	A	ALLELIC	10/12	8/42	7.069	1	0.007843
1	dbsnp	rs2045332	A	G	ALLELIC	10/12	8/42	7.069	1	0.007843
1	dbsnp	rs1280971	A	G	ALLELIC	10/12	8/42	7.069	1	0.007843
1	dbsnp	rs12565650	A	G	ALLELIC	1/21	17/33	7.069	1	0.007843
1	dbsnp	rs1288627	A	G	ALLELIC	10/12	8/42	7.069	1	0.007843
1	dbsnp	rs943521	A	C	ALLELIC	10/12	8/42	7.069	1	0.007843
1	dbsnp	rs1288637	G	A	ALLELIC	10/12	8/42	7.069	1	0.007843
1	dbsnp	rs1747924	C	A	ALLELIC	10/12	8/42	7.069	1	0.007843
1	dbsnp	rs4606347	A	G	ALLELIC	10/12	8/42	7.069	1	0.007843
1	dbsnp	rs8179183	C	G	ALLELIC	10/12	8/42	7.069	1	0.007843
1	dbsnp	rs17415296	A	C	ALLELIC	10/12	8/42	7.069	1	0.007843
1	dbsnp	rs11807752	G	A	ALLELIC	10/12	8/42	7.069	1	0.007843
1	dbsnp	rs17407594	A	G	ALLELIC	10/12	8/42	7.069	1	0.007843
1	dbsnp	rs4085003	C	A	ALLELIC	10/12	8/42	7.069	1	0.007843
1	dbsnp	rs6694924	A	G	ALLELIC	1/21	17/33	7.069	1	0.007843
1	dbsnp	rs4147591	A	C	ALLELIC	1/21	17/33	7.069	1	0.007843
1	dbsnp	rs4147595	G	C	ALLELIC	1/21	17/33	7.069	1	0.007843
1	dbsnp	rs3753931	A	G	ALLELIC	10/12	8/42	7.069	1	0.007843
1	dbsnp	rs16861715	G	A	ALLELIC	10/12	8/42	7.069	1	0.007843
1	dbsnp	rs10911592	C	A	ALLELIC	10/12	8/42	7.069	1	0.007843

1	dbsnp	rs10158710	C	A	ALLELIC	10/12	8/42	7.069	1	0.007843
1	dbsnp	rs4449992	G	A	ALLELIC	10/12	8/42	7.069	1	0.007843
1	dbsnp	rs1338302	A	G	ALLELIC	10/12	8/42	7.069	1	0.007843
1	dbsnp	rs3820119	A	G	ALLELIC	10/12	8/42	7.069	1	0.007843
1	dbsnp	rs4658673	A	G	ALLELIC	1/21	17/33	7.069	1	0.007843
1	dbsnp	rs10927345	G	A	ALLELIC	1/21	17/33	7.069	1	0.007843
2	dbsnp	rs730632	A	G	ALLELIC	10/12	8/42	7.069	1	0.007843
2	dbsnp	rs11686640	G	A	ALLELIC	10/12	8/42	7.069	1	0.007843
2	dbsnp	rs6542761	A	G	ALLELIC	10/12	8/42	7.069	1	0.007843
2	dbsnp	rs2198465	G	A	ALLELIC	10/12	8/42	7.069	1	0.007843
2	dbsnp	rs6745104	A	C	ALLELIC	10/12	8/42	7.069	1	0.007843
2	dbsnp	rs13432931	A	G	ALLELIC	1/21	17/33	7.069	1	0.007843
2	dbsnp	rs155128	G	A	ALLELIC	1/21	17/33	7.069	1	0.007843
2	dbsnp	rs826185	G	A	ALLELIC	10/12	8/42	7.069	1	0.007843
2	dbsnp	rs984773	G	A	ALLELIC	10/12	8/42	7.069	1	0.007843
2	dbsnp	rs6734283	A	G	ALLELIC	10/12	8/42	7.069	1	0.007843
3	dbsnp	rs11915246	G	A	ALLELIC	10/12	8/42	7.069	1	0.007843
3	dbsnp	rs9880404	A	G	ALLELIC	10/12	8/42	7.069	1	0.007843
3	dbsnp	rs13080281	G	A	ALLELIC	10/12	8/42	7.069	1	0.007843
3	dbsnp	rs2087919	A	C	ALLELIC	10/12	8/42	7.069	1	0.007843
3	dbsnp	rs2164356	C	A	ALLELIC	10/12	8/42	7.069	1	0.007843
3	dbsnp	rs3922891	G	A	ALLELIC	10/12	8/42	7.069	1	0.007843
3	dbsnp	rs2733393	A	G	ALLELIC	1/21	17/33	7.069	1	0.007843
3	dbsnp	rs2718430	A	G	ALLELIC	1/21	17/33	7.069	1	0.007843
3	dbsnp	rs1447699	A	G	ALLELIC	10/12	8/42	7.069	1	0.007843
3	dbsnp	rs1447698	A	G	ALLELIC	10/12	8/42	7.069	1	0.007843
3	dbsnp	rs9866988	A	G	ALLELIC	10/12	8/42	7.069	1	0.007843
3	dbsnp	rs1980275	A	G	ALLELIC	10/12	8/42	7.069	1	0.007843
3	dbsnp	rs7618742	A	G	ALLELIC	1/21	17/33	7.069	1	0.007843
4	dbsnp	rs7684204	A	G	ALLELIC	1/21	17/33	7.069	1	0.007843
4	dbsnp	rs1078758	A	G	ALLELIC	1/21	17/33	7.069	1	0.007843
4	dbsnp	rs10938932	A	G	ALLELIC	10/12	8/42	7.069	1	0.007843
4	dbsnp	rs16873417	A	G	ALLELIC	10/12	8/42	7.069	1	0.007843
4	dbsnp	rs12503311	A	G	ALLELIC	10/12	8/42	7.069	1	0.007843
4	dbsnp	rs758951	A	C	ALLELIC	1/21	17/33	7.069	1	0.007843
4	dbsnp	rs7678075	G	A	ALLELIC	10/12	8/42	7.069	1	0.007843
4	dbsnp	rs716693	G	A	ALLELIC	10/12	8/42	7.069	1	0.007843
4	dbsnp	rs12640320	A	C	ALLELIC	10/12	8/42	7.069	1	0.007843
4	dbsnp	rs4234848	G	A	ALLELIC	10/12	8/42	7.069	1	0.007843
4	dbsnp	rs2901383	G	A	ALLELIC	10/12	8/42	7.069	1	0.007843
4	dbsnp	rs13140099	A	G	ALLELIC	10/12	8/42	7.069	1	0.007843
5	dbsnp	rs10512644	C	A	ALLELIC	10/12	8/42	7.069	1	0.007843
5	dbsnp	rs2561621	A	G	ALLELIC	1/21	17/33	7.069	1	0.007843
5	dbsnp	rs1904165	A	C	ALLELIC	1/21	17/33	7.069	1	0.007843
5	dbsnp	rs2406371	G	A	ALLELIC	10/12	8/42	7.069	1	0.007843
5	dbsnp	rs3886214	A	G	ALLELIC	10/12	8/42	7.069	1	0.007843
5	dbsnp	rs252668	C	A	ALLELIC	1/21	17/33	7.069	1	0.007843
5	dbsnp	rs6884132	A	G	ALLELIC	1/21	17/33	7.069	1	0.007843

5	dbsnp	rs10044900	G	A	ALLELIC	1/21	17/33	7.069	1	0.007843
5	dbsnp	rs10042643	A	G	ALLELIC	1/21	17/33	7.069	1	0.007843
5	dbsnp	rs6860785	A	G	ALLELIC	1/21	17/33	7.069	1	0.007843
6	dbsnp	rs12206701	A	G	ALLELIC	10/12	8/42	7.069	1	0.007843
6	dbsnp	rs10946475	G	A	ALLELIC	1/21	17/33	7.069	1	0.007843
6	dbsnp	rs12213912	A	G	ALLELIC	10/12	8/42	7.069	1	0.007843
6	dbsnp	rs9382460	A	C	ALLELIC	10/12	8/42	7.069	1	0.007843
6	dbsnp	rs969970	A	G	ALLELIC	10/12	8/42	7.069	1	0.007843
6	dbsnp	rs6940845	A	G	ALLELIC	1/21	17/33	7.069	1	0.007843
6	dbsnp	rs9383820	A	G	ALLELIC	1/21	17/33	7.069	1	0.007843
7	dbsnp	rs952042	G	A	ALLELIC	10/12	8/42	7.069	1	0.007843
7	dbsnp	rs11762346	A	G	ALLELIC	10/12	8/42	7.069	1	0.007843
7	dbsnp	rs17687519	A	G	ALLELIC	10/12	8/42	7.069	1	0.007843
7	dbsnp	rs7804217	A	G	ALLELIC	1/21	17/33	7.069	1	0.007843
7	dbsnp	rs2053338	G	A	ALLELIC	1/21	17/33	7.069	1	0.007843
7	dbsnp	rs1347879	A	G	ALLELIC	1/21	17/33	7.069	1	0.007843
7	dbsnp	rs7810530	G	A	ALLELIC	1/21	17/33	7.069	1	0.007843
7	dbsnp	rs2709915	A	G	ALLELIC	10/12	8/42	7.069	1	0.007843
7	dbsnp	rs810640	A	G	ALLELIC	1/21	17/33	7.069	1	0.007843
7	dbsnp	rs4732283	C	A	ALLELIC	10/12	8/42	7.069	1	0.007843
7	dbsnp	rs17225888	A	C	ALLELIC	1/21	17/33	7.069	1	0.007843
8	dbsnp	rs9918794	A	C	ALLELIC	1/21	17/33	7.069	1	0.007843
8	dbsnp	rs4871923	A	G	ALLELIC	1/21	17/33	7.069	1	0.007843
8	dbsnp	rs1119539	G	A	ALLELIC	10/12	8/42	7.069	1	0.007843
8	dbsnp	rs755162	A	C	ALLELIC	1/21	17/33	7.069	1	0.007843
8	dbsnp	rs1227646	A	G	ALLELIC	1/21	17/33	7.069	1	0.007843
8	dbsnp	rs1227649	A	G	ALLELIC	1/21	17/33	7.069	1	0.007843
8	dbsnp	rs7817332	A	G	ALLELIC	1/21	17/33	7.069	1	0.007843
8	dbsnp	rs2437769	A	G	ALLELIC	10/12	8/42	7.069	1	0.007843
8	dbsnp	rs13279316	G	A	ALLELIC	10/12	8/42	7.069	1	0.007843
8	dbsnp	rs10505532	A	C	ALLELIC	10/12	8/42	7.069	1	0.007843
8	dbsnp	rs1372454	A	G	ALLELIC	10/12	8/42	7.069	1	0.007843
8	dbsnp	rs4909356	A	G	ALLELIC	10/12	8/42	7.069	1	0.007843
8	dbsnp	rs6990911	G	A	ALLELIC	10/12	8/42	7.069	1	0.007843
8	dbsnp	rs1588022	A	G	ALLELIC	10/12	8/42	7.069	1	0.007843
8	dbsnp	rs7846671	A	G	ALLELIC	10/12	8/42	7.069	1	0.007843
9	dbsnp	rs12378556	G	A	ALLELIC	10/12	8/42	7.069	1	0.007843
9	dbsnp	rs10974423	A	G	ALLELIC	10/12	8/42	7.069	1	0.007843
9	dbsnp	rs10975148	G	A	ALLELIC	10/12	8/42	7.069	1	0.007843
9	dbsnp	rs717372	A	G	ALLELIC	10/12	8/42	7.069	1	0.007843
9	dbsnp	rs7035163	A	G	ALLELIC	10/12	8/42	7.069	1	0.007843
9	dbsnp	rs12346541	A	G	ALLELIC	1/21	17/33	7.069	1	0.007843
9	dbsnp	rs7776	C	A	ALLELIC	10/12	8/42	7.069	1	0.007843
9	dbsnp	rs7020021	A	G	ALLELIC	10/12	8/42	7.069	1	0.007843
10	dbsnp	rs2387739	G	A	ALLELIC	1/21	17/33	7.069	1	0.007843
10	dbsnp	rs17583338	A	G	ALLELIC	1/21	17/33	7.069	1	0.007843
10	dbsnp	rs2358277	A	G	ALLELIC	10/12	8/42	7.069	1	0.007843
10	dbsnp	rs2015920	G	A	ALLELIC	10/12	8/42	7.069	1	0.007843

10	dbsnp	rs11188799	G	A	ALLELIC	1/21	17/33	7.069	1	0.007843
10	dbsnp	rs4962696	A	G	ALLELIC	10/12	8/42	7.069	1	0.007843
11	dbsnp	rs10769175	A	G	ALLELIC	1/21	17/33	7.069	1	0.007843
11	dbsnp	rs1378357	G	A	ALLELIC	10/12	8/42	7.069	1	0.007843
11	dbsnp	rs2729843	G	A	ALLELIC	1/21	17/33	7.069	1	0.007843
11	dbsnp	rs7929471	A	G	ALLELIC	1/21	17/33	7.069	1	0.007843
11	dbsnp	rs663767	A	C	ALLELIC	1/21	17/33	7.069	1	0.007843
11	dbsnp	rs621724	C	A	ALLELIC	1/21	17/33	7.069	1	0.007843
12	dbsnp	rs4625554	G	A	ALLELIC	10/12	8/42	7.069	1	0.007843
12	dbsnp	rs4761873	A	G	ALLELIC	10/12	8/42	7.069	1	0.007843
12	dbsnp	rs11612513	A	C	ALLELIC	1/21	17/33	7.069	1	0.007843
12	dbsnp	rs1163656	A	G	ALLELIC	1/21	17/33	7.069	1	0.007843
12	dbsnp	rs7960030	G	A	ALLELIC	1/21	17/33	7.069	1	0.007843
13	dbsnp	rs9315439	A	G	ALLELIC	1/21	17/33	7.069	1	0.007843
13	dbsnp	rs1539549	A	G	ALLELIC	1/21	17/33	7.069	1	0.007843
13	dbsnp	rs1171090	A	G	ALLELIC	1/21	17/33	7.069	1	0.007843
13	dbsnp	rs12875111	A	G	ALLELIC	10/12	8/42	7.069	1	0.007843
13	dbsnp	rs2765584	G	A	ALLELIC	10/12	8/42	7.069	1	0.007843
14	dbsnp	rs8019056	A	G	ALLELIC	10/12	8/42	7.069	1	0.007843
14	dbsnp	rs740072	C	A	ALLELIC	10/12	8/42	7.069	1	0.007843
14	dbsnp	rs17096112	A	G	ALLELIC	10/12	8/42	7.069	1	0.007843
15	dbsnp	rs12909095	G	A	ALLELIC	10/12	8/42	7.069	1	0.007843
15	dbsnp	rs748404	G	A	ALLELIC	10/12	8/42	7.069	1	0.007843
15	dbsnp	rs7497273	G	A	ALLELIC	10/12	8/42	7.069	1	0.007843
15	dbsnp	rs4775419	A	G	ALLELIC	10/12	8/42	7.069	1	0.007843
15	dbsnp	rs17271123	A	C	ALLELIC	1/21	17/33	7.069	1	0.007843
15	dbsnp	rs12101383	A	G	ALLELIC	10/12	8/42	7.069	1	0.007843
15	dbsnp	rs7180119	C	A	ALLELIC	1/21	17/33	7.069	1	0.007843
16	dbsnp	rs737008	C	A	ALLELIC	10/12	8/42	7.069	1	0.007843
16	dbsnp	rs8057014	C	A	ALLELIC	10/12	8/42	7.069	1	0.007843
16	dbsnp	rs1992623	A	C	ALLELIC	10/12	8/42	7.069	1	0.007843
17	dbsnp	rs9912391	A	G	ALLELIC	1/21	17/33	7.069	1	0.007843
17	dbsnp	rs1642763	A	G	ALLELIC	1/21	17/33	7.069	1	0.007843
17	dbsnp	rs1641511	G	A	ALLELIC	1/21	17/33	7.069	1	0.007843
17	dbsnp	rs4054884	A	G	ALLELIC	1/21	17/33	7.069	1	0.007843
17	dbsnp	rs4792636	G	A	ALLELIC	1/21	17/33	7.069	1	0.007843
17	dbsnp	rs11657864	G	A	ALLELIC	10/12	8/42	7.069	1	0.007843
17	dbsnp	rs11080182	G	A	ALLELIC	10/12	8/42	7.069	1	0.007843
17	dbsnp	rs4793688	A	G	ALLELIC	1/21	17/33	7.069	1	0.007843
17	dbsnp	rs17192740	G	A	ALLELIC	10/12	8/42	7.069	1	0.007843
18	dbsnp	rs6506633	G	A	ALLELIC	10/12	8/42	7.069	1	0.007843
18	dbsnp	rs3863524	G	A	ALLELIC	10/12	8/42	7.069	1	0.007843
18	dbsnp	rs3851821	A	G	ALLELIC	10/12	8/42	7.069	1	0.007843
18	dbsnp	rs10502480	A	G	ALLELIC	10/12	8/42	7.069	1	0.007843
18	dbsnp	rs330326	A	G	ALLELIC	1/21	17/33	7.069	1	0.007843
18	dbsnp	rs8088726	A	G	ALLELIC	1/21	17/33	7.069	1	0.007843
18	dbsnp	rs12456911	G	A	ALLELIC	1/21	17/33	7.069	1	0.007843
18	dbsnp	rs1944577	A	G	ALLELIC	1/21	17/33	7.069	1	0.007843

18	dbsnp	rs6507940	A	G	ALLELIC	10/12	8/42	7.069	1	0.007843
18	dbsnp	rs17716559	G	A	ALLELIC	10/12	8/42	7.069	1	0.007843
18	dbsnp	rs4941253	G	A	ALLELIC	1/21	17/33	7.069	1	0.007843
19	dbsnp	rs247781	A	C	ALLELIC	10/12	8/42	7.069	1	0.007843
20	dbsnp	rs6085262	A	G	ALLELIC	10/12	8/42	7.069	1	0.007843
20	dbsnp	rs6085263	A	C	ALLELIC	10/12	8/42	7.069	1	0.007843
20	dbsnp	rs6040169	A	C	ALLELIC	1/21	17/33	7.069	1	0.007843
20	dbsnp	rs6040175	G	A	ALLELIC	1/21	17/33	7.069	1	0.007843
20	dbsnp	rs2423523	A	G	ALLELIC	1/21	17/33	7.069	1	0.007843
20	dbsnp	rs6044967	G	A	ALLELIC	10/12	8/42	7.069	1	0.007843
22	dbsnp	rs2232176	A	C	ALLELIC	1/21	17/33	7.069	1	0.007843
22	dbsnp	rs4822021	A	G	ALLELIC	10/12	8/42	7.069	1	0.007843
22	dbsnp	rs9611565	G	A	ALLELIC	10/12	8/42	7.069	1	0.007843
22	dbsnp	rs12484449	A	G	ALLELIC	10/12	8/42	7.069	1	0.007843
22	dbsnp	rs132902	G	A	ALLELIC	10/12	8/42	7.069	1	0.007843
22	dbsnp	rs11913873	G	A	ALLELIC	1/21	17/33	7.069	1	0.007843
23	dbsnp	rs5933725	G	A	ALLELIC	1/21	17/33	7.069	1	0.007843
23	dbsnp	rs5990069	A	G	ALLELIC	10/12	8/42	7.069	1	0.007843
23	dbsnp	rs1396305	A	G	ALLELIC	10/12	8/42	7.069	1	0.007843
23	dbsnp	rs3952885	G	A	ALLELIC	1/21	17/33	7.069	1	0.007843
23	dbsnp	rs2038452	G	A	ALLELIC	10/12	8/42	7.069	1	0.007843
23	dbsnp	rs1565828	G	A	ALLELIC	10/12	8/42	7.069	1	0.007843
23	dbsnp	rs1802288	A	G	ALLELIC	1/21	17/33	7.069	1	0.007843
4	dbsnp	rs10034133	G	A	ALLELIC	12/6	14/32	7.041	1	0.007966
7	dbsnp	rs2727564	G	A	ALLELIC	13/5	18/32	7.001	1	0.008148
1	dbsnp	rs10746477	A	G	ALLELIC	8/12	6/44	7	1	0.008151
1	dbsnp	rs3856273	A	G	ALLELIC	8/12	6/44	7	1	0.008151
1	dbsnp	rs764457	G	A	ALLELIC	8/12	6/44	7	1	0.008151
1	dbsnp	rs2454199	A	G	ALLELIC	8/12	6/44	7	1	0.008151
1	dbsnp	rs6666696	A	G	ALLELIC	8/12	6/44	7	1	0.008151
1	dbsnp	rs16846167	A	G	ALLELIC	0/20	14/36	7	1	0.008151
2	dbsnp	rs11891417	G	A	ALLELIC	5/15	2/48	7	1	0.008151
2	dbsnp	rs4260216	C	A	ALLELIC	15/5	20/30	7	1	0.008151
2	dbsnp	rs4952002	A	G	ALLELIC	5/15	30/20	7	1	0.008151
2	dbsnp	rs7595790	G	A	ALLELIC	5/15	2/48	7	1	0.008151
2	dbsnp	rs13418468	A	G	ALLELIC	5/15	30/20	7	1	0.008151
2	dbsnp	rs3914724	G	A	ALLELIC	5/15	2/48	7	1	0.008151
2	dbsnp	rs2931462	G	A	ALLELIC	5/15	2/48	7	1	0.008151
2	dbsnp	rs10191221	G	A	ALLELIC	5/15	2/48	7	1	0.008151
2	dbsnp	rs11681817	A	G	ALLELIC	5/15	2/48	7	1	0.008151
2	dbsnp	rs1358227	G	A	ALLELIC	5/15	2/48	7	1	0.008151
2	dbsnp	rs11688142	A	G	ALLELIC	5/15	2/48	7	1	0.008151
3	dbsnp	rs6789043	A	G	ALLELIC	15/5	20/30	7	1	0.008151
3	dbsnp	rs9311149	C	A	ALLELIC	15/5	20/30	7	1	0.008151
3	dbsnp	rs2302503	G	A	ALLELIC	15/5	20/30	7	1	0.008151
3	dbsnp	rs13086717	G	A	ALLELIC	5/15	2/48	7	1	0.008151
3	dbsnp	rs6809164	C	A	ALLELIC	0/20	14/36	7	1	0.008151
3	dbsnp	rs977159	A	G	ALLELIC	5/15	2/48	7	1	0.008151

3	dbsnp	rs7616632	C	A	ALLELIC	15/5	20/30	7	1	0.008151
3	dbsnp	rs9824917	A	C	ALLELIC	5/15	2/48	7	1	0.008151
3	dbsnp	rs1814982	G	A	ALLELIC	15/5	20/30	7	1	0.008151
3	dbsnp	rs1520137	A	G	ALLELIC	5/15	30/20	7	1	0.008151
4	dbsnp	rs9291627	A	G	ALLELIC	5/15	2/48	7	1	0.008151
4	dbsnp	rs6821591	A	G	ALLELIC	5/15	30/20	7	1	0.008151
4	dbsnp	rs6551661	G	A	ALLELIC	5/15	2/48	7	1	0.008151
4	dbsnp	rs998065	G	A	ALLELIC	15/5	20/30	7	1	0.008151
4	dbsnp	rs2298989	T	C	ALLELIC	5/15	30/20	7	1	0.008151
4	dbsnp	rs17049001	G	A	ALLELIC	5/15	2/48	7	1	0.008151
4	dbsnp	rs2043805	G	A	ALLELIC	5/15	2/48	7	1	0.008151
5	dbsnp	rs12516758	G	A	ALLELIC	0/20	14/36	7	1	0.008151
5	dbsnp	rs1510936	G	A	ALLELIC	8/12	6/44	7	1	0.008151
5	dbsnp	rs13170861	C	A	ALLELIC	15/5	20/30	7	1	0.008151
5	dbsnp	rs1000648	G	A	ALLELIC	5/15	2/48	7	1	0.008151
5	dbsnp	rs17771891	A	G	ALLELIC	8/12	6/44	7	1	0.008151
5	dbsnp	rs1016988	G	A	ALLELIC	8/12	6/44	7	1	0.008151
5	dbsnp	rs7730247	C	A	ALLELIC	8/12	6/44	7	1	0.008151
5	dbsnp	rs10059157	G	A	ALLELIC	5/15	2/48	7	1	0.008151
5	dbsnp	rs11135072	A	G	ALLELIC	5/15	30/20	7	1	0.008151
5	dbsnp	rs4869066	A	G	ALLELIC	0/20	14/36	7	1	0.008151
6	dbsnp	rs6906460	A	T	ALLELIC	0/20	14/36	7	1	0.008151
6	dbsnp	rs17315964	G	A	ALLELIC	5/15	2/48	7	1	0.008151
6	dbsnp	rs12943	G	A	ALLELIC	8/12	6/44	7	1	0.008151
6	dbsnp	rs6935267	A	C	ALLELIC	0/20	14/36	7	1	0.008151
6	dbsnp	rs12215687	G	A	ALLELIC	5/15	2/48	7	1	0.008151
7	dbsnp	rs10253443	A	G	ALLELIC	8/12	6/44	7	1	0.008151
8	dbsnp	rs9650614	A	G	ALLELIC	8/12	6/44	7	1	0.008151
8	dbsnp	rs11249895	A	G	ALLELIC	8/12	6/44	7	1	0.008151
8	dbsnp	rs3802196	A	C	ALLELIC	5/15	2/48	7	1	0.008151
8	dbsnp	rs2584364	A	G	ALLELIC	5/15	2/48	7	1	0.008151
9	dbsnp	rs4461956	C	A	ALLELIC	8/12	6/44	7	1	0.008151
9	dbsnp	rs10511827	G	A	ALLELIC	0/20	14/36	7	1	0.008151
9	dbsnp	rs1331436	A	G	ALLELIC	0/20	14/36	7	1	0.008151
9	dbsnp	rs7863684	A	G	ALLELIC	5/15	2/48	7	1	0.008151
10	dbsnp	rs4339935	C	A	ALLELIC	5/15	30/20	7	1	0.008151
10	dbsnp	rs4750422	G	A	ALLELIC	5/15	2/48	7	1	0.008151
10	dbsnp	rs1857579	G	A	ALLELIC	8/12	6/44	7	1	0.008151
10	dbsnp	rs8178992	A	G	ALLELIC	5/15	2/48	7	1	0.008151
10	dbsnp	rs2338044	A	G	ALLELIC	0/20	14/36	7	1	0.008151
10	dbsnp	rs12255651	G	A	ALLELIC	8/12	6/44	7	1	0.008151
10	dbsnp	rs16917302	C	A	ALLELIC	5/15	2/48	7	1	0.008151
10	dbsnp	rs2066158	G	A	ALLELIC	8/12	6/44	7	1	0.008151
10	dbsnp	rs10829351	G	A	ALLELIC	0/20	14/36	7	1	0.008151
11	dbsnp	rs11024580	A	C	ALLELIC	8/12	6/44	7	1	0.008151
11	dbsnp	rs16912392	A	C	ALLELIC	0/20	14/36	7	1	0.008151
11	dbsnp	rs11029147	A	G	ALLELIC	5/15	2/48	7	1	0.008151
11	dbsnp	rs6592663	G	A	ALLELIC	0/20	14/36	7	1	0.008151

12	dbsnp	rs492540	A	C	ALLELIC	8/12	6/44	7	1	0.008151
12	dbsnp	rs11056875	A	G	ALLELIC	8/12	6/44	7	1	0.008151
12	dbsnp	rs11171710	G	A	ALLELIC	5/15	30/20	7	1	0.008151
12	dbsnp	rs11180712	A	C	ALLELIC	15/5	20/30	7	1	0.008151
12	dbsnp	rs12823001	A	G	ALLELIC	5/15	30/20	7	1	0.008151
12	dbsnp	rs8553	A	G	ALLELIC	8/12	6/44	7	1	0.008151
12	dbsnp	rs7308394	A	G	ALLELIC	5/15	2/48	7	1	0.008151
12	dbsnp	rs6539050	G	A	ALLELIC	8/12	6/44	7	1	0.008151
12	dbsnp	rs17808695	G	A	ALLELIC	5/15	2/48	7	1	0.008151
12	dbsnp	rs2070873	A	C	ALLELIC	8/12	6/44	7	1	0.008151
12	dbsnp	rs1798813	A	G	ALLELIC	8/12	6/44	7	1	0.008151
12	dbsnp	rs12424139	A	G	ALLELIC	8/12	6/44	7	1	0.008151
13	dbsnp	rs9553887	A	G	ALLELIC	5/15	2/48	7	1	0.008151
13	dbsnp	rs11147629	G	A	ALLELIC	15/5	20/30	7	1	0.008151
13	dbsnp	rs301687	G	A	ALLELIC	5/15	2/48	7	1	0.008151
13	dbsnp	rs1590379	G	A	ALLELIC	8/12	6/44	7	1	0.008151
14	dbsnp	rs11159693	A	G	ALLELIC	5/15	2/48	7	1	0.008151
14	dbsnp	rs7155560	C	A	ALLELIC	5/15	2/48	7	1	0.008151
14	dbsnp	rs10133171	A	G	ALLELIC	5/15	30/20	7	1	0.008151
16	dbsnp	rs2738893	A	G	ALLELIC	5/15	30/20	7	1	0.008151
16	dbsnp	rs490662	G	A	ALLELIC	15/5	20/30	7	1	0.008151
17	dbsnp	rs12601358	C	A	ALLELIC	8/12	6/44	7	1	0.008151
17	dbsnp	rs17587658	A	G	ALLELIC	5/15	2/48	7	1	0.008151
17	dbsnp	rs3744089	G	A	ALLELIC	8/12	6/44	7	1	0.008151
17	dbsnp	rs9911155	A	C	ALLELIC	8/12	6/44	7	1	0.008151
17	dbsnp	rs7220127	G	A	ALLELIC	15/5	20/30	7	1	0.008151
17	dbsnp	rs12601558	C	A	ALLELIC	8/12	6/44	7	1	0.008151
18	dbsnp	rs11081459	G	A	ALLELIC	5/15	2/48	7	1	0.008151
18	dbsnp	rs264230	A	G	ALLELIC	5/15	30/20	7	1	0.008151
18	dbsnp	rs1507052	G	A	ALLELIC	5/15	30/20	7	1	0.008151
18	dbsnp	rs1947948	A	G	ALLELIC	5/15	30/20	7	1	0.008151
18	dbsnp	rs12969375	G	A	ALLELIC	5/15	2/48	7	1	0.008151
19	dbsnp	rs10422305	A	G	ALLELIC	8/12	6/44	7	1	0.008151
20	dbsnp	rs6111549	G	A	ALLELIC	0/20	14/36	7	1	0.008151
20	dbsnp	rs761269	A	G	ALLELIC	8/12	6/44	7	1	0.008151
20	dbsnp	rs11086907	G	A	ALLELIC	5/15	2/48	7	1	0.008151
20	dbsnp	rs7270917	A	G	ALLELIC	5/15	2/48	7	1	0.008151
21	dbsnp	rs926164	C	A	ALLELIC	8/12	6/44	7	1	0.008151
21	dbsnp	rs2187292	G	A	ALLELIC	5/15	30/20	7	1	0.008151
22	dbsnp	rs135793	A	G	ALLELIC	5/15	2/48	7	1	0.008151
22	dbsnp	rs135826	A	G	ALLELIC	5/15	2/48	7	1	0.008151
22	dbsnp	rs9616915	A	G	ALLELIC	15/5	20/30	7	1	0.008151
23	dbsnp	rs5916332	G	A	ALLELIC	5/15	2/48	7	1	0.008151
23	dbsnp	rs1551076	A	G	ALLELIC	5/15	2/48	7	1	0.008151
23	dbsnp	rs10482265	C	A	ALLELIC	5/15	2/48	7	1	0.008151
23	dbsnp	rs5990919	C	A	ALLELIC	5/15	2/48	7	1	0.008151
23	dbsnp	rs929018	A	C	ALLELIC	5/15	30/20	7	1	0.008151
23	dbsnp	rs6418252	C	A	ALLELIC	8/12	6/44	7	1	0.008151

1	dbsnp	rs4654991	G	A	ALLELIC	0/22	13/37	6.98	1	0.008241
1	dbsnp	rs3753270	A	G	ALLELIC	0/22	13/37	6.98	1	0.008241
1	dbsnp	rs11208830	A	G	ALLELIC	0/22	13/37	6.98	1	0.008241
1	dbsnp	rs1926301	G	A	ALLELIC	0/22	13/37	6.98	1	0.008241
1	dbsnp	rs11161635	G	A	ALLELIC	0/22	13/37	6.98	1	0.008241
1	dbsnp	rs16839051	G	A	ALLELIC	0/22	13/37	6.98	1	0.008241
2	dbsnp	rs6756361	A	G	ALLELIC	0/22	13/37	6.98	1	0.008241
2	dbsnp	rs713514	A	G	ALLELIC	0/22	13/37	6.98	1	0.008241
2	dbsnp	rs952455	G	A	ALLELIC	0/22	13/37	6.98	1	0.008241
2	dbsnp	rs2541191	G	A	ALLELIC	0/22	13/37	6.98	1	0.008241
2	dbsnp	rs6728107	G	A	ALLELIC	0/22	13/37	6.98	1	0.008241
2	dbsnp	rs10191711	A	C	ALLELIC	0/22	13/37	6.98	1	0.008241
2	dbsnp	rs10171662	A	G	ALLELIC	0/22	13/37	6.98	1	0.008241
2	dbsnp	rs10170310	G	A	ALLELIC	0/22	13/37	6.98	1	0.008241
2	dbsnp	rs1882891	A	C	ALLELIC	0/22	13/37	6.98	1	0.008241
2	dbsnp	rs2736619	A	G	ALLELIC	0/22	13/37	6.98	1	0.008241
2	dbsnp	rs11675231	G	A	ALLELIC	0/22	13/37	6.98	1	0.008241
3	dbsnp	rs10780025	G	A	ALLELIC	0/22	13/37	6.98	1	0.008241
3	dbsnp	rs4591494	G	A	ALLELIC	0/22	13/37	6.98	1	0.008241
3	dbsnp	rs6442403	G	A	ALLELIC	0/22	13/37	6.98	1	0.008241
3	dbsnp	rs7640776	A	G	ALLELIC	0/22	13/37	6.98	1	0.008241
3	dbsnp	rs7609933	A	G	ALLELIC	0/22	13/37	6.98	1	0.008241
4	dbsnp	rs566672	G	A	ALLELIC	0/22	13/37	6.98	1	0.008241
5	dbsnp	rs16902679	A	G	ALLELIC	0/22	13/37	6.98	1	0.008241
5	dbsnp	rs10473352	G	A	ALLELIC	0/22	13/37	6.98	1	0.008241
6	dbsnp	rs4716077	A	G	ALLELIC	0/22	13/37	6.98	1	0.008241
6	dbsnp	rs10484434	A	G	ALLELIC	0/22	13/37	6.98	1	0.008241
6	dbsnp	rs11755492	G	A	ALLELIC	0/22	13/37	6.98	1	0.008241
6	dbsnp	rs4413610	A	G	ALLELIC	0/22	13/37	6.98	1	0.008241
6	dbsnp	rs280308	G	A	ALLELIC	0/22	13/37	6.98	1	0.008241
6	dbsnp	rs6453613	A	G	ALLELIC	0/22	13/37	6.98	1	0.008241
6	dbsnp	rs3798939	A	G	ALLELIC	0/22	13/37	6.98	1	0.008241
7	dbsnp	rs2079459	G	A	ALLELIC	0/22	13/37	6.98	1	0.008241
7	dbsnp	rs2723512	A	G	ALLELIC	0/22	13/37	6.98	1	0.008241
7	dbsnp	rs12113960	G	A	ALLELIC	0/22	13/37	6.98	1	0.008241
7	dbsnp	rs1994468	G	A	ALLELIC	0/22	13/37	6.98	1	0.008241
8	dbsnp	rs11135856	A	G	ALLELIC	0/22	13/37	6.98	1	0.008241
8	dbsnp	rs7462051	A	G	ALLELIC	0/22	13/37	6.98	1	0.008241
8	dbsnp	rs7817246	G	A	ALLELIC	0/22	13/37	6.98	1	0.008241
9	dbsnp	rs1322473	A	G	ALLELIC	0/22	13/37	6.98	1	0.008241
9	dbsnp	rs7871884	G	A	ALLELIC	0/22	13/37	6.98	1	0.008241
9	dbsnp	rs11791447	A	G	ALLELIC	0/22	13/37	6.98	1	0.008241
9	dbsnp	rs2289006	A	C	ALLELIC	0/22	13/37	6.98	1	0.008241
9	dbsnp	rs2033541	A	G	ALLELIC	0/22	13/37	6.98	1	0.008241
9	dbsnp	rs12685193	C	A	ALLELIC	0/22	13/37	6.98	1	0.008241
9	dbsnp	rs10868791	G	A	ALLELIC	0/22	13/37	6.98	1	0.008241
10	dbsnp	rs6585463	G	A	ALLELIC	0/22	13/37	6.98	1	0.008241
11	dbsnp	rs1486536	A	C	ALLELIC	0/22	13/37	6.98	1	0.008241

11	dbsnp	rs2403435	G	A	ALLELIC	0/22	13/37	6.98	1	0.008241
11	dbsnp	rs7930272	A	G	ALLELIC	0/22	13/37	6.98	1	0.008241
11	dbsnp	rs10768709	A	G	ALLELIC	0/22	13/37	6.98	1	0.008241
12	dbsnp	rs2300266	C	A	ALLELIC	0/22	13/37	6.98	1	0.008241
12	dbsnp	rs704219	A	G	ALLELIC	0/22	13/37	6.98	1	0.008241
12	dbsnp	rs1436121	A	G	ALLELIC	0/22	13/37	6.98	1	0.008241
12	dbsnp	rs4762643	A	G	ALLELIC	0/22	13/37	6.98	1	0.008241
12	dbsnp	rs1982138	G	A	ALLELIC	0/22	13/37	6.98	1	0.008241
13	dbsnp	rs9542034	A	C	ALLELIC	0/22	13/37	6.98	1	0.008241
14	dbsnp	rs1980523	C	A	ALLELIC	0/22	13/37	6.98	1	0.008241
14	dbsnp	rs3783899	A	G	ALLELIC	0/22	13/37	6.98	1	0.008241
15	dbsnp	rs902025	A	G	ALLELIC	0/22	13/37	6.98	1	0.008241
16	dbsnp	rs11076787	A	G	ALLELIC	0/22	13/37	6.98	1	0.008241
17	dbsnp	rs12941504	G	A	ALLELIC	0/22	13/37	6.98	1	0.008241
20	dbsnp	rs2269047	G	A	ALLELIC	0/22	13/37	6.98	1	0.008241
20	dbsnp	rs6016274	A	G	ALLELIC	0/22	13/37	6.98	1	0.008241
20	dbsnp	rs6028942	G	A	ALLELIC	0/22	13/37	6.98	1	0.008241
20	dbsnp	rs4809958	C	A	ALLELIC	0/22	13/37	6.98	1	0.008241
20	dbsnp	rs6128541	G	A	ALLELIC	0/22	13/37	6.98	1	0.008241
21	dbsnp	rs7278274	A	G	ALLELIC	0/22	13/37	6.98	1	0.008241
23	dbsnp	rs12559033	A	G	ALLELIC	0/22	13/37	6.98	1	0.008241
23	dbsnp	rs7061649	A	G	ALLELIC	0/22	13/37	6.98	1	0.008241
23	dbsnp	rs12852604	A	G	ALLELIC	0/22	13/37	6.98	1	0.008241
1	dbsnp	rs17131041	A	G	ALLELIC	4/16	1/49	6.978	1	0.00825
1	dbsnp	rs12747498	G	A	ALLELIC	4/16	1/49	6.978	1	0.00825
1	dbsnp	rs16843957	A	G	ALLELIC	4/16	1/49	6.978	1	0.00825
2	dbsnp	rs7598895	G	A	ALLELIC	4/16	1/49	6.978	1	0.00825
2	dbsnp	rs7591286	A	G	ALLELIC	4/16	1/49	6.978	1	0.00825
4	dbsnp	rs4835011	G	A	ALLELIC	4/16	1/49	6.978	1	0.00825
4	dbsnp	rs6537368	G	A	ALLELIC	4/16	1/49	6.978	1	0.00825
4	dbsnp	rs4379045	A	G	ALLELIC	4/16	1/49	6.978	1	0.00825
4	dbsnp	rs4414935	A	G	ALLELIC	4/16	1/49	6.978	1	0.00825
5	dbsnp	rs2291628	A	G	ALLELIC	4/16	1/49	6.978	1	0.00825
5	dbsnp	rs17703748	A	G	ALLELIC	4/16	1/49	6.978	1	0.00825
5	dbsnp	rs9313473	A	G	ALLELIC	4/16	1/49	6.978	1	0.00825
5	dbsnp	rs10516111	G	A	ALLELIC	4/16	1/49	6.978	1	0.00825
5	dbsnp	rs335423	A	G	ALLELIC	4/16	1/49	6.978	1	0.00825
6	dbsnp	rs7749754	G	A	ALLELIC	4/16	1/49	6.978	1	0.00825
6	dbsnp	rs7761161	G	A	ALLELIC	4/16	1/49	6.978	1	0.00825
7	dbsnp	rs17161595	A	C	ALLELIC	4/16	1/49	6.978	1	0.00825
7	dbsnp	rs11975282	G	A	ALLELIC	4/16	1/49	6.978	1	0.00825
8	dbsnp	rs2450564	A	G	ALLELIC	4/16	1/49	6.978	1	0.00825
9	dbsnp	rs2811712	G	A	ALLELIC	4/16	1/49	6.978	1	0.00825
9	dbsnp	rs1333035	G	A	ALLELIC	4/16	1/49	6.978	1	0.00825
9	dbsnp	rs1333034	G	A	ALLELIC	4/16	1/49	6.978	1	0.00825
10	dbsnp	rs1150037	A	G	ALLELIC	4/16	1/49	6.978	1	0.00825
10	dbsnp	rs1150039	A	G	ALLELIC	4/16	1/49	6.978	1	0.00825
11	dbsnp	rs2399749	G	A	ALLELIC	4/16	1/49	6.978	1	0.00825

12	dbsnp	rs7486836	A	G	ALLELIC	4/16	1/49	6.978	1	0.00825
12	dbsnp	rs12303590	G	A	ALLELIC	4/16	1/49	6.978	1	0.00825
12	dbsnp	rs3849228	G	A	ALLELIC	4/16	1/49	6.978	1	0.00825
12	dbsnp	rs345644	A	C	ALLELIC	4/16	1/49	6.978	1	0.00825
12	dbsnp	rs345659	G	A	ALLELIC	4/16	1/49	6.978	1	0.00825
13	dbsnp	rs9556879	A	G	ALLELIC	4/16	1/49	6.978	1	0.00825
15	dbsnp	rs1677236	A	G	ALLELIC	4/16	1/49	6.978	1	0.00825
15	dbsnp	rs2119940	G	A	ALLELIC	4/16	1/49	6.978	1	0.00825
15	dbsnp	rs9783690	A	G	ALLELIC	4/16	1/49	6.978	1	0.00825
15	dbsnp	rs8041078	A	G	ALLELIC	4/16	1/49	6.978	1	0.00825
16	dbsnp	rs7187075	A	G	ALLELIC	4/16	1/49	6.978	1	0.00825
16	dbsnp	rs4782612	A	G	ALLELIC	4/16	1/49	6.978	1	0.00825
20	dbsnp	rs6123921	G	A	ALLELIC	4/16	1/49	6.978	1	0.00825
23	dbsnp	rs178993	A	C	ALLELIC	4/16	1/49	6.978	1	0.00825
23	dbsnp	rs12557638	A	G	ALLELIC	4/16	1/49	6.978	1	0.00825
23	dbsnp	rs5912942	A	G	ALLELIC	4/16	1/49	6.978	1	0.00825
2	dbsnp	rs441098	A	G	ALLELIC	1/19	18/32	6.942	1	0.008419
3	dbsnp	rs9824781	A	G	ALLELIC	1/19	18/32	6.942	1	0.008419
3	dbsnp	rs1460118	G	A	ALLELIC	1/19	18/32	6.942	1	0.008419
3	dbsnp	rs777490	A	G	ALLELIC	1/19	18/32	6.942	1	0.008419
4	dbsnp	rs2949619	A	G	ALLELIC	1/19	18/32	6.942	1	0.008419
4	dbsnp	rs3113500	A	G	ALLELIC	1/19	18/32	6.942	1	0.008419
4	dbsnp	rs10017800	A	G	ALLELIC	1/19	18/32	6.942	1	0.008419
6	dbsnp	rs213228	C	A	ALLELIC	1/19	18/32	6.942	1	0.008419
6	dbsnp	rs3734563	G	A	ALLELIC	1/19	18/32	6.942	1	0.008419
6	dbsnp	rs16894060	G	A	ALLELIC	1/19	18/32	6.942	1	0.008419
6	dbsnp	rs4357130	C	A	ALLELIC	1/19	18/32	6.942	1	0.008419
6	dbsnp	rs6908137	C	A	ALLELIC	1/19	18/32	6.942	1	0.008419
6	dbsnp	rs4254981	C	A	ALLELIC	1/19	18/32	6.942	1	0.008419
6	dbsnp	rs1124131	C	A	ALLELIC	1/19	18/32	6.942	1	0.008419
6	dbsnp	rs6922169	A	G	ALLELIC	1/19	18/32	6.942	1	0.008419
6	dbsnp	rs3800328	A	G	ALLELIC	1/19	18/32	6.942	1	0.008419
6	dbsnp	rs7740351	C	A	ALLELIC	1/19	18/32	6.942	1	0.008419
7	dbsnp	rs992880	A	G	ALLELIC	1/19	18/32	6.942	1	0.008419
12	dbsnp	rs2303963	G	A	ALLELIC	1/19	18/32	6.942	1	0.008419
12	dbsnp	rs1593519	A	G	ALLELIC	1/19	18/32	6.942	1	0.008419
12	dbsnp	rs1426367	C	A	ALLELIC	1/19	18/32	6.942	1	0.008419
12	dbsnp	rs2551394	G	A	ALLELIC	1/19	18/32	6.942	1	0.008419
12	dbsnp	rs11067587	G	A	ALLELIC	1/19	18/32	6.942	1	0.008419
12	dbsnp	rs7313293	G	A	ALLELIC	1/19	18/32	6.942	1	0.008419
14	dbsnp	rs1951083	G	A	ALLELIC	1/19	18/32	6.942	1	0.008419
14	dbsnp	rs1609698	G	A	ALLELIC	1/19	18/32	6.942	1	0.008419
16	dbsnp	rs2914457	G	A	ALLELIC	1/19	18/32	6.942	1	0.008419
17	dbsnp	rs6504595	A	C	ALLELIC	1/19	18/32	6.942	1	0.008419
18	dbsnp	rs731665	A	G	ALLELIC	1/19	18/32	6.942	1	0.008419
19	dbsnp	rs1042122	G	A	ALLELIC	1/19	18/32	6.942	1	0.008419
20	dbsnp	rs2235749	A	G	ALLELIC	1/19	18/32	6.942	1	0.008419
20	dbsnp	rs910079	G	A	ALLELIC	1/19	18/32	6.942	1	0.008419

22	dbsnp	rs2285164	A	G	ALLELIC	1/19	18/32	6.942	1	0.008419
1	dbsnp	rs16840051	A	G	ALLELIC	3/19	23/27	6.936	1	0.008448
1	dbsnp	rs1485471	A	G	ALLELIC	3/19	23/27	6.936	1	0.008448
1	dbsnp	rs11577575	A	G	ALLELIC	3/19	23/27	6.936	1	0.008448
1	dbsnp	rs2147030	A	G	ALLELIC	3/19	23/27	6.936	1	0.008448
1	dbsnp	rs12135811	A	G	ALLELIC	3/19	23/27	6.936	1	0.008448
2	dbsnp	rs747709	A	G	ALLELIC	3/19	23/27	6.936	1	0.008448
2	dbsnp	rs10191680	A	G	ALLELIC	3/19	23/27	6.936	1	0.008448
2	dbsnp	rs2678289	A	G	ALLELIC	3/19	23/27	6.936	1	0.008448
2	dbsnp	rs3768969	A	G	ALLELIC	3/19	23/27	6.936	1	0.008448
3	dbsnp	rs1564257	A	G	ALLELIC	3/19	23/27	6.936	1	0.008448
4	dbsnp	rs3733280	G	A	ALLELIC	3/19	23/27	6.936	1	0.008448
4	dbsnp	rs1440743	C	A	ALLELIC	3/19	23/27	6.936	1	0.008448
4	dbsnp	rs11723570	A	G	ALLELIC	3/19	23/27	6.936	1	0.008448
5	dbsnp	rs7725217	C	A	ALLELIC	3/19	23/27	6.936	1	0.008448
5	dbsnp	rs11747629	A	G	ALLELIC	3/19	23/27	6.936	1	0.008448
5	dbsnp	rs13171757	A	G	ALLELIC	3/19	23/27	6.936	1	0.008448
6	dbsnp	rs9293982	G	A	ALLELIC	3/19	23/27	6.936	1	0.008448
6	dbsnp	rs12204999	A	C	ALLELIC	3/19	23/27	6.936	1	0.008448
6	dbsnp	rs6911971	A	G	ALLELIC	3/19	23/27	6.936	1	0.008448
6	dbsnp	rs4709075	G	A	ALLELIC	3/19	23/27	6.936	1	0.008448
9	dbsnp	rs13295101	G	A	ALLELIC	3/19	23/27	6.936	1	0.008448
10	dbsnp	rs7087371	G	A	ALLELIC	3/19	23/27	6.936	1	0.008448
10	dbsnp	rs2861579	A	G	ALLELIC	3/19	23/27	6.936	1	0.008448
11	dbsnp	rs4084127	A	G	ALLELIC	3/19	23/27	6.936	1	0.008448
11	dbsnp	rs4936120	A	G	ALLELIC	3/19	23/27	6.936	1	0.008448
11	dbsnp	rs12279359	A	G	ALLELIC	3/19	23/27	6.936	1	0.008448
12	dbsnp	rs12368476	A	G	ALLELIC	3/19	23/27	6.936	1	0.008448
12	dbsnp	rs2343877	A	G	ALLELIC	3/19	23/27	6.936	1	0.008448
12	dbsnp	rs11115542	A	G	ALLELIC	3/19	23/27	6.936	1	0.008448
12	dbsnp	rs4442612	G	A	ALLELIC	3/19	23/27	6.936	1	0.008448
13	dbsnp	rs1417482	A	G	ALLELIC	3/19	23/27	6.936	1	0.008448
13	dbsnp	rs2388538	G	A	ALLELIC	3/19	23/27	6.936	1	0.008448
13	dbsnp	rs689338	A	G	ALLELIC	3/19	23/27	6.936	1	0.008448
13	dbsnp	rs493369	A	G	ALLELIC	3/19	23/27	6.936	1	0.008448
13	dbsnp	rs2351871	G	A	ALLELIC	3/19	23/27	6.936	1	0.008448
13	dbsnp	rs9589298	G	A	ALLELIC	3/19	23/27	6.936	1	0.008448
13	dbsnp	rs2793701	A	C	ALLELIC	3/19	23/27	6.936	1	0.008448
13	dbsnp	rs1570896	G	A	ALLELIC	3/19	23/27	6.936	1	0.008448
13	dbsnp	rs1952590	A	G	ALLELIC	3/19	23/27	6.936	1	0.008448
13	dbsnp	rs9557986	G	A	ALLELIC	3/19	23/27	6.936	1	0.008448
14	dbsnp	rs12372906	A	G	ALLELIC	3/19	23/27	6.936	1	0.008448
17	dbsnp	rs2191113	G	A	ALLELIC	3/19	23/27	6.936	1	0.008448
18	dbsnp	rs2957145	G	A	ALLELIC	3/19	23/27	6.936	1	0.008448
19	dbsnp	rs506425	G	A	ALLELIC	3/19	23/27	6.936	1	0.008448
20	dbsnp	rs6109143	C	A	ALLELIC	3/19	23/27	6.936	1	0.008448
20	dbsnp	rs6036896	A	G	ALLELIC	3/19	23/27	6.936	1	0.008448
20	dbsnp	rs12480916	C	A	ALLELIC	3/19	23/27	6.936	1	0.008448

20	dbsnp	rs33992134	A	G	ALLELIC	3/19	23/27	6.936	1	0.008448
21	dbsnp	rs396032	A	G	ALLELIC	3/19	23/27	6.936	1	0.008448
21	dbsnp	rs7279991	A	G	ALLELIC	3/19	23/27	6.936	1	0.008448
22	dbsnp	rs756638	A	G	ALLELIC	3/19	23/27	6.936	1	0.008448
23	dbsnp	rs6528242	A	C	ALLELIC	3/19	23/27	6.936	1	0.008448
23	dbsnp	rs17315891	A	G	ALLELIC	3/19	23/27	6.936	1	0.008448
23	dbsnp	rs4503212	G	A	ALLELIC	3/19	23/27	6.936	1	0.008448
23	dbsnp	rs4364814	A	C	ALLELIC	3/19	23/27	6.936	1	0.008448
2	dbsnp	rs11127081	G	A	ALLELIC	8/10	6/38	6.935	1	0.00845
11	dbsnp	rs7931562	A	G	ALLELIC	8/10	6/38	6.935	1	0.00845
2	dbsnp	rs17560229	G	A	ALLELIC	6/14	3/45	6.934	1	0.008455
9	dbsnp	rs3758271	G	A	ALLELIC	6/14	3/45	6.934	1	0.008455
23	dbsnp	rs6617635	G	A	ALLELIC	6/14	3/45	6.934	1	0.008455
8	dbsnp	rs1032406	G	A	ALLELIC	12/8	12/34	6.928	1	0.008486
19	dbsnp	rs3786835	A	G	ALLELIC	6/16	26/16	6.926	1	0.008493
1	dbsnp	rs4418592	G	A	ALLELIC	3/17	0/44	6.925	1	0.008502
20	dbsnp	rs4809751	G	A	ALLELIC	3/13	23/17	6.899	1	0.008622
0	dbsnp	rs12455984	A	G	ALLELIC	13/7	14/32	6.889	1	0.008671
1	dbsnp	rs885846	A	G	ALLELIC	2/20	20/30	6.879	1	0.008723
1	dbsnp	rs1185700	A	G	ALLELIC	2/20	20/30	6.879	1	0.008723
1	dbsnp	rs12074264	G	A	ALLELIC	2/20	20/30	6.879	1	0.008723
1	dbsnp	rs10801344	C	A	ALLELIC	2/20	20/30	6.879	1	0.008723
1	dbsnp	rs16843742	G	A	ALLELIC	2/20	20/30	6.879	1	0.008723
2	dbsnp	rs7592040	A	G	ALLELIC	2/20	20/30	6.879	1	0.008723
2	dbsnp	rs12712508	G	A	ALLELIC	2/20	20/30	6.879	1	0.008723
2	dbsnp	rs3755196	G	A	ALLELIC	2/20	20/30	6.879	1	0.008723
2	dbsnp	rs13406184	G	A	ALLELIC	2/20	20/30	6.879	1	0.008723
2	dbsnp	rs7608332	G	A	ALLELIC	2/20	20/30	6.879	1	0.008723
2	dbsnp	rs1541980	G	A	ALLELIC	2/20	20/30	6.879	1	0.008723
2	dbsnp	rs4302206	A	G	ALLELIC	2/20	20/30	6.879	1	0.008723
2	dbsnp	rs2250204	G	A	ALLELIC	2/20	20/30	6.879	1	0.008723
2	dbsnp	rs7592897	A	G	ALLELIC	2/20	20/30	6.879	1	0.008723
2	dbsnp	rs10498208	G	A	ALLELIC	2/20	20/30	6.879	1	0.008723
2	dbsnp	rs1109991	G	A	ALLELIC	2/20	20/30	6.879	1	0.008723
2	dbsnp	rs4487050	G	A	ALLELIC	2/20	20/30	6.879	1	0.008723
3	dbsnp	rs6766464	G	A	ALLELIC	2/20	20/30	6.879	1	0.008723
3	dbsnp	rs3856765	G	A	ALLELIC	2/20	20/30	6.879	1	0.008723
3	dbsnp	rs2175927	G	A	ALLELIC	2/20	20/30	6.879	1	0.008723
3	dbsnp	rs1877818	G	A	ALLELIC	2/20	20/30	6.879	1	0.008723
3	dbsnp	rs12491930	G	A	ALLELIC	2/20	20/30	6.879	1	0.008723
3	dbsnp	rs2722019	G	A	ALLELIC	2/20	20/30	6.879	1	0.008723
3	dbsnp	rs2029034	A	G	ALLELIC	2/20	20/30	6.879	1	0.008723
3	dbsnp	rs4365667	A	C	ALLELIC	2/20	20/30	6.879	1	0.008723
4	dbsnp	rs6448303	A	G	ALLELIC	2/20	20/30	6.879	1	0.008723
4	dbsnp	rs6833072	G	A	ALLELIC	2/20	20/30	6.879	1	0.008723
4	dbsnp	rs12511477	A	G	ALLELIC	2/20	20/30	6.879	1	0.008723
4	dbsnp	rs6535838	C	A	ALLELIC	2/20	20/30	6.879	1	0.008723
4	dbsnp	rs10020119	C	A	ALLELIC	2/20	20/30	6.879	1	0.008723

4	dbsnp	rs11734029	A	G	ALLELIC	2/20	20/30	6.879	1	0.008723
5	dbsnp	rs1532331	C	A	ALLELIC	2/20	20/30	6.879	1	0.008723
5	dbsnp	rs6895341	A	G	ALLELIC	2/20	20/30	6.879	1	0.008723
6	dbsnp	rs9296990	A	G	ALLELIC	2/20	20/30	6.879	1	0.008723
6	dbsnp	rs1778484	G	A	ALLELIC	2/20	20/30	6.879	1	0.008723
6	dbsnp	rs6903535	G	A	ALLELIC	2/20	20/30	6.879	1	0.008723
6	dbsnp	rs2273017	G	A	ALLELIC	2/20	20/30	6.879	1	0.008723
6	dbsnp	rs12207548	A	G	ALLELIC	2/20	20/30	6.879	1	0.008723
6	dbsnp	rs4546468	A	G	ALLELIC	2/20	20/30	6.879	1	0.008723
6	dbsnp	rs1953319	G	A	ALLELIC	2/20	20/30	6.879	1	0.008723
7	dbsnp	rs17459166	A	G	ALLELIC	2/20	20/30	6.879	1	0.008723
7	dbsnp	rs11769827	A	G	ALLELIC	2/20	20/30	6.879	1	0.008723
7	dbsnp	rs6963259	G	A	ALLELIC	2/20	20/30	6.879	1	0.008723
7	dbsnp	rs1209254	A	G	ALLELIC	2/20	20/30	6.879	1	0.008723
7	dbsnp	rs6979626	A	G	ALLELIC	2/20	20/30	6.879	1	0.008723
8	dbsnp	rs2599644	A	G	ALLELIC	2/20	20/30	6.879	1	0.008723
8	dbsnp	rs11998282	G	A	ALLELIC	2/20	20/30	6.879	1	0.008723
9	dbsnp	rs2849049	A	C	ALLELIC	2/20	20/30	6.879	1	0.008723
9	dbsnp	rs4617221	G	A	ALLELIC	2/20	20/30	6.879	1	0.008723
9	dbsnp	rs894674	A	G	ALLELIC	2/20	20/30	6.879	1	0.008723
10	dbsnp	rs11253471	A	G	ALLELIC	2/20	20/30	6.879	1	0.008723
10	dbsnp	rs12358475	A	G	ALLELIC	2/20	20/30	6.879	1	0.008723
10	dbsnp	rs1058202	A	G	ALLELIC	2/20	20/30	6.879	1	0.008723
10	dbsnp	rs2812425	A	G	ALLELIC	2/20	20/30	6.879	1	0.008723
10	dbsnp	rs1268514	A	C	ALLELIC	2/20	20/30	6.879	1	0.008723
11	dbsnp	rs443661	C	A	ALLELIC	2/20	20/30	6.879	1	0.008723
11	dbsnp	rs494942	A	C	ALLELIC	2/20	20/30	6.879	1	0.008723
11	dbsnp	rs1648144	A	G	ALLELIC	2/20	20/30	6.879	1	0.008723
11	dbsnp	rs11215196	A	G	ALLELIC	2/20	20/30	6.879	1	0.008723
12	dbsnp	rs7297554	A	G	ALLELIC	2/20	20/30	6.879	1	0.008723
13	dbsnp	rs9528685	G	A	ALLELIC	2/20	20/30	6.879	1	0.008723
14	dbsnp	rs1461556	G	A	ALLELIC	2/20	20/30	6.879	1	0.008723
14	dbsnp	rs12878653	C	A	ALLELIC	2/20	20/30	6.879	1	0.008723
15	dbsnp	rs17271534	A	G	ALLELIC	2/20	20/30	6.879	1	0.008723
15	dbsnp	rs7182577	C	A	ALLELIC	2/20	20/30	6.879	1	0.008723
15	dbsnp	rs2270635	A	G	ALLELIC	2/20	20/30	6.879	1	0.008723
17	dbsnp	rs9898946	G	A	ALLELIC	2/20	20/30	6.879	1	0.008723
18	dbsnp	rs1529210	G	A	ALLELIC	2/20	20/30	6.879	1	0.008723
19	dbsnp	rs2059877	A	C	ALLELIC	2/20	20/30	6.879	1	0.008723
20	dbsnp	rs6124039	A	G	ALLELIC	2/20	20/30	6.879	1	0.008723
21	dbsnp	rs2830400	A	G	ALLELIC	2/20	20/30	6.879	1	0.008723
21	dbsnp	rs2300306	A	G	ALLELIC	2/20	20/30	6.879	1	0.008723
21	dbsnp	rs13049109	A	G	ALLELIC	2/20	20/30	6.879	1	0.008723
22	dbsnp	rs9967	G	A	ALLELIC	2/20	20/30	6.879	1	0.008723
23	dbsnp	rs5912125	G	A	ALLELIC	2/20	20/30	6.879	1	0.008723
23	dbsnp	rs2361298	G	A	ALLELIC	2/20	20/30	6.879	1	0.008723
23	dbsnp	rs7060868	A	G	ALLELIC	2/20	20/30	6.879	1	0.008723
23	dbsnp	rs5951349	A	G	ALLELIC	2/20	20/30	6.879	1	0.008723

6	dbsnp	rs6906639	C	A	ALLELIC	4/14	1/43	6.857	1	0.008829
2	dbsnp	rs7584993	C	A	ALLELIC	10/10	9/39	6.847	1	0.008877
8	dbsnp	rs4006555	G	A	ALLELIC	10/10	9/39	6.847	1	0.008877
1	dbsnp	rs12073545	C	A	ALLELIC	12/10	11/37	6.841	1	0.00891
11	dbsnp	rs7950933	G	A	ALLELIC	12/10	11/37	6.841	1	0.00891
18	dbsnp	rs1940994	G	A	ALLELIC	12/10	11/37	6.841	1	0.00891
1	dbsnp	rs16844884	G	A	ALLELIC	3/19	0/48	6.839	1	0.008921
1	dbsnp	rs16830311	A	C	ALLELIC	3/19	0/48	6.839	1	0.008921
1	dbsnp	rs17021749	A	G	ALLELIC	3/19	0/48	6.839	1	0.008921
1	dbsnp	rs10925013	G	A	ALLELIC	3/19	0/48	6.839	1	0.008921
2	dbsnp	rs4233772	G	A	ALLELIC	3/19	0/48	6.839	1	0.008921
4	dbsnp	rs10032586	G	A	ALLELIC	3/19	0/48	6.839	1	0.008921
4	dbsnp	rs13130490	G	A	ALLELIC	3/19	0/48	6.839	1	0.008921
5	dbsnp	rs435913	A	G	ALLELIC	3/19	0/48	6.839	1	0.008921
5	dbsnp	rs2290674	A	G	ALLELIC	3/19	0/48	6.839	1	0.008921
5	dbsnp	rs6891420	C	A	ALLELIC	3/19	0/48	6.839	1	0.008921
6	dbsnp	rs16890508	G	A	ALLELIC	3/19	0/48	6.839	1	0.008921
6	dbsnp	rs9262138	G	A	ALLELIC	3/19	0/48	6.839	1	0.008921
7	dbsnp	rs4148851	T	A	ALLELIC	3/19	0/48	6.839	1	0.008921
7	dbsnp	rs844522	A	G	ALLELIC	3/19	0/48	6.839	1	0.008921
8	dbsnp	rs17632120	C	A	ALLELIC	3/19	0/48	6.839	1	0.008921
8	dbsnp	rs34259886	A	G	ALLELIC	3/19	0/48	6.839	1	0.008921
9	dbsnp	rs9410127	A	G	ALLELIC	3/19	0/48	6.839	1	0.008921
10	dbsnp	rs2486507	G	A	ALLELIC	3/19	0/48	6.839	1	0.008921
11	dbsnp	rs9665778	G	A	ALLELIC	3/19	0/48	6.839	1	0.008921
11	dbsnp	rs4257001	G	A	ALLELIC	3/19	0/48	6.839	1	0.008921
11	dbsnp	rs12360927	A	G	ALLELIC	3/19	0/48	6.839	1	0.008921
12	dbsnp	rs2388061	C	A	ALLELIC	3/19	0/48	6.839	1	0.008921
17	dbsnp	rs4412997	A	G	ALLELIC	3/19	0/48	6.839	1	0.008921
18	dbsnp	rs7239105	G	A	ALLELIC	3/19	0/48	6.839	1	0.008921
23	dbsnp	rs5931383	G	A	ALLELIC	3/19	0/48	6.839	1	0.008921
23	dbsnp	rs6608322	A	C	ALLELIC	3/19	0/48	6.839	1	0.008921
6	dbsnp	rs3127640	C	A	ALLELIC	5/17	27/21	6.831	1	0.008957
20	dbsnp	rs2244895	A	C	ALLELIC	5/17	27/21	6.831	1	0.008957
23	dbsnp	rs2050399	G	A	ALLELIC	5/17	27/21	6.831	1	0.008957
1	dbsnp	rs4845962	A	G	ALLELIC	5/17	28/22	6.813	1	0.009051
1	dbsnp	rs11576243	G	A	ALLELIC	5/17	28/22	6.813	1	0.009051
1	dbsnp	rs12740360	A	G	ALLELIC	5/17	28/22	6.813	1	0.009051
1	dbsnp	rs7550138	C	A	ALLELIC	5/17	28/22	6.813	1	0.009051
1	dbsnp	rs4656260	G	A	ALLELIC	5/17	28/22	6.813	1	0.009051
1	dbsnp	rs6679871	G	A	ALLELIC	5/17	28/22	6.813	1	0.009051
1	dbsnp	rs815731	G	A	ALLELIC	17/5	22/28	6.813	1	0.009051
1	dbsnp	rs1320748	G	A	ALLELIC	17/5	22/28	6.813	1	0.009051
1	dbsnp	rs10864219	G	A	ALLELIC	5/17	28/22	6.813	1	0.009051
1	dbsnp	rs4658558	G	A	ALLELIC	5/17	28/22	6.813	1	0.009051
2	dbsnp	rs11903092	G	A	ALLELIC	5/17	28/22	6.813	1	0.009051
4	dbsnp	rs4308327	A	G	ALLELIC	5/17	28/22	6.813	1	0.009051
4	dbsnp	rs11722979	C	A	ALLELIC	17/5	22/28	6.813	1	0.009051

4	dbsnp	rs2214389	C	A	ALLELIC	17/5	22/28	6.813	1	0.009051
4	dbsnp	rs6828751	A	G	ALLELIC	5/17	28/22	6.813	1	0.009051
5	dbsnp	rs2974501	A	G	ALLELIC	5/17	28/22	6.813	1	0.009051
5	dbsnp	rs1030206	G	A	ALLELIC	5/17	28/22	6.813	1	0.009051
5	dbsnp	rs1507808	G	A	ALLELIC	17/5	22/28	6.813	1	0.009051
6	dbsnp	rs4715309	G	A	ALLELIC	5/17	28/22	6.813	1	0.009051
6	dbsnp	rs2245410	A	G	ALLELIC	5/17	28/22	6.813	1	0.009051
6	dbsnp	rs12198149	A	G	ALLELIC	5/17	28/22	6.813	1	0.009051
6	dbsnp	rs9351108	A	G	ALLELIC	5/17	28/22	6.813	1	0.009051
6	dbsnp	rs9480440	A	C	ALLELIC	5/17	28/22	6.813	1	0.009051
7	dbsnp	rs9655107	A	G	ALLELIC	5/17	28/22	6.813	1	0.009051
7	dbsnp	rs6963278	A	C	ALLELIC	5/17	28/22	6.813	1	0.009051
8	dbsnp	rs2005212	A	G	ALLELIC	5/17	28/22	6.813	1	0.009051
8	dbsnp	rs2912236	G	A	ALLELIC	5/17	28/22	6.813	1	0.009051
8	dbsnp	rs2309308	A	G	ALLELIC	5/17	28/22	6.813	1	0.009051
9	dbsnp	rs11999408	A	G	ALLELIC	5/17	28/22	6.813	1	0.009051
9	dbsnp	rs2542250	A	G	ALLELIC	5/17	28/22	6.813	1	0.009051
10	dbsnp	rs12413476	A	G	ALLELIC	5/17	28/22	6.813	1	0.009051
10	dbsnp	rs7915813	A	G	ALLELIC	5/17	28/22	6.813	1	0.009051
11	dbsnp	rs931811	G	A	ALLELIC	5/17	28/22	6.813	1	0.009051
11	dbsnp	rs4754550	A	G	ALLELIC	5/17	28/22	6.813	1	0.009051
12	dbsnp	rs10845469	A	G	ALLELIC	5/17	28/22	6.813	1	0.009051
12	dbsnp	rs2264771	G	A	ALLELIC	5/17	28/22	6.813	1	0.009051
13	dbsnp	rs11619235	G	A	ALLELIC	5/17	28/22	6.813	1	0.009051
13	dbsnp	rs7328541	A	G	ALLELIC	17/5	22/28	6.813	1	0.009051
13	dbsnp	rs4374055	A	C	ALLELIC	17/5	22/28	6.813	1	0.009051
13	dbsnp	rs2765058	G	A	ALLELIC	17/5	22/28	6.813	1	0.009051
16	dbsnp	rs4426342	A	G	ALLELIC	17/5	22/28	6.813	1	0.009051
16	dbsnp	rs1532471	G	A	ALLELIC	5/17	28/22	6.813	1	0.009051
17	dbsnp	rs4791020	A	C	ALLELIC	5/17	28/22	6.813	1	0.009051
18	dbsnp	rs6507528	G	A	ALLELIC	5/17	28/22	6.813	1	0.009051
18	dbsnp	rs4564650	A	G	ALLELIC	5/17	28/22	6.813	1	0.009051
18	dbsnp	rs11874653	G	A	ALLELIC	5/17	28/22	6.813	1	0.009051
19	dbsnp	rs12983947	A	C	ALLELIC	5/17	28/22	6.813	1	0.009051
19	dbsnp	rs12610315	A	C	ALLELIC	5/17	28/22	6.813	1	0.009051
20	dbsnp	rs6078306	A	G	ALLELIC	5/17	28/22	6.813	1	0.009051
20	dbsnp	rs6067173	G	A	ALLELIC	5/17	28/22	6.813	1	0.009051
21	dbsnp	rs2837508	A	G	ALLELIC	5/17	28/22	6.813	1	0.009051
21	dbsnp	rs424372	G	A	ALLELIC	5/17	28/22	6.813	1	0.009051
21	dbsnp	rs2837521	G	A	ALLELIC	5/17	28/22	6.813	1	0.009051
21	dbsnp	rs2837529	A	G	ALLELIC	5/17	28/22	6.813	1	0.009051
23	dbsnp	rs1989891	A	G	ALLELIC	17/5	22/28	6.813	1	0.009051
23	dbsnp	rs4825359	A	G	ALLELIC	17/5	22/28	6.813	1	0.009051
23	dbsnp	rs1736673	A	C	ALLELIC	5/17	28/22	6.813	1	0.009051
23	dbsnp	rs1751088	A	G	ALLELIC	5/17	28/22	6.813	1	0.009051
23	dbsnp	rs6635272	A	G	ALLELIC	5/17	28/22	6.813	1	0.009051
23	dbsnp	rs1202911	G	A	ALLELIC	5/17	28/22	6.813	1	0.009051
23	dbsnp	rs1202925	C	A	ALLELIC	5/17	28/22	6.813	1	0.009051

2	dbsnp	rs6740317	A	G	ALLELIC	3/19	22/26	6.811	1	0.009058
6	dbsnp	rs2759263	C	A	ALLELIC	3/19	22/26	6.811	1	0.009058
13	dbsnp	rs9317650	A	C	ALLELIC	3/19	22/26	6.811	1	0.009058
13	dbsnp	rs1808632	A	G	ALLELIC	3/19	22/26	6.811	1	0.009058
1	dbsnp	rs10737541	A	C	ALLELIC	1/21	16/32	6.799	1	0.00912
3	dbsnp	rs1552037	G	A	ALLELIC	1/21	16/32	6.799	1	0.00912
8	dbsnp	rs4255152	G	A	ALLELIC	1/21	16/32	6.799	1	0.00912
16	dbsnp	rs1406814	G	A	ALLELIC	1/21	16/32	6.799	1	0.00912
20	dbsnp	rs4814783	A	G	ALLELIC	1/21	16/32	6.799	1	0.00912
5	dbsnp	rs32358	C	A	ALLELIC	1/13	20/24	6.749	1	0.009379
1	dbsnp	rs1338442	A	G	ALLELIC	8/14	5/43	6.716	1	0.009555
1	dbsnp	rs16847402	G	A	ALLELIC	8/14	5/43	6.716	1	0.009555
1	dbsnp	rs12065882	G	A	ALLELIC	0/20	13/35	6.697	1	0.009658
1	dbsnp	rs4648381	A	G	ALLELIC	7/15	4/46	6.696	1	0.009663
1	dbsnp	rs11807599	A	G	ALLELIC	7/15	4/46	6.696	1	0.009663
1	dbsnp	rs11588698	A	G	ALLELIC	7/15	4/46	6.696	1	0.009663
1	dbsnp	rs6669972	A	G	ALLELIC	7/15	4/46	6.696	1	0.009663
1	dbsnp	rs6588096	A	C	ALLELIC	7/15	4/46	6.696	1	0.009663
1	dbsnp	rs7541833	G	A	ALLELIC	7/15	4/46	6.696	1	0.009663
1	dbsnp	rs2297138	A	G	ALLELIC	7/15	4/46	6.696	1	0.009663
1	dbsnp	rs6677309	C	A	ALLELIC	7/15	4/46	6.696	1	0.009663
1	dbsnp	rs2300747	G	A	ALLELIC	7/15	4/46	6.696	1	0.009663
1	dbsnp	rs6692981	A	G	ALLELIC	7/15	4/46	6.696	1	0.009663
1	dbsnp	rs549191	G	A	ALLELIC	7/15	4/46	6.696	1	0.009663
1	dbsnp	rs1416612	A	G	ALLELIC	7/15	4/46	6.696	1	0.009663
1	dbsnp	rs4426040	A	G	ALLELIC	7/15	4/46	6.696	1	0.009663
1	dbsnp	rs4658413	G	A	ALLELIC	7/15	4/46	6.696	1	0.009663
2	dbsnp	rs6719038	A	G	ALLELIC	7/15	4/46	6.696	1	0.009663
2	dbsnp	rs6431861	G	A	ALLELIC	7/15	4/46	6.696	1	0.009663
2	dbsnp	rs346830	G	A	ALLELIC	7/15	4/46	6.696	1	0.009663
2	dbsnp	rs6713691	G	A	ALLELIC	7/15	4/46	6.696	1	0.009663
2	dbsnp	rs6734939	A	C	ALLELIC	7/15	4/46	6.696	1	0.009663
2	dbsnp	rs7598331	G	A	ALLELIC	7/15	4/46	6.696	1	0.009663
2	dbsnp	rs219545	A	C	ALLELIC	7/15	4/46	6.696	1	0.009663
2	dbsnp	rs6739811	A	G	ALLELIC	7/15	4/46	6.696	1	0.009663
2	dbsnp	rs84181	A	G	ALLELIC	7/15	4/46	6.696	1	0.009663
2	dbsnp	rs227777	G	A	ALLELIC	7/15	4/46	6.696	1	0.009663
2	dbsnp	rs2303599	G	A	ALLELIC	7/15	4/46	6.696	1	0.009663
2	dbsnp	rs10520309	A	G	ALLELIC	7/15	4/46	6.696	1	0.009663
2	dbsnp	rs2188907	A	G	ALLELIC	7/15	4/46	6.696	1	0.009663
2	dbsnp	rs2521930	A	G	ALLELIC	7/15	4/46	6.696	1	0.009663
2	dbsnp	rs9287421	A	G	ALLELIC	7/15	4/46	6.696	1	0.009663
2	dbsnp	rs10497702	G	A	ALLELIC	7/15	4/46	6.696	1	0.009663
2	dbsnp	rs13396978	A	G	ALLELIC	7/15	4/46	6.696	1	0.009663
3	dbsnp	rs1153483	G	A	ALLELIC	7/15	4/46	6.696	1	0.009663
3	dbsnp	rs17050220	A	C	ALLELIC	7/15	4/46	6.696	1	0.009663
3	dbsnp	rs293927	A	G	ALLELIC	7/15	4/46	6.696	1	0.009663
3	dbsnp	rs293926	G	A	ALLELIC	7/15	4/46	6.696	1	0.009663

3	dbsnp	rs4362751	A	G	ALLELIC	7/15	4/46	6.696	1	0.009663
3	dbsnp	rs9816677	A	G	ALLELIC	7/15	4/46	6.696	1	0.009663
3	dbsnp	rs7340540	A	G	ALLELIC	7/15	4/46	6.696	1	0.009663
3	dbsnp	rs992803	A	C	ALLELIC	7/15	4/46	6.696	1	0.009663
3	dbsnp	rs6438884	A	G	ALLELIC	7/15	4/46	6.696	1	0.009663
4	dbsnp	rs16893622	C	A	ALLELIC	7/15	4/46	6.696	1	0.009663
4	dbsnp	rs1378360	A	C	ALLELIC	7/15	4/46	6.696	1	0.009663
4	dbsnp	rs7436142	C	A	ALLELIC	7/15	4/46	6.696	1	0.009663
4	dbsnp	rs12509052	A	G	ALLELIC	7/15	4/46	6.696	1	0.009663
4	dbsnp	rs11935777	G	A	ALLELIC	7/15	4/46	6.696	1	0.009663
4	dbsnp	rs17502284	A	G	ALLELIC	7/15	4/46	6.696	1	0.009663
4	dbsnp	rs4696352	G	A	ALLELIC	7/15	4/46	6.696	1	0.009663
4	dbsnp	rs1504786	A	G	ALLELIC	7/15	4/46	6.696	1	0.009663
4	dbsnp	rs12643033	C	A	ALLELIC	7/15	4/46	6.696	1	0.009663
5	dbsnp	rs545493	G	A	ALLELIC	7/15	4/46	6.696	1	0.009663
5	dbsnp	rs16883128	A	G	ALLELIC	7/15	4/46	6.696	1	0.009663
5	dbsnp	rs4383731	A	G	ALLELIC	7/15	4/46	6.696	1	0.009663
5	dbsnp	rs10076533	G	A	ALLELIC	7/15	4/46	6.696	1	0.009663
5	dbsnp	rs13187028	A	G	ALLELIC	7/15	4/46	6.696	1	0.009663
5	dbsnp	rs923957	A	G	ALLELIC	7/15	4/46	6.696	1	0.009663
5	dbsnp	rs181270	A	G	ALLELIC	7/15	4/46	6.696	1	0.009663
5	dbsnp	rs7702514	A	G	ALLELIC	7/15	4/46	6.696	1	0.009663
5	dbsnp	rs11744593	A	G	ALLELIC	7/15	4/46	6.696	1	0.009663
5	dbsnp	rs12516185	C	A	ALLELIC	7/15	4/46	6.696	1	0.009663
5	dbsnp	rs10035791	A	G	ALLELIC	7/15	4/46	6.696	1	0.009663
5	dbsnp	rs10462978	G	A	ALLELIC	7/15	4/46	6.696	1	0.009663
5	dbsnp	rs2964115	A	G	ALLELIC	7/15	4/46	6.696	1	0.009663
6	dbsnp	rs17139342	A	G	ALLELIC	7/15	4/46	6.696	1	0.009663
6	dbsnp	rs11752390	G	A	ALLELIC	7/15	4/46	6.696	1	0.009663
6	dbsnp	rs9328361	A	G	ALLELIC	7/15	4/46	6.696	1	0.009663
6	dbsnp	rs9383372	G	A	ALLELIC	7/15	4/46	6.696	1	0.009663
6	dbsnp	rs17268697	A	C	ALLELIC	7/15	4/46	6.696	1	0.009663
6	dbsnp	rs16867628	A	G	ALLELIC	7/15	4/46	6.696	1	0.009663
6	dbsnp	rs3734837	A	G	ALLELIC	7/15	4/46	6.696	1	0.009663
6	dbsnp	rs3815081	G	A	ALLELIC	7/15	4/46	6.696	1	0.009663
6	dbsnp	rs2074477	A	G	ALLELIC	7/15	4/46	6.696	1	0.009663
6	dbsnp	rs9380156	G	A	ALLELIC	7/15	4/46	6.696	1	0.009663
6	dbsnp	rs12200371	G	A	ALLELIC	7/15	4/46	6.696	1	0.009663
6	dbsnp	rs532830	G	A	ALLELIC	7/15	4/46	6.696	1	0.009663
6	dbsnp	rs3799792	G	A	ALLELIC	7/15	4/46	6.696	1	0.009663
6	dbsnp	rs964080	G	A	ALLELIC	7/15	4/46	6.696	1	0.009663
6	dbsnp	rs17082894	G	A	ALLELIC	7/15	4/46	6.696	1	0.009663
6	dbsnp	rs9490073	A	G	ALLELIC	7/15	4/46	6.696	1	0.009663
6	dbsnp	rs7754723	G	A	ALLELIC	7/15	4/46	6.696	1	0.009663
6	dbsnp	rs9371587	A	C	ALLELIC	7/15	4/46	6.696	1	0.009663
7	dbsnp	rs13242034	A	G	ALLELIC	7/15	4/46	6.696	1	0.009663
7	dbsnp	rs4263639	A	G	ALLELIC	7/15	4/46	6.696	1	0.009663
7	dbsnp	rs1362360	A	G	ALLELIC	7/15	4/46	6.696	1	0.009663

7	dbsnp	rs6962730	A	G	ALLELIC	7/15	4/46	6.696	1	0.009663
7	dbsnp	rs11763766	A	C	ALLELIC	7/15	4/46	6.696	1	0.009663
7	dbsnp	rs10252442	A	G	ALLELIC	7/15	4/46	6.696	1	0.009663
7	dbsnp	rs28828764	G	A	ALLELIC	7/15	4/46	6.696	1	0.009663
7	dbsnp	rs2135116	G	A	ALLELIC	7/15	4/46	6.696	1	0.009663
7	dbsnp	rs16886849	G	A	ALLELIC	7/15	4/46	6.696	1	0.009663
7	dbsnp	rs17552854	G	A	ALLELIC	7/15	4/46	6.696	1	0.009663
7	dbsnp	rs878992	A	G	ALLELIC	7/15	4/46	6.696	1	0.009663
7	dbsnp	rs744684	C	A	ALLELIC	7/15	4/46	6.696	1	0.009663
7	dbsnp	rs12534057	G	A	ALLELIC	7/15	4/46	6.696	1	0.009663
7	dbsnp	rs1638021	A	G	ALLELIC	7/15	4/46	6.696	1	0.009663
8	dbsnp	rs4735916	A	G	ALLELIC	7/15	4/46	6.696	1	0.009663
8	dbsnp	rs1545702	A	G	ALLELIC	7/15	4/46	6.696	1	0.009663
8	dbsnp	rs1700096	G	A	ALLELIC	7/15	4/46	6.696	1	0.009663
8	dbsnp	rs2003880	A	G	ALLELIC	7/15	4/46	6.696	1	0.009663
8	dbsnp	rs1480696	A	G	ALLELIC	7/15	4/46	6.696	1	0.009663
8	dbsnp	rs11986821	G	A	ALLELIC	7/15	4/46	6.696	1	0.009663
8	dbsnp	rs4257991	G	A	ALLELIC	7/15	4/46	6.696	1	0.009663
8	dbsnp	rs1455573	A	G	ALLELIC	7/15	4/46	6.696	1	0.009663
8	dbsnp	rs920016	C	A	ALLELIC	7/15	4/46	6.696	1	0.009663
8	dbsnp	rs7825271	C	A	ALLELIC	7/15	4/46	6.696	1	0.009663
8	dbsnp	rs4308771	A	C	ALLELIC	7/15	4/46	6.696	1	0.009663
8	dbsnp	rs16906754	A	G	ALLELIC	7/15	4/46	6.696	1	0.009663
8	dbsnp	rs6998323	A	G	ALLELIC	7/15	4/46	6.696	1	0.009663
9	dbsnp	rs17774954	A	G	ALLELIC	7/15	4/46	6.696	1	0.009663
9	dbsnp	rs1880947	A	G	ALLELIC	7/15	4/46	6.696	1	0.009663
9	dbsnp	rs10971207	G	A	ALLELIC	7/15	4/46	6.696	1	0.009663
9	dbsnp	rs10971212	A	G	ALLELIC	7/15	4/46	6.696	1	0.009663
9	dbsnp	rs12380408	G	A	ALLELIC	7/15	4/46	6.696	1	0.009663
9	dbsnp	rs10986270	A	G	ALLELIC	7/15	4/46	6.696	1	0.009663
10	dbsnp	rs2145939	A	C	ALLELIC	7/15	4/46	6.696	1	0.009663
10	dbsnp	rs11009508	A	C	ALLELIC	7/15	4/46	6.696	1	0.009663
10	dbsnp	rs12411643	A	G	ALLELIC	7/15	4/46	6.696	1	0.009663
10	dbsnp	rs16913153	G	A	ALLELIC	7/15	4/46	6.696	1	0.009663
10	dbsnp	rs12412348	G	A	ALLELIC	7/15	4/46	6.696	1	0.009663
10	dbsnp	rs1836034	G	A	ALLELIC	7/15	4/46	6.696	1	0.009663
10	dbsnp	rs7091087	A	G	ALLELIC	7/15	4/46	6.696	1	0.009663
10	dbsnp	rs17105579	A	G	ALLELIC	7/15	4/46	6.696	1	0.009663
10	dbsnp	rs2902548	A	G	ALLELIC	7/15	4/46	6.696	1	0.009663
11	dbsnp	rs7480497	G	A	ALLELIC	7/15	4/46	6.696	1	0.009663
11	dbsnp	rs10836495	A	G	ALLELIC	7/15	4/46	6.696	1	0.009663
11	dbsnp	rs16917433	G	A	ALLELIC	7/15	4/46	6.696	1	0.009663
11	dbsnp	rs3751050	G	A	ALLELIC	7/15	4/46	6.696	1	0.009663
11	dbsnp	rs1486698	G	A	ALLELIC	7/15	4/46	6.696	1	0.009663
11	dbsnp	rs1486681	A	C	ALLELIC	7/15	4/46	6.696	1	0.009663
11	dbsnp	rs10796842	G	A	ALLELIC	7/15	4/46	6.696	1	0.009663
11	dbsnp	rs2154948	A	G	ALLELIC	7/15	4/46	6.696	1	0.009663
11	dbsnp	rs1790544	C	A	ALLELIC	7/15	4/46	6.696	1	0.009663

11	dbsnp	rs12293066	A	G	ALLELIC	7/15	4/46	6.696	1	0.009663
11	dbsnp	rs10895730	G	A	ALLELIC	7/15	4/46	6.696	1	0.009663
11	dbsnp	rs997989	A	G	ALLELIC	7/15	4/46	6.696	1	0.009663
11	dbsnp	rs12291500	G	A	ALLELIC	7/15	4/46	6.696	1	0.009663
12	dbsnp	rs2240512	G	A	ALLELIC	7/15	4/46	6.696	1	0.009663
12	dbsnp	rs2058821	G	A	ALLELIC	7/15	4/46	6.696	1	0.009663
12	dbsnp	rs4764259	A	C	ALLELIC	7/15	4/46	6.696	1	0.009663
12	dbsnp	rs11056858	A	G	ALLELIC	7/15	4/46	6.696	1	0.009663
12	dbsnp	rs1373431	G	A	ALLELIC	7/15	4/46	6.696	1	0.009663
12	dbsnp	rs215351	G	A	ALLELIC	7/15	4/46	6.696	1	0.009663
12	dbsnp	rs12582811	C	A	ALLELIC	7/15	4/46	6.696	1	0.009663
12	dbsnp	rs11174356	G	A	ALLELIC	7/15	4/46	6.696	1	0.009663
12	dbsnp	rs10778409	A	G	ALLELIC	7/15	4/46	6.696	1	0.009663
12	dbsnp	rs12299627	A	C	ALLELIC	7/15	4/46	6.696	1	0.009663
12	dbsnp	rs632650	A	C	ALLELIC	7/15	4/46	6.696	1	0.009663
12	dbsnp	rs11615997	A	G	ALLELIC	7/15	4/46	6.696	1	0.009663
13	dbsnp	rs636437	A	G	ALLELIC	7/15	4/46	6.696	1	0.009663
13	dbsnp	rs1373905	C	A	ALLELIC	7/15	4/46	6.696	1	0.009663
13	dbsnp	rs9541597	G	A	ALLELIC	7/15	4/46	6.696	1	0.009663
13	dbsnp	rs9544762	A	C	ALLELIC	7/15	4/46	6.696	1	0.009663
13	dbsnp	rs7997669	A	G	ALLELIC	7/15	4/46	6.696	1	0.009663
13	dbsnp	rs7986168	A	G	ALLELIC	7/15	4/46	6.696	1	0.009663
13	dbsnp	rs4772140	G	A	ALLELIC	7/15	4/46	6.696	1	0.009663
13	dbsnp	rs8000393	A	G	ALLELIC	7/15	4/46	6.696	1	0.009663
13	dbsnp	rs7998733	G	A	ALLELIC	7/15	4/46	6.696	1	0.009663
13	dbsnp	rs17357162	A	G	ALLELIC	7/15	4/46	6.696	1	0.009663
13	dbsnp	rs12861680	A	G	ALLELIC	7/15	4/46	6.696	1	0.009663
13	dbsnp	rs2147152	A	G	ALLELIC	7/15	4/46	6.696	1	0.009663
13	dbsnp	rs7986145	A	G	ALLELIC	7/15	4/46	6.696	1	0.009663
14	dbsnp	rs229594	G	A	ALLELIC	7/15	4/46	6.696	1	0.009663
14	dbsnp	rs17103483	A	G	ALLELIC	7/15	4/46	6.696	1	0.009663
14	dbsnp	rs12434141	A	G	ALLELIC	7/15	4/46	6.696	1	0.009663
15	dbsnp	rs2196213	C	A	ALLELIC	7/15	4/46	6.696	1	0.009663
15	dbsnp	rs8041011	G	A	ALLELIC	7/15	4/46	6.696	1	0.009663
15	dbsnp	rs11072070	G	A	ALLELIC	7/15	4/46	6.696	1	0.009663
15	dbsnp	rs3784659	G	A	ALLELIC	7/15	4/46	6.696	1	0.009663
16	dbsnp	rs11644598	G	A	ALLELIC	7/15	4/46	6.696	1	0.009663
16	dbsnp	rs7193100	A	G	ALLELIC	7/15	4/46	6.696	1	0.009663
16	dbsnp	rs7189823	A	C	ALLELIC	7/15	4/46	6.696	1	0.009663
16	dbsnp	rs16947560	A	G	ALLELIC	7/15	4/46	6.696	1	0.009663
16	dbsnp	rs6540007	A	G	ALLELIC	7/15	4/46	6.696	1	0.009663
17	dbsnp	rs16951710	A	G	ALLELIC	7/15	4/46	6.696	1	0.009663
17	dbsnp	rs735176	G	A	ALLELIC	7/15	4/46	6.696	1	0.009663
17	dbsnp	rs3744365	C	A	ALLELIC	7/15	4/46	6.696	1	0.009663
17	dbsnp	rs16967150	A	G	ALLELIC	7/15	4/46	6.696	1	0.009663
17	dbsnp	rs17744461	G	A	ALLELIC	7/15	4/46	6.696	1	0.009663
17	dbsnp	rs11870323	A	G	ALLELIC	7/15	4/46	6.696	1	0.009663
17	dbsnp	rs17745049	A	G	ALLELIC	7/15	4/46	6.696	1	0.009663

17	dbsnp	rs2041396	G	A	ALLELIC	7/15	4/46	6.696	1	0.009663
17	dbsnp	rs4789501	C	A	ALLELIC	7/15	4/46	6.696	1	0.009663
17	dbsnp	rs7212265	A	G	ALLELIC	7/15	4/46	6.696	1	0.009663
18	dbsnp	rs1125425	A	G	ALLELIC	7/15	4/46	6.696	1	0.009663
18	dbsnp	rs2852306	G	A	ALLELIC	7/15	4/46	6.696	1	0.009663
18	dbsnp	rs7237137	A	C	ALLELIC	7/15	4/46	6.696	1	0.009663
18	dbsnp	rs4396615	G	A	ALLELIC	7/15	4/46	6.696	1	0.009663
19	dbsnp	rs12460069	A	G	ALLELIC	7/15	4/46	6.696	1	0.009663
19	dbsnp	rs12460946	A	G	ALLELIC	7/15	4/46	6.696	1	0.009663
19	dbsnp	rs12462540	G	A	ALLELIC	7/15	4/46	6.696	1	0.009663
20	dbsnp	rs6132156	A	C	ALLELIC	7/15	4/46	6.696	1	0.009663
20	dbsnp	rs16980823	G	A	ALLELIC	7/15	4/46	6.696	1	0.009663
20	dbsnp	rs4813431	G	A	ALLELIC	7/15	4/46	6.696	1	0.009663
20	dbsnp	rs6114006	A	G	ALLELIC	7/15	4/46	6.696	1	0.009663
20	dbsnp	rs6060617	A	C	ALLELIC	7/15	4/46	6.696	1	0.009663
20	dbsnp	rs2232571	G	A	ALLELIC	7/15	4/46	6.696	1	0.009663
20	dbsnp	rs6020238	G	A	ALLELIC	7/15	4/46	6.696	1	0.009663
21	dbsnp	rs12482125	A	G	ALLELIC	7/15	4/46	6.696	1	0.009663
21	dbsnp	rs2836437	G	A	ALLELIC	7/15	4/46	6.696	1	0.009663
21	dbsnp	rs7277942	A	G	ALLELIC	7/15	4/46	6.696	1	0.009663
22	dbsnp	rs5765930	A	G	ALLELIC	7/15	4/46	6.696	1	0.009663
22	dbsnp	rs11090995	A	G	ALLELIC	7/15	4/46	6.696	1	0.009663
23	dbsnp	rs11797752	A	G	ALLELIC	7/15	4/46	6.696	1	0.009663
23	dbsnp	rs630169	G	A	ALLELIC	7/15	4/46	6.696	1	0.009663
23	dbsnp	rs12838662	C	A	ALLELIC	7/15	4/46	6.696	1	0.009663
23	dbsnp	rs5927914	A	C	ALLELIC	7/15	4/46	6.696	1	0.009663
23	dbsnp	rs3203642	A	G	ALLELIC	7/15	4/46	6.696	1	0.009663
23	dbsnp	rs5924439	G	A	ALLELIC	7/15	4/46	6.696	1	0.009663
23	dbsnp	rs2524594	A	G	ALLELIC	7/15	4/46	6.696	1	0.009663
23	dbsnp	rs2360134	A	G	ALLELIC	7/15	4/46	6.696	1	0.009663
23	dbsnp	rs5976862	C	A	ALLELIC	7/15	4/46	6.696	1	0.009663
23	dbsnp	rs7066569	G	A	ALLELIC	7/15	4/46	6.696	1	0.009663
1	dbsnp	rs1985652	G	A	ALLELIC	4/16	27/23	6.693	1	0.009678
1	dbsnp	rs12033518	G	A	ALLELIC	4/16	27/23	6.693	1	0.009678
1	dbsnp	rs10798572	A	G	ALLELIC	4/16	27/23	6.693	1	0.009678
2	dbsnp	rs6432157	A	C	ALLELIC	4/16	27/23	6.693	1	0.009678
2	dbsnp	rs2570479	A	C	ALLELIC	16/4	23/27	6.693	1	0.009678
2	dbsnp	rs1424760	G	A	ALLELIC	4/16	27/23	6.693	1	0.009678
2	dbsnp	rs4234047	A	G	ALLELIC	4/16	27/23	6.693	1	0.009678
3	dbsnp	rs6809297	A	G	ALLELIC	4/16	27/23	6.693	1	0.009678
3	dbsnp	rs9869432	G	A	ALLELIC	4/16	27/23	6.693	1	0.009678
3	dbsnp	rs4452278	A	G	ALLELIC	4/16	27/23	6.693	1	0.009678
3	dbsnp	rs815415	G	A	ALLELIC	4/16	27/23	6.693	1	0.009678
4	dbsnp	rs17601255	C	A	ALLELIC	4/16	27/23	6.693	1	0.009678
4	dbsnp	rs4507403	G	A	ALLELIC	4/16	27/23	6.693	1	0.009678
5	dbsnp	rs12520518	A	C	ALLELIC	4/16	27/23	6.693	1	0.009678
7	dbsnp	rs1018954	A	T	ALLELIC	4/16	27/23	6.693	1	0.009678
7	dbsnp	rs692489	G	A	ALLELIC	4/16	27/23	6.693	1	0.009678

7	dbsnp	rs4540368	A	C	ALLELIC	4/16	27/23	6.693	1	0.009678
9	dbsnp	rs6474855	G	A	ALLELIC	4/16	27/23	6.693	1	0.009678
9	dbsnp	rs7045576	A	G	ALLELIC	4/16	27/23	6.693	1	0.009678
9	dbsnp	rs2301612	C	G	ALLELIC	4/16	27/23	6.693	1	0.009678
9	dbsnp	rs2028002	G	A	ALLELIC	4/16	27/23	6.693	1	0.009678
12	dbsnp	rs28706067	G	A	ALLELIC	4/16	27/23	6.693	1	0.009678
14	dbsnp	rs8007165	A	G	ALLELIC	4/16	27/23	6.693	1	0.009678
15	dbsnp	rs12904384	C	A	ALLELIC	4/16	27/23	6.693	1	0.009678
16	dbsnp	rs1809844	A	G	ALLELIC	4/16	27/23	6.693	1	0.009678
17	dbsnp	rs1122200	G	A	ALLELIC	4/16	27/23	6.693	1	0.009678
18	dbsnp	rs7232000	A	G	ALLELIC	4/16	27/23	6.693	1	0.009678
19	dbsnp	rs8182587	A	G	ALLELIC	4/16	27/23	6.693	1	0.009678
20	dbsnp	rs235028	A	G	ALLELIC	4/16	27/23	6.693	1	0.009678
23	dbsnp	rs12009653	G	A	ALLELIC	16/4	23/27	6.693	1	0.009678
23	dbsnp	rs1337655	G	A	ALLELIC	4/16	27/23	6.693	1	0.009678
2	dbsnp	rs1867868	A	G	ALLELIC	4/16	26/22	6.685	1	0.009725
5	dbsnp	rs2936948	G	A	ALLELIC	4/16	26/22	6.685	1	0.009725
7	dbsnp	rs4595080	G	A	ALLELIC	4/16	26/22	6.685	1	0.009725
13	dbsnp	rs7985903	G	A	ALLELIC	4/16	26/22	6.685	1	0.009725
23	dbsnp	rs35906400	C	A	ALLELIC	4/16	26/22	6.685	1	0.009725
4	dbsnp	rs13142734	G	A	ALLELIC	2/20	19/29	6.679	1	0.009754
8	dbsnp	rs7008434	G	A	ALLELIC	7/11	5/41	6.667	1	0.009821
15	dbsnp	rs8024070	A	G	ALLELIC	7/11	5/41	6.667	1	0.009821
1	dbsnp	rs2811630	A	G	ALLELIC	11/11	10/40	6.655	1	0.009885
1	dbsnp	rs1288521	A	G	ALLELIC	11/11	10/40	6.655	1	0.009885
1	dbsnp	rs2797092	G	A	ALLELIC	11/11	10/40	6.655	1	0.009885
1	dbsnp	rs1445583	C	A	ALLELIC	11/11	10/40	6.655	1	0.009885
1	dbsnp	rs2213635	A	G	ALLELIC	11/11	10/40	6.655	1	0.009885
1	dbsnp	rs6688599	A	G	ALLELIC	11/11	10/40	6.655	1	0.009885
1	dbsnp	rs9430069	G	A	ALLELIC	11/11	10/40	6.655	1	0.009885
1	dbsnp	rs10864040	A	G	ALLELIC	11/11	10/40	6.655	1	0.009885
1	dbsnp	rs4359010	C	A	ALLELIC	11/11	10/40	6.655	1	0.009885
1	dbsnp	rs1416527	G	A	ALLELIC	11/11	10/40	6.655	1	0.009885
1	dbsnp	rs12742834	G	A	ALLELIC	11/11	10/40	6.655	1	0.009885
2	dbsnp	rs11900728	A	G	ALLELIC	11/11	10/40	6.655	1	0.009885
2	dbsnp	rs12713595	G	A	ALLELIC	11/11	10/40	6.655	1	0.009885
2	dbsnp	rs1483333	G	A	ALLELIC	11/11	10/40	6.655	1	0.009885
2	dbsnp	rs10199359	G	A	ALLELIC	11/11	10/40	6.655	1	0.009885
2	dbsnp	rs729666	A	G	ALLELIC	11/11	10/40	6.655	1	0.009885
2	dbsnp	rs11682330	G	A	ALLELIC	11/11	10/40	6.655	1	0.009885
2	dbsnp	rs10497039	A	G	ALLELIC	11/11	10/40	6.655	1	0.009885
2	dbsnp	rs4664695	G	A	ALLELIC	11/11	10/40	6.655	1	0.009885
2	dbsnp	rs12624082	G	A	ALLELIC	11/11	10/40	6.655	1	0.009885
2	dbsnp	rs4148797	G	A	ALLELIC	11/11	10/40	6.655	1	0.009885
2	dbsnp	rs17732729	A	C	ALLELIC	11/11	10/40	6.655	1	0.009885
3	dbsnp	rs12633231	A	G	ALLELIC	11/11	10/40	6.655	1	0.009885
3	dbsnp	rs17665280	G	A	ALLELIC	11/11	10/40	6.655	1	0.009885
3	dbsnp	rs4074259	A	C	ALLELIC	11/11	10/40	6.655	1	0.009885

3	dbsnp	rs10936678	A	G	ALLELIC	11/11	10/40	6.655	1	0.009885
4	dbsnp	rs11947258	A	G	ALLELIC	11/11	10/40	6.655	1	0.009885
4	dbsnp	rs2028315	G	A	ALLELIC	11/11	10/40	6.655	1	0.009885
4	dbsnp	rs7657354	A	G	ALLELIC	11/11	10/40	6.655	1	0.009885
4	dbsnp	rs17051001	A	G	ALLELIC	11/11	10/40	6.655	1	0.009885
5	dbsnp	rs7702532	G	A	ALLELIC	11/11	10/40	6.655	1	0.009885
5	dbsnp	rs2172532	A	G	ALLELIC	11/11	10/40	6.655	1	0.009885
5	dbsnp	rs3094348	G	A	ALLELIC	11/11	10/40	6.655	1	0.009885
5	dbsnp	rs2905184	A	G	ALLELIC	11/11	10/40	6.655	1	0.009885
5	dbsnp	rs1008253	C	A	ALLELIC	11/11	10/40	6.655	1	0.009885
5	dbsnp	rs442392	A	C	ALLELIC	11/11	10/40	6.655	1	0.009885
5	dbsnp	rs294983	A	G	ALLELIC	11/11	10/40	6.655	1	0.009885
5	dbsnp	rs451838	G	A	ALLELIC	11/11	10/40	6.655	1	0.009885
6	dbsnp	rs952578	A	G	ALLELIC	11/11	10/40	6.655	1	0.009885
6	dbsnp	rs13215124	G	A	ALLELIC	11/11	10/40	6.655	1	0.009885
6	dbsnp	rs1330674	C	A	ALLELIC	11/11	10/40	6.655	1	0.009885
6	dbsnp	rs9346917	A	G	ALLELIC	11/11	10/40	6.655	1	0.009885
7	dbsnp	rs161192	C	A	ALLELIC	11/11	10/40	6.655	1	0.009885
7	dbsnp	rs12667860	A	G	ALLELIC	11/11	10/40	6.655	1	0.009885
7	dbsnp	rs12056077	A	G	ALLELIC	11/11	10/40	6.655	1	0.009885
7	dbsnp	rs4726484	A	C	ALLELIC	11/11	10/40	6.655	1	0.009885
8	dbsnp	rs10106998	A	G	ALLELIC	11/11	10/40	6.655	1	0.009885
8	dbsnp	rs1721101	A	G	ALLELIC	11/11	10/40	6.655	1	0.009885
8	dbsnp	rs359824	A	G	ALLELIC	11/11	10/40	6.655	1	0.009885
8	dbsnp	rs10101821	G	A	ALLELIC	11/11	10/40	6.655	1	0.009885
8	dbsnp	rs13252107	G	A	ALLELIC	11/11	10/40	6.655	1	0.009885
8	dbsnp	rs2047962	A	G	ALLELIC	11/11	10/40	6.655	1	0.009885
9	dbsnp	rs6476808	G	A	ALLELIC	11/11	10/40	6.655	1	0.009885
9	dbsnp	rs10975152	A	C	ALLELIC	11/11	10/40	6.655	1	0.009885
9	dbsnp	rs7037454	A	C	ALLELIC	11/11	10/40	6.655	1	0.009885
10	dbsnp	rs2060138	G	A	ALLELIC	11/11	10/40	6.655	1	0.009885
10	dbsnp	rs816318	A	G	ALLELIC	11/11	10/40	6.655	1	0.009885
10	dbsnp	rs1327000	G	A	ALLELIC	11/11	10/40	6.655	1	0.009885
10	dbsnp	rs7087184	A	G	ALLELIC	11/11	10/40	6.655	1	0.009885
11	dbsnp	rs11033352	G	A	ALLELIC	11/11	10/40	6.655	1	0.009885
11	dbsnp	rs1355052	C	A	ALLELIC	11/11	10/40	6.655	1	0.009885
11	dbsnp	rs10838121	G	A	ALLELIC	11/11	10/40	6.655	1	0.009885
11	dbsnp	rs1488665	A	C	ALLELIC	11/11	10/40	6.655	1	0.009885
11	dbsnp	rs7106294	A	G	ALLELIC	11/11	10/40	6.655	1	0.009885
12	dbsnp	rs10734694	C	A	ALLELIC	11/11	10/40	6.655	1	0.009885
12	dbsnp	rs860447	A	G	ALLELIC	11/11	10/40	6.655	1	0.009885
12	dbsnp	rs4913296	A	G	ALLELIC	11/11	10/40	6.655	1	0.009885
12	dbsnp	rs7960337	G	A	ALLELIC	11/11	10/40	6.655	1	0.009885
12	dbsnp	rs11068674	A	G	ALLELIC	11/11	10/40	6.655	1	0.009885
12	dbsnp	rs2555298	A	G	ALLELIC	11/11	10/40	6.655	1	0.009885
13	dbsnp	rs2802509	G	A	ALLELIC	11/11	10/40	6.655	1	0.009885
13	dbsnp	rs9590039	G	A	ALLELIC	11/11	10/40	6.655	1	0.009885
13	dbsnp	rs9516436	C	A	ALLELIC	11/11	10/40	6.655	1	0.009885

14	dbsnp	rs12432962	A	G	ALLELIC	11/11	10/40	6.655	1	0.009885
14	dbsnp	rs2765915	C	A	ALLELIC	11/11	10/40	6.655	1	0.009885
14	dbsnp	rs11625405	G	A	ALLELIC	11/11	10/40	6.655	1	0.009885
15	dbsnp	rs904211	G	A	ALLELIC	11/11	10/40	6.655	1	0.009885
15	dbsnp	rs784417	G	A	ALLELIC	11/11	10/40	6.655	1	0.009885
16	dbsnp	rs2745129	G	A	ALLELIC	11/11	10/40	6.655	1	0.009885
16	dbsnp	rs17200189	G	A	ALLELIC	11/11	10/40	6.655	1	0.009885
16	dbsnp	rs7192266	G	A	ALLELIC	11/11	10/40	6.655	1	0.009885
16	dbsnp	rs7185362	A	G	ALLELIC	11/11	10/40	6.655	1	0.009885
16	dbsnp	rs889574	A	G	ALLELIC	11/11	10/40	6.655	1	0.009885
17	dbsnp	rs63953	G	A	ALLELIC	11/11	10/40	6.655	1	0.009885
17	dbsnp	rs2958934	G	A	ALLELIC	11/11	10/40	6.655	1	0.009885
18	dbsnp	rs1692481	A	G	ALLELIC	11/11	10/40	6.655	1	0.009885
18	dbsnp	rs356874	G	A	ALLELIC	11/11	10/40	6.655	1	0.009885
18	dbsnp	rs9952451	G	A	ALLELIC	11/11	10/40	6.655	1	0.009885
18	dbsnp	rs3852844	A	G	ALLELIC	11/11	10/40	6.655	1	0.009885
18	dbsnp	rs7241176	G	A	ALLELIC	11/11	10/40	6.655	1	0.009885
18	dbsnp	rs12150701	A	G	ALLELIC	11/11	10/40	6.655	1	0.009885
18	dbsnp	rs8099286	C	A	ALLELIC	11/11	10/40	6.655	1	0.009885
18	dbsnp	rs1893833	A	G	ALLELIC	11/11	10/40	6.655	1	0.009885
19	dbsnp	rs8108282	C	A	ALLELIC	11/11	10/40	6.655	1	0.009885
19	dbsnp	rs8101889	G	A	ALLELIC	11/11	10/40	6.655	1	0.009885
20	dbsnp	rs6084900	G	A	ALLELIC	11/11	10/40	6.655	1	0.009885
20	dbsnp	rs911148	C	A	ALLELIC	11/11	10/40	6.655	1	0.009885
21	dbsnp	rs2823093	A	G	ALLELIC	11/11	10/40	6.655	1	0.009885
22	dbsnp	rs131849	G	A	ALLELIC	11/11	10/40	6.655	1	0.009885
23	dbsnp	rs5905622	G	A	ALLELIC	11/11	10/40	6.655	1	0.009885
23	dbsnp	rs5906465	G	A	ALLELIC	11/11	10/40	6.655	1	0.009885
23	dbsnp	rs991972	A	G	ALLELIC	11/11	10/40	6.655	1	0.009885
23	dbsnp	rs5980419	G	A	ALLELIC	11/11	10/40	6.655	1	0.009885
1	dbsnp	rs6603802	A	G	ALLELIC	6/14	32/18	6.655	1	0.00989
1	dbsnp	rs3118058	A	G	ALLELIC	14/6	18/32	6.655	1	0.00989
1	dbsnp	rs1028737	A	G	ALLELIC	14/6	18/32	6.655	1	0.00989
1	dbsnp	rs6675978	A	G	ALLELIC	14/6	18/32	6.655	1	0.00989
1	dbsnp	rs4971282	A	C	ALLELIC	14/6	18/32	6.655	1	0.00989
2	dbsnp	rs7558186	C	A	ALLELIC	14/6	18/32	6.655	1	0.00989
3	dbsnp	rs267527	G	A	ALLELIC	14/6	18/32	6.655	1	0.00989
3	dbsnp	rs17202056	G	A	ALLELIC	14/6	18/32	6.655	1	0.00989
4	dbsnp	rs2859980	A	G	ALLELIC	14/6	18/32	6.655	1	0.00989
4	dbsnp	rs953014	A	G	ALLELIC	14/6	18/32	6.655	1	0.00989
4	dbsnp	rs5012200	G	A	ALLELIC	14/6	18/32	6.655	1	0.00989
6	dbsnp	rs9380610	C	A	ALLELIC	14/6	18/32	6.655	1	0.00989
6	dbsnp	rs6928440	C	A	ALLELIC	14/6	18/32	6.655	1	0.00989
7	dbsnp	rs3789243	A	G	ALLELIC	14/6	18/32	6.655	1	0.00989
7	dbsnp	rs6969259	G	A	ALLELIC	14/6	18/32	6.655	1	0.00989
7	dbsnp	rs6966416	A	G	ALLELIC	14/6	18/32	6.655	1	0.00989
9	dbsnp	rs2297231	A	G	ALLELIC	14/6	18/32	6.655	1	0.00989
10	dbsnp	rs10998957	G	A	ALLELIC	14/6	18/32	6.655	1	0.00989

10	dbsnp	rs954148	G	A	ALLELIC	14/6	18/32	6.655	1	0.00989
10	dbsnp	rs10788063	A	G	ALLELIC	14/6	18/32	6.655	1	0.00989
11	dbsnp	rs1524143	A	G	ALLELIC	14/6	18/32	6.655	1	0.00989
11	dbsnp	rs7952073	C	A	ALLELIC	14/6	18/32	6.655	1	0.00989
11	dbsnp	rs1508617	G	A	ALLELIC	14/6	18/32	6.655	1	0.00989
12	dbsnp	rs7980567	G	A	ALLELIC	14/6	18/32	6.655	1	0.00989
12	dbsnp	rs12322163	A	G	ALLELIC	14/6	18/32	6.655	1	0.00989
12	dbsnp	rs7295116	A	G	ALLELIC	14/6	18/32	6.655	1	0.00989
13	dbsnp	rs7333781	G	A	ALLELIC	6/14	32/18	6.655	1	0.00989
14	dbsnp	rs6572449	C	A	ALLELIC	14/6	18/32	6.655	1	0.00989
14	dbsnp	rs11621044	A	C	ALLELIC	14/6	18/32	6.655	1	0.00989
16	dbsnp	rs9936111	A	G	ALLELIC	14/6	18/32	6.655	1	0.00989
17	dbsnp	rs9896436	A	G	ALLELIC	14/6	18/32	6.655	1	0.00989
20	dbsnp	rs6059027	G	A	ALLELIC	14/6	18/32	6.655	1	0.00989
20	dbsnp	rs2070317	A	G	ALLELIC	14/6	18/32	6.655	1	0.00989
21	dbsnp	rs2824760	G	A	ALLELIC	14/6	18/32	6.655	1	0.00989
22	dbsnp	rs2401203	A	G	ALLELIC	14/6	18/32	6.655	1	0.00989
1	dbsnp	rs12035857	G	A	ALLELIC	4/16	1/47	6.652	1	0.009902
11	dbsnp	rs1939672	A	G	ALLELIC	4/16	1/47	6.652	1	0.009902
4	dbsnp	rs11937236	G	A	ALLELIC	4/16	22/18	6.652	1	0.009907
7	dbsnp	rs9692468	A	C	ALLELIC	8/10	7/41	6.647	1	0.009934
2	dbsnp	rs1506563	G	A	ALLELIC	11/9	11/37	6.64	1	0.009972
5	dbsnp	rs13188234	A	G	ALLELIC	11/11	9/37	6.64	1	0.009972
7	dbsnp	rs2971760	A	G	ALLELIC	11/9	11/37	6.64	1	0.009972
9	dbsnp	rs461567	A	G	ALLELIC	13/7	15/33	6.639	1	0.009976
1	dbsnp	rs2270701	A	C	ALLELIC	0/22	12/36	6.638	1	0.009983
8	dbsnp	rs17053273	A	G	ALLELIC	0/22	12/36	6.638	1	0.009983
17	dbsnp	rs11868879	A	G	ALLELIC	0/22	12/36	6.638	1	0.009983
20	dbsnp	rs6013905	G	A	ALLELIC	0/22	12/36	6.638	1	0.009983
2	dbsnp	rs10930995	A	G	ALLELIC	5/15	2/46	6.635	1	0.009997
10	dbsnp	rs17145800	G	A	ALLELIC	5/15	2/46	6.635	1	0.009997

Tbl. 9 PLINK-analysis results *Bioinformatic analysis of the Infinium BeadChip data was carried using PLINK (PUTTY Link). Two categories of clusters were formed based on the shunt stenosis caused by neointimal hyperplasia (Group 1= 0-39.9% lumen stenosis (n=26); Group 2 = >40% lumen stenosis (n=5)). A cut-off of 40 % was chosen based on a preliminary analysis suggesting clinical relevance in that a shunt stenosis of greater than 40% was associated with an increased risk for cardiac interventions, such as balloon dilatation or shunt stenting. PLINK calculated the SNPs that significantly differed in the two groups (p-value<0.01, Chi's Square).*
Chr = Chromosome; rs + number = Single-nucleotide polymorphism identification number; ALLELIC = test method; CHISQ = Chi`s-Square; DF = Degrees of freedom
